# Nanoparticle-enhanced proton beam immunoradiotherapy drives immune activation and durable tumor rejection

**DOI:** 10.1172/jci.insight.167749

**Published:** 2023-06-22

**Authors:** Yun Hu, Sébastien Paris, Narayan Sahoo, Genevieve Bertolet, Qi Wang, Qianxia Wang, Hampartsoum B. Barsoumian, Jordan Da Silva, Ailing Huang, Denaha J. Doss, David P. Pollock, Ethan Hsu, Nanez Selene, Claudia S. Kettlun Leyton, Tiffany A. Voss, Fatemeh Masrorpour, Shonik Ganjoo, Carola Leuschner, Jordan T. Pietz, Nahum Puebla-Osorio, Saumil Gandhi, Quynh-Nhu Nguyen, Jing Wang, Maria Angelica Cortez, James W. Welsh

**Affiliations:** 1 Department of Radiation Oncology, The University of Texas MD Anderson Cancer Center, Houston, Texas, USA.; 2 Department of Translational Science, Nanobiotix, Paris, France.; 3 Department of Radiation Physics, and; 4 Department of Bioinformatics and Computational Biology, The University of Texas MD Anderson Cancer Center, Houston, Texas, USA.; 5 Department of Physics and Astronomy, Rice University, Houston, Texas, USA.; 6 Department of Genetics, and; 7 Department of Strategic Communication, The University of Texas MD Anderson Cancer Center, Houston, Texas, USA.

**Keywords:** Therapeutics, Radiation therapy

## Abstract

The combination of radiation therapy (RT) and immunotherapy has emerged as a promising treatment option in oncology. Historically, x-ray radiation (XRT) has been the most commonly used form of RT. However, proton beam therapy (PBT) is gaining recognition as a viable alternative, as it has been shown to produce similar outcomes to XRT while minimizing off-target effects. The effects of PBT on the antitumor immune response have only just begun to be described, and to our knowledge no studies to date have examined the effect of PBT as part of a combinatorial immunoradiotherapeutic strategy. Here, using a 2-tumor model of lung cancer in mice, we show that PBT in tandem with an anti-PD1 antibody substantially reduced growth in both irradiated and unirradiated tumors. This was accompanied by robust activation of the immune response, as evidenced by whole-tumor and single-cell RNA sequencing showing upregulation of a multitude of immune-related transcripts. This response was further significantly enhanced by the injection of the tumor to be irradiated with NBTXR3 nanoparticles. Tumors of mice treated with the triple combination exhibited increased infiltration and activation of cytotoxic immune cells. This triple combination eradicated both tumors in 37.5% of the treated mice and showed robust long-term immunity to cancer.

## Introduction

Immunoradiotherapy (IRT) has recently been evaluated in both preclinical and clinical settings for its ability to produce antitumor immune responses that are both specific and systemic (1–3). Research from our group and others shows that IRT is able to activate antitumor CD8^+^ T cells within the irradiated tumors and facilitate cytotoxic T cell infiltration into unirradiated tumor metastases, thereby expanding the treatment efficacy of localized radiation to distant tumors (4–6). This effect can be augmented by the use of a nanosized radioenhancer (NBTXR3), even going so far as to enable IRT to effectively overcome anti-PD1 antibody (αPD1) resistance, leading to tumor recession outside the radiated site (the so-called “abscopal effect”) in an αPD1-resistant murine lung cancer model (4). Recent clinical data suggest a potential enhancement of IRT-induced tumor regression in αPD1 nonresponders with the use of NBTXR3 (7). These data are of great significance, as most cancer patients do not respond to αPD1 treatment (8).

To date, the radiation component of most of these studies, including ours, has consisted of XRT. Proton beam therapy (PBT) has emerged relatively recently as a promising alternative to XRT (9, 10). PBT is capable of depositing most of the beam energy into the tumor volume and significantly reduces damage to the surrounding healthy tissues because of its unique Brag peak (11). This is particularly beneficial to pediatric tumor patients and those with tumors close to vital organs. Despite these benefits, research involving PBT has rarely been reported, mainly due to the limited number of proton centers around the world (12). Encouragingly, tremendous resources have been invested in building new proton centers worldwide in the past decade, and consequently, PBT has become more available to patients undergoing RT. In theory, PBT is superior to XRT in generating antitumor immune responses, as PBT results in less radiation-induced lymphopenia due to the reduced exposure of immune cells in the blood, lymph nodes, spleen, and bone marrow to radiation (13, 14). In addition, studies suggest that PBT’s higher linear energy transfer may directly benefit antitumor immune activity (15). Therefore, exploring the treatment efficacy of PBT-based IRT is of great clinical interest.

Recent studies have suggested that high-Z (atomic number) metal nanoparticles have great potential to serve as radioenhancers in PBT, just as they have for XRT. Using these nanoparticles in conjunction with PBT has been documented to effectively reduce cancer cell survival (16, 17). However, these studies have not explored the influence of these nanoparticles on the activation of the antitumor immune response. Given the significant immunopotentiating effects of NBTXR3 when combined with XRT previously reported by us and others (4, 5, 18–20), we reasoned that NBTXR3 in concert with PBT would also elicit a robust antitumor immune response and, thereby, enhance control of both irradiated and unirradiated tumors. To test this hypothesis, we explored the treatment efficacy of various combination therapies of NBTXR3, αPD1, and localized PBT in our αPD1-resistant murine lung cancer model. Our results demonstrate 4 significant findings for PBT-mediated IRT: (a) localized PBT was able to produce an abscopal effect, (b) the addition of αPD1 to PBT significantly improved both local tumor control and abscopal effect, (c) integration of NBTXR3 into PBT+αPD1 further enhanced treatment efficacy on both irradiated and unirradiated tumors, and (d) enhanced tumor control coincided with immune activation.

## Results

### The impact of nanoparticle-enhanced proton beam immunoradiotherapy on tumor control and survival.

**Figure 1:**
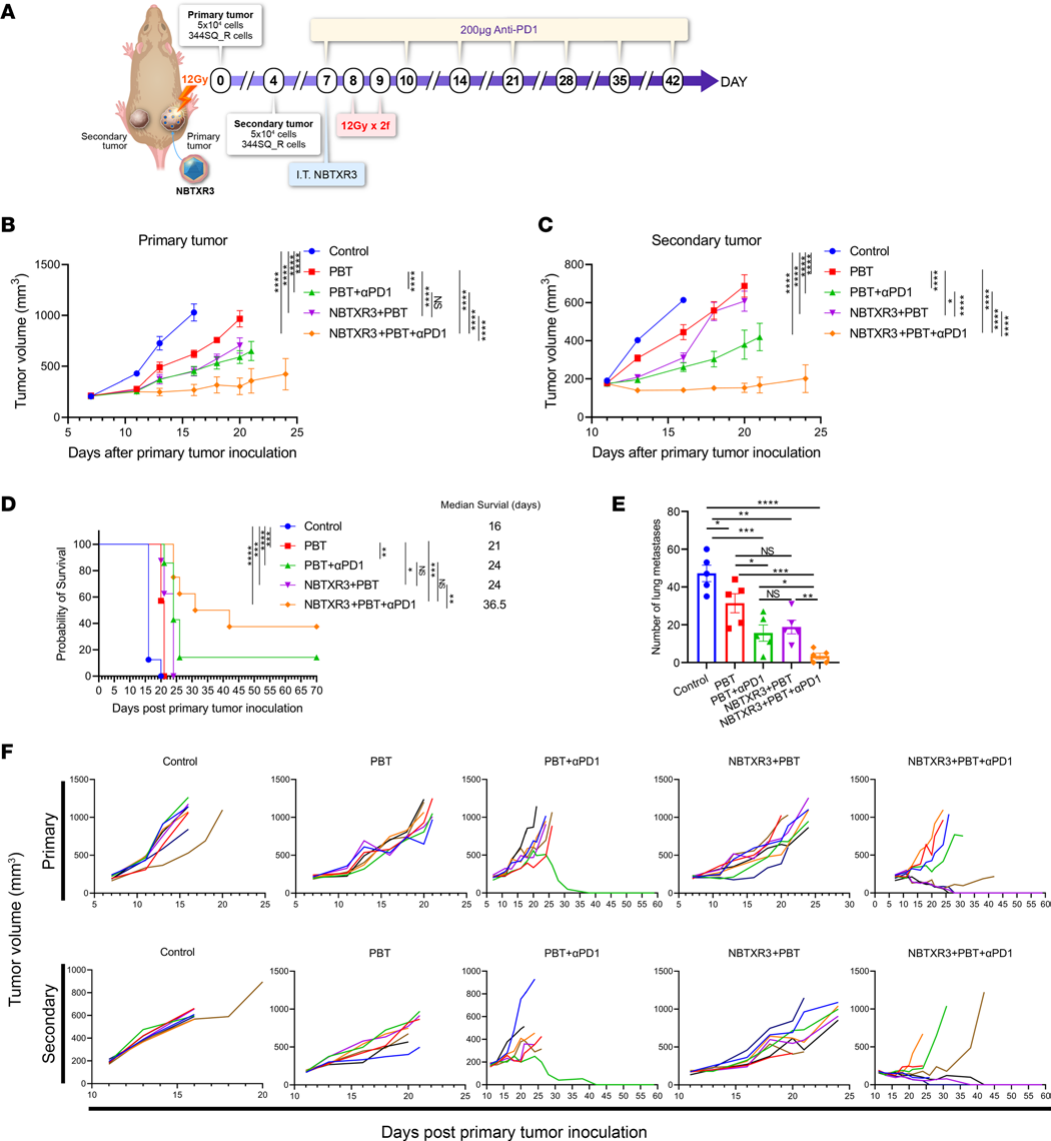
Triple therapy of NBTXR3, PBT, and αPD1 improves primary tumor control, abscopal effect, and survival in αPD1-resistant lung cancer. (**A**) Treatment schema for combination therapies of NBTXR3, PBT, and αPD1. (**B**) Average growth of primary tumors (*n* = 7–8). (**C**) Average growth of secondary tumors (*n* = 7–8). (**D**) Survival rates and median survival times. (**E**) Number of lung metastases on day 19 (*n* = 5). (**F**) Individual tumor growth curves. Eight- to 12-week old 129/SvEv syngeneic female mice were inoculated with 344SQR cells on the right and left legs to establish primary and secondary tumors, respectively. The primary tumors were intratumorally injected with NBTXR3 on day 7, followed by 2 fractions (2f) of 12 Gy proton beam radiation on days 8 and 9. αPD1 (200 μg) was administered to the mice via intraperitoneal injection on days 7, 10, 14, 21, 28, 35, and 42. Tumor volumes were assessed using 2-way ANOVA, while mouse survival rates were examined with the Kaplan-Meier method and compared through log-rank tests. The number of lung metastases was analyzed utilizing 2-tailed *t* tests. Data are expressed as mean ± SEM. *P* < 0.05 was considered statistically significant. *P < 0.05; **P < 0.01; ***P < 0.001; ****P < 0.0001. NS, not significant.

In this study, as illustrated in Figure 1A, we explored whether NBTXR3 nanoparticles, combined with PBT and PD1 blockade, could produce an abscopal effect in αPD1-resistant lung cancer. PBT alone significantly delayed the growth of primary (irradiated) tumors (Figure 1B and Supplemental Figure 1; supplemental material available online with this article; https://doi.org/10.1172/jci.insight.167749DS1). Adding either αPD1 or NBTXR3 nanoparticles to PBT further slowed primary tumor growth to a degree comparable to each other. Interestingly, the triple combination of NBTXR3+PBT+αPD1 resulted in significantly better primary tumor control than the other therapies.

Similar results were observed in the secondary (unirradiated) tumors: PBT alone was able to delay tumor growth; PBT+NBTXR3 significantly improved abscopal effect as compared with PBT; PBT+αPD1 was significantly better in terms of tumor control than NBTXR3+PBT; and NBTXR3+PBT+αPD1 was best of all, with the strongest abscopal effect compared with all other therapies (Figure 1C and Supplemental Figure 1). The superior tumor control of the triple therapy translated to a markedly better survival rate (Figure 1D). The median survival days were 16, 21, 24, 24, and 36.5 days for the control, PBT, PBT+αPD1, NBTXR3+PBT, and NBTXR3+PBT+αPD1 groups, respectively. Notably, 37.5% (3 out 8) of mice in the triple therapy group achieved complete remission in both primary and secondary tumors. In contrast, PBT, NBTXR3+PBT, and PBT+αPD1 led to survival rates of 0%, 0%, and 14.3% (1 out of 7), respectively. To evaluate whether the therapies could reduce the number of spontaneous lung metastases, we harvested lung tissues from the mice on day 19 and counted the lung tumor nodules. The average numbers of lung metastases were the following: control (48 ± 5), PBT (32 ± 6), PBT+αPD1 (16 ± 5), NBTXR3+PBT (19 ± 4), and NBTXR3+PBT+αPD1 (4 ± 2) (Figure 1E). Individual tumor growth curves demonstrated that, although PBT, either alone or in combination with NBTXR3, slowed down the growth of both primary and secondary tumors, tumor growth was not universally eliminated in any treated mice (Figure 1F). However, PBT+αPD1 and NBTXR3+PBT+αPD1 were both able to eliminate primary and secondary tumors in 14.3% and 37.5% of the mice in their respective cohorts, illustrating the superiority of IRT involving PBT.

### NanoString analysis of the primary tumors treated with various combination of NBTXR3, PBT, and αPD1.

**Figure 2:**
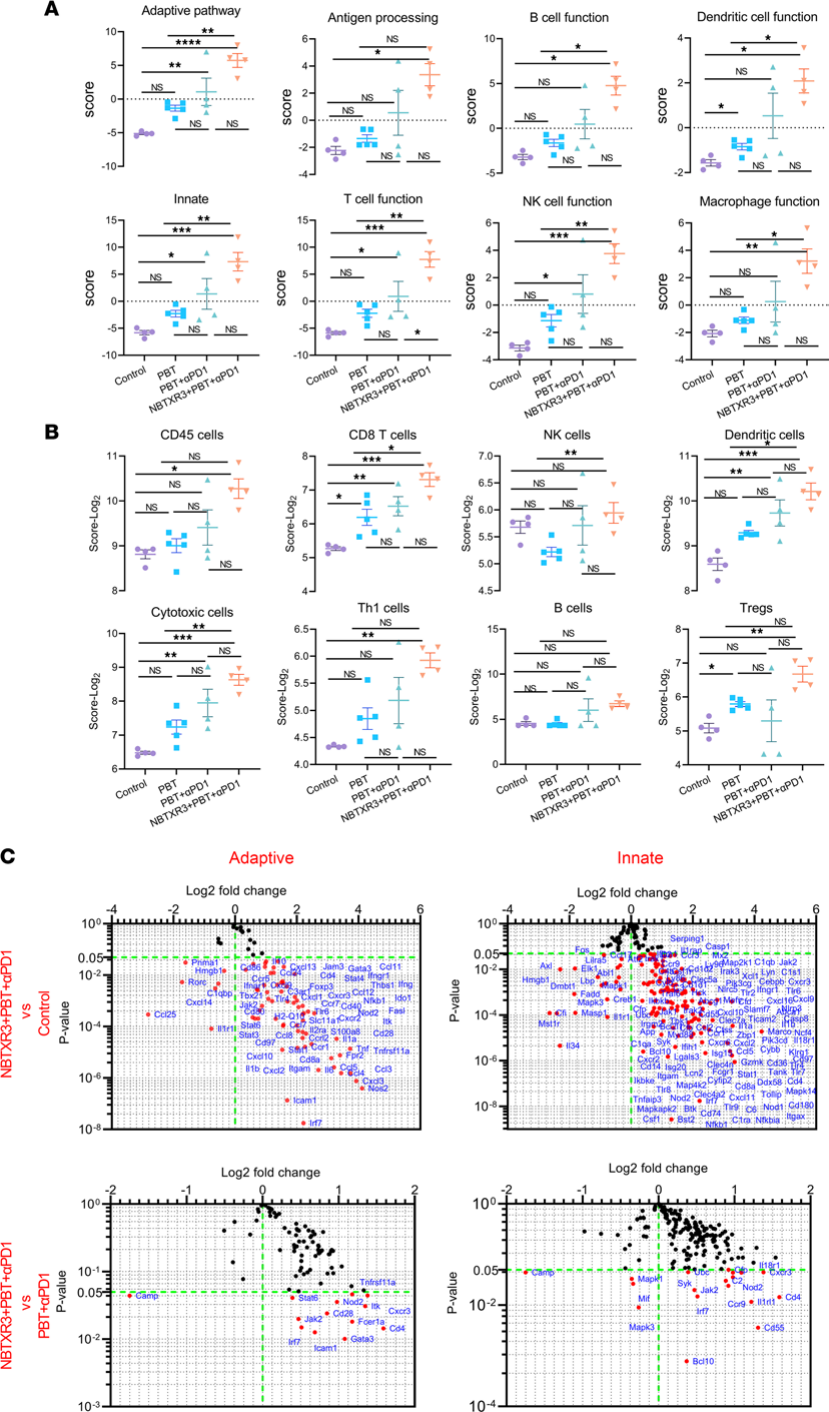
Triple therapy of NBTXR3, PBT, and αPD1 modulates the expression of immune-related genes in favor of antitumor immune response in the irradiated (primary) tumors. (**A**) Activity scores of different immune pathways. (**B**) Relative abundance of immune cell populations. (**C**) Changes in gene expression in adaptive and innate pathways. Primary tumors were harvested from mice (*n* = 4–5) treated with different combinations of NBTXR3, PBT, and αPD1 10 days after irradiation. Total RNAs were extracted from the tumors on day 19, followed by an analysis of immune-related genes with an nCounter PanCancer Immune Profiling Panel. The gene expression data were then analyzed with the PanCancer Immune Profiling Advanced Analysis Module. Activity scores of immune pathways and immune cell abundance were analyzed using either ordinary 1-way ANOVA or the Kruskal-Wallis test. Data are expressed as mean ± SEM. *P* < 0.05 was considered statistically significant. *P < 0.05; **P < 0.01; ***P < 0.001; ****P < 0.0001. NS, not significant.

Having established the efficacy of our respective therapies, we queried the tumor transcriptome in order to gauge changes in the activities of immune pathways induced thereby. RNAs from irradiated (primary) tumors were extracted 10 days after PBT, and the expression of immune-related genes was analyzed with an nCounter PanCancer Immune Profiling Panel. Primary tumors of mice treated with PBT monotherapy exhibited an increase in the activity of several different immune pathways relative to the control, albeit none reached statistical significance (Figure 2A and Supplemental Figure 2A). However, PBT+αPD1 significantly elevated the activities of multiple immune pathways, including the adaptive pathway, innate pathway, T cell function, NK cell function, humoral response, etc. The triple therapy of NBXR3+PBT+αPD1 significantly promoted the activities of all examined immune pathways relative to not just the control, but also PBT alone and markedly (albeit not significantly) increased immune activities relative to PBT+αPD1 dual therapy. Notably, a significant increase in the abundance of CD8^+^ T cells was detected in the tumors treated with PBT, PBT+αPD1, and NBTXR3+PBT+αPD1 compared with the control (Figure 2B).

Moreover, NBTXR3+PBT+αPD1 significantly increased the populations of many types of immune cells, including CD8^+^ T cells, NK cells, dendritic cells (DCs), cytotoxic cells, etc., when compared with PBT monotherapy. However, this was not observed when compared to the tumors treated with PBT+αPD1, despite a marked trend. In addition, the triple therapy also led to a significant increase in the abundance of regulatory T cells (Tregs) compared with the dual therapy of PBT+αPD1 and the control.

Looking at the individual genes that were differentially expressed in response to treatment, the triple therapy increased the expression of many genes involved in both innate and adaptive immunity relative to the control tumors (Figure 2C and Supplemental Table 1). These included genes encoding a litany of chemokines and chemokine receptors, such as *Ccl2*, -*3*, -*4*, -*5*, -*7*, -*8*, -*11*, and -*12*; *Cxcl1*, -*2*, -*9*, -*10*, -*11*, -*13*, and -*16*; *Ccr1*, -*3*, -*5*, -*7*, and -*9*; and *Cxcr2*, -*3*, and -*6*, indicating significant immune mobilization and recruitment to the irradiated tumor site. There was upregulation of several genes associated with inflammation, including *Nlrp3*, *Casp1* and -*8*, *Irak1*, *Traf6*, *Jak1* and -*2*, *Stat1* and -*4*, *Il1a*, *Il1b*, *Il6*, *Ifnar1*, *Ifngr1*, *Irf7*, and *Zbp1*. In keeping with the cell signature data (Figure 2B), multiple T cell signature genes were upregulated, including *Cd4* and *Cd8a*. The T cell costimulatory receptor *Cd28* and its ligands *Cd80* and *Cd86* were also upregulated, indicative of productive T cell stimulation by antigen-presenting cells. The expression of *Gzmk*, a mouse-specific granzyme, was also upregulated, indicating cytotoxic T lymphocyte (CTL) activation. Genes associated with M1 macrophage function were also elevated. These included *Marco*, *Slamf7*, *Myd88*, and *Nos2*, as well as the aforementioned *Cd80* and *Cd86*. Concordantly, *Mst1r*, which is more highly expressed in M2 macrophages (21), was downregulated. Lymphocyte activation was also evidenced by upregulation of various intracellular signaling genes, including *Pik3cd*, *Pik3cg*, *Btk*, *Itk* (also known as LYK), *Hck*, *Lyn*, *Syk*, *Ikbkb*, *Ikbke*, and a host of MAP kinases such as *Mapk14*, *Map2k1* (also known as MEK1), *Map4k2*, and *Mapkapk2*. In addition, multiple components of the complement system, a central mediator of RT-induced tumor-specific immunity (22), were upregulated — specifically, *C1qa*, *C1qb*, *C1ra*, *C1s1*, *C3*, *C3ar1*, *C4b*, and *C6*. We previously observed such an engagement of the complement system following treatment of the same tumor model with NBTXR3+XRT in tandem with checkpoint inhibitors of PD1, LAG3, and TIGIT — a treatment combination that yielded similarly impressive results to the therapy in this study (23). A summary of the top 15 differentially upregulated pathways in both PBT+αPD1 and NBTXR3+PBT+αPD1 relative to control is provided in Supplemental Figure 4A.

When comparing the triple therapy to PBT+αPD1, a much smaller fraction of genes was differentially expressed. Almost all of those were also differentially expressed between the primary tumors in the control group and those treated with the triple therapy (Supplemental Figure 2B). In total, 191 genes were differentially upregulated between the primary tumors of the PBT+αPD1 and control groups; this number jumped to 247 when NBTXR3 was added, 168 of which were shared between PBT+αPD1 and the triple therapy, and 79 of which were new (Supplemental Figure 4B). Only 39 were differentially expressed between the triple therapy and PBT+αPD1 group, and, of these, 38 were also upregulated by either PBT+αPD1 or NBTXR3+PBT+αPD1 relative to the control, indicating that the majority of the effect seen in the triple therapy was due to the combined action of the PBT+αPD1 component. Those genes that were differentially regulated between the triple therapy and PBT+αPD1 included *Cd4*, *Cd28*, *Gata3*, and *Syk*, suggesting that CD4^+^ T cell engagement was specifically enhanced by the presence of the nanoparticles (Figure 2C). *C2*, *Cd55*, and *Cfp* were also upregulated, indicating that NBTXR3 boosted certain components of the complement response. *Camp*, an M2 macrophage marker (24), was very strongly downregulated. Finally, multiple components of the innate inflammatory response were upregulated in the triple therapy relative to PBT+αPD1, including *Il1rl1*, *Il18r1*, *Nod2*, and *Stat6*. Altogether, the differential transcriptome of the tumor microenvironment showed broad inflammatory immune activation within tumors treated with PBT and evidence of T cell priming, the effects of which were further enhanced with the amplification of the radiation signal by the NBTXR3 nanoparticles.

### NanoString analysis of secondary tumors from mice treated with various combinations of NBTXR3, PBT, and αPD1.

**Figure 3:**
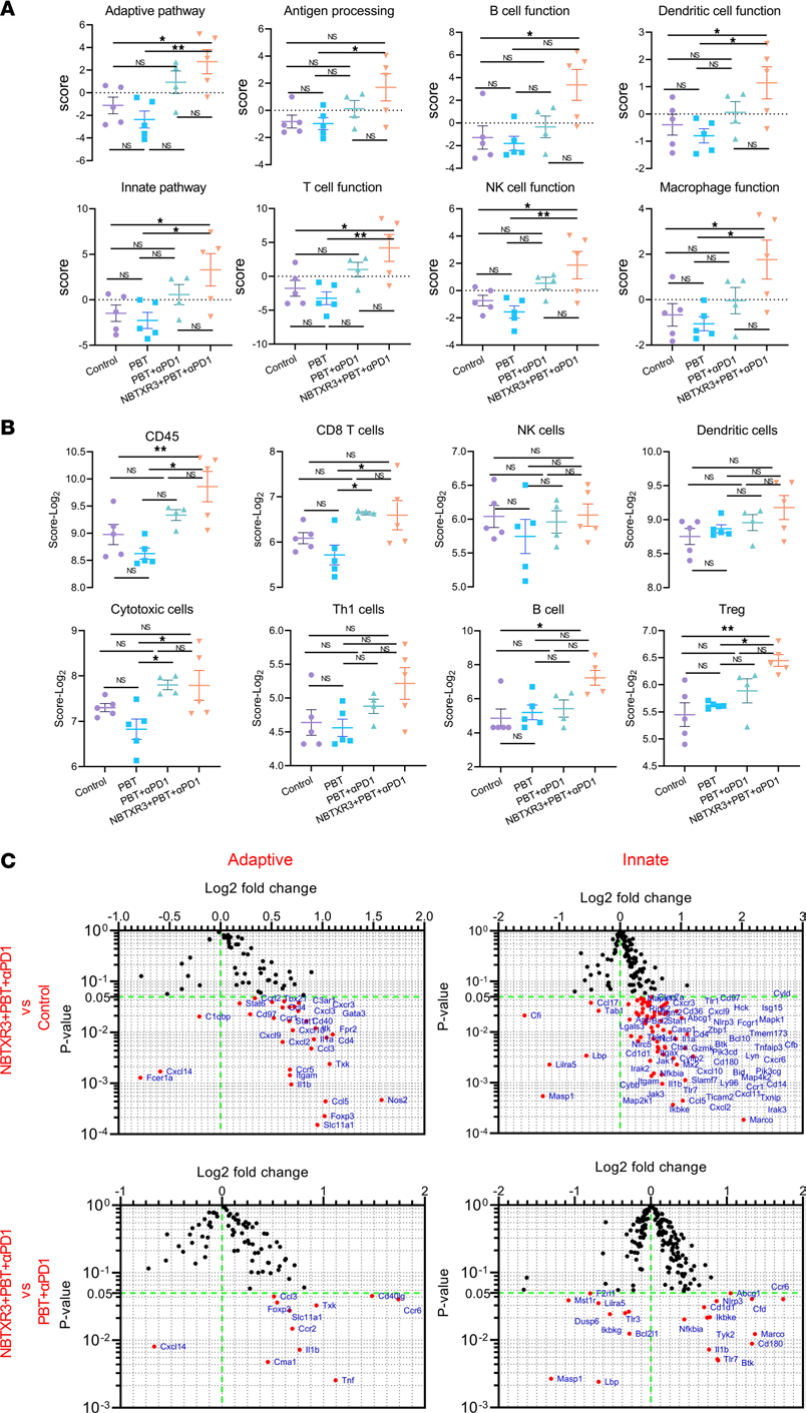
Triple therapy of NBTXR3, PBT, and αPD1 modulates the expression of immune-related genes in favor of antitumor immune response in the unirradiated (secondary) tumors. (**A**) Activity scores of different immune pathways. (**B**) Relative abundance of major immune cell populations. (**C**) Changes in gene expression in adaptive and innate pathways. Secondary tumors were harvested from mice (*n* = 4–5) treated with different combinations of NBTXR3, PBT, and αPD1 10 days after irradiation. Total RNAs were extracted from the tumors, followed by an analysis of immune-related genes with an nCounter PanCancer Immune Profiling Panel. The gene expression data were then analyzed with the PanCancer Immune Profiling Advanced Analysis Module. Activity scores of immune pathways and immune cell abundance were analyzed using either ordinary 1-way ANOVA or the Kruskal-Wallis test. Data are expressed as mean ± SEM. *P* < 0.05 was considered statistically significant. *P < 0.05; **P < 0.01. NS, not significant.

We next applied our RNAseq analysis to the secondary tumors, seeking to elucidate the mechanism underlying the abscopal effect induced by PBT+αPD1 and NBTXR3+PBT+αPD1. RNAs from the unirradiated tumors (harvested from the same mice for the primary tumors) were analyzed with an nCounter PanCancer Immune Profiling Panel. Just as in the irradiated tumors, the activities of several immune pathways — including the adaptive pathway, B cell function, DC function, innate pathway, T cell function, NK cell function, etc. — were all significantly increased in the secondary tumors of mice treated with the triple therapy of NBTXR3+PBT+αPD1 relative to untreated control mice or those treated with PBT monotherapy (Figure 3A and Supplemental Figure 3A). Dual therapy of PBT+αPD1 also increased the activities of a wide range of immune pathways, albeit not significantly. A significant increase in CD8^+^ T cells was also detected in the unirradiated tumors treated with PBT+αPD1 compared with those treated with PBT monotherapy (Figure 3B). This was not significantly increased by the addition of the nanoparticles. However, B cells were significantly enriched in the secondary tumors of triply treated mice relative to the control (Figure 3B).

When examining the individual differentially expressed genes, many of the same genes that had been upregulated in the irradiated tumors were also upregulated in the unirradiated tumors (Figure 3C). These included chemokines (*Ccl3*, -*4*, and -*5*; *Cxcl2*, -*9*, -*10*, and -*11*; *Ccr1* and -*5*; *Cxcr3* and -*6*); inflammatory mediators (*Nlrp3*, *Casp1*, *Il1a*, *Il1b*, *Jak1*, *Stat1*, *Zbp1*); M1 macrophage markers (*Marco*, *Slamf7*, *Nos2*); lymphocyte activation markers (*Btk*, *Hck*, *Pik3cd*, *Pik3cg*, *Mapk1*, *Map2k1*, *Map4k2*); and signs of T cell signaling and function (*Cd4*, *Gzmk*). *Cd8a* was not significantly upregulated; however, *Cd1d1* was, suggesting the involvement of invariant NKT cells at the secondary tumor site instead of more conventional CD8^+^ CTLs. In support of this notion, *Itk* and *Txk* — 2 kinases involved in NKT cell development and function — were also elevated by 90.5% and 109%, respectively, after treatment with NBTXR3+PBT+αPD1. Uniquely to the secondary tumors, *Ripk2*, a kinase involved in inflammatory signaling and necroptosis, was also increased. A summary of the top 15 pathways differentially upregulated in the secondary tumors can be seen in Supplemental Figure 4C.

Comparing transcript expression between unirradiated tumors in mice treated with the triple therapy and those treated with only PBT+αPD1 showed the same trend as observed in the primary tumors (Figure 3C, Supplemental Table 2, Supplemental Figure 3B, and Supplemental Figure 4, C and D). Among those differentially expressed genes were *Mst1r*, the aforementioned M2 marker, and *Bcl2l1*, a potent inhibitor of caspase-mediated cell death. Both were downregulated in the triple therapy mice compared with the PBT+αPD1 group, possibly underscoring the involvement of inflammatory programmed cell death in these tumors. On the other hand, differentially upregulated genes included *Cd40lg*, a potent lymphocyte costimulatory molecule; inflammatory mediators *Il1b*, *Tnf*, and *Nlrp3*; M1 macrophage marker *Marco*; and NKT indicator *Cd1d1*. However, inhibitory molecules such as the Treg transcription factor *Foxp3* and the NF-κB signaling inhibitor *Nfkbia* were also upregulated, suggesting the engagement of homeostatic mechanisms to rein in the inflammatory response. Overall, the immune signature of secondary tumors in treated mice, like that of the primary tumors, strongly indicated inflammatory immune activation possibly involving immunogenic programmed cell death and NKT cell engagement. We also compared immune pathway scores and cell type scores between primary and secondary tumors in the control, PBT+αPD1, and NBTXR3+PBT+αPD1 groups (Supplemental Figure 5). We observed that secondary tumors exhibited more active immune pathways than primary tumors in the control group. In contrast, secondary tumors demonstrated attenuated immune activities compared with primary tumors in both PBT+αPD1 and NBTXR3+PBT+αPD1 groups. In terms of relative cell abundance as indicated by cell type scores, primary tumors had lower abundance of various immune cells compared with secondary tumors. However, treatments with PBT+αPD1 and NBTXR3+PBT+αPD1 reversed this trend for most immune cell types.

### Single-cell analysis of the primary tumors in the treatments with control, PBT+αPD1, and NBTXR3+PBT+αPD1.

We next sought to assess how individual immune cell populations were affected by each treatment combination. To this end, we isolated primary tumors from 5 mice in 3 experimental groups — control, PBT+αPD1, and NBTXR3+PBT+αPD1 — 9 days following irradiation. These tumors were dissociated, and immune cells were isolated via flow sorting. The resulting immunocyte suspensions from each of the 5 mice within each group were pooled and then analyzed with single-cell RNA-seq (scRNA-seq). Using RNA profiling, we identified 16 distinct types of immune cells, which included macrophages, neutrophils, DCs, CTLs, etc. (Supplemental Table 3). We then quantified relative intratumoral abundances for each population within the 3 treatment groups.

**Figure 4:**
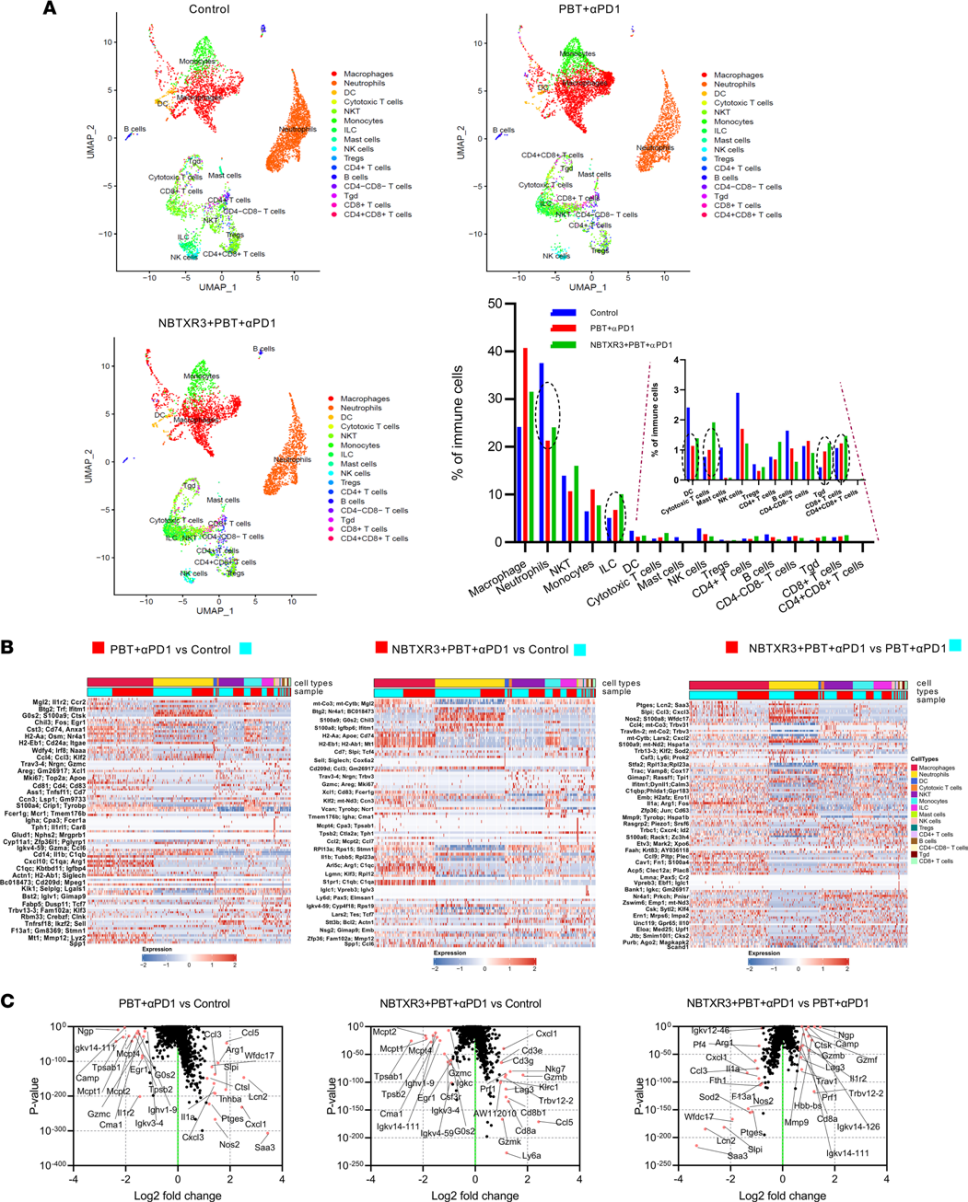
scRNA-seq of the irradiated tumors reveals changes in immune cell populations and distinct gene expression patterns induced by the triple therapy of NBTXR3, PBT, and αPD1. (**A**) UMAP visualization and percentages of immune cell populations in total immune cells. (**B**) Heatmap of differentially expressed genes in immune cell populations after combination therapies. (**C**) Differentially expressed genes in immune cells treated with combination therapies. The irradiated tumors were harvested from mice (*n* = 5) in the control, PBT+αPD1, and NBTXR3+PBT+αPD1 groups 9 days after irradiation. Immune cells (CD45^+^) were sorted from the tumors and analyzed by scRNA-seq. Differentially expressed genes were analyzed using the Kruskal-Wallis test.

We found that the prevalence of neutrophils, DCs, mast cells, NK cells, and B cells declined in the tumors of mice treated with either PBT+αPD1 (by 43.2%, 52.9%, 93.8%, 41.4%, and 35.9%, respectively) or NBTXR3+PBT+αPD1 (by 35.9%, 42.4%, 92.8%, 58.2%, and 63.0%, respectively) relative to those of untreated control mice. In contrast, the percentages of innate lymphoid cells (ILCs), CTLs, γδ T cells, and noncytotoxic CD8^+^ T cells increased after treatment with PBT+αPD1 (by 32.8%, 29.9%, 123.1%, and 14.3% respectively) or NBTXR3+PBT+αPD1 (by 97.2%, 148.8%, 189.2%, and 37.7%, respectively) (Figure 4A). We also found that, as suggested by gene expression data from the secondary tumors (Figure 3C), NKT cells were present and plentiful, although their abundance did not change meaningfully in response to treatment.

Next, we examined the expression of individual genes indicative of our various cell lineages in response to therapy (Figure 4B and Supplemental Figure 6). Collectively, within tumors treated with either PBT+αPD1 or NBTXR3+PBT+αPD1, a myriad of genes were significantly up- or downregulated relative to their levels in cells from untreated tumors. In response to either therapeutic combination, monocytes and macrophages showed strong upregulation of several chemokines, including *Ccl3*, *Ccl4*, *Cxcl1*, *Cxcl2*, and *Cxcl3*, as well as inflammatory cytokines (*Il1a*) and genes involved in reactive oxygen species (ROS) production and lysosomal function, such as *Nos2* and *Ctsl* (Supplemental Figure 6, A and B). On the other hand, genes associated with arginine metabolism (e.g., *Arg1*, *Ass1*), which is often a marker of M2 macrophage function, and *Wfdc17*, a marker of myeloid-derived suppressor cells (MDSCs), were also highly upregulated, indicating engagement of these cohorts as well. In neutrophils, most significantly differentially regulated genes tended to be downregulated, with the most strongly downregulated including many ribosomal protein transcripts and several transcription factors, including *Junb*, *Fos*, *Nr4a1*, and *Grk2*.

In the myeloid populations (monocytes, macrophages, DCs, and neutrophils), the differences between treated and untreated tumors tended to become less pronounced when NBTXR3 nanoparticles were added (Supplemental Figure 6C). On the other hand, the differences in gene expression for cells in the lymphocytic lineages become more pronounced with the addition of NBTXR3. Among the most upregulated genes were also those encoding chemokines (with *Ccl5* being the most abundant), the T cell costimulatory receptor *Icos*, and several coinhibitory receptors, including *Havcr2* (also known as TIM3), *Lag3*, *Pdcd1* (PD1), *Ctla4*, and, in the case of NKT cells, *Klrc1* and *Klrd1*. The upregulation of all these genes raises the possibility of immune suppression, while also signifying immune activation, as checkpoint receptors are upregulated following lymphocyte activation (25) and checkpoint inhibition (26, 27).

We then pulled back our focus from individual cell populations to the top 15 most up- and downregulated transcripts across all immunocyte populations, as defined by all CD45^+^ cells (Figure 4C). In the primary tumors of mice treated with PBT+αPD1, among the most upregulated transcripts were *Arg1* and *Wfdc17*, indicative of M2 macrophages and MDSCs, respectively. On the other hand, *Nos2* and *Il1a*, indicative of M1 macrophages, were also upregulated, suggesting a mix of macrophage subtypes within the primary tumor in the wake of irradiation. In mice treated with NBTXR3+PBT+αPD1, the top upregulated genes were predominated by hallmark indicators of cytolytic lymphocyte activity: *Cd3e*, *Cd3g*, *Cd8a*, *Cd8b1*, *Gzmb*, *Gzmk*, *Nkg7*, *Prf1*, etc. When directly comparing the tumors of mice treated with the triple therapy versus those treated with PBT+αPD1, *Cd8a*, *Gzmb*, and *Prf1* remained significantly elevated, indicating that the upregulation of these genes was the specific result of the nanoparticles. In contrast, among the most downregulated genes of triple therapy versus dual therapy–treated mice were *Wfdc17*, *Arg1*, *Nos2*, and *Il1a*. Altogether, this pattern of up- and downregulation in the triple therapy is suggestive of a shift in immune response from a mixed myeloid response to one characterized by lymphoid cell–mediated cytotoxicity.

**Figure 5:**
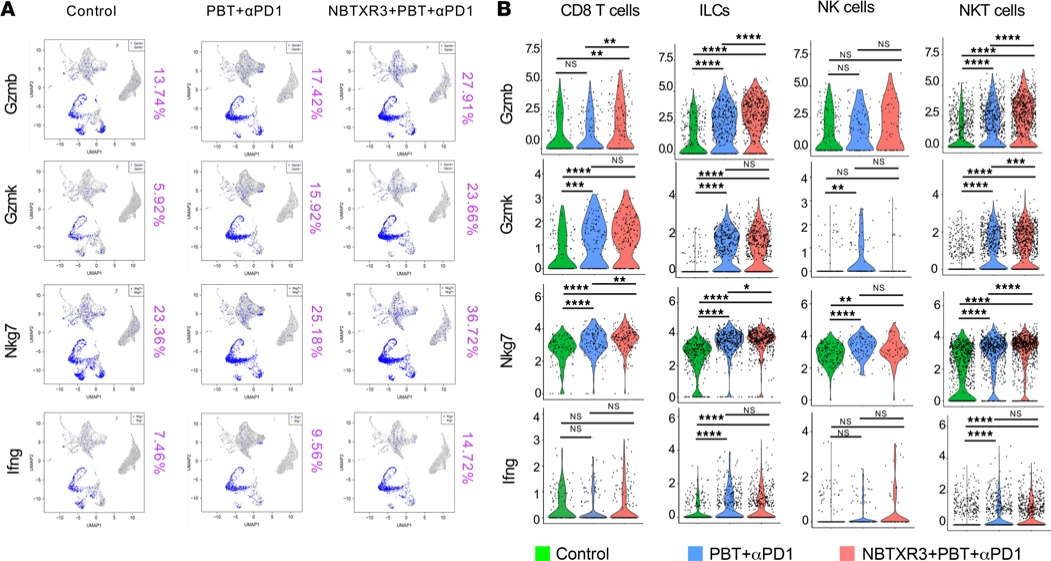
scRNA-seq of the irradiated tumors demonstrates the activation of antitumor lymphocytes induced by the triple therapy of NBTXR3, PBT, and αPD1. (**A**) UMAP color-coded for immune activation markers. (**B**) The expression level of activation markers in cytotoxic effector cells. Expression levels of activation markers were analyzed using the Kruskal-Wallis test. *P* < 0.05 was considered statistically significant. *P < 0.05; **P < 0.01; ***P < 0.001; ****P < 0.0001. NS, not significant.

Given their prominent upregulation in triple therapy–treated tumors, we focused on 4 critical cytotoxic and inflammatory genes: *Gzmb*, *Gzmk*, *Nkg7*, and *Ifng*. Each of these transcripts increased in abundance between the primary tumors of control mice and those of mice treated with PBT+αPD1, and then further magnified with the addition of NBTXR3 (Figure 5A). Using the cell-specific nature of our scRNA-seq analysis, we were able to pinpoint in which cell population(s) expression of these 4 transcripts changed. We found that NKT cells, on the whole, showed the most consistent statistically significant upregulation of all 4 transcripts (Figure 5B), indicating that this population, in addition to being quite numerous within the tumors, also expressed high levels of cytotoxic and proinflammatory genes. ILCs also showed robust upregulation of all 4 transcripts. In fact, *Gzmb* was among the top 10 highest upregulated transcripts in both NKT cells and ILCs for every treatment comparison (PBT+αPD1 vs. Ctrl, NBTXR3+PBT+αPD1 vs. Ctrl, and NBTXR3+PBT+αPD1 vs. PBT+αPD1) (Supplemental Figure 6). CD8^+^ T cells, in comparison, lacked statistically significant upregulation of *Ifng*, and NK cells upregulated only *Nkg7* in response to both treatment combinations.

Among the 16 immunocyte types, macrophages and neutrophils were the most abundant (Figure 4A). Both macrophages and neutrophils have a complicated relationship with cancer. On the one hand, the contribution of both populations to tumorigenesis, progression, immune suppression, and metastasis is extensively documented; however, antitumor activity has also been reported for both (28–31). In light of this functional duality, we sought to delineate what role(s) macrophages and neutrophils played within the tumors of treated and untreated mice.

In keeping with previous reports of large phenotypic heterogeneity within neutrophils (32–35), we identified 14 neutrophil subpopulations (P_N_0–P_N_13) based on their differentially expressed genes (Supplemental Figure 7A). Among them, the subtypes P_N_1, P_N_3, P_N_5, and P_N_7 were elevated in tumors treated with either PBT+αPD1 or NBTXR3+PBT+αPD1 (Supplemental Figure 7B). It is noteworthy that the P_N_7 subtype, which was elevated by both the dual and triple therapies, upregulated multiple genes associated with antitumor function, such as *Ifi47*, an interferon-inducible gene involved in interferon-mediated immunity and cell proliferation (36); and *Gbp5*, *Gbp2b*, *Gbp2*, and *Gbp7*, genes encoding guanylate-binding proteins associated with favorable prognostic outcomes with certain tumors (37, 38). In addition, adding NBTXR3 to PBT+αPD1 further increased the infiltration of P_N_7. However, the P_N_1 subtype was characterized by genes such as *Lilrb4a*, which inhibits both monocyte activation and T cell proliferation, and *Cd9*, which is known to promote metastasis (39–41).

In contrast, the P_N_2, P_N_4, P_N_6, and P_N_8 subtypes were more prevalent in control tumors. Of these, the only subcluster that might have had any evident impact on cancer outcome was subcluster P_N_8, which upregulated genes such as *Ccng1* that encodes cyclin G1, enabling cancer cells to overcome radiation-induced cell cycle arrest (42). The other 3 appeared to have functional specializations, but their role in the tumors or the immune response was unclear.

We also identified 13 different macrophage subpopulations based on their differentially expressed genes (Supplemental Figure 8A). The subclusters P_M_2, P_M_5, P_M_7, and P_M_9 were also the most differentially upregulated in the treated tumors (Supplemental Figure 8B). The P_M_2 cluster upregulated numerous genes associated with antitumor M1 macrophage polarization, such as *Inhba* that encodes the inhibin βA subunit (43), *Nos2* (44), and *Il7r* (45) (Supplemental Figure 8C). The P_M_5 overexpressed genes, including *Gbp5*, *Gbp2*, and *Gbp2b*, are considered signature markers of M1 macrophages (46, 47). P_M_9 upregulated M2 macrophage polarization markers, such as *Gas6* and *Cbr2* (48, 49). In contrast, the subclusters P_M_3, P_M_4, P_M_11, and P_M_12 were downregulated by the treatments.

To evaluate the impact of treatment on T cell specificity, we also interrogated the T cell repertoire by analyzing T cell receptor α and β (TCRα and -β) pairs. In the control group, the top 10 TCR-pair cells accounted for only 28.29% of the entire T cell population. However, in tumors treated with PBT+αPD1 and NBTXR3+PBT+αPD1, the percentages were 55.67% and 57.62%, respectively (Supplemental Figure 9A). In conjunction with a lower inverse Simpson index in the irradiated tumors treated with the triple therapy, these results suggest T cell clonal enrichment (Supplemental Figure 9B).

### Single-cell analysis of the secondary tumors in the treatments with control, PBT+αPD1, and NBTXR3+PBT+αPD1.

**Figure 6:**
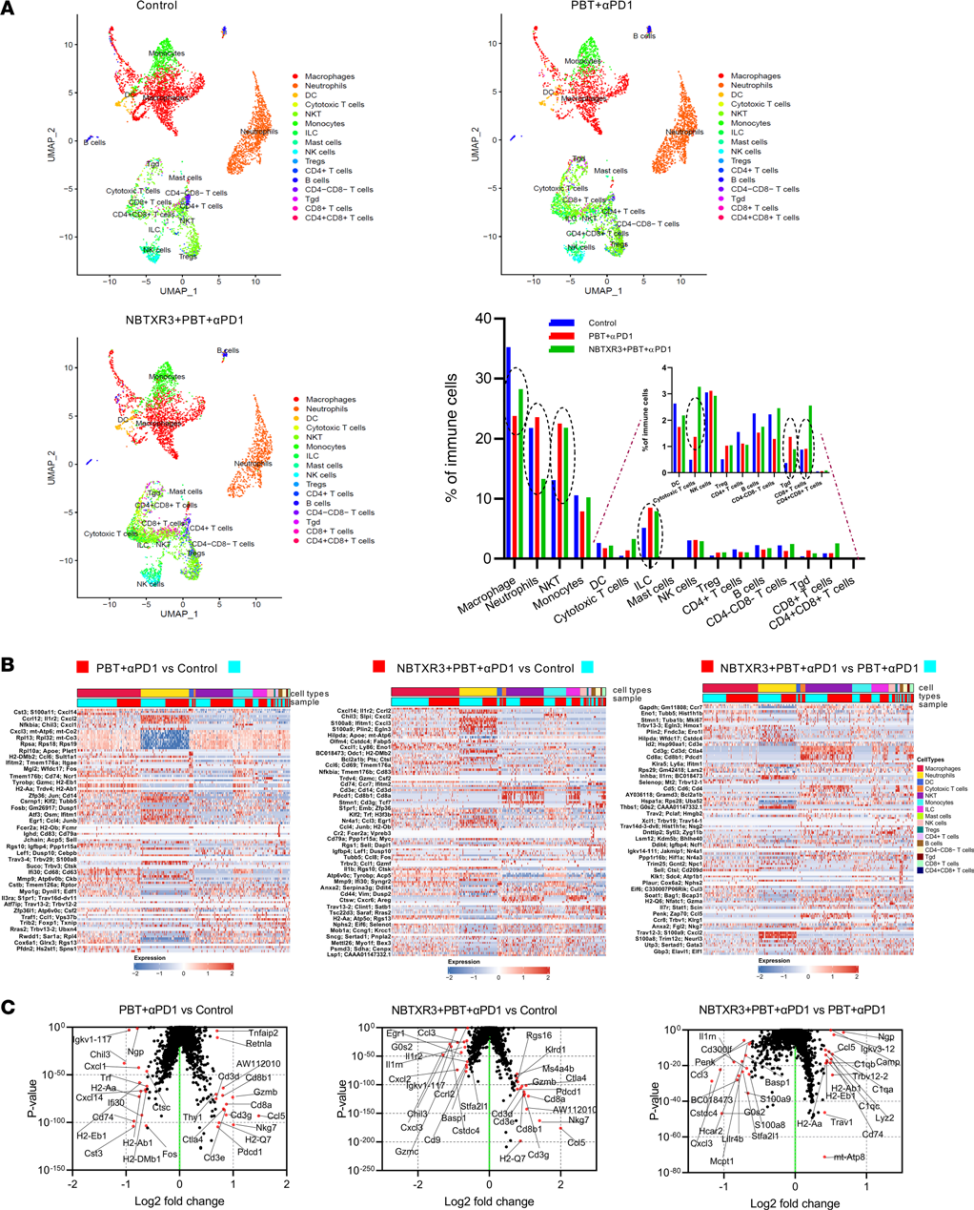
scRNA-seq of the unirradiated tumors uncovers changes in immune cell populations and unique gene expression patterns induced by the triple therapy of NBTXR3, PBT, and αPD1. (**A**) UMAP visualization and percentages of immune cell populations in total immune cells. (**B**) Differentially expressed genes in different immune cell populations after combination therapies. (**C**) Differentially expressed genes in immune cells treated with combination therapies. The unirradiated tumors were harvested from mice (*n* = 5) in the control, PBT+αPD1, and NBTXR3+PBT+αPD1 groups 9 days after irradiation. Immune cells (CD45^+^) were sorted from the tumors and analyzed by scRNA-seq. Differentially expressed genes were analyzed using the Kruskal-Wallis test.

Next, we applied the same scRNA-seq analysis to the unirradiated secondary tumors. Once again, 16 immune cell populations, including macrophages, neutrophils, NKT cells, NK cells, etc. were detected in the unirradiated tumors (Figure 6A). As in the irradiated tumors, ILCs, CTLs, γδ T cells, and CD8^+^ T cells increased in prevalence following treatment with either PBT+αPD1 (by 66.0%, 176.8%, 269.1%, and 3.6%, respectively) or NBTXR3+PBT+αPD1 (by 54.0%, 561.1%, 141.2%, and 190.3%, respectively), while neutrophils and B cells decreased in prevalence. The unirradiated tumors differed in some trajectories, however. Whereas the abundance of NKT cells and Tregs had been more or less unaffected by therapeutic intervention in the primary tumors, in the secondary tumors, intervention with either therapeutic combination led to increased prevalence of both of these populations.

Moreover, some trajectories were opposite to what had been observed in the primary tumors. In the primary tumors, CD4^+^ T cells had increased in prevalence following NBTXR3+PBT+αPD1, and macrophages had increased in response to both therapies. However, in the secondary tumors, both of these cell populations decreased.

We once again examined the expression of individual genes within these cell populations (Figure 6B). Differences between treated and untreated cells tended to be much more pronounced in the secondary tumors than in the primary tumors. As observed in the primary tumors, the overall effect of treatment was a transcriptional upregulation in macrophages (but not in monocytes this time) and transcriptional downregulation in neutrophils (Supplemental Figure 10, A and B). Chemokines once again predominated the upregulated genes in macrophages, making up more than half of the top 15% of transcriptional upregulation. Also highly upregulated were a number of genes involved in various metabolic processes. These included *Cbr2* (carbonyl reductase 2) and *Folr2* (folate receptor β). Exogenous overexpression of carbonyl reductase has been observed to promote necrosis, inflammatory cell infiltration, and apoptotic phagocytosis in ovarian cancer in vivo (50), and FOLR2^+^ macrophages have been reported to associate with CD8^+^ T cell infiltration in human breast cancer (51). On the other hand, another highly upregulated metabolic gene, *Pltp*, has been shown to promote cholesterol accumulation in macrophages (52), which promotes protumoral foam cell development (53, 54) and reduces inflammation (55). The aforementioned MDSC marker, *Wfdc17*, was also highly upregulated — the second-highest upregulated transcript.

As they had been in the primary tumors, neutrophilic transcript levels were depressed in the secondary tumors of treated mice. Once again, among the most depressed transcripts were many ribosomal proteins. Also downregulated were several mitochondrial genes involved in the electron transport chain, indicating a collapse of both protein translation and mitochondrial respiration. The situation for lymphoid cells in the secondary tumors was similar to that in the primary tumors; *Ccl5* and *Ccl4* were upregulated in ILCs and NKT cells, *Icos* was upregulated in CTLs, and checkpoint receptors were upregulated across the board (Supplemental Figure 10, A and B).

We again widened our analysis to interrogate the top 15 up- and downregulated transcripts within the secondary tumors from each of the 3 treatment groups. Unlike what we had observed in the primary tumors, we observed significant upregulation of *Cd3e*, *Cd3d*, *Cd3g*, *Cd8a*, *CD8b1*, *Gzmb*, and *Nkg7* in the secondary tumors of mice treated with PBT+αPD1 (Figure 6C). These genes had only been significantly elevated in primary tumors of mice given NBTXR3+PBT+αPD1, but in the secondary tumors, the addition of NBTXR3 was unnecessary for their upregulation. Each of these genes was also upregulated in the secondary tumors of triple therapy–treated mice relative to untreated controls. However, they were not among the top up- or downregulated genes when the triple therapy was compared to the dual therapy in secondary tumors. This would suggest that, unlike the case in primary tumors, PBT+αPD1 alone is sufficient for cytotoxic lymphocyte engagement in unirradiated tumors. We noted several genes, including *H2-Ab1*, *H2-Eb1*, and *H2-Aa* for major histocompatibility complex class II molecules and *Trav1* for the TCR when examining those differentially upregulated genes in the secondary tumors of triple therapy–treated mice relative to dual therapy. As CD4^+^ T cells were not significantly enriched within the secondary tumors (Figure 6A), we do not believe this increase in MHC class II presentation indicated increased T helper cell recruitment. Notably, in addition to the aforementioned cytotoxicity-associated genes, 2 coinhibitory receptor genes (*Ctla4* and *Pdcd1*) were upregulated in the secondary tumors of triple therapy–treated mice relative to dual therapy (Figure 6C). As in the primary tumor, we infer that their elevation here simultaneously denotes immune activation and the potential for immune suppression.

**Figure 7:**
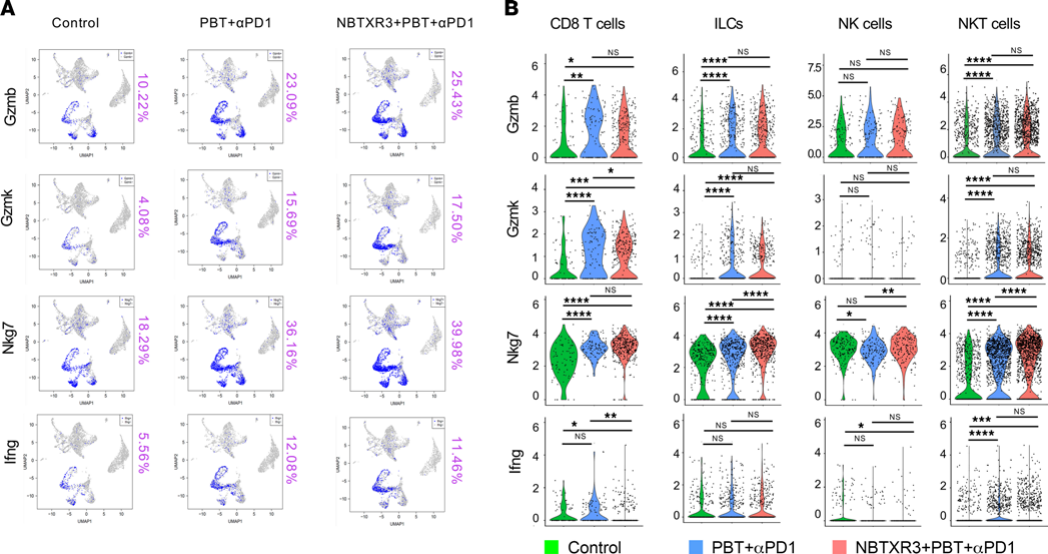
scRNA-seq of the unirradiated tumors demonstrates the activation of antitumor lymphocytes induced by the triple therapy of NBTXR3, PBT, and αPD1. (**A**) UMAP color-coded for immune activation markers. (**B**) The expression level of activation markers in cytotoxic effector cells. Expression levels of activation markers were analyzed using the Kruskal-Wallis test. *P* < 0.05 was considered statistically significant. *P < 0.05; **P < 0.01; ***P < 0.001; ****P < 0.0001. NS, not significant.

We once more looked at the expression of our 4 critical antitumor effector genes: *Gzmb*, *Gzmk*, *Nkg7*, and *Ifng*. As they had in the primary tumors, expression of each of the 4 increased in the secondary tumors of mice treated with PBT+αPD1 (Figure 7A). Unlike in the primary tumors, however, the addition of NBTXR3 only slightly changed their expression. Overall, NKT cells and ILCs once again displayed the most robust increases in each of the 4 transcripts (except for *Ifng*, whose expression was not significantly altered in ILCs following either treatment) (Figure 7B). *Gzmb* and *Gzmk* were again among the top upregulated genes in both NKT cells and ILCs of mice treated with either dual or triple therapy relative to controls, though this was not the case for triple versus dual therapy (Supplemental Figure 10). For CD8^+^ T cells, increases in *Nkg7* were the most robust, although *Gzmb* was statistically more highly expressed in CTLs of triple therapy–treated mice versus those treated with dual therapy. NK cells exhibited only a mild increase in *Nkg7* (which was not statistically significant between the triple therapy and control) and in *Ifng*, which was statistically significant only in the triple therapy versus control. Overall, these data suggest that treatment of mice with PBT+αPD1 substantially increased the expression of critical cytotoxic and inflammatory genes in the secondary and primary tumors of mice. Meanwhile, the effects of NBTXR3 nanoparticles on these particular genes were primarily relegated to the primary tumors.

As in the irradiated tumors, we also identified 14 subclusters of neutrophils (Supplemental Figure 7B). Subgroup abundance tended to be more similar between the treatment and control groups in the secondary tumors than in the primary tumors. The greatest outliers were the P_N_2 and P_N_7 subpopulations, which were elevated in treatment groups, and the P_N_0, P_N_4, and P_N_5 subgroups, whose populations were depressed in the treatment groups relative to the untreated controls. In addition, 13 subclusters of macrophages were also observed in the unirradiated tumors (Supplemental Figure 8B). The P_M_1, P_M_3, and P_M_5 subclusters were distinctly upregulated in the triple therapy but not in the PBT+αPD1 group. The P_M_3 subcluster was notable in that it contained 3 separate genes for CD3: *Cd3d*, -*e*, and -*g*. CD3 is the defining marker of T cells, being the essential intracellular signaling component of the TCR. However, recent reports have indicated that there is a macrophage subpopulation that expresses CD3. These CD3^+^ macrophages are highly phagocytic and proinflammatory (56, 57). The P_M_0 and P_M_4 subclusters were downregulated in the treatment groups. The P_M_11 subcluster, whose population was reduced in the treatment groups relative to the untreated control, upregulated gene markers, including *Mcm6* (58), *Mcm2* (59), and *Mcm7* (60), which are associated with M2 macrophage polarization.

We also analyzed the T cell repertoire in the unirradiated tumors. In the control group, the top 10 TCR-pair cells accounted for only 17% of the T cell population. However, in tumors treated with PBT+αPD1 and NBTXR3+PBT+αPD1, the percentages were 23.70% and 38.87%, respectively (Supplemental Figure 11A), indicating an expansion of a few TCR clonotypes. In addition, a lower inverse Simpson index in the unirradiated tumors treated with the triple therapy demonstrated T cell clonal enrichment (Supplemental Figure 11B).

### Evaluation of memory immune response in the survivor mice treated with triple therapy.

**Figure 8:**
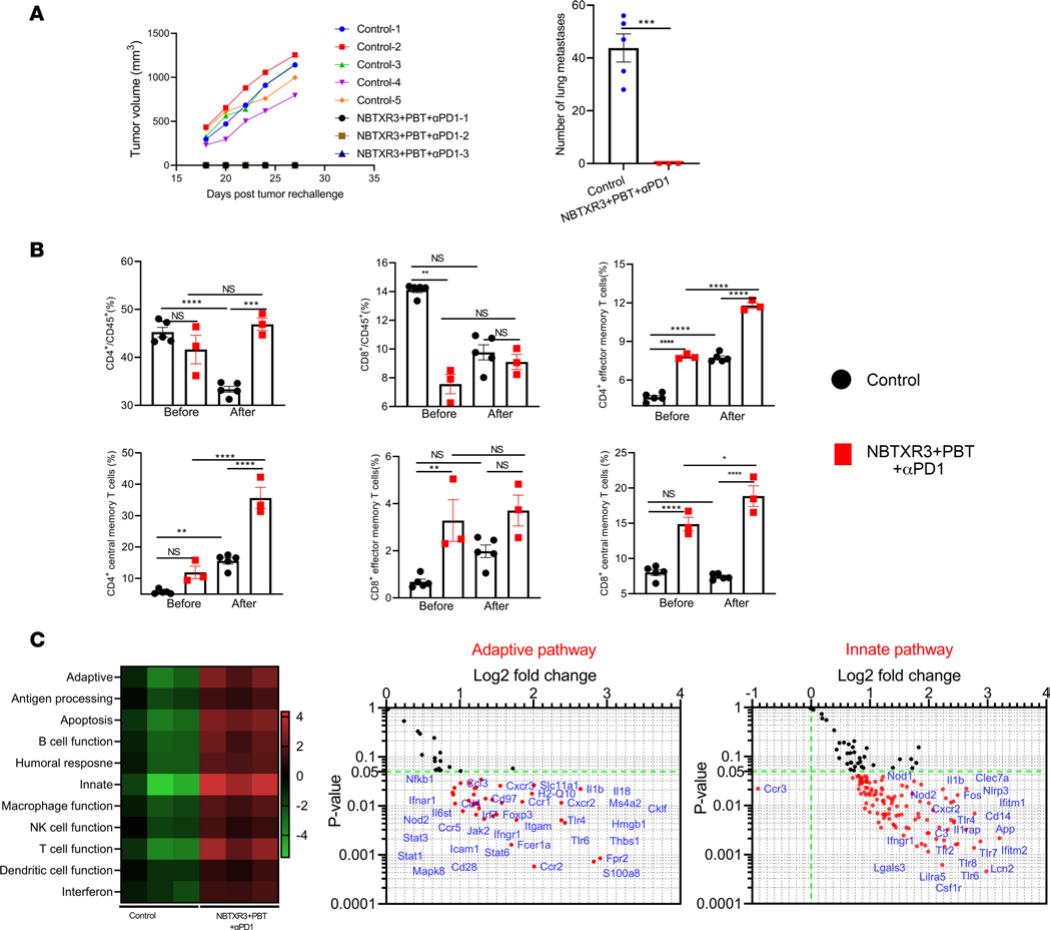
Triple therapy of NBTXR3, PBT, and αPD1 induces antitumor memory immune response in mice. (**A**) Tumor growth and the number of lung metastases in the survivor mice rechallenged with 344SQR cells. (**B**) Blood memory CD4^+^ and CD8^+^ T cells in the survivor mice before and after tumor rechallenge. (**C**) NanoString analysis of the blood immune cell populations 3 days before tumor rechallenge. The survivor mice treated with NBTXR3+PBT+αPD1 were rechallenged with 5 × 10^4^ 344SQR cells in the right flank 67 days after PBT, and the tumor growth was monitored. The memory immune status in the blood was profiled with flow cytometry 3 days before and 19 days after tumor rechallenge. The immune pathway activities in the blood were characterized by NanoString. The number of lung metastases was analyzed utilizing 2-tailed *t* tests. The percentages of blood memory CD4^+^ and CD8^+^ T cells were analyzed using either ordinary 1-way ANOVA or the Kruskal-Wallis test. Data are expressed as mean ± SEM. *P* < 0.05 was considered statistically significant. *P < 0.05; **P < 0.01; ***P < 0.001; ****P < 0.0001. NS, not significant.

Previously, we demonstrated that mice cured by NBTXR3-enhanced IRT developed robust antitumor memory immune responses, which led to complete tumor rejection upon rechallenge (5). Here, we explored whether the triple therapy of NBTXR3+PBT+αPD1 induced a comparable memory response. To determine this, we took mice previously treated with NBTXR3+PBT+αPD1 and that had survived the initial tumor challenge, and we rechallenged them with the same tumor line. As was seen in our previous therapeutic report (5), the survivor mice effectively rejected the 344SQR cells upon rechallenge, while all mice in the control group developed tumors after tumor challenge (Figure 8A). In addition, no lung metastases were detected in any survivor mice after tumor rechallenge. In contrast, all the naive mice developed numerous lung metastases (44 ± 6).

To probe the mechanism underlying this immunity, we collected blood from naive and survivor mice 3 days before and 19 days after rechallenge and queried their circulating memory T cell prevalence via flow cytometry, examining the levels of effector memory (Tem) and central memory (Tcm) T cells. Following tumor insult, naive mice showed a comparable prevalence of CD4^+^ T cells to their survivor counterparts and a significantly higher percentage of CD8^+^ T cells (Figure 8B). Following tumor insult, both the CD4^+^ and CD8^+^ populations had significantly contracted in the naive mice. This was not the case for the survivor mice, with their CD4^+^ and CD8^+^ populations being preserved, perhaps even slightly elevated compared with their prechallenge levels (Figure 8B). Before the rechallenge, the cured survivor mice had significantly higher percentages of CD4^+^ Tem (7.8% ± 0.1% vs. 4.7% ± 0.1%) and CD8^+^ Tem (3.3% ± 0.9% vs. 0.7% ± 0.1%) cells and higher percentages of CD8^+^ Tcm cells than their naive counterparts (Figure 8B and Supplemental Figure 12). This lead was maintained and even extended after tumor rechallenge, with the cured mice maintaining significantly higher percentages of CD4^+^ Tem cells (11.8% ± 0.2% vs. 7.7% ± 0.2%) and CD8^+^ Tcm cells (18.9% ± 1.5% vs. 7.4% ± 0.2%) than the controls. The cured mice also had developed a significantly higher percentage of CD4^+^ Tcm cells (35.6% ± 3.4% vs. 15.6% ± 1.0%). Notably, the treatment group had similar ratios of CD4^+^/CD45^+^ cells before tumor rechallenge; however, the treatment group had a significantly higher ratio of CD4^+^/CD45^+^ cells (46.9% ± 1.3% vs. 33.3% ± 0.6%) after tumor rechallenge, compared with the control group. In addition, there was a significantly higher ratio of CD8^+^/CD45^+^ cells (14.1% ± 0.2% vs. 7.6% ± 0.7%) in the control group than in the treatment group before tumor rechallenge; however, this difference was not observed after tumor rechallenge.

To measure the differences between survivor and naive mice, we interrogated the activities of immune pathways in the blood before tumor rechallenge using NanoString analysis of blood immunocytes (Figure 8C). The survivor mice had elevated activities in pathways involving adaptive response, antigen processing, B cell function, T cell function, innate response, etc., as compared with the control, along with significantly higher expression of a wide range of proinflammatory genes, including *Ifngr1*, *Il1b*, *Nod2*, *Tlr4*, *Tlr2*, *Tlr8*, etc. (Figure 8C and Supplemental Table 4).

## Discussion

In the past decade, increasing clinical and preclinical evidence has indicated that RT combined with immune checkpoint blockade can activate the immune system and safely eradicate irradiated tumors and unirradiated metastases (1, 2). However, almost all of these studies have been based on RT delivered using photons. PBT causes less lymphopenia and fewer immunosuppressive effects than XRT (15); therefore, it represents a promising candidate for eliciting a more robust immune response. In this study, we found that PBT alone slowed the growth of irradiated and unirradiated tumors in an αPD1-resistant lung cancer model. Adding αPD1 further enhanced control of the 2 tumors, indicating that PBT and αPD1 may work synergistically.

Our work here follows up on the seminal study of this topic by Mirjolet and colleagues (61). Like the Mirjolet et al. study, we achieved a 37.5% survival rate in treated mice. We also observed a potent innate immune response and increased tumor infiltration by M1-phenotype macrophages, CD4^+^ and CD8^+^ T cells, NK cells, and Tregs. Many of the genes previously reported to be upregulated following PBT were also elevated in our study. These included *Ccl5*, *Cd40*, *Cfb*, *Gzmb*, *Ifih1*, *Irf7*, *Isg15*, *Klrg1*, *Lcn2*, *Serpinb9b*, and *Tlr9*. Thus, our results confirm the previously described immune effects of PBT and extend them to another tumor type.

Our study builds further on this initial report by showing the abscopal immunostimulatory properties of PBT. Our experimental model uses a 2-tumor system that mimics a metastatic site. One site (the primary tumor) is irradiated, while the other is not. The combination therapies of NBTXR3+PBT+αPD1 and PBT+αPD1 induced recession of not only the irradiated tumors but also the unirradiated tumors. This coincided with the upregulation of a broad variety of genes that are specific to immune cells in both tumors. De novo metastasis was also better controlled in the triple therapy, providing further evidence of systemic tumor control following this therapeutic intervention.

The use of NBTXR3 nanoparticles lends further potential novelty to our study. In vivo studies on the combination of high-Z nanoparticles with PBT are presently limited (17). For the first time to our knowledge, we have demonstrated that high-Z metal nanoparticles represented by NBTXR3 could benefit abscopal tumor control when combined with PBT or PBT+αPD1. The addition of NBTXR3 to PBT+αPD1 enhanced the effects of PBT+αPD1 in systemic immune activation and tumor control.

Our previous studies have demonstrated that NBTX3-mediated XRT can increase CD8^+^ T cell tumor infiltration into tumors (4). Here, we demonstrate that PBT+αPD1 and NBTXR3+PBT+αPD1 were able to increase infiltration of not just CD8^+^ T cells but also macrophages, NKT cells, ILCs, γδ T cells, etc. scRNA-seq confirmed that NBTXR3 increased CD8^+^ T cell infiltration in irradiated and unirradiated tumors when combined with PBT+αPD1. NKT cells, in particular, represented a large fraction of the immune infiltrate within the primary and secondary tumors. NKT cells are a subset of T cells that can recognize lipid antigens and are able to exert potent antitumor response through direct release of cytotoxic granules or through secreting cytokines that induce activation of other effector lymphocytes (62). Tumor immunogenic cell death elicited by PBT likely increases lipid and glycolipid antigen release, leading to elevated NKT cell infiltration. ILCs are a heterogeneous immune cell population that can be divided into 3 groups (ILC1s, ILC2s, and ILC3s) based on the expression of transcription factors and cytokines (63). Depending on the cytokine environment, these ILC subsets can favor or suppress tumor growth (64). The precise role of these cells in our system remains to be determined; however, given the marked increase in inflammatory and cytolytic genes we observed following therapy, our data suggest that these cells positively contributed to the recession of the tumors.

Interestingly, we found that the triple therapy reduced the abundance of neutrophils in the 2 tumors. Tumor-associated neutrophils (TANs) predominantly exist in many types of solids tumors, including melanoma, brain tumors, lung cancers, etc. (65, 66). TANs can be divided into N1 TANs and N2 TANs based on their cytokine status and anti- or protumor functions (67). Several studies have found that a high neutrophil/lymphocyte ratio in solid tumors correlates with poor patient outcomes (68). This is consistent with the reduced percentages of TANs in the tumors treated with NBTXR3+PBT+αPD1. Our results demonstrate that the triple therapy can reshape the immune microenvironment by minimizing the protumor effect of TANs in both irradiated and unirradiated tumors.

Importantly, we observed that treatment with PBT+αPD1 and NBTXR3+PBT+αPD1 enriched certain TCR sequences. Though the antigen specificity of these TCR clonotypes remains unknown, we suspect that the enriched clones target tumor-specific antigens.

Perhaps most profoundly, mice treated with NBTXR3+PBT+αPD1 that successfully cleared their tumors proved immune to rechallenge by the same tumors. We have previously seen this effect in mice treated with NBTXR3-mediated IRT (5). Survivor mice eliminated repeat tumor exposure, with 100% clearance in both cases. The rejection of tumor rechallenge in the survivor mice offers the tantalizing possibility that patients treated with NBTXR3+PBT+αPD1 may develop a long-term and potent antitumor memory immune response, which may effectively prevent tumor relapse.

### Conclusion.

In this study, we demonstrate for the first time to our knowledge that PBT-mediated IRT can potently activate the antitumor immune response, leading to improved control of irradiated and unirradiated tumors. Adding NBTXR3 significantly enhances the antitumor immune response, improves survival, and induces robust antitumor memory immunity. These results paved the way for the application of PBT in activating antitumor immune response and extending its role as a localized therapy to a systemic treatment option.

## Methods

### Materials.

Nanobiotix provided radiation-enhancing nanoparticles (NBTXR3). Bristol Myers Squibb provided αPD1. Antibodies for flow cytometry, including αCD45–Pacific Blue (catalog 103126), αCD4–APC/Fire 750 (catalog 100568), αCD8–PerCP-Cy5.5 (catalog 100734), αCD62L–PE-Cy7 (catalog 104418), and αCD44-APC (catalog 103012) were purchased from BioLegend. Bouin’s fixative solution for staining lung metastases was from Polysciences Inc. (catalog 16045-1).

### Cell line and culture methods.

The αPD1-resistant lung cancer cell line 344SQR, created in a previous study (69), was used for all experiments in this study. The culture conditions were the same as previously reported (4).

### Tumor inoculation and combination treatment.

Eight- to 12-week-old 129/SvEv syngeneic female mice from Taconic Biosciences were used in this study. The experimental mice were housed in specific pathogen–free facilities. They were fed Picolab Rodent Diet 5053 and had unlimited access to water. Light cycles were regulated using a timer set to 12-hour light/12-hour dark periods, and the ambient temperature was maintained within a range of 20.6°C–22.2°C. Primary and secondary tumors were established using previously described methods (4, 5). Briefly, 344SQR cells (5 × 10^4^ in 100 μL PBS) were subcutaneously injected into the right leg on day 0 and into the left leg on day 4 to create primary (to be irradiated by PBT) and secondary tumors (not to be irradiated by PBT), respectively. The tumors were monitored, and the tumor volumes were calculated as *V* = 0.5 × width^2^ × length. αPD1 (200 μg) was given to the mice via intraperitoneal injection on days 7, 10, 14, 21, 28, 35, and 42. NBTXR3 nanoparticles at 25% tumor volume in 5% glucose were intratumorally injected into the primary tumors on day 7. Primary tumors were irradiated with 2 fractions of 12 Gy (total dose 24 Gy) proton radiation on days 8 and 9 using a 200 MeV proton beam from a Hitachi PROBEAT (Hitachi America, Ltd.) at the MD Anderson Proton Therapy Center.

### Tumor processing.

Both primary and secondary tumors in mice treated as described above were harvested on day 18 for scRNA-seq and day 19 for NanoString analysis. The tumor tissues were processed according to methods reported in a previous study (4). The dissociated cells designated for scRNA-seq were stained with αCD45-FITC. Cells designated for NanoString analysis were frozen in TRIzol (catalog 15596026, Thermo Fisher Scientific) for RNA extraction.

### RNA extraction and NanoString analysis of immune-related genes.

Total RNA was extracted from the tumors and blood via the chloroform/phenol method. The RNA was analyzed with an nCounter PanCancer Immune Profiling Panel and an nCounter MAX Analysis System, as previously described (4, 5).

### scRNA-seq.

Primary and secondary tumors harvested from mice (*n* = 5) in control, PBT+αPD1, and NBTXR3+PBT+αPD1 groups on day 18 were processed as described above. The dissociated cells from each mouse in the same group were pooled and stained with αCD45-FITC, then washed with RPMI 1640 medium supplemented with 2% fetal bovine serum (FBS), followed by sorting with a BD FACSAria II cell sorter. After flow sorting, at least 1 × 10^5^ CD45^+^ cells with at least 85% viability were used for scRNA-seq.

Sample processing was performed following 10× Genomics’ 5′ scRNAseq (version 2) and TCR enrichment guidelines (CG000331_ChromiumNextGEMSingleCell5-v2_UserGuide_RevC). Quality control steps after cDNA amplification and library preparation steps were carried out by running a Qubit HS dsDNA Assay (Thermo Fisher Scientific) along with an Agilent HS DNA Bioanalyzer for concentration and quality assessments, respectively. Library sample concentrations were verified by qPCR using a KAPA Biosystems KAPA Library Quantification Kit (Sigma-Aldrich). Samples were normalized to 5 nM for pooling. The gene expression libraries and TCR libraries were pooled in a ratio of 5 volumes gene expression library to 1 volume TCR library. The pool was sequenced using an Illumina NovaSeq 6000 S2, 100-cycle flow cell. The run parameters used were 26 cycles for read 1, 90 cycles for read 2, 10 cycles for index 1, and 10 cycles for index 2, as stipulated in the protocol mentioned above.

The raw unique molecular identifier (UMI) count data were loaded into the R Seurat (version 4.1.0) package for downstream analysis (70). Cells with over 25% mitochondria-derived UMI counts and those with no *Ptprc* expression were filtered out. The count matrix was first log-normalized with the scale factor set to 10,000 total genes per cell. The 6 samples were then integrated into a new matrix using the IntegrateData function with the top 2,000 highly variable genes selected by the Seurat function FindVariableGenes with default parameters. Principle component analysis (PCA) was performed on the integrated data matrix. The top PCs were selected based on the Elbowplot, representing at least 80% of the total variances. The main cell clusters were identified with the FindClusters function offered by Seurat, with a resolution set to 0.5. They were then visualized with uniform manifold approximation and projection (UMAP) plots.

The cell clusters were identified using the ImmGenData reference data in SingleR package (71) and manual selection by experts. Since all samples were composed of CD45^+^ cells, 6 nonimmune cell types from the reference data set (endothelial cells, stem cells, stromal cells, epithelial cells, fibroblast, and microglia) were excluded. T cells were further separated into CD4^+^ and CD8^+^ T cells. For each cell type, the marker genes were identified by comparing the cluster with the other clusters using the FindConservedMarkers function. These marker genes were further used to confirm the cell types identified by SingleR. The Seurat Findallmarker function was performed to identify preferentially expressed genes in clusters and differentially expressed genes between different sample cells.

The bioinformatics analysis and data visualization of the single-cell TCR sequencing data was performed using the Immunarch package in R (72). The diversity of the repertories was measured using the inverse Simpson index, 
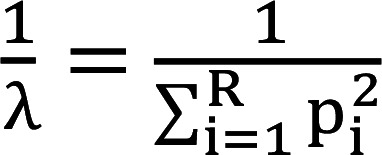
.

### Tumor rechallenge.

Survivor mice from NBTXR3+PBT+αPD1 were subcutaneously injected with 5 × 10^4^ 344SQR cells in the right flank on day 76 following tumor inoculation. Five untreated mice of similar age were also injected with the same number of 344SQR cells and served as the control. No additional treatments were given to the mice. All the mice were sacrificed 27 days after tumor rechallenge, and lungs and blood samples were harvested to count the number of lung metastases and to profile memory T cells.

### Profiling memory immune cell populations via flow cytometry.

Circulating immune cells were collected 3 days before and 19 days after tumor rechallenge and were stained with αCD45–Pacific Blue, αCD4–APC/Fire 750, αCD8–PerCP-Cy5.5, αCD62L–PE-Cy7, and αCD44-APC. Samples were analyzed with a Gallios Flow Cytometer (Beckman Coulter), and the resulting flow data were analyzed with Kaluza software version 2.1.

### Counting the number of lung metastases.

The initial studies harvested lung tissues on day 19 following tumor implantation. For the tumor rechallenge study, lung tissues from both the control group and the NBTXR3+PBT+αPD1 group were harvested 27 days after tumor reinjection. Lung tissues were treated with Bouin’s fixative solution for 3 days before the lung tumor nodules were counted.

### Statistics.

All statistical analyses were performed with Prism 9 (GraphPad Software). Tumor volumes are expressed as mean tumor volume ± standard error of the mean (SEM) and were compared by 2-way analysis of variance (ANOVA). Mouse survival rates were analyzed using the Kaplan-Meier method and were compared using log-rank tests. The number of lung metastases was analyzed utilizing 2-tailed *t* tests. The remaining data were analyzed using either ordinary 1-way ANOVA or the Kruskal-Wallis test. The data are expressed as mean ± SEM. *P* values of less than 0.05 were considered statistically significant.

### Study approval.

All animal procedures were approved by the Institutional Animal Care and Use Committee at MD Anderson Cancer Center.

### Data availability.

The data and materials supporting the findings of this study can be obtained from the corresponding author upon reasonable request. The scRNA-seq data have been deposited in figshare (https://doi.org/10.6084/m9.figshare.22755167.v1).

## Author contributions

YH, SP, N Sahoo, MAC, and JWW designed the study. YH, N Sahoo, Qianxia Wang, AH, HBB, DJD, DPP, EH, N Selene, CSKL, and TAV performed the experiments. YH, Qi Wang, JW, GB, and SP analyzed the data. YH, GB, and SP wrote the manuscript. JDS, FM, S Ganjoo, CL, JTP, NPO, S Gandhi, and QNN discussed the results, contributed to data interpretation, and reviewed the manuscript. All authors discussed the results and reviewed the manuscript.

## Supplementary Material

Supplemental data

## Version 1 06/22/2023

Electronic publication
