# Supplementary material for: Nanoparticle-enhanced proton beam immunoradiotherapy drives immune activation and durable tumor rejection
Source: JCI Insight. 2023 Jun 22;8(12):e167749. doi: 10.1172/jci.insight.167749 (PMC10371249; doi:10.1172/jci.insight.167749)
Supplement: Supplemental data [file jciinsight-8-167749-s105.pdf]

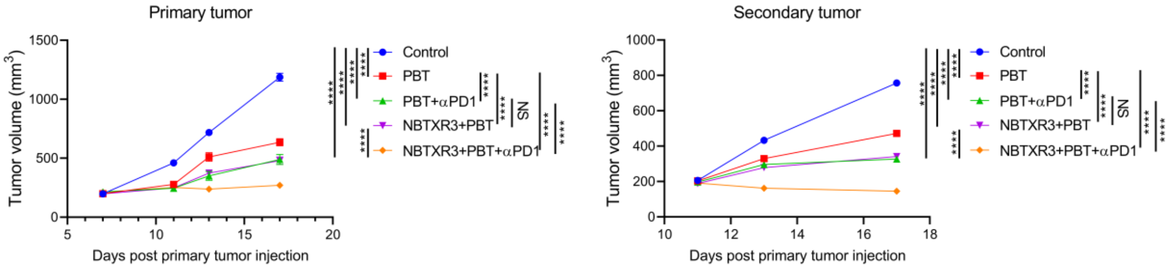

Supplemental Figure 1. Average growth of primary and secondary tumors (n=5) treated with various combinations of NBTXR3, PBT, and αPD1. Female 129/SvEv syngeneic mice aged 8-12 weeks were inoculated with 344SQR cells on the right and left legs to establish primary tumors on day 0 and secondary tumors on day 4, respectively. On day 7, primary tumors received intratumoral injections of NBTXR3, followed by two 12Gy proton beam radiation fractions on days 8 and 9. Mice were administered 200 µg of αPD1 via intraperitoneal injections on days 7, 10, and 14. Data are presented as mean ± standard error of the mean (SEM). A p-value < 0.05 was considered statistically significant. ****P < 0.0001, NS not significant.

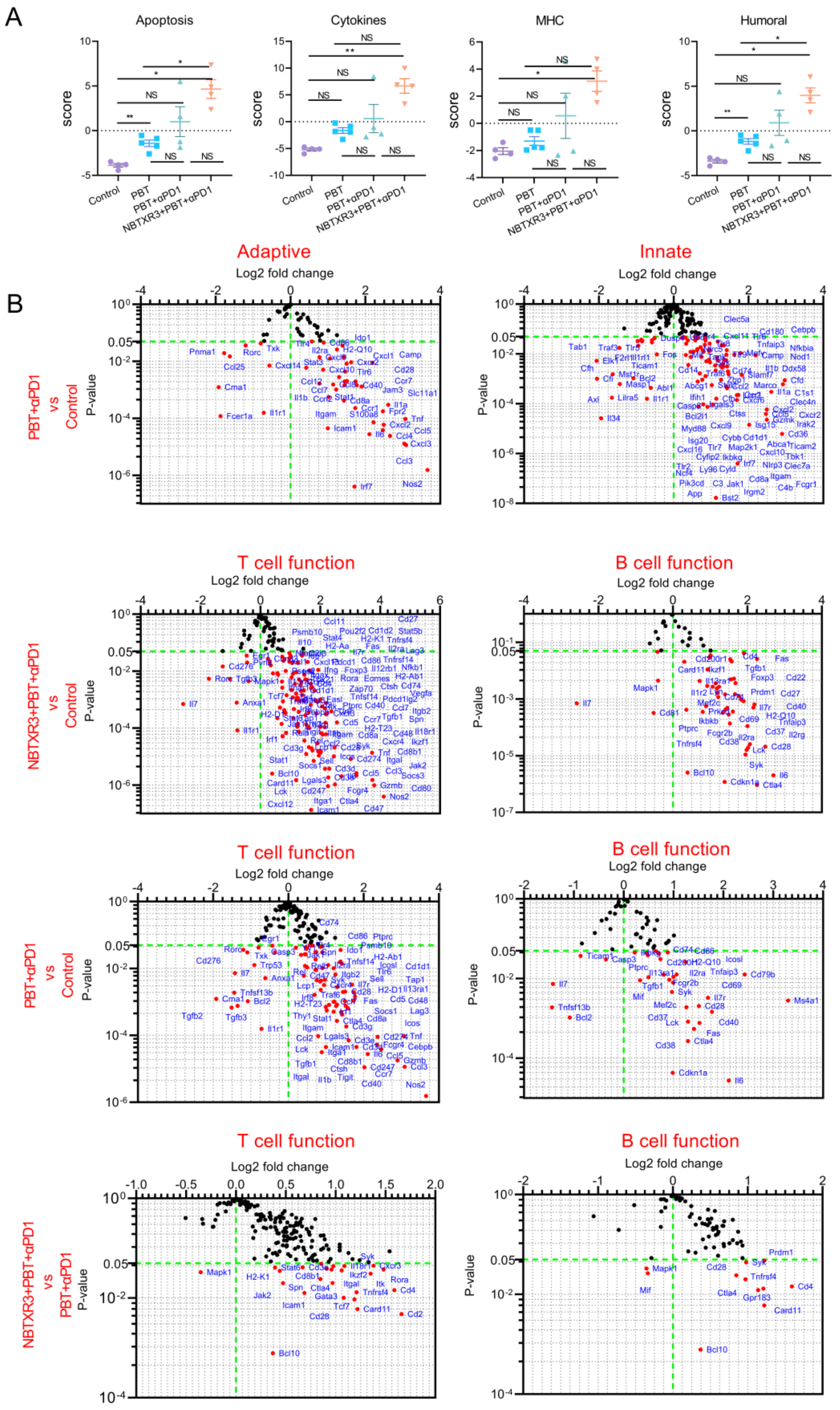
A
Apoptosis
Cytokines
MHC
Humoral
score
Control
PBT
PBT+αPD1
NBTXR3+PBT+αPD1
NS
B
Adaptive
Innate
Log2 fold change
P-value
PBT+αPD1 vs Control
T cell function
B cell function
NBTXR3+PBT+αPD1 vs Control
NBTXR3+PBT+αPD1 vs PBT+αPD1

Supplemental Figure 2. Triple therapy of NBTXR3, PBT, and αPD1 modulates the expression of immune-related genes in favor of antitumor immune response in the irradiated (Primary) tumors. (A) Activity scores of different immune pathways. **(B)** Changes in gene expression in adaptive, innate pathways, T cell function, and B cell function. Primary tumors were harvested from mice (n=4-5) treated with different combinations of NBTXR3, PBT, and αPD1 10 days post irradiation. Total RNAs were extracted from the tumors, followed by an analysis of immune-related genes with a nCounter PanCancer Immune Profiling Panel. The gene expression data were then analyzed with the PanCancer Immune Profiling Advanced Analysis Module. Data are expressed as means ± standard error of the mean (SEM). $P < 0.05$ was considered statistically significant. *$P < 0.05$, **$P < 0.01$, ***$P < 0.001$, NS not significant.

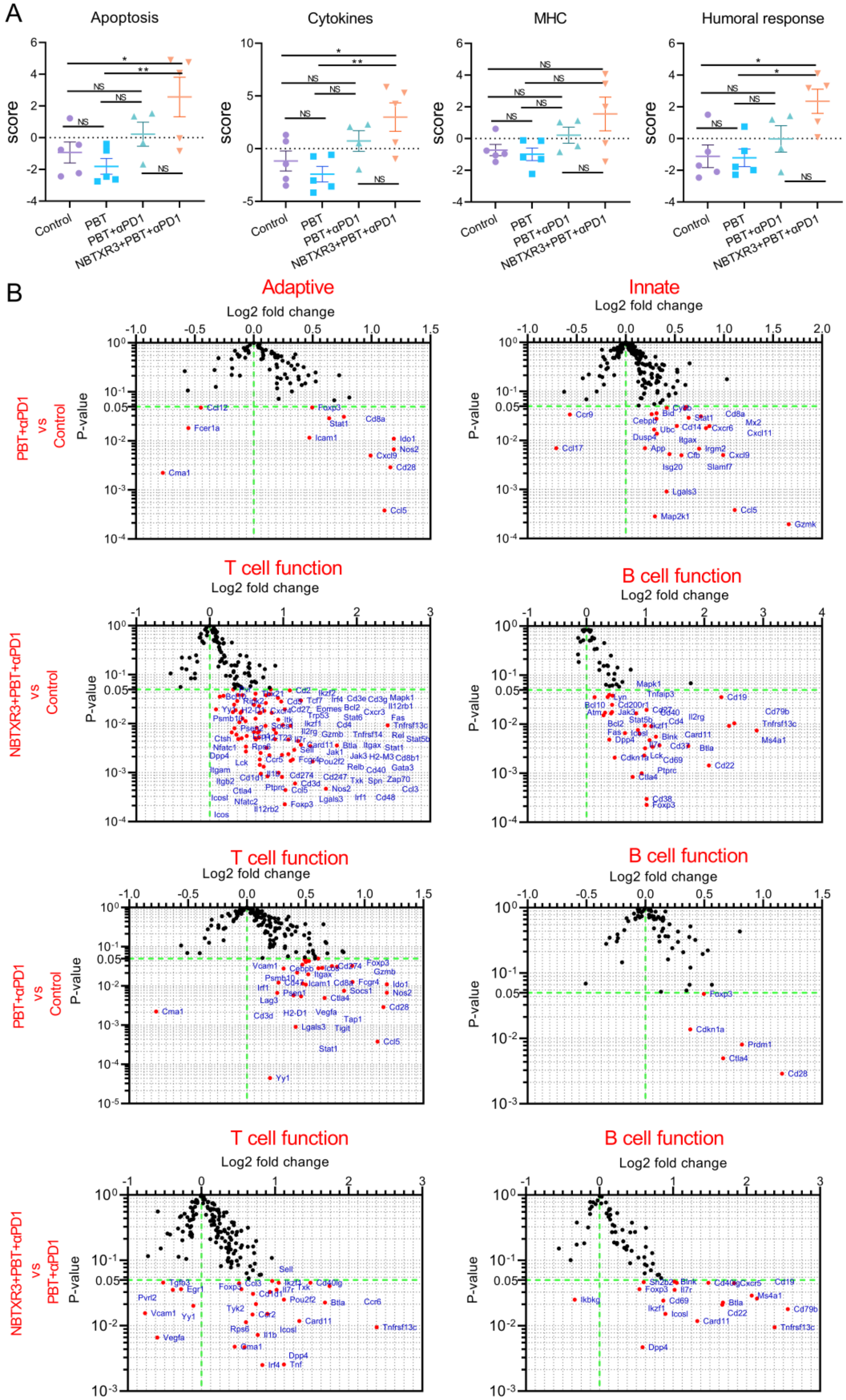
A
Apoptosis
Cytokines
MHC
Humoral response
score
Control
PBT
PBT+αPD1
NBTXR3+PBT+αPD1
NS
B
Adaptive
Innate
T cell function
B cell function
Log2 fold change
P-value
PBT+αPD1 vs Control
NBTXR3+PBT+αPD1 vs Control
PBT+αPD1 vs Control
NBTXR3+PBT+αPD1 vs PBT+αPD1

Supplemental Figure 3. Triple therapy of NBTXR3, PBT, and αPD1 modulates the expression of immune-related genes in favor of antitumor immune response in the unirradiated (Secondary) tumors. (A) Activity scores of different immune pathways. **(B)** Changes in gene expression in adaptive, innate pathways, T cell function, and B cell function. Secondary tumors were harvested from mice (n=4-5) treated with different combinations of NBTXR3, PBT, and αPD1 10 days post irradiation. Total RNAs were extracted from the tumors, followed by an analysis of immune-related genes with a nCounter PanCancer Immune Profiling Panel. The gene expression data were then analyzed with the PanCancer Immune Profiling Advanced Analysis Module. Data are expressed as means ± standard error of the mean (SEM). P< 0.05 was considered statistically significant. *P< 0.05, **P< 0.01, NS not significant.

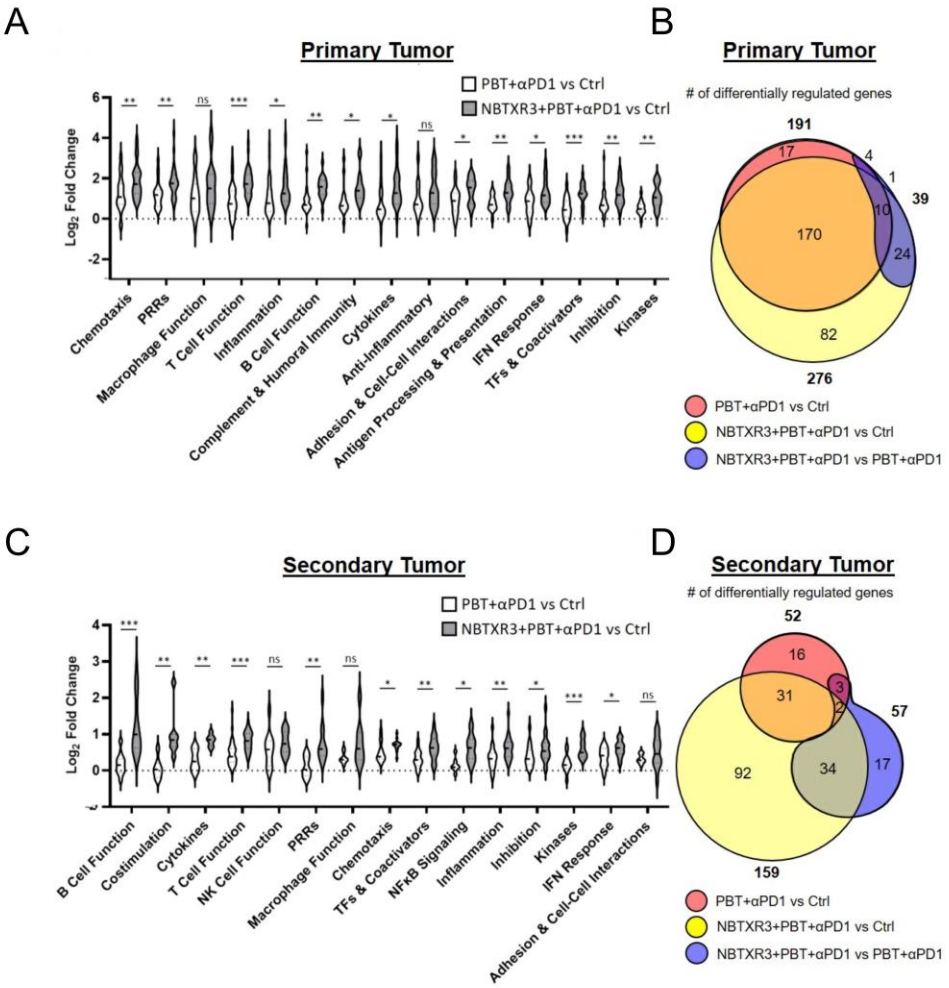

Supplemental Figure 4. Functional distribution of immuno-related genes differentially regulated between experimental groups. Gene transcripts identified as significantly upregulated in either the PBT+αPD1 or NBTXR3+PBT+αPD1 group relative to the control from the NanoString data (**Figure 2**, **Figure 3**, and **Supp. Figure 2** and **3**) were manually assigned to functional categories based on their known functions and associations. **(A)** Top 15 differentially expressed pathways in the primary tumors. **(B)** Genes differentially upregulated in the primary tumors in PBT+αPD1 relative to Control mice (red), NBTXR3+PBT+αPD1 relative to Control mice (green), and NBTXR3+PBT+αPD1 relative to PBT+αPD1 mice (blue). The area of each diagram is proportional to the number of genes contained therein, as is the area of overlap (i.e., number of genes shared) between them. **(C)** Top 15 differentially expressed pathways in the secondary tumors. **(D)** Genes differentially upregulated in the secondary tumors.

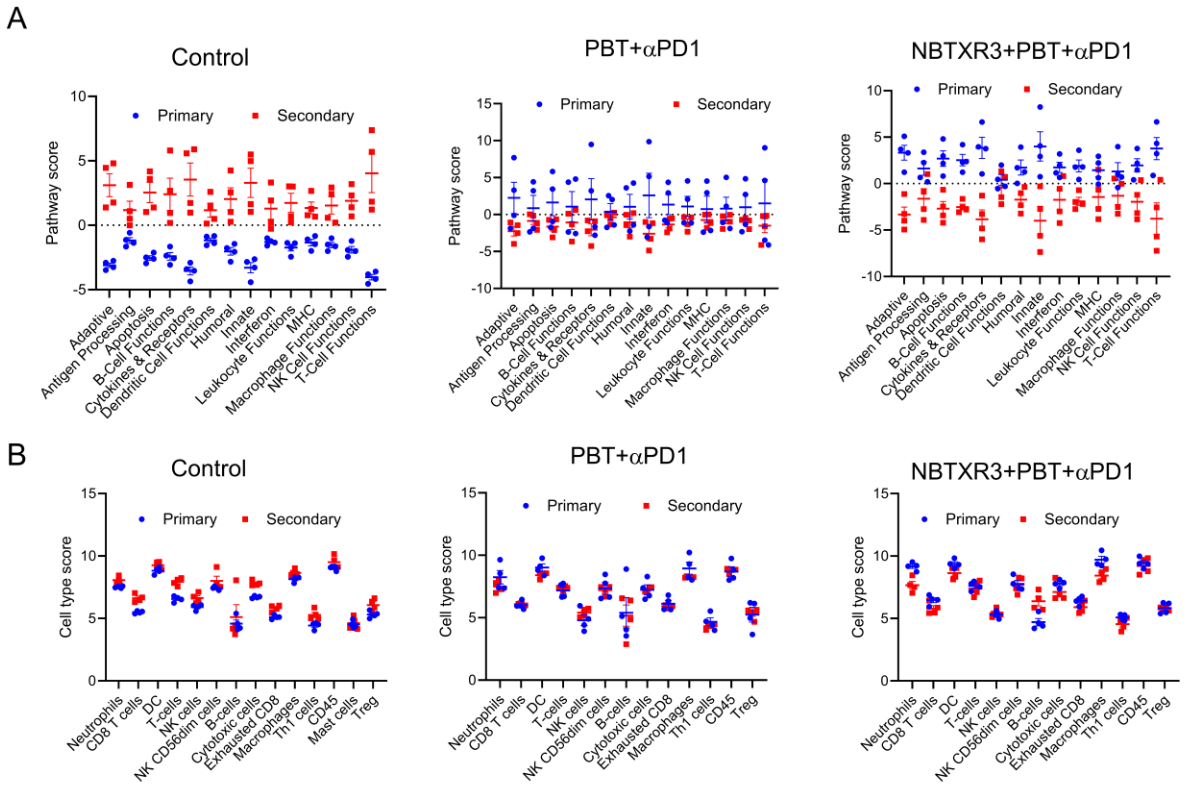

Supplemental Figure 5. (A) Comparison of immune pathway activities, and **(B)** relative cell abundance between the primary and secondary tumors.

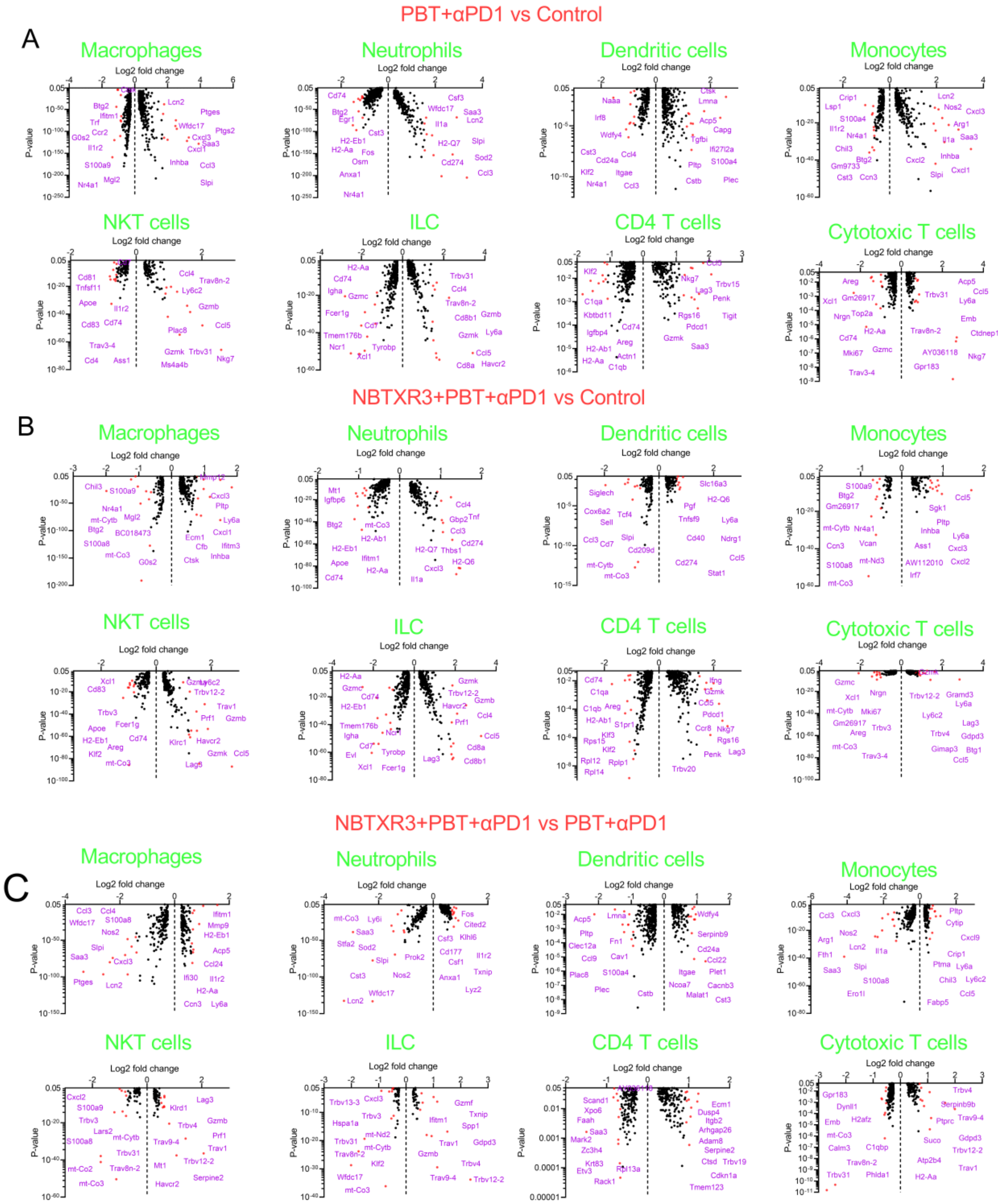

Supplemental Figure 6. Significantly changed gene expression in macrophages, neutrophils, DCs, monocytes, NKT cells, ILC, CD4^{+} T cells, and cytotoxic T cells in the primary tumors when treated with **(A)** Control vs PBT+αPD1, **(B)** Control vs NBTXR3+PBT+αPD1, and **(C)** PBT+αPD1 vs NBTXR3+PBT+αPD1.

A

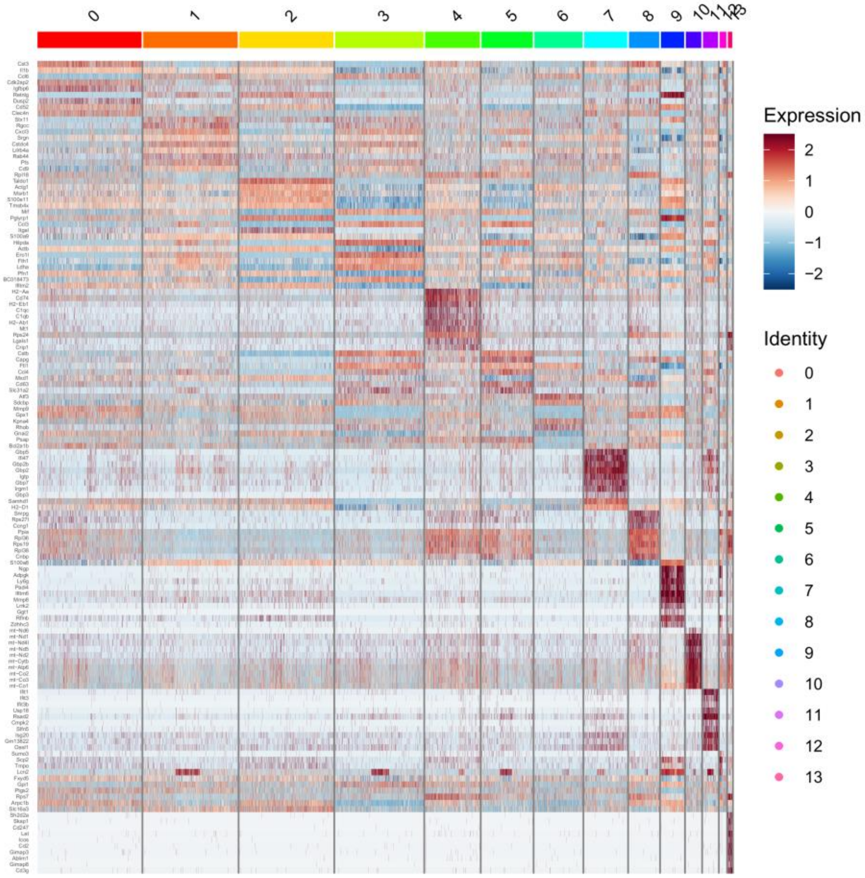
0
1
2
3
4
5
6
7
8
9
10
11
12
13
Expression
2
1
0
−1
−2
Identity
0
1
2
3
4
5
6
7
8
9
10
11
12
13

B

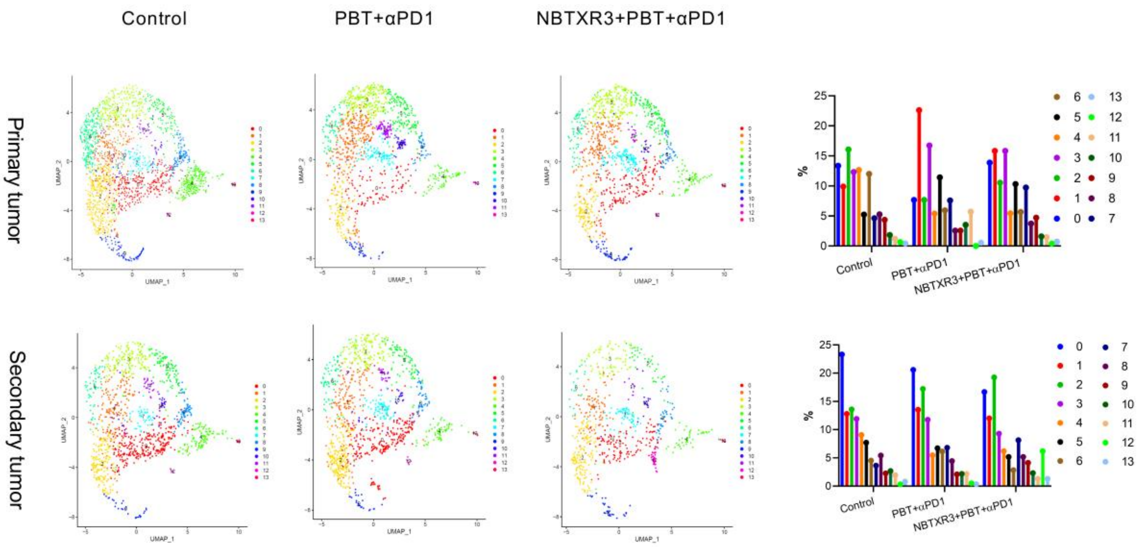
Control
PBT+αPD1
NBTXR3+PBT+αPD1
Primary tumor
Secondary tumor
UMAP_1
UMAP_2
%
25
20
15
10
5
0
Control
PBT+αPD1
NBTXR3+PBT+αPD1
6
5
4
3
2
1
0
13
12
11
10
9
8
7

Supplemental Figure 7. (A) Heatmap showing row-scaled expression of differently expressed genes per cluster for all neutrophils. **(B)** UMAP visualization and percentages of subclusters of neutrophils.

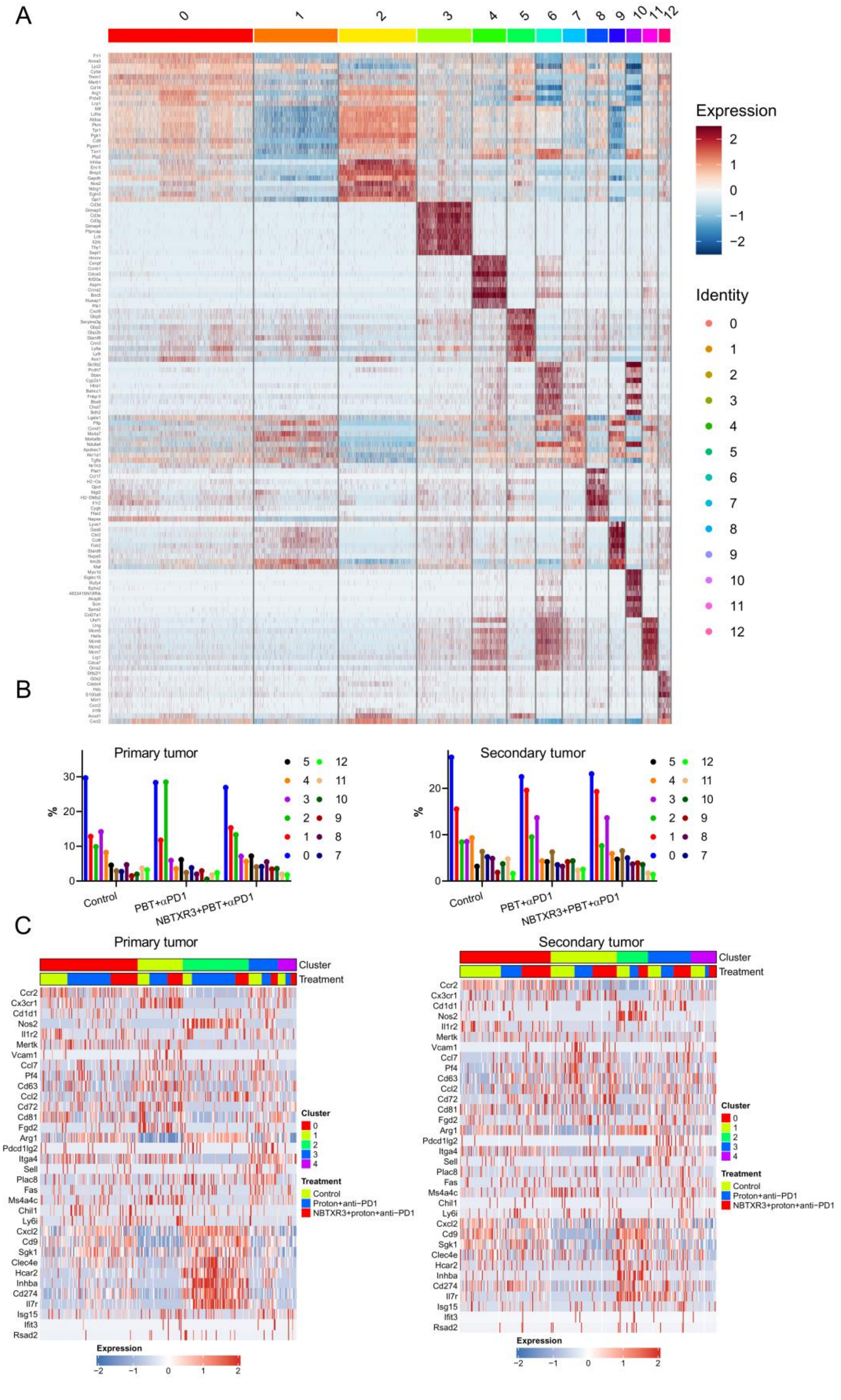
A
0 1 2 3 4 5 6 7 8 9 10 11 12
Expression
Identity
B
Primary tumor
Secondary tumor
%
Control
PBT+αPD1
NBTXR3+PBT+αPD1
C
Primary tumor
Secondary tumor
Cluster
Treatment
Ccr2
Cx3cr1
Cd1d1
Nos2
Il1r2
Mertk
Vcam1
Ccl7
Pf4
Cd63
Ccl2
Cd72
Cd81
Fgd2
Arg1
Pdcd1lg2
Itga4
Sell
Plac8
Fas
Ms4a4c
Chil1
Ly6i
Cxcl2
Cd9
Sgk1
Clec4e
Hcar2
Inhba
Cd274
Il7r
Isg15
Ifit3
Rsad2
Control
Proton+anti−PD1
NBTXR3+proton+anti−PD1
Expression

Supplemental Figure 8. (A) Heatmap showing row-scaled expression of differently expressed genes per cluster for all macrophages. **(B)** Percentages of subclusters of macrophages. **(C)** Gene set enrichment analysis performed on the top 5 macrophages in both primary and secondary tumors.

A

Control

Clonotype ID	CDR3s	Frequency	Proportion
1	TRB:CASSLDAGGGAETLYF<br>TRA:CAVSAGTGGADRLTF	161	11.45%
2	TRB:CASSSDWGDYAEQFF<br>TRA:CALWELKWNNNNRIFF<br>TRA:CAMRDYGGSGNKLIF	56	3.98%
3	TRB:CASSPDRGREQYF<br>TRA:CAVRDTNNNRIFF	46	3.27%
4	TRB:CASSLRGSQNTLYF	39	2.77%
5	TRB:CASSLGQSNTEVFF	29	2.06%
6	TRB:CTCSAGENTLYF<br>TRA:CAANNAGAKLTF	19	1.35%
7	TRB:CASRSDWGDYAEQFF<br>TRA:CAMREANYNVLYF	17	1.21%
8	TRB:CASVQGTEVFF<br>TRA:CAVTHNYAQGLTF	11	0.78%
9	TRB:CASSPRDTQYF<br>TRA:CAASMGNNNRIFF	11	0.78%
10	TRB:CASSPTGGAFAETLYF<br>TRA:CAASPSSGSWQLIF	9	0.64%

PBT+αPD1

Clonotype ID	CDR3s	Frequency	Proportion
1	TRB:CAWSLPGGEAEQFF<br>TRA:CAFSSNTDKVVF	335	23.14%
2	TRB:CASSLDWGDYAEQFF<br>TRA:CAMREHSGTYQRF	182	12.57%
3	TRB:CASSLDWGDYAEQFF<br>TRB:CASSRDWGGSSYEQYF<br>TRA:CAGDDYSNNRLTL	54	3.73%
4	TRB:CASRQGTEVFF<br>TRA:CAMVGNYAQGLTF	52	3.59%
5	TRB:CASSLDLGGIAETLYF<br>TRA:CAMRINAGAKLTF	43	2.97%
6	TRB:CASSLDTGGSSYEQYF<br>TRA:CAAAGTGGYKVVF	43	2.97%
7	TRB:CASSDDRGSPLYF<br>TRB:CASSDTISNERLFF<br>TRA:CAMSRGTGNYKYVF	36	2.49%
8	TRB:CASSTGHNNYAEQFF<br>TRA:CATVASSGSWQLIF	24	1.66%
9	TRB:CASSQDRGASEVFF<br>TRA:CARSGGNYKPTF	21	1.45%
10	TRB:CASRQGTEVFF<br>TRA:CAIVGNYAQGLTF	16	1.10%

NBTXR3+PBT+αPD1

Clonotype ID	CDR3s	Frequency	Proportion
1	TRB:CASSHRVNQDTQYF<br>TRB:CASSLLLNQDTQYF<br>TRB:CASSLDTYEQYF<br>TRB:CASSQNSDYTF<br>TRA:CAVSMNTGYQNFYF	319	19.04%
2	TRB:CASSRDRGREQYF<br>TRA:CAVRLNNNNRIFF	268	16.00%
3	TRB:CASSQDWGGTSYEQYF<br>TRA:CAADNYAQGLTF	82	4.90%
4	TRB:CASSPDWGGDTGQLYF<br>TRA:CATDRYSNNRLTL<br>TRA:CAVEDYSNNRLTL	73	4.36%
5	TRB:CASSQDRGREQYF<br>TRA:CAVRDSAQGLTF	47	2.81%
6	TRB:CASSYGTGGHSDYTF	47	2.81%
7	TRB:CASSLDTYEQYF<br>TRA:CAVENTGYQNFYF	44	2.63%
8	TRB:CASSLRVNQDTQYF<br>TRA:CAVSMNTGYQNFYF	37	2.21%
9	TRB:CASSDARDTLYF<br>TRA:CALREGNTGNYKYVF<br>TRA:CATGTGGADRLTF	26	1.55%
10	TRB:CASSPGFYEQYF<br>TRA:CAIDQNNYAQGLTF	22	1.31%

B

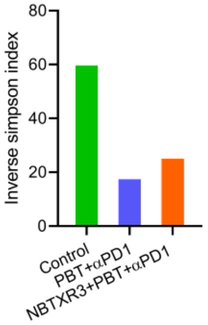

Supplemental Figure 9. (A) Top 10 TCRα and TCRβ pairs. (**B)** inverse Simpson index in T cell populations. Mice (n=5) inoculated with 344SQR tumors were treated with dual therapy of PBT+αPD1 or triple therapy of NBTXR3+PBT+αPD1 shown in **Figure 1a**, immune cells extracted from the irradiated tumors 17 days post radiation were analyzed via scRNAseq.

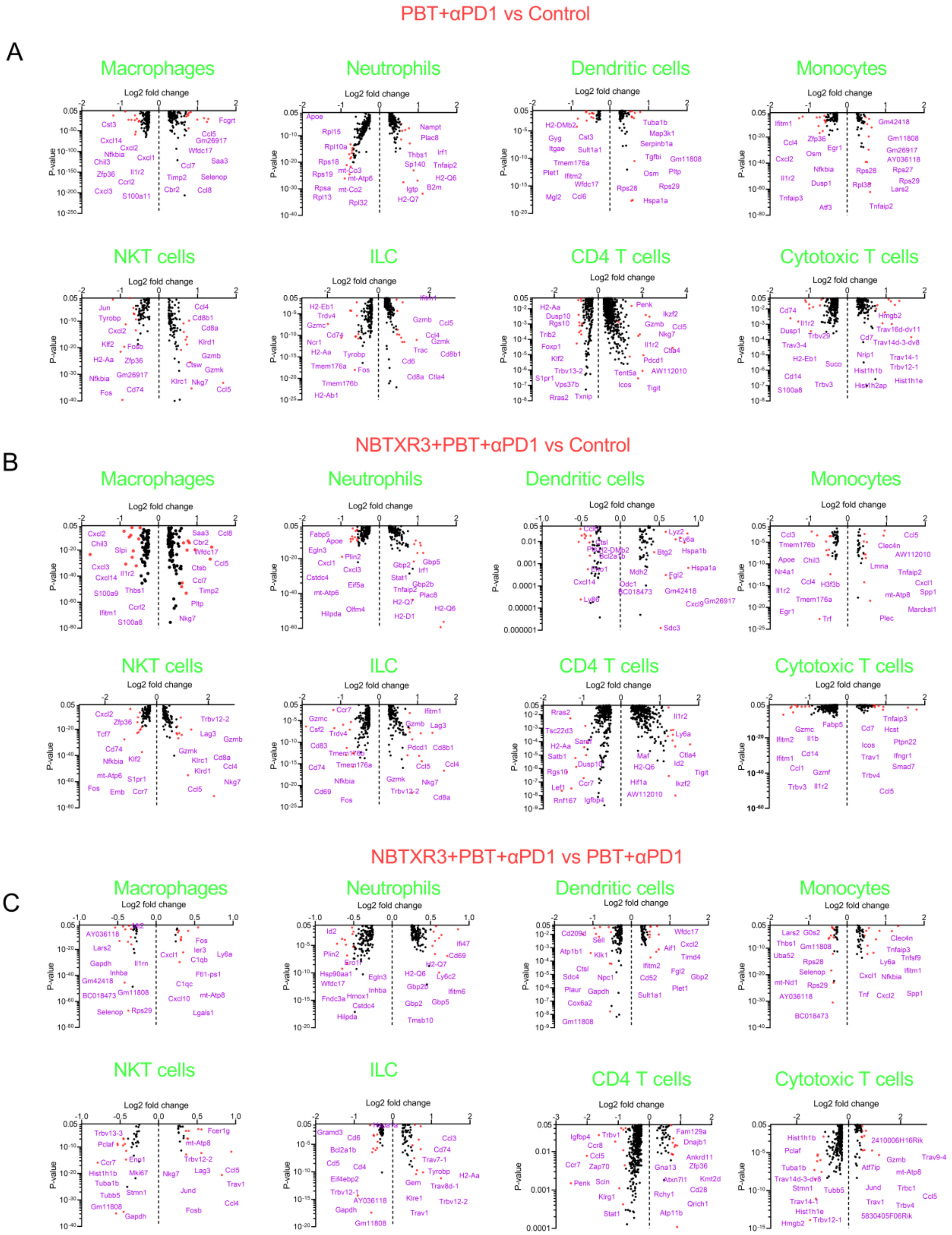
PBT+αPD1 vs Control
A
Macrophages
Neutrophils
Dendritic cells
Monocytes
NKT cells
ILC
CD4 T cells
Cytotoxic T cells
NBTXR3+PBT+αPD1 vs Control
B
Macrophages
Neutrophils
Dendritic cells
Monocytes
NKT cells
ILC
CD4 T cells
Cytotoxic T cells
NBTXR3+PBT+αPD1 vs PBT+αPD1
C
Macrophages
Neutrophils
Dendritic cells
Monocytes
NKT cells
ILC
CD4 T cells
Cytotoxic T cells
Log2 fold change
P-value

Supplemental Figure 10. Significantly changed gene expression in macrophages, neutrophils, DCs, monocytes, NKT cells, ILC, CD4^{+} T cells, and cytotoxic T cells in the secondary tumors when treated with **(A)** Control vs PBT+αPD1, **(B)** Control vs NBTXR3+PBT+αPD1, and **(C)** PBT+αPD1 vs NBTXR3+PBT+αPD1.

A

Control

Clonotype ID	CDR3s	Frequency	Proportion
1	TRB:CASSLDAGGGAETLYF<br>TRA:CAVSAGTGGADRLTF	67	5.20%
2	TRB:CASRSDWGDYAEQFF<br>TRA:CAMREANYNVLYF	51	3.96%
3	TRB:CASSSDWGDYAEQFF<br>TRA:CALWELKWNNNNRIFF<br>TRA:CAMRDYGGSGNKLIF	27	2.10%
4	TRB:CASSLRGSQNTLYF	15	1.16%
5	TRB:CASVQGTEVFF<br>TRA:CAVTHNYAQGLTF	11	0.85%
6	TRB:CASSPDRGREQYF<br>TRA:CAVRDTNNNRIFF	11	0.85%
7	TRB:CASSLGQSNTEVFF<br>TRA:CAVTWTQVVGQLTF	10	0.78%
8	TRB:CTCSTDSHYAEQFF<br>TRA:CAASANNRIFF	10	0.78%
9	TRB:CASSWADQISNERLFF<br>TRA:CAASEHGGSGNKLIF	9	0.70%
10	TRB:CASSPDRGFTEVFF<br>TRA:CAPLDRGSALGRLHF	8	0.62%

PBT+αPD1

Clonotype ID	CDR3s	Frequency	Proportion
1	TRB:CASSLDTGGSSYEQYF<br>TRA:CAAAGTGGYKVVF	76	4.10%
2	TRB:CASSLDLGGIAETLYF<br>TRA:CAMRINAGAKLTF	74	4.00%
3	TRB:CASSLDDWGNYAEQFF<br>TRA:CAAGKGGSAKLIF<br>TRA:CAASARQGGSAKLIF	66	3.56%
4	TRB:CASSSDWGDYAEQFF<br>TRA:CAMRLMDYANKMIF	47	2.54%
5	TRB:CASRQGTEVFF<br>TRA:CAMVGNYAQGLTF	45	2.43%
6	TRB:CASSIGTDLSNERLFF<br>TRA:CIVLLSGSFNKLTF	33	1.78%
7	TRB:CASSSTGGEQYF<br>TRA:CAMREGGGGSNAKLTF<br>TRA:CAVRPPTASLGKLQF	28	1.51%
8	TRB:CASSLGGDQNTLYF<br>TRA:CALVDAYKVIF	25	1.35%
9	TRB:CASSAGTGNERLFF<br>TRA:CAAEASNTDKVVF<br>TRA:CAVRDGGSGNKLIF<br>TRA:CAVSMGYQNFYF	25	1.35%
10	TRB:CASSDDRGSPLYF<br>TRB:CASSDTISNERLFF<br>TRA:CAMSRGTGNYKYVF	20	1.08%

NBTXR3+PBT+αPD1

Clonotype ID	CDR3s	Frequency	Proportion
1	TRB:CASSPDWGGDTGQLYF<br>TRA:CATDRYSNNRLTL<br>TRA:CAVEDYSNNRLTL	183	8.26%
2	TRB:CASSRDRGREQYF<br>TRA:CAVRLNNNNRIFF	170	7.67%
3	TRB:CASSHRVNQDTQYF<br>TRA:CAVSMNTGYQNFYF	155	7.00%
4	TRB:CASSQDWGGTSYEQYF<br>TRA:CAADNYAQGLTF	77	3.48%
5	TRB:CASSLLLNQDTQYF<br>TRB:CASSQNSDYTF<br>TRA:CAVSMNTGYQNFYF	60	2.71%
6	TRB:CASSLRVNQDTQYF<br>TRA:CAVSMNTGYQNFYF	59	2.66%
7	TRB:CASSYGTGGHSDYTF	53	2.39%
8	TRB:CASSRDRGREQYF<br>TRB:CASSSGQGEDTQYF<br>TRA:CAVRDSNNNRIFF	42	1.90%
9	TRB:CASSVTGGATNERLFF<br>TRA:CAASKTRIFF<br>TRA:CAVSANNYAQGLTF	33	1.49%
10	TRB:CSSSTGVTEVFF<br>TRA:CAMREGGYGGSGNKLIF	29	1.31%

B

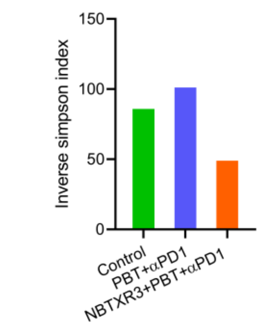

Supplemental Figure 11. (A) Top 10 TCRα and TCRβ pairs. (**B)** Inverse Simpson index in T cell populations. Mice (n=5) inoculated with 344SQR tumors were treated with dual therapy of PBT+αPD1 or triple therapy of NBTXR3+PBT+αPD1 shown in Figure 1A, immune cells extracted from the unirradiated tumors 9 days post radiation were analyzed via scRNAseq.

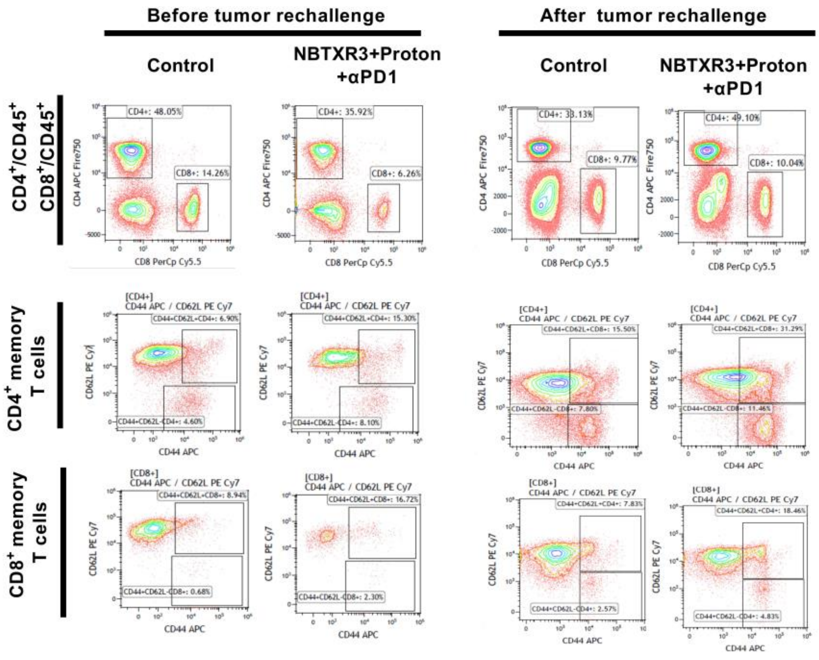

Supplemental Figure 12. Representative FACS graphs of memory T cells before and after tumor rechallenge.

Supplemental Tables

Supplemental Table 1: Genes Differentially Upregulated in Primary (Irradiated) Tumor

PBT+αPD1 vs Ctrl				
**Gene**	**Log2 Fold Change**	**Full Name**	**Notable Aliases**	**Function**
**Adhesion & Cell-Cell Interactions**				
Cd37	1.26	Cluster of differentiation 37	Tetraspanin-26	Cell surface glycoprotein known to complex with integrins and other transmembrane 4 superfamily proteins; may play a role in T cell-B cell interactions
Cd47	0.412	Cluster of differentiation 47	Integrin-associated protein (IAP)	Partners with membrane integrins to serve as an inhibitor of phagocytosis
Cyfip2	1.26	Cytoplasmic FMR1-interacting protein 2		Involved in T cell adhesion and p53/TP53-dependent induction of apoptosis
Icam1	0.997	Intracellular adhesion molecule 1	CD54	Cell surface glycoprotein that serves as strong adhesive ligand for LFA-1; important for leukocyte mobility and costimulation

Itga1	0.886	Integrin subunit alpha 1	CD49a; very late activation protein 1 (VLA-1)	Alpha 1 subunit for common integrin receptors; pairs with the β1 subunit to form a cell-surface receptor for collagen and laminin; involved in cell-cell adhesion and may play a role in inflammation and fibrosis
Itgal	1.2	Integrin alpha L		Pairs with ITGB2 to form lymphocyte function-associated antigen-1 (LFA-1), a common leukocyte adhesion molecule and costimulatory receptor
Itgam	1.45	Integrin alpha M	CD11b	Pairs with CD18 to form Mac-1 aka complement receptor 3; mediates leukocyte activation, adhesion, chemotaxis, migration, phagocytosis, and cell-mediated cytotoxicity; serves as a macrophage marker
Itgb2	1.25	Integrin subunit beta 2		Pairs with ITGAL to form a receptor for ICAM1, with ITGAM or ITGAX for iC3b and fibronectin
Jam3	1.9	Junctional adhesion molecule C		Immunoglobulin that mediates tight junctions between endothelial cells; mediates transepithelial migration of PMNs
Lgals3	0.794	Galectin 3		Galactose-specific lectin that binds IgE; involved in acute inflammatory responses, including neutrophil activation and adhesion, chemoattraction of monocytes macrophages,

				opsonization of apoptotic neutrophils, and activation of mast cells
Map2k1	0.393	Dual specificity mitogen-activated protein kinase kinase 1	MAPK/ERK kinase 1 (MEK1)	Essential component of the MAP kinase signal transduction pathway; participates in numerous biological functions, including cell growth, adhesion, survival, differentiation, transcription, metabolism, and cytoskeletal remodeling
S100a8	2.22	S100 calcium-binding protein A8	Calgranulin A	Calcium- and zinc-binding protein involved in pro-inflammatory, antimicrobial, oxidant-scavenging and apoptosis-inducing activities; can induce neutrophil chemotaxis, adhesion, phagocytosis, and degranulation; predominantly found as calprotectin (S100A8/A9) which has a wide plethora of intra- and extracellular functions, including adhesion, apoptosis, autophagy, cytoskeletal remodeling, cytokine production, chemotaxis, migration, inflammation, arachidonic acid metabolism, oxidant-scavenging, and PRR signaling
Sell	1.08	L-selectin		Mediates cell adhesion by binding to glycoproteins on neighboring cells

Thy1	0.719	Thy-1 T cell antigen		Cell surface glycoprotein involved in cell adhesion and communication in immune and nerve cells
**Antigen Processing & Presentation**				
Cd1d1	0.845	Cluster of differentiation 1 D1		Murine non-classical class I MHC; primarily presents lipid and glycolipid Ags
Cd74	0.643	Cluster of differentiation 74	MHC class II gamma chain	Stabilizes peptide-free class II αβ heterodimers during MHC-Ag complex formation
Ctss	1.43	Cathepsin S		Lysosomal protease that participates in processing of Ag by MHC class II
Cyfip2	1.26	Cytoplasmic FMR1-interacting protein 2		Involved in T cell adhesion and p53/TP53-dependent induction of apoptosis
H2-Ab1	0.902	Histocompatibility 2, class II antigen A, beta 1		MHC class II molecule; presents Ags to $CD4^+$ T cells
H2-D1	0.437	Histocompatibility 2, D region locus 1		MHC class I molecule; presents Ags to $CD8^+$ T cells
H2-Q10	1.24	Histocompatibility 2, Q region locus 10		MHC class I molecule; presents Ags to $CD8^+$ T cells

H2-T23	0.993	Histocompatibility 2, Q region locus 10		MHC class I molecule; presents Ags to CD8$^+$ T cells
Psmb10	0.461	Proteasome subunit beta type 10		20S core β subunit of the proteasome involved in Ag processing to generate class I binding peptides
Tap1	0.749	Transporter antigen peptide 1	Really interesting new gene 4 (RING4)	ATP-binding cassette transporter that pumps degraded cytosolic peptides from the cytosol to the ER for packaging into MHC class I molecules
**Anti-Inflammatory**				
Cd200r1	0.709	Cluster of differentiation 200 receptor 1		Inhibitory receptor for the CD200/OX2 cell surface glycoprotein; limits inflammation by inhibiting the expression of proinflammatory molecules including TNFα, IFNs, and iNOS
Cd274	2.37	Cluster of differentiation 274	Programmed cell death receptor ligand 1 (PD-L1)	Ubiquitously expressed ligand for co-inhibitory receptor PD-1; upregulated by tumors as an immune evasion strategy
Ctla4	1.29	Cytotoxic T lymphocyte antigen 4	CD152	Inhibitory receptor that blocks CD28 costimulation by competitively binding its ligands CD80 and CD86
Lag3	1.73	Lymphocyte activating gene 3	CD223	Inhibitory receptor on activated T cells; binds to ligands, such as FGL1; constitutively

				expressed on a subset of regulatory Tregs and contributes to their suppressive function; acts as a negative regulator of plasmacytoid DC activation
Mefv	1.79	Mediterranean fever	Marenostrin; pyrin	Involved in the regulation of innate immunity and the inflammatory response in response to IFNγ; both stimulates and restrains the inflammasome; also acts as a mediator of pyroptosis
Tnfaip3	0.804	Tumor necrosis factor, alpha-induced protein 3		Ubiquitin-editing enzyme that complexes with ITCH to degrade inflammatory signaling components in the TNF, IL1, and TLR pathways; targets TRAF2, TRAF6, and IKK
**Apoptosis**				
Casp8	0.314	Caspase 8		Cysteine-aspartic acid protease; cleaves and activates effector caspases CASP3, -4, -6, -7, -9, and -10; initiates pyroptosis through cleavage of gasdermin-D; mediates noncanonical cleavage of IL-1β in DCs and macrophages
Cyfip2	1.26	Cytoplasmic FMR1-interacting protein 2		Involved in T cell adhesion and p53/TP53-dependent induction of apoptosis

Fas	1.52	Fragment apoptosis stimulating		Cell surface death receptor; interaction with FAS-ligand triggers an apoptotic signaling cascade; also activates NFκB, ERK1, and MAPK8
S100a8	2.22	S100 calcium-binding protein A8	Calgranulin A	Calcium- and zinc-binding protein involved in pro-inflammatory, antimicrobial, oxidant-scavenging and apoptosis-inducing activities; can induce neutrophil chemotaxis, adhesion, phagocytosis, and degranulation; predominantly found as calprotectin (S100A8/A9) which has a wide plethora of intra- and extracellular functions, including adhesion, apoptosis, autophagy, cytoskeletal remodeling, cytokine production, chemotaxis, migration, inflammation, arachidonic acid metabolism, oxidant-scavenging, and PRR signaling
Tnfsf14	1.39	TNF superfamily member 14	CD258; LIGHT; Herpesvirus entry mediator ligand (HVEML)	Cytokine that binds to TNFRSF3/LTBR and TNFRSF14/HVEM; delivers costimulation to T cells; triggers apoptosis of various tumor cells
**Autophagy**				

Irgm2	0.453	Immunity-related GTPase family M member 2	Interferon-inducible protein 1 (IFI1)	Function not fully known, but most likely regulates autophagy and pro-inflammatory cytokine production
S100a8	2.22	S100 calcium-binding protein A8	Calgranulin A	Calcium- and zinc-binding protein involved in pro-inflammatory, antimicrobial, oxidant-scavenging and apoptosis-inducing activities; can induce neutrophil chemotaxis, adhesion, phagocytosis, and degranulation; predominantly found as calprotectin (S100A8/A9) which has a wide plethora of intra- and extracellular functions, including adhesion, apoptosis, autophagy, cytoskeletal remodeling, cytokine production, chemotaxis, migration, inflammation, arachidonic acid metabolism, oxidant-scavenging, and PRR signaling
Ubc	0.263	Polyubiquitin C		Serves various roles, including innate immunity, DNA repair, and stimulation of autophagy and the proteasomal response
**B Cell-associated Genes**				
Cd37	1.26	Cluster of differentiation 37	Tetraspanin-26	Cell surface glycoprotein known to complex with integrins and other transmembrane 4

				superfamily proteins; may play a role in T cell-B cell interactions
Cd48	1.5	Cluster of differentiation 48	B-lymphocyte activation marker (BLAST-1); signaling lymphocytic activation molecule 2 (SLAMF2)	B cell-specific cellular differentiation Ag; when bound to CD2, promotes T cell activation, and the formation of lipid rafts and caveolae for macrophages
Cd69	0.994	Cluster of differentiation 69	C-type lectin domain family 2, member C	Signal transmitting receptor in lymphocytes, NK cells, and platelets; induced upon T cell activation; involved in lymphocyte proliferation
Cd79b	2.43	Cluster of differentiation 79b	B29	One of the two flanking proteins that initiate signaling downstream of the BCR
Icosl	0.484	Inducible T cell costimulator ligand	CD275	Ligand for T cell-specific co-receptor ICOS; also induces B cell proliferation and plasma cell differentiation
Fcgr1	1.16	Fc fragment of IgG receptor Ia	CD64	High affinity receptor for the Fc region of γ-Igs; functions in both innate and adaptive immune responses
Fcgr2b	0.915	Fc fragment of IgG receptor IIb	CD32	Low affinity receptor for the Fc region of complexed or aggregated γ-Igs; involved in a variety of effector and regulatory functions

				such as phagocytosis of immune complexes and modulation of Ab production by B cells; essential for the maintenance of humoral tolerance; acts as a late checkpoint at the level of class-switched memory B cells, plasmablasts or plasma cells; regulates plasma-cell homeostasis and survival
Mef2c	0.998	Myocyte enhancer factor 2c		Transcriptional activator that binds specifically to the MEF2 element present in the regulatory regions of many muscle-specific genes; controls cardiac morphogenesis and myogenesis, and is also involved in vascular development; required for B cell survival and proliferation in response to BCR stimulation, efficient IgG1 Ab responses to T cell-dependent Ags, and for normal induction of GC B cells
Ms4a1	3.3	Membrane spanning 4-domains A1	CD20; Bp35	B cell-specific membrane protein that functions as a store-operated Ca(2+) channel component, promoting Ca(2+) influx after BCR activation
Pik3cd	0.584	Phosphatidylinositol-4,5-bisphosphate 3-		A subunit of PI3K; acts downstream of TLR4, TCR, BCR, and CD40; contributes to T helper cell expansion, mast cell development, and

		kinase catalytic subunit delta isoform		neutrophil chemotaxis, extravasation, and respiratory burst
Spn	0.72	Sialophorin	Leukosialin; CD43	Cell surface sialoglycoprotein expressed by T cells, B cells, monocytes, and granulocytes; promotes lymph node localization in T cells; shunts T cells away from the T_H2 phenotype and towards T_H1; promotes the expression of IFNγ in $CD4^+$ T cells
Syk	0.969	Spleen-associated tyrosine kinase		Critical kinase that transmits signals from the TCR and BCR
**Cell Cycle**				
Cdkn1a	0.986	Cyclin dependent kinase inhibitor 1A	p21; CDK-interaction protein 1 (CIP1)	Binds to and inhibits cyclin-dependent kinase activity, preventing phosphorylation of critical cyclin-dependent kinase substrates and blocking cell cycle progression
**Chemotaxis**				
Ccl2	1.4	C-C motif chemokine ligand 2		Chemoattractant ligand for CCR2 and -4; attracts monocytes and basophils
Ccl3	3.09	C-C motif chemokine ligand 3	Macrophage inflammatory protein 1α (MIP1α)	Chemoattractant ligand for CCR1, -4, and -5

Ccl4	2.66	C-C motif chemokine ligand 4	Macrophage inflammatory protein 1β (MIP1β)	Chemoattractant for NK cells and monocytes; binds to CCR5 receptors
Ccl5	2.47	C-C motif chemokine ligand 5	Regulated upon activation, normally T-expressed, and presumably secreted (RANTES))	Chemoattractant ligand for CCR1, -3, -4, and -5; attracts blood monocytes, memory T helper cells and eosinophils; causes the release of histamine from basophils and activates eosinophils
Ccl7	1.22	C-C motif chemokine ligand 7	Monocte chemotactic protein 3 (MCP3)	General chemokine that recruits leukocytes to infected tissues; mainly observed in monocyte mobilization
Ccl8	1.14	C-C motif chemokine ligand 8	Monocyte chemoattractant protein 2 (MCP2)	General chemokine that recruits leukocytes to infected tissues
Ccl12	0.773	C-C motif chemokine ligand 12	Monocyte chemotactic protein 5 (MCP5)	Chemoattractant specific for eosinophils, monocytes, and lymphocytes; found primarily in the lymph nodes and thymus, but can be strongly expressed by macrophages
Ccr1	1.73	C-C motif chemokine receptor 1	MIP1α receptor	Receptor for CCL3, -5, -7, and -23

Ccr7	1.74	C-C chemokine receptor type 7	CD197	Chemokine receptor that activates B and T cells and promotes their homing to secondary lymphoid organs; also stimulates DC expression of MHC class I and II
Ccrl2	1.44	C-C chemokine receptor-like 2		Stabilizes TLR4 surface expression on macrophages
Cxcl1	1.5	C-X-C motif chemokine ligand 1	GRO1 oncogene	Chemoattractant ligand for CXCR2; plays a role in inflammation and as a chemoattractant for neutrophils
Cxcl2	2.49	C-X-C motif chemokine ligand 2	Macrophage inflammatory protein 2-alpha (MIP2α); GRO2 oncogene	Chemokine produced by activated monocytes and neutrophils and expressed at sites of inflammation
Cxcl3	3.04	C-X-C motif chemokine ligand 3	GRO3 oncogene	Ligand for CXCR2; attracts neutrophils
Cxcl9	0.774	C-X-C motif chemokine ligand 9	Humig	Chemoattractant ligand for CXCR3; attracts activated T cells
Cxcl10	0.874	C-X-C motif chemokine ligand 10	IFNγ-induced protein 10 (IP-10)	Macrophage, DC, T cell, and NK cell chemattractant secreted by several cell types in response to IFNγ; binds to CXCR3

Cxcl11	0.954	C-X-C motif chemokine ligand 11		Dominant ligand for CXCR3; attracts activated T cells; strongly induced by IFNγ
Cxcl16	0.961	C-X-C motif chemokine ligand 16		Chemoattractant for T cells and NKT cells produced by DCs in response to IFNγ and TNFα
Cxcr2	1.62	C-X-C motif chemokine receptor 2	CD182; IL-8 receptor B	Receptor for IL-8 and CXCL3; powerful chemoattractant for neutrophils
Cxcr4	0.947	C-X-C motif chemokine receptor 4	CD184; fusin	Alpha-chemokine receptor specific for SDF1 aka CXCL12
Cxcr6	1.69	C-X-C motif chemokine receptor 6	CD186	Receptor for the C-X-C chemokine CXCL16; expressed in several T lymphocyte subsets and bone marrow stromal cells
Isg15	2.02	Interferon-stimulated gene 15		Ubiquitin-like protein that binds intracellular target proteins upon activation by IFNα or β; can also be secreted to induce NK cell proliferation, act as a chemoattractant for neutrophils, and induce IFNγ upon binding to ITGAL/ITGB2
Itgam	1.45	Integrin alpha M	CD11b	Pairs with CD18 to form Mac-1 aka complement receptor 3; mediates leukocyte activation, adhesion, chemotaxis, migration,

				phagocytosis, and cell-mediated cytotoxicity; serves as a macrophage marker
S100a8	2.22	S100 calcium-binding protein A8	Calgranulin A	Calcium- and zinc-binding protein involved in pro-inflammatory, antimicrobial, oxidant-scavenging and apoptosis-inducing activities; can induce neutrophil chemotaxis, adhesion, phagocytosis, and degranulation; predominantly found as calprotectin (S100A8/A9) which has a wide plethora of intra- and extracellular functions, including adhesion, apoptosis, autophagy, cytoskeletal remodeling, cytokine production, chemotaxis, migration, inflammation, arachidonic acid metabolism, oxidant-scavenging, and PRR signaling
**Complement & Humoral Immunity**				
C1s1	0.85	Complement component 1s		Serine protease that enzymatically cleaves C4 and C2
C3	0.656	Complement component 3		Cleaved by C3 convertase to form C3a and C3b, an anaphalotoxin and an opsonizing agent, respectively
C4b	0.624	Complement component 4B		Mediates interactions between Ab-bound Ags and other complement components

Cfb	1.14	Complement factor B		Alternate complement pathway component; when cleaved, produces a serine protease that binds to C3b to form C3 convertase
Cfd	2.97	Complement factor D	Adipsin	Chymotrypsin-family peptidase that cleaves factor B when the latter is complexed with factor C3b, activating C3 convertase
Fcgr1	1.16	Fc fragment of IgG receptor Ia	CD64	High affinity receptor for the Fc region of γ-Igs; functions in both innate and adaptive immune responses
Fcgr2b	0.915	Fc fragment of IgG receptor IIb	CD32	Low affinity receptor for the Fc region of complexed or aggregated γ-Igs; involved in a variety of effector and regulatory functions such as phagocytosis of immune complexes and modulation of Ab production by B cells; essential for the maintenance of humoral tolerance; acts as a late checkpoint at the level of class-switched memory B cells, plasmablasts or plasma cells; regulates plasma-cell homeostasis and survival
Fcgr4	2.36	Fragment crystallizable gamma receptor 4	Fc receptor-like 3 (Fcrl3); CD16-2	Putative mouse ortholog to human FcγRIIIA
**Costimulation**				

Cd28	1.51	Cluster of differentiation 28		Essential T cell co-receptor that enhances T cell activation, proliferation, cytokine production, and survival; binds to CD80 and CD86
Cd40	1.77	Cluster of differentiation 40		APC-expressed costimulatory protein that binds to CD40L on $CD4^{+}$ T cells, causing activation of both
Cd86	0.886	Cluster of differentiation 86	B7-2	One of the two ligands for the CD28 costimulatory receptor and the CTLA4 inhibitory receptor, the other being CD80
Icam1	0.997	Intracellular adhesion molecule 1	CD54	Cell surface glycoprotein that serves as strong adhesive ligand for LFA-1; important for leukocyte mobility and costimulation
Icos	1.58	Inducible T cell costimulator	CD278	Enhances all basic T cell responses to foreign Ag; essential both for efficient interaction between T and B cells and for normal Ab responses to T cell-dependent Ags
Icosl	0.484	Inducible T cell costimulator ligand	CD275	Ligand for T cell-specific co-receptor ICOS; also induces B cell proliferation and plasma cell differentiation

Ptprc	0.739	Protein tyrosine phosphatase receptor type C	CD45; leukocyte common antigen (LCA)	Delivers costimulation during T cell activation upon binding to its ligand DPP4; dephosphorylates Lyn and suppresses JAK kinases
Tnfsf14	1.39	TNF superfamily member 14	CD258; LIGHT; Herpesvirus entry mediator ligand (HVEML)	Cytokine that binds to TNFRSF3/LTBR and TNFRSF14/HVEM; delivers costimulation to T cells; triggers apoptosis of various tumor cells
**Cytokines**				
Il1a	2.61	Interleukin 1 alpha	Hematopoietin-1	Cytokine produced by monocytes and macrophages in response to cell injury; stimulates thymocyte proliferation by inducing IL-2 release; also stimulates B cell maturation and proliferation, and fibroblast growth factor activity
Il1b	1.3	Interleukin 1 beta	Catabolin	One of the two primary inflammatory cytokines produced by the inflammasome (the other one being IL-18); induces neutrophil influx and activation, T cell activation and cytokine production, B cell activation and Ab production, fibroblast proliferation, and collagen production; synergizes with IL-12 to induce IFNγ synthesis from T_H1 cells

Il2ra	1.06	Interleukin 2 receptor subunit alpha	CD25	Alpha chain of the IL-2 receptor
Il6	2.11	Interleukin 6		Pro-inflammatory cytokine that signals through the JAK and STAT pathways
Il7r	1.69	Interleukin 7 receptor	CD127	Receptor for IL-7
Mif	0.328	Macrophage migration inhibitory factor	L-dopachrome tautomerase	Pro-inflammatory cytokine that promotes macrophage function through suppression of anti-inflammatory effects of glucocorticoids
Tgfb1	0.498	Transforming growth factor beta 1		Multifunctional protein that regulates the growth and differentiation of various cell types and is involved in various processes, such as normal development, immune function, microglia function and responses to neurodegeneration; can induce EMT and cell migration in various cell types; frequently acts as an immunosuppressive cytokine in the TME
Tnf	3.07	Tumor necrosis factor	Cachectin	Inflammatory cytokine mainly produced by macrophages; binds to TNFRSF1A/TNFR1 and TNFRSF1B/TNFBR; capable of inducing cell death in certain tumor cell lines

Tnfsf14	1.39	TNF superfamily member 14	CD258; LIGHT; Herpesvirus entry mediator ligand (HVEML)	Cytokine that binds to TNFRSF3/LTBR and TNFRSF14/HVEM; delivers costimulation to T cells; triggers apoptosis of various tumor cells
**Cytotoxicity**				
Gzmb	2.9	Granzyme B	Fragmentin 2	Abundant protease in the cytosolic granules of cytotoxic T and NK cells that activates caspase-mediated cell death when delivered into the target cell through the immunological synapse
Gzmk	2.49	Granzyme K	Tryptase II	Granule-secreted, pro-apoptotic serine protease found in the cytoplasmic granules of CTLs
Fcgr4	2.36	Fragment crystallizable gamma receptor 4	Fc receptor-like 3 (Fcrl3); CD16-2	Putative mouse ortholog to human FcγRIIIA
Itgam	1.45	Integrin alpha M	CD11b	Pairs with CD18 to form Mac-1 aka complement receptor 3; mediates leukocyte activation, adhesion, chemotaxis, migration, phagocytosis, and cell-mediated cytotoxicity; serves as a macrophage marker

S100a8	2.22	S100 calcium-binding protein A8	Calgranulin A	Calcium- and zinc-binding protein involved in pro-inflammatory, antimicrobial, oxidant-scavenging and apoptosis-inducing activities; can induce neutrophil chemotaxis, adhesion, phagocytosis, and degranulation; predominantly found as calprotectin (S100A8/A9) which has a wide plethora of intra- and extracellular functions, including adhesion, apoptosis, autophagy, cytoskeletal remodeling, cytokine production, chemotaxis, migration, inflammation, arachidonic acid metabolism, oxidant-scavenging, and PRR signaling
**Dendritic Cell Function**				
Casp8	0.314	Caspase 8		Cysteine-aspartic acid protease; cleaves and activates effector caspases CASP3, -4, -6, -7, -9, and -10; initiates pyroptosis through cleavage of gasdermin-D; mediates noncanonical cleavage of IL-1β in DCs and macrophages
Tigit	0.861	T cell immunoreceptor with Ig and ITIM domains		Binds with high affinity to the poliovirus receptor, causing increased secretion of IL-10, decreased secretion of IL-12B, and

				suppressing T cell activation by promoting the generation of mature immunoregulatory DCs
**Growth/Proliferation**				
Lcn2	1.69	Lipocalin 2	Neutrophil gelatinase-associated lipocalin (NGAL)	Neutrophil-secreted factor that sequesters iron-containing siderophores; also functions as a growth factor
Map2k1	0.393	Dual specificity mitogen-activated protein kinase kinase 1	MAPK/ERK kinase 1 (MEK1)	Essential component of the MAP kinase signal transduction pathway; participates in numerous biological functions, including cell growth, adhesion, survival, differentiation, transcription, metabolism, and cytoskeletal remodeling
Tgfb1	0.498	Transforming growth factor beta 1		Multifunctional protein that regulates the growth and differentiation of various cell types and is involved in various processes, such as normal development, immune function, microglia function and responses to neurodegeneration; can induce EMT and cell migration in various cell types; frequently acts as an immunosuppressive cytokine in the TME
Yy1	0.196	Yin yang 1		Ubiquitous factor that serves as a transcriptional "switch", either promoting or

				repressing the transcription of numerous genes through the selective recruitment of either histone deacetylases or acetyltransferases; plays a fundamental role in diverse processes, such as differentiation, replication, and cellular proliferation
**Inflammation**				
Bst2	1.13	Bone marrow stromal cell antigen 2	Tethrin; CD317	IFN-induced antiviral factor that blocks budding of enveloped viruses by directly tethering nascent virions to the membranes of infected cells
Casp8	0.314	Caspase 8		Cysteine-aspartic acid protease; cleaves and activates effector caspases CASP3, -4, -6, -7, -9, and -10; initiates pyroptosis through cleavage of gasdermin-D; mediates noncanonical cleavage of IL-1β in DCs and macrophages
Cd38	1.41	Cluster of differentiation 38	ADP-ribosyl cyclase 1	Synthesizes the second messengers cyclic ADP-ribose and NADPH; appears to play a critical role in inflammation, although its exact immunological function(s) remain(s) poorly defined

Cebpb	0.724	CCAAT/enhancer-binding protein beta		Critical macrophage TF that promotes expression of several acute-phase and inflammatory cytokine genes, including Il6
Ctsh	0.906	Cathepsin H		Lysosomal protease; increased in macrophages in response to IFNγ
Il1b	1.3	Interleukin 1 beta	Catabolin	One of the two primary inflammatory cytokines produced by the inflammasome (the other one being IL-18); induces neutrophil influx and activation, T cell activation and cytokine production, B cell activation and Ab production, fibroblast proliferation, and collagen production; synergizes with IL-12 to induce IFNγ synthesis from T_H1 cells
Il6	2.11	Interleukin 6		Pro-inflammatory cytokine that signals through the JAK and STAT pathways
Irf1	1.2	Interferon regulatory factor 1		Transcriptional regulator that promotes inflammatory innate and adaptive immune responses
Irf7	1.71	Interferon regulatory factor 7		Key transcriptional regulator of type I IFN-dependent immune responses; promotes transcription of IFNα and -β

Irf8	0.841	Interferon regulatory factor 8		TF that regulates of lineage commitment in myeloid cell maturation; promotes monocyte and plasmacytoid DC development
Irgm2	0.453	Immunity-related GTPase family M member 2	Interferon-inducible protein 1 (IFI1)	Function not fully known, but most likely regulates autophagy and pro-inflammatory cytokine production
Isg15	2.02	Interferon-stimulated gene 15		Ubiquitin-like protein that binds intracellular target proteins upon activation by IFNα or β; can also be secreted to induce NK cell proliferation, act as a chemoattractant for neutrophils, and induce IFNγ upon binding to ITGAL/ITGB2
Isg20	0.904	Interferon-stimulated gene 20		IFN-induced antiviral exoribonuclease that acts on ssRNA with minor activity towards ssDNA
Jak1	0.337	Janus kinase 1		Essential tyrosine kinase involved signal transduction in type I and II cytokines and IFNs
Mif	0.328	Macrophage migration inhibitory factor	L-dopachrome tautomerase	Pro-inflammatory cytokine that promotes macrophage function through suppression of anti-inflammatory effects of glucocorticoids
Mefv	1.79	Mediterranean fever	Marenostrin; pyrin	Involved in the regulation of innate immunity and the inflammatory response in response to IFNγ; both stimulates and restrains the

				inflammasome; also acts as a mediator of pyroptosis
Nlrp3	1.47	NACHT domain-, leucine-rich repeat-, and PYD-containing protein 3	Cryopyrin	PRR with a wide diversity of recognized targets that activates the NLRP3 inflammasome consisting of NLRP3, PYCARD, and caspase-1/-8
Nos2	3.66	Inducible nitric oxide synthase (iNOS)		Produces reactive oxygen species and contributes to inflammatory cytokine production
S100a8	2.22	S100 calcium-binding protein A8	Calgranulin A	Calcium- and zinc-binding protein involved in pro-inflammatory, antimicrobial, oxidant-scavenging and apoptosis-inducing activities; can induce neutrophil chemotaxis, adhesion, phagocytosis, and degranulation; predominantly found as calprotectin (S100A8/A9) which has a wide plethora of intra- and extracellular functions, including adhesion, apoptosis, autophagy, cytoskeletal remodeling, cytokine production, chemotaxis, migration, inflammation, arachidonic acid metabolism, oxidant-scavenging, and PRR signaling
Spn	0.72	Sialophorin	Leukosialin; CD43	Cell surface sialoglycoprotein expressed by T cells, B cells, monocytes, and granulocytes;

				promotes lymph node localization in T cells; shunts T cells away from the T_H2 phenotype and towards T_H1; promotes the expression of IFNγ in $CD4^+$ T cells
Tbk1	0.767	TANK-binding kinase 1		Coordinates the activation of IRF3 and NFκB and induction of type I IFNs
Tnf	3.07	Tumor necrosis factor	Cachectin	Inflammatory cytokine mainly produced by macrophages; binds to TNFRSF1A/TNFR1 and TNFRSF1B/TNFBR; capable of inducing cell death in certain tumor cell lines
Traf6	0.697	Tumor necrosis factor receptor-associated factor 6		Adaptor protein that acts in the CD40 signaling cascade; promotes inflammation, IL-6, and TNFα
**Inhibition**				
Bcl2l1	0.665	B cell lymphoma 2 like 1	Protein phosphatase 1	Potent inhibitor of caspase-mediated cell death
Cd47	0.412	Cluster of differentiation 47	Integrin-associated protein (IAP)	Partners with membrane integrins to serve as an inhibitor of phagocytosis
Cd200r1	0.709	Cluster of differentiation 200 receptor 1		Inhibitory receptor for the CD200/OX2 cell surface glycoprotein; limits inflammation by inhibiting the expression of proinflammatory molecules including TNFα, IFNs, and iNOS

Cd274	2.37	Cluster of differentiation 274	Programmed cell death receptor ligand 1 (PD-L1)	Ubiquitously expressed ligand for co-inhibitory receptor PD-1; upregulated by tumors as an immune evasion strategy
Cdkn1a	0.986	Cyclin dependent kinase inhibitor 1A	p21; CDK-interaction protein 1 (CIP1)	Binds to and inhibits cyclin-dependent kinase activity, preventing phosphorylation of critical cyclin-dependent kinase substrates and blocking cell cycle progression
Ctla4	1.29	Cytotoxic T lymphocyte antigen 4	CD152	Inhibitory receptor that blocks CD28 costimulation by competitively binding its ligands CD80 and CD86
Cyld	0.381	Cylindromatosis lysine 63 deubiquitinase		Inhibits NFκB activation by deubiquitinating upstream signaling factors; inhibits Wnt signaling; restricts polyubiquitination of RIPK1 and -2, thereby limiting necroptosis
Ido1	1.39	Indoleamine 2,3-dioxygenase 1		Initiates catabolism of tryptophan; limits immunopathology by inhibiting T cell division
Lag3	1.73	Lymphocyte activating gene 3	CD223	Inhibitory receptor on activated T cells; binds to ligands, such as FGL1; constitutively expressed on a subset of regulatory Tregs and contributes to their suppressive function; acts as a negative regulator of plasmacytoid DC activation

Nfkbia	1.12	Nuclear factor kappa B inhibitor alpha		Inhibits activity of REL dimers by masking of their nuclear localization signals
Nlrc5	0.633	NLR family CARD domain containing 5	NOD27	Inhibits NFκB and type I IFN signaling pathways; may also regulate the type II IFN signaling pathway
Socs1	1.27	Suppressor of cytokine signaling 1		Inhibits JAK proteins; negative regulator of IL-6
Tank	0.743	TRAF family member-associated NFκB activator		Inhibitory protein that sequesters TRAFs in the cytoplasm, constitutively binds TBK1, and serves as a negative regulator of NFκB
Tgfb1	0.498	Transforming growth factor beta 1		Multifunctional protein that regulates the growth and differentiation of various cell types and is involved in various processes, such as normal development, immune function, microglia function and responses to neurodegeneration; can induce EMT and cell migration in various cell types; frequently acts as an immunosuppressive cytokine in the TME
Tigit	0.861	T cell immunoreceptor with Ig and ITIM domains		Binds with high affinity to the poliovirus receptor, causing increased secretion of IL-10, decreased secretion of IL-12B, and suppressing T cell activation by promoting the generation of mature immunoregulatory DCs

Interferon Response				
Bst2	1.13	Bone marrow stromal cell antigen 2	Tethrin; CD317	IFN-induced antiviral factor that blocks budding of enveloped viruses by directly tethering nascent virions to the membranes of infected cells
Ctsh	0.906	Cathepsin H		Lysosomal protease; increased in macrophages in response to IFNγ
Ifih1	1.05	Interferon induced with helicase C domain 1	Helicard; melanoma differentiation-associated protein 5 (MDA5)	PRR for cytoplasmic dsRNA; upon target recognition, associates with MAVS to activate TNK1 and IKBKE, which phosphorylate IRF3 and -7, which, in turn, activate transcription of IFNα and -β
Irf1	1.2	Interferon regulatory factor 1		Transcriptional regulator that promotes inflammatory innate and adaptive immune responses
Irf7	1.71	Interferon regulatory factor 7		Key transcriptional regulator of type I IFN-dependent immune responses; promotes transcription of IFNα and -β
Irf8	0.841	Interferon regulatory factor 8		TF that regulates of lineage commitment in myeloid cell maturation; promotes monocyte and plasmacytoid DC development

Isg15	2.02	Interferon-stimulated gene 15		Ubiquitin-like protein that binds intracellular target proteins upon activation by IFNα or β; can also be secreted to induce NK cell proliferation, act as a chemoattractant for neutrophils, and induce IFNγ upon binding to ITGAL/ITGB2
Isg20	0.904	Interferon-stimulated gene 20		IFN-induced antiviral exoribonuclease that acts on ssRNA with minor activity towards ssDNA
Jak1	0.337	Janus kinase 1		Essential tyrosine kinase involved signal transduction in type I and II cytokines and IFNs
Mefv	1.79	Mediterranean fever	Marenostrin; pyrin	Involved in the regulation of innate immunity and the inflammatory response in response to IFNγ; both stimulates and restrains the inflammasome; also acts as a mediator of pyroptosis
Tbk1	0.767	TANK-binding kinase 1		Coordinates the activation of IRF3 and NFκB and induction of type I IFNs
**Ion Transport**				
App	0.458	Amyloid-beta precursor protein		Cell surface receptor and transmembrane precursor protein that is cleaved by secretases to form a number of peptides; involved in cell

				mobility, copper homeostasis, and oxidative stress
Ms4a1	3.3	Membrane spanning 4-domains A1	CD20; Bp35	B cell-specific membrane protein that functions as a store-operated Ca(2+) channel component, promoting Ca(2+) influx after BCR activation
Slc11a1	1.46	Natural resistance-associated macrophage protein 1		Macrophage-specific metal ion transporter; uptakes divalent metal cations to neutralize ROSs
**IRAKs & TRAFs**				
Irak2	0.943	Interleukin-1 receptor-associated kinase 2		Adaptor protein involved in TLR and IL-1 signaling
Traf6	0.697	Tumor necrosis factor receptor-associated factor 6		Adaptor protein that acts in the CD40 signaling cascade; promotes inflammation, IL-6, and TNFα
**JAK-STAT Pathway**				
Jak1	0.337	Janus kinase 1		Essential tyrosine kinase involved signal transduction in type I and II cytokines and IFNs

Stat1	1.02	Signal transducer and activator of transcription 1		Transcriptional activator that mediates cellular responses to IFNs, cytokines, and other growth factors
Stat3	0.418	Signal transducer and activator of transcription 3		Transcriptional activator of genes involved in cell growth and apoptosis; activated by JAKs
**Kinases**				
Lck	1.29	Lymphocyte cell kinase		Src family tyrosine kinase that acts as one of the main signaling intermediaries downstream of the TCR; constitutively associated with the cytoplasmic portion of CD4
Jak1	0.337	Janus kinase 1		Essential tyrosine kinase involved signal transduction in type I and II cytokines and IFNs
Map2k1	0.393	Dual specificity mitogen-activated protein kinase kinase 1	MAPK/ERK kinase 1 (MEK1)	Essential component of the MAP kinase signal transduction pathway; participates in numerous biological functions, including cell growth, adhesion, survival, differentiation, transcription, metabolism, and cytoskeletal remodeling
Syk	0.969	Spleen-associated tyrosine kinase		Critical kinase that transmits signals from the TCR and BCR
**Lysosomal Activity**				

Camp	2.19	Cathelicidin antimicrobial peptide		Polypeptide stored in the lysosomes of macrophages and PMNs that digests phagocytosed cells
Ctsh	0.906	Cathepsin H		Lysosomal protease; increased in macrophages in response to IFNγ
Ctss	1.43	Cathepsin S		Lysosomal protease that participates in processing of Ag by MHC class II
**Macrophage Function**				
Camp	2.19	Cathelicidin antimicrobial peptide		Polypeptide stored in the lysosomes of macrophages and PMNs that digests phagocytosed cells
Casp8	0.314	Caspase 8		Cysteine-aspartic acid protease; cleaves and activates effector caspases CASP3, -4, -6, -7, -9, and -10; initiates pyroptosis through cleavage of gasdermin-D; mediates noncanonical cleavage of IL-1β in DCs and macrophages
Cd14	1.11	Cluster of differentiation 14		PRR that recognizes LPS; mostly found on macrophages
Cebpb	0.724	CCAAT/enhancer-binding protein beta		Critical macrophage TF that promotes expression of several acute-phase and inflammatory cytokine genes, including Il6

Clec5a	1.18	C-Type lectin domain family 5, member a	Myeloid DAP12-associating lectin-1	Critical macrophage receptor for dengue virus serotypes 1-4; positive regulator of osteoclastogenesis
Ctsh	0.906	Cathepsin H		Lysosomal protease; increased in macrophages in response to IFNγ
Itgam	1.45	Integrin alpha M	CD11b	Pairs with CD18 to form Mac-1 aka complement receptor 3; mediates leukocyte activation, adhesion, chemotaxis, migration, phagocytosis, and cell-mediated cytotoxicity; serves as a macrophage marker
Marco	2.92	Macrophage receptor with collagenous structure		A PRR that recognizes LDL
Mif	0.328	Macrophage migration inhibitory factor	L-dopachrome tautomerase	Pro-inflammatory cytokine that promotes macrophage function through suppression of anti-inflammatory effects of glucocorticoids
Slamf7	1.82	Signaling lymphocytic activation molecule family member 7		A super-activator of macrophages and a strong promoter of phagocytosis; binds to CD74
Slc11a1	1.46	Natural resistance-associated		Macrophage-specific metal ion transporter; uptakes divalent metal cations to neutralize ROSs

		macrophage protein 1		
Tnf	3.07	Tumor necrosis factor	Cachectin	Inflammatory cytokine mainly produced by macrophages; binds to TNFRSF1A/TNFR1 and TNFRSF1B/TNFBR; capable of inducing cell death in certain tumor cell lines
**Metabolism**				
Abca1	1.35	ATP-binding cassette transporter A1		Membrane-associated cholesterol efflux pump
Abcg1	1.4	ATP-binding cassette transporter G1		Membrane-associated cholesterol efflux pump
Cd36	2.9	Cluster of differentiation 36	Fatty acid translocase (FAT)	Class B scavenger receptor that mediates fatty acid uptake
Map2k1	0.393	Dual specificity mitogen-activated protein kinase kinase 1	MAPK/ERK kinase 1 (MEK1)	Essential component of the MAP kinase signal transduction pathway; participates in numerous biological functions, including cell growth, adhesion, survival, differentiation, transcription, metabolism, and cytoskeletal remodeling
**Migration/Motility**				

App	0.458	Amyloid-beta precursor protein		Cell surface receptor and transmembrane precursor protein that is cleaved by secretases to form a number of peptides; involved in cell mobility, copper homeostasis, and oxidative stress
Icam1	0.997	Intracellular adhesion molecule 1	CD54	Cell surface glycoprotein that serves as strong adhesive ligand for LFA-1; important for leukocyte mobility and costimulation
Itgam	1.45	Integrin alpha M	CD11b	Pairs with CD18 to form Mac-1 aka complement receptor 3; mediates leukocyte activation, adhesion, chemotaxis, migration, phagocytosis, and cell-mediated cytotoxicity; serves as a macrophage marker
Jam3	1.9	Junctional adhesion molecule C		Immunoglobulin that mediates tight junctions between endothelial cells; mediates transepithelial migration of PMNs
S100a8	2.22	S100 calcium-binding protein A8	Calgranulin A	Calcium- and zinc-binding protein involved in pro-inflammatory, antimicrobial, oxidant-scavenging and apoptosis-inducing activities; can induce neutrophil chemotaxis, adhesion, phagocytosis, and degranulation; predominantly found as calprotectin (S100A8/A9) which has a wide plethora of intra- and extracellular functions, including

				adhesion, apoptosis, autophagy, cytoskeletal remodeling, cytokine production, chemotaxis, migration, inflammation, arachidonic acid metabolism, oxidant-scavenging, and PRR signaling
Tgfb1	0.498	Transforming growth factor beta 1		Multifunctional protein that regulates the growth and differentiation of various cell types and is involved in various processes, such as normal development, immune function, microglia function and responses to neurodegeneration; can induce EMT and cell migration in various cell types; frequently acts as an immunosuppressive cytokine in the TME
**Neutrophil Function**				
Camp	2.19	Cathelicidin antimicrobial peptide		Polypeptide stored in the lysosomes of macrophages and PMNs that digests phagocytosed cells
Fpr2	2.47	Formyl peptide receptor 2	Lipoxin A4 receptor	Low affinity receptor for N-formyl-methionyl peptides; activates neutrophils
Lcn2	1.69	Lipocalin 2	Neutrophil gelatinase-associated lipocalin (NGAL)	Neutrophil-secreted factor that sequesters iron-containing siderophores; also functions as a growth factor

Ncf4	0.833	Neutrophil cytosolic factor 4	SH3 and PX domain-containing protein 4 (SH3PXD4)	Cytosolic regulatory component of the superoxide-producing phagocyte NADPH-oxidase, a multicomponent enzyme system important for host defense
Pik3cd	0.584	Phosphatidylinositol-4,5-bisphosphate 3-kinase catalytic subunit delta isoform		A subunit of PI3K; acts downstream of TLR4, TCR, BCR, and CD40; contributes to T helper cell expansion, mast cell development, and neutrophil chemotaxis, extravasation, and respiratory burst
S100a8	2.22	S100 calcium-binding protein A8	Calgranulin A	Calcium- and zinc-binding protein involved in pro-inflammatory, antimicrobial, oxidant-scavenging and apoptosis-inducing activities; can induce neutrophil chemotaxis, adhesion, phagocytosis, and degranulation; predominantly found as calprotectin (S100A8/A9) which has a wide plethora of intra- and extracellular functions, including adhesion, apoptosis, autophagy, cytoskeletal remodeling, cytokine production, chemotaxis, migration, inflammation, arachidonic acid metabolism, oxidant-scavenging, and PRR signaling
**NFκB Signaling**				

Ikbkg	0.223	Inhibitor of nuclear factor kappa B kinase subunit gamma	NFκB essential modifier (NEMO)	Regulatory subunit of the IKK core complex that phosphorylates inhibitors of NFκB thus leading to the dissociation of the inhibitor/NFκB complex and ultimately the degradation of the inhibitor
Rel	0.74	Avian reticuloendotheliosis viral oncogene homolog	c-Rel	One of the NFκB family TFs; important for B cell and Treg development
Relb	0.443	Avian reticuloendotheliosis viral oncogene homolog B		One of the NFκB family TFs; controls lymphoid development, DC biology, and noncanonical NFκB signaling
**NK Cell Function**				
Cd69	0.994	Cluster of differentiation 69	C-type lectin domain family 2, member C	Signal transmitting receptor in lymphocytes, NK cells, and platelets; induced upon T cell activation; involved in lymphocyte proliferation
Gzmb	2.9	Granzyme B	Fragmentin 2	Abundant protease in the cytosolic granules of cytotoxic T and NK cells that activates caspase-mediated cell death when delivered into the target cell through the immunological synapse

Gzmk	2.49	Granzyme K	Tryptase II	Granule-secreted, pro-apoptotic serine protease found in the cytoplasmic granules of CTLs
**Pattern Recognition Receptors**				
Ccrl2	1.44	C-C chemokine receptor-like 2		Stabilizes TLR4 surface expression on macrophages
Cd14	1.11	Cluster of differentiation 14		PRR that recognizes LPS; mostly found on macrophages
Cd180	1.28	Cluster of differentiation 180		Heterodimeric binding partner of Ly86 that participates in LPS binding in APCs
Clec4n	1.32	C-type lectin domain family 4, member N	Dectin-2	PRR specific for Mycobacterial mannose-capped lipoarabinomannan
Clec5a	1.18	C-Type lectin domain family 5, member a	Myeloid DAP12-associating lectin-1	Critical macrophage receptor for dengue virus serotypes 1-4; positive regulator of osteoclastogenesis
Clec7a	1.83	C-Type lectin domain family 7, member a	Dectin-1	PRR specific for β-1,3- and β-1,6-linked glucans from fungi and plants; necessary for the TLR2-mediated inflammatory response and for TLR2-mediated activation of NF-κB

Ddx58	0.528	DExD/H-box helicase 58	Retinoic acid-inducible gene I (RIG-I)	Cytoplasmic PRR that recognizes dsRNA; can promote T cell-independent B cell activation; uses MAVS as an adaptor
Fpr2	2.47	Formyl peptide receptor 2	Lipoxin A4 receptor	Low affinity receptor for N-formyl-methionyl peptides; activates neutrophils
Ifih1	1.05	Interferon induced with helicase C domain 1	Helicard; melanoma differentiation-associated protein 5 (MDA5)	PRR for cytoplasmic dsRNA; upon target recognition, associates with MAVS to activate TNK1 and IKBKE, which phosphorylate IRF3 and -7, which, in turn, activate transcription of IFNα and -β
Ly96	0.578	Lymphocyte antigen 96	Myeloid differentiation factor 2 (MD-2)	Heterodimeric binding partner of TLR4 that participates in LPS binding
Marco	2.92	Macrophage receptor with collagenous structure		A PRR that recognizes LDL
Myd88	0.64	Myeloid differentiation primary response 88		Key adaptor in the TLR signaling pathways; interacts with all TLRs except TLR3; activates NFκB and IRFs
Nlrp3	1.47	NACHT domain-, leucine-rich repeat-,	Cryopyrin	PRR with a wide diversity of recognized targets that activates the NLRP3

		and PYD-containing protein 3		inflammasome consisting of NLRP3, PYCARD, and caspase-1/-8
Nod1	0.947	Nucleotide binding oligomerization domain containing 1		Intracellular PRR that recognizes peptidoglycan-derived muropeptides and Shigella effector proteins
S100a8	2.22	S100 calcium-binding protein A8	Calgranulin A	Calcium- and zinc-binding protein involved in pro-inflammatory, antimicrobial, oxidant-scavenging and apoptosis-inducing activities; can induce neutrophil chemotaxis, adhesion, phagocytosis, and degranulation; predominantly found as calprotectin (S100A8/A9) which has a wide plethora of intra- and extracellular functions, including adhesion, apoptosis, autophagy, cytoskeletal remodeling, cytokine production, chemotaxis, migration, inflammation, arachidonic acid metabolism, oxidant-scavenging, and PRR signaling
Ticam2	1.18	TIR domain-containing adaptor molecule 2		Sorting adapter in various innate immune signaling cascades; bridges TLR2 and MyD88
Tlr2	0.853	Toll-like receptor 2	CD282	Surface PRR that binds to various lipid-containing PAMPs

Tlr4	0.571	Toll-like receptor 4	CD284	Surface PRR that recognizes LPS; pairs with LY96 and CD14; acts via MYD88, TIRAP, and TRAF6, leading to NFκB activation, cytokine secretion, and the inflammatory response; in complex with TLR6, promotes sterile inflammation in monocytes/macrophages in response to oxLDL or amyloid-β 42
Tlr6	1.42	Toll like receptor 6	CD286	Surface PRR that recognizes diacylated and triacylated lipopeptides
Tlr7	1.34	Toll-like receptor 7	CD287	Endosomic PRR that recognizes ssRNA
Tlr8	0.964	Toll-like receptor 8	CD288	Endosomic PRR that recognizes ssRNA
Tlr9	1.41	Toll like receptor 9	CD289	Endosomic PRR that recognizes unmethylated CpG dinucleotides
**Phagocytosis**				
Camp	2.19	Cathelicidin antimicrobial peptide		Polypeptide stored in the lysosomes of macrophages and PMNs that digests phagocytosed cells
Itgam	1.45	Integrin alpha M	CD11b	Pairs with CD18 to form Mac-1 aka complement receptor 3; mediates leukocyte activation, adhesion, chemotaxis, migration, phagocytosis, and cell-mediated cytotoxicity; serves as a macrophage marker

Ncf4	0.833	Neutrophil cytosolic factor 4	SH3 and PX domain-containing protein 4 (SH3PXD4)	Cytosolic regulatory component of the superoxide-producing phagocyte NADPH-oxidase, a multicomponent enzyme system important for host defense
S100a8	2.22	S100 calcium-binding protein A8	Calgranulin A	Calcium- and zinc-binding protein involved in pro-inflammatory, antimicrobial, oxidant-scavenging and apoptosis-inducing activities; can induce neutrophil chemotaxis, adhesion, phagocytosis, and degranulation; predominantly found as calprotectin (S100A8/A9) which has a wide plethora of intra- and extracellular functions, including adhesion, apoptosis, autophagy, cytoskeletal remodeling, cytokine production, chemotaxis, migration, inflammation, arachidonic acid metabolism, oxidant-scavenging, and PRR signaling
Slamf7	1.82	Signaling lymphocytic activation molecule family member 7		A super-activator of macrophages and a strong promoter of phagocytosis; binds to CD74
**ROS Generation & Protection**				
App	0.458	Amyloid-beta precursor protein		Cell surface receptor and transmembrane precursor protein that is cleaved by secretases to form a number of peptides; involved in cell

				mobility, copper homeostasis, and oxidative stress
Cybb	1.32	Cytochrome b-245 heavy chain	Nox2	Part of the NADPH oxidase process; generates superoxides
Ncf4	0.833	Neutrophil cytosolic factor 4	SH3 and PX domain-containing protein 4 (SH3PXD4)	Cytosolic regulatory component of the superoxide-producing phagocyte NADPH-oxidase, a multicomponent enzyme system important for host defense
Nos2	3.66	Inducible nitric oxide synthase (iNOS)		Produces reactive oxygen species and contributes to inflammatory cytokine production
**T Cell Function**				
Cd3d	1.81	Cluster of differentiation 3 delta		Component of the TCR-CD3 complex; upon phosphorylation by Lck, serves as a docking station for downstream TCR signaling adaptors
Cd3e	1.6	Cluster of differentiation 3 epsilon		Component of the TCR-CD3 complex; initiates the TCR-CD3 complex assembly by forming the two heterodimers CD3δ/CD3ε and CD3γ/CD3ε; also participates in internalization and cell surface down-regulation of TCR-CD3

				complexes via endocytosis sequences present in CD3ε cytosolic region
Cd3g	1.54	Cluster of differentiation 3 gamma		Component of the TCR-CD3 complex; plays an essential role in the dynamic regulation of TCR expression at the cell surface
Cd5	1.37	Cluster of differentiation 5	LEU1	Type-I transmembrane glycoprotein found on the surface of T and B cells; may act as a receptor in regulating T cell proliferation
Cd8a	1.43	Cluster of differentiation 8 alpha	LEU2	Alpha chain of the CD8 coreceptor, which binds to MHC class I; in T cells, functions primarily as a coreceptor for MHC class I molecule:peptide complex; in NK cells, the presence of CD8α homodimers at the cell surface provides a survival mechanism allowing conjugation and lysis of multiple target cells
Cd8b1	1.2	Cluster of differentiation 8 beta 1		Beta chain of the CD8 coreceptor, which binds to MHC class I
Cd28	1.51	Cluster of differentiation 28		Essential T cell co-receptor that enhances T cell activation, proliferation, cytokine production, and survival; binds to CD80 and CD86

Cd37	1.26	Cluster of differentiation 37	Tetraspanin-26	Cell surface glycoprotein known to complex with integrins and other transmembrane 4 superfamily proteins; may play a role in T cell-B cell interactions
Cd69	0.994	Cluster of differentiation 69	C-type lectin domain family 2, member C	Signal transmitting receptor in lymphocytes, NK cells, and platelets; induced upon T cell activation; involved in lymphocyte proliferation
Cd247	2.02	Cluster of differentiation 247	T cell surface glycoprotein CD3 zeta chain	Central intracellular signaling chain of the TCR, to which downstream signaling adaptors dock
Cyfip2	1.26	Cytoplasmic FMR1-interacting protein 2		Involved in T cell adhesion and p53/TP53-dependent induction of apoptosis
Gzmb	2.9	Granzyme B	Fragmentin 2	Abundant protease in the cytosolic granules of cytotoxic T and NK cells that activates caspase-mediated cell death when delivered into the target cell through the immunological synapse
Gzmk	2.49	Granzyme K	Tryptase II	Granule-secreted, pro-apoptotic serine protease found in the cytoplasmic granules of CTLs

Icos	1.58	Inducible T cell costimulator	CD278	Enhances all basic T cell responses to foreign Ag; essential both for efficient interaction between T and B cells and for normal Ab responses to T cell-dependent Ags
Lck	1.29	Lymphocyte cell kinase		Src family tyrosine kinase that acts as one of the main signaling intermediaries downstream of the TCR; constitutively associated with the cytoplasmic portion of CD4
Lcp1	0.867	Lymphocyte cytosolic protein 1	Plastin-2	Actin-binding protein that promotes T cell activation in response to costimulation through TCR/CD3 and CD2 or CD28; assists with IL2RA transport to the cell surface
Pik3cd	0.584	Phosphatidylinositol-4,5-bisphosphate 3-kinase catalytic subunit delta isoform		A subunit of PI3K; acts downstream of TLR4, TCR, BCR, and CD40; contributes to T helper cell expansion, mast cell development, and neutrophil chemotaxis, extravasation, and respiratory burst
Ptprc	0.739	Protein tyrosine phosphatase receptor type C	CD45; leukocyte common antigen (LCA)	Delivers costimulation during T cell activation upon binding to its ligand DPP4; dephosphorylates Lyn and suppresses JAK kinases
Spn	0.72	Sialophorin	Leukosialin; CD43	Cell surface sialoglycoprotein expressed by T cells, B cells, monocytes, and granulocytes;

				promotes lymph node localization in T cells; shunts T cells away from the T_H2 phenotype and towards T_H1; promotes the expression of IFNγ in $CD4^+$ T cells
Syk	0.969	Spleen-associated tyrosine kinase		Critical kinase that transmits signals from the TCR and BCR
**Transcription Factors & Coactivators**				
Cebpb	0.724	CCAAT/enhancer-binding protein beta		Critical macrophage TF that promotes expression of several acute-phase and inflammatory cytokine genes, including Il6
Irf1	1.2	Interferon regulatory factor 1		Transcriptional regulator that promotes inflammatory innate and adaptive immune responses
Irf7	1.71	Interferon regulatory factor 7		Key transcriptional regulator of type I IFN-dependent immune responses; promotes transcription of IFNα and -β
Irf8	0.841	Interferon regulatory factor 8		TF that regulates of lineage commitment in myeloid cell maturation; promotes monocyte and plasmacytoid DC development
Mef2c	0.998	Myocyte enhancer factor 2c		Transcriptional activator that binds specifically to the MEF2 element present in the regulatory regions of many muscle-specific genes;

				controls cardiac morphogenesis and myogenesis, and is also involved in vascular development; required for B cell survival and proliferation in response to BCR stimulation, efficient IgG1 Ab responses to T cell-dependent Ags, and for normal induction of GC B cells
Stat1	1.02	Signal transducer and activator of transcription 1		Transcriptional activator that mediates cellular responses to IFNs, cytokines, and other growth factors
Stat3	0.418	Signal transducer and activator of transcription 3		Transcriptional activator of genes involved in cell growth and apoptosis; activated by JAKs
Yy1	0.196	Yin yang 1		Ubiquitous factor that serves as a transcriptional "switch", either promoting or repressing the transcription of numerous genes through the selective recruitment of either histone deacetylases or acetyltransferases; plays a fundamental role in diverse processes, such as differentiation, replication, and cellular proliferation
Zbp1	1.27	Z-DNA binding protein 1	Tumor stroma and activated	Key innate sensor that recognizes and binds Z-RNA structures, which are produced by a number of viruses, and induces type-I IFN

			macrophage protein DLM-1	production; key activator of cellular necroptosis; promotes IL-1α induction in an NLRP3-inflammasome-independent manner
**Ubiquitin Regulation**				
Cyld	0.381	Cylindromatosis lysine 63 deubiquitinase		Inhibits NFκB activation by deubiquitinating upstream signaling factors; inhibits Wnt signaling; restricts polyubiquitination of RIPK1 and -2, thereby limiting necroptosis
Tnfaip3	0.804	Tumor necrosis factor, alpha-induced protein 3		Ubiquitin-editing enzyme that complexes with ITCH to degrade inflammatory signaling components in the TNF, IL1, and TLR pathways; targets TRAF2, TRAF6, and IKK
Ubc	0.263	Polyubiquitin C		Serves various roles, including innate immunity, DNA repair, and stimulation of autophagy and the proteasomal response

**NBTXR3+PBT+αPD1 vs Ctrl**				
**Gene**	**Log2 fold change**	**Full Name**	**Notable Aliases**	**Function**
**Acute Phase Response**				

App	0.801	Amyloid-beta precursor protein		Cell surface receptor and transmembrane precursor protein that is cleaved by secretases to form a number of peptides; involved in cell mobility, copper homeostasis, and oxidative stress
Psen2	0.848	Presenilin-2		Putative catalytic subunit of the gamma-secretase complex, which cleaves integral membrane proteins such as APP; also involved in Ca(2+) homeostasis between the ER and the mitochondria
Serping1	0.72	Serpin family G member 1	C1-inhibitor	Inflammation-induced acute phase protein that inhibits C1r and C1s proteases in the C1 complex
**Adhesion & Cell-Cell Interactions**				
Cd2	1.68	Cluster of differentiation 2	Leukocyte functional antigen 2 (LFA-2)	Interacts with LFA-3 and CD48 to mediate adhesion between T cells and other cell types
Cd37	2.07	Cluster of differentiation 37	Tetraspanin-26	Cell surface glycoprotein known to complex with integrins and other transmembrane 4 superfamily proteins; may play a role in T cell-B cell interactions

Cd47	0.743	Cluster of differentiation 47	Integrin-associated protein (IAP)	Partners with membrane integrins to serve as an inhibitor of phagocytosis
Cd97	0.748	Cluster of differentiation 97	BL-Ac[F2]	GPCR that promotes granulocyte adhesion and migration; activates T cells via binding to CD55; stimulates angiogenesis through binding integrin counterreceptors on endothelial cells
Cyfip2	1.74	Cytoplasmic FMR1-interacting protein 2		Involved in T cell adhesion and p53/TP53-dependent induction of apoptosis
Icam1	1.69	Intracellular adhesion molecule 1	CD54	Cell surface glycoprotein that serves as strong adhesive ligand for LFA-1; important for leukocyte mobility and costimulation
Ifitm1	0.626	Interferon-induced transmembrane protein 1	CD225	IFN-induced antiviral protein implicated in cell adhesion and control of cell growth and migration
Itga1	0.653	Integrin subunit alpha 1	CD49a; very late activation protein 1 (VLA-1)	Alpha 1 subunit for common integrin receptors; pairs with the β1 subunit to form a cell-surface receptor for collagen and laminin; involved in cell-cell adhesion and may play a role in inflammation and fibrosis
Itgal	2.16	Integrin alpha L		Pairs with ITGB2 to form lymphocyte function-associated antigen-1 (LFA-1), a common

				leukocyte adhesion molecule and costimulatory receptor
Itgam	2.19	Integrin alpha M	CD11b	Pairs with CD18 to form Mac-1 aka complement receptor 3; mediates leukocyte activation, adhesion, chemotaxis, migration, phagocytosis, and cell-mediated cytotoxicity; serves as a macrophage marker
Itgax	1.47	Integrin alpha X	CD11c	Adhesion molecule; signature marker of Ag-presenting DCs
Itgb2	1.87	Integrin subunit beta 2		Pairs with ITGAL to form a receptor for ICAM1, with ITGAM or ITGAX for iC3b and fibronectin
Jam3	1.54	Junctional adhesion molecule C		Immunoglobulin that mediates tight junctions between endothelial cells; mediates transepithelial migration of PMNs; promotes chemotaxis of vascular endothelial cells and stimulates angiogenesis
Lgals3	1.19	Galectin 3		Galactose-specific lectin that binds IgE; involved in acute inflammatory responses, including neutrophil activation and adhesion, chemoattraction of monocytes macrophages, opsonization of apoptotic neutrophils, and activation of mast cells

Map2k1	0.266	Dual specificity mitogen-activated protein kinase kinase 1	MAPK/ERK kinase 1 (MEK1)	Essential component of the MAP kinase signal transduction pathway; participates in numerous biological functions, including cell growth, adhesion, survival, differentiation, transcription, metabolism, and cytoskeletal remodeling
S100a8	2.3	S100 calcium-binding protein A8	Calgranulin A	Calcium- and zinc-binding protein involved in pro-inflammatory, antimicrobial, oxidant-scavenging and apoptosis-inducing activities; can induce neutrophil chemotaxis, adhesion, phagocytosis, and degranulation; predominantly found as calprotectin (S100A8/A9) which has a wide plethora of intra- and extracellular functions, including adhesion, apoptosis, autophagy, cytoskeletal remodeling, cytokine production, chemotaxis, migration, inflammation, arachidonic acid metabolism, oxidant-scavenging, and PRR signaling
Sell	1.78	L-selectin		Mediates cell adhesion by binding to glycoproteins on neighboring cells
Thbs1	0.589	Thrombospondin 1		Adhesive glycoprotein that mediates cell-to-cell and cell-to-matrix interactions; ligand for CD36

Thy1	1.25	Thy-1 T cell antigen		Cell surface glycoprotein involved in cell adhesion and communication in immune and nerve cells
**Angiogenesis**				
Cd97	0.748	Cluster of differentiation 97	BL-Ac[F2]	GPCR that promotes granulocyte adhesion and migration; activates T cells via binding to CD55; stimulates angiogenesis through binding integrin counterreceptors on endothelial cells
Itgam	2.19	Integrin alpha M	CD11b	Pairs with CD18 to forms Mac-1 aka complement receptor 3; mediates leukocyte activation, adhesion, chemotaxis, migration, phagocytosis, and cell-mediated cytotoxicity; serves as a macrophage marker
Jam3	1.54	Junctional adhesion molecule C		Immunoglobulin that mediates tight junctions between endothelial cells; mediates transepithelial migration of PMNs
S100a8	2.3	S100 calcium-binding protein A8	Calgranulin A	Calcium- and zinc-binding protein involved in pro-inflammatory, antimicrobial, oxidant-scavenging and apoptosis-inducing activities; can induce neutrophil chemotaxis, adhesion, phagocytosis, and degranulation; predominantly found as calprotectin

				(S100A8/A9) which has a wide plethora of intra- and extracellular functions, including adhesion, apoptosis, autophagy, cytoskeletal remodeling, cytokine production, chemotaxis, migration, inflammation, arachidonic acid metabolism, oxidant-scavenging, and PRR signaling
Tgfb1	0.803	Transforming growth factor beta 1		Multifunctional protein that regulates the growth and differentiation of various cell types and is involved in various processes, such as normal development, immune function, microglia function and responses to neurodegeneration; can induce EMT and cell migration in various cell types; frequently acts as an immunosuppressive cytokine in the TME
Vegfa	0.592	Vascular endothelial growth factor A		Glycosylated mitogen that promotes vascular permeability, vasculogenesis, angiogenesis, and cell migration
**Antigen Processing & Presentation**				
Cd1d1	1.45	Cluster of differentiation 1 D1		Murine non-classical class I MHC; primarily presents lipid and glycolipid Ags
Cd1d2	1.64	Cluster of differentiation 1 D2		Pairs with CD1d1 to form the murine non-classical class I MHC, CD1d; primarily presents

				lipid and glycolipid Ags; essential for NKT cell development; presents shorter acyl chain Ags than CD1d1
Cd74	1.22	Cluster of differentiation 74	MHC class II gamma chain	Stabilizes peptide-free class II αβ heterodimers during MHC-Ag complex formation
Clec4a2	1.75	C-type lectin domain family 4 member A2		PRR that, upon binding mannose or fucose, is endocytosed and processed in the Ag presentation pathway
Ctss	2.53	Cathepsin S		Lysosomal protease that participates in processing of Ag by MHC class II
Cyfip2	1.74	Cytoplasmic FMR1-interacting protein 2		Involved in T cell adhesion and p53/TP53-dependent induction of apoptosis
H2-Aa	1.09	Histocompatibility 2, class II antigen A, alpha		MHC class II molecule; presents Ags to $CD4^+$ T cells
H2-Ab1	1.34	Histocompatibility 2, class II antigen A, beta 1		MHC class II molecule; presents Ags to $CD4^+$ T cells
H2-D1	0.841	Histocompatibility 2, D region locus 1		MHC class I molecule; presents Ags to $CD8^+$ T cells

H2-K1	0.421	Histocompatibility 2, K1, K region		MHC class I molecule; presents Ags to $CD8^+$ T cells
H2-Q10	2.19	Histocompatibility 2, Q region locus 10		MHC class I molecule; presents Ags to $CD8^+$ T cells
H2-T23	1.23	Histocompatibility 2, Q region locus 10		MHC class I molecule; presents Ags to $CD8^+$ T cells
Psmb10	0.571	Proteasome subunit beta type 10		20S core β subunit of the proteasome involved in Ag processing to generate class I binding peptides
Tap1	1.1	Transporter antigen peptide 1	Really interesting new gene 4 (RING4)	ATP-binding cassette transporter that pumps degraded cytosolic peptides from the cytosol to the ER for packaging into MHC class I molecules
**Anti-Inflammatory**				
Cd200r1	1.18	Cluster of differentiation 200 receptor 1		Inhibitory receptor for the CD200/OX2 cell surface glycoprotein; limits inflammation by inhibiting the expression of proinflammatory molecules including TNFα, IFNs, and iNOS
Cd274	3.02	Cluster of differentiation 274	Programmed cell death receptor ligand 1 (PD-L1)	Ubiquitously expressed ligand for co-inhibitory receptor PD-1; upregulated by tumors as an immune evasion strategy

Ctla4	2.26	Cytotoxic T lymphocyte antigen 4	CD152	Inhibitory receptor that blocks CD28 costimulation by competitively binding its ligands CD80 and CD86
Pdcd1	0.768	Programmed cell death 1	CD279; PD-1	Checkpoint receptor; inhibits T and NK cell activation
Pdcd1lg2	1.45	Programmed cell death 1 ligand 2		
Lag3	2.17	Lymphocyte activating gene 3	CD223	Inhibitory receptor on activated T cells; binds to ligands, such as FGL1; constitutively expressed on a subset of regulatory Tregs and contributes to their suppressive function; acts as a negative regulator of plasmacytoid DC activation
Nfkb1	0.349	Nuclear factor kappa B subunit 1	p105/p50	One of the NFκB family TFs; inhibits inflammation
Serping1	0.72	Serpin family G member 1	C1-inhibitor	Inflammation-induced acute phase protein that inhibits C1r and C1s proteases in the C1 complex
Tgfb1	0.803	Transforming growth factor beta 1		Multifunctional protein that regulates the growth and differentiation of various cell types and is involved in various processes, such as normal development, immune function, microglia function and responses to

				neurodegeneration; can induce EMT and cell migration in various cell types; frequently acts as an immunosuppressive cytokine in the TME
Tigit	1.28	T cell immunoreceptor with Ig and ITIM domains		Binds with high affinity to the poliovirus receptor, causing increased secretion of IL-10, decreased secretion of IL-12B, and suppressing T cell activation by promoting the generation of mature immunoregulatory DCs
Tnfaip3	1.34	Tumor necrosis factor, alpha-induced protein 3		Ubiquitin-editing enzyme that complexes with ITCH to degrade inflammatory signaling components in the TNF, IL1, and TLR pathways; targets TRAF2, TRAF6, and IKK
Tollip	0.413	Toll interacting protein		Inhibitory adaptor protein; recruits IRAK1 to the IL-1 receptor complex and inhibitively phosphorylates it
**Apoptosis**				
Casp1	0.882	Caspase 1	Interleukin 1β convertase	Cysteine-aspartic acid protease that mediates cleavage-based activation of IL-1β and IL-18; serves as the central enzymatic core of the inflammasome; also induces apoptosis
Casp8	0.536	Caspase 8		Cysteine-aspartic acid protease; cleaves and activates effector caspases CASP3, -4, -6, -7, -9, and -10; initiates pyroptosis through

				cleavage of gasdermin-D; mediates noncanonical cleavage of IL-1β in DCs and macrophages
Cyfip2	1.74	Cytoplasmic FMR1-interacting protein 2		Involved in T cell adhesion and p53/TP53-dependent induction of apoptosis
Fas	1.4	Fragment apoptosis stimulating		Cell surface death receptor; interaction with FAS-ligand triggers an apoptotic signaling cascade; also activates NFκB, ERK1, and MAPK8
Fasl	2.01	Fas ligand	CD178; CD95L; apoptosis antigen ligand (APTL)<br>Tumor necrosis factor ligand superfamily member 6 (TNFSF6)	Ligand for the Fas death receptor; involved in CTL and NK cell-mediated apoptosis
Prkcd	0.892	Protein kinase C delta		Ca(2+)-independent, phospholipid- and DAG-dependent serine/threonine-protein kinase that promotes apoptosis in response to DNA damage but inhibits it during cytokine receptor-initiated cell death; required for oxygen radical production by NADPH oxidase

S100a8	2.3	S100 calcium-binding protein A8	Calgranulin A	Calcium- and zinc-binding protein involved in pro-inflammatory, antimicrobial, oxidant-scavenging and apoptosis-inducing activities; can induce neutrophil chemotaxis, adhesion, phagocytosis, and degranulation; predominantly found as calprotectin (S100A8/A9) which has a wide plethora of intra- and extracellular functions, including adhesion, apoptosis, autophagy, cytoskeletal remodeling, cytokine production, chemotaxis, migration, inflammation, arachidonic acid metabolism, oxidant-scavenging, and PRR signaling
Tnfsf14	2.19	TNF superfamily member 14	CD258; LIGHT; Herpesvirus entry mediator ligand (HVEML)	Cytokine that binds to TNFRSF3/LTBR and TNFRSF14/HVEM; delivers costimulation to T cells; triggers apoptosis of various tumor cells
**Autophagy**				
Irgm2	0.832	Immunity-related GTPase family M member 2	Interferon-inducible protein 1 (IFI1)	Function not fully known, but most likely regulates autophagy and pro-inflammatory cytokine production
S100a8	2.3	S100 calcium-binding protein A8	Calgranulin A	Calcium- and zinc-binding protein involved in pro-inflammatory, antimicrobial, oxidant-scavenging and apoptosis-inducing activities;

				can induce neutrophil chemotaxis, adhesion, phagocytosis, and degranulation; predominantly found as calprotectin (S100A8/A9) which has a wide plethora of intra- and extracellular functions, including adhesion, apoptosis, autophagy, cytoskeletal remodeling, cytokine production, chemotaxis, migration, inflammation, arachidonic acid metabolism, oxidant-scavenging, and PRR signaling
Ubc	0.653	Polyubiquitin C		Serves various roles, including innate immunity, DNA repair, and stimulation of autophagy and the proteasomal response
**B Cell Function**				
Blnk	1.54	B cell linker	Src homology 1 domain-containing leukocyte protein of 65 kDa (SLP-65); Ly57	Functions as a central linker protein downstream of the B cell receptor, bridging SYK kinase to a multitude of signaling pathways and regulating biological outcomes of B cell function and development; plays a role in the activation of ERK/EPHB2, MAP kinase p38 and JNK; modulates AP1 activation; important for the activation of NFκB and NFAT

Btk	1.2	Bruton's tyrosine kinase		Crucial kinase in B cell receptor signal transmission and B cell activation
Cd22	1.67	Cluster of differentiation 22	Sialic acid-binding Ig-like lectin 2 (SIGLEC2)<br><br>B lymphocyte cell adhesion molecule (BL-CAM)	Mediates interactions between B cells; binds CD45
Cd37	2.07	Cluster of differentiation 37	Tetraspanin-26	Cell surface glycoprotein known to complex with integrins and other transmembrane 4 superfamily proteins; may play a role in T cell-B cell interactions
Cd48	2.08	Cluster of differentiation 48	B-lymphocyte activation marker (BLAST-1); signaling lymphocytic activation molecule 2 (SLAMF2)	B cell-specific cellular differentiation Ag; when bound to CD2, promotes T cell activation, and the formation of lipid rafts and caveolae for macrophages
Cd69	1.46	Cluster of differentiation 69	C-type lectin domain family 2, member C	Signal transmitting receptor in lymphocytes, NK cells, and platelets; induced upon T cell activation; involved in lymphocyte proliferation

Cd79b	1.91	Cluster of differentiation 79b	B29	One of the two flanking proteins that initiate signaling downstream of the BCR
Cxcl13	1.58	C-X-C motif chemokine ligand 13	BLC, BCA-1	B cell chemokine induced by type I interferons; participates in GC formation
Fcgr1	2.08	Fc fragment of IgG receptor Ia	CD64	High affinity receptor for the Fc region of γ-Igs; functions in both innate and adaptive immune responses
Fcgr2b	1.41	Fc fragment of IgG receptor IIb	CD32	Low affinity receptor for the Fc region of complexed or aggregated γ-Igs; involved in a variety of effector and regulatory functions such as phagocytosis of immune complexes and modulation of Ab production by B cells; essential for the maintenance of humoral tolerance; acts as a late checkpoint at the level of class-switched memory B cells, plasmablasts or plasma cells; regulates plasma-cell homeostasis and survival
Gpr183	0.932	G protein-coupled receptor 183	EBV-induced G protein-coupled receptor 2	Lymphocyte GPCR that acts as a chemotactic receptor for B cells, T cells, splenic DCs, monocytes/macrophages, and astrocytes
Ikzf1	1.19	IKAROS family zinc finger 1		Transcriptional regulator of hematopoietic cell differentiation; plays a role in T and B cell development

Lyn	0.921	Lck/Yes-related novel kinase		Src family tyrosine kinase that potentiates signaling from the B cell receptor and CD40
Mef2c	1.24	Myocyte enhancer factor 2c		Transcriptional activator that binds specifically to the MEF2 element present in the regulatory regions of many muscle-specific genes; controls cardiac morphogenesis and myogenesis, and is also involved in vascular development; required for B cell survival and proliferation in response to BCR stimulation, efficient IgG1 Ab responses to T cell-dependent Ags, and for normal induction of GC B cells
Ms4a1	2.26	Membrane spanning 4-domains A1	CD20; Bp35	B cell-specific membrane protein that functions as a store-operated Ca(2+) channel component, promoting Ca(2+) influx after BCR activation
Pik3cd	1.19	Phosphatidylinositol-4,5-bisphosphate 3-kinase catalytic subunit delta isoform		A subunit of PI3K; acts downstream of TLR4, TCR, BCR, and CD40; contributes to T helper cell expansion, mast cell development, and neutrophil chemotaxis, extravasation, and respiratory burst

Pik3cg	1.21	Phosphatidylinositol-4,5-bisphosphate 3-kinase catalytic subunit gamma isoform		A subunit of PI3K; modulates leukocyte chemotaxis to inflammatory sites and in response to chemoattractant agents
Pou2f2	1.81	POU domain class 2, transcription factor 2		TF that regulates Ab and IL-6 expression in B cells
Prdm1	1.77	Positive regulatory domain I-binding factor	B lymphocyte-induced maturation protein (BLIMP1)	TF that plays a role in the development, retention, and long-term establishment of T cell, NK cell, and NK-T cells in non-lymphoid organs; drives the maturation of B cell into Ig secreting cells
Spn	1.63	Sialophorin	Leukosialin; CD43	Cell surface sialoglycoprotein expressed by T cells, B cells, monocytes, and granulocytes; promotes lymph node localization in T cells; shunts T cells away from the T_H2 phenotype and towards T_H1; promotes the expression of IFNγ in $CD4^+$ T cells
Syk	1.95	Spleen-associated tyrosine kinase		Critical kinase that transmits signals from the TCR and BCR
Zap70	1.72	Zeta chain of T cell receptor associated protein kinase 70		Tyrosine kinase that plays an essential role in regulation of the adaptive immune response; regulates motility, adhesion, proliferation,

				differentiation, and cytokine expression in T cells; contributes to the development and activation of primary B cells; phosphorylates at least two essential adapter proteins: LAT and LCP2, leading in turn to a large number of signaling molecules being recruited
**Cell Cycle**				
Ccnd3	0.254	Cyclin D3		Regulatory component of the cyclin D3-CDK4 complex that inhibitively phosphorylates members of the retinoblastoma protein family; regulates the cell-cycle during G1/S transition
Cdkn1a	1.39	Cyclin dependent kinase inhibitor 1A	p21; CDK-interaction protein 1 (CIP1)	Binds to and inhibits cyclin-dependent kinase activity, preventing phosphorylation of critical cyclin-dependent kinase substrates and blocking cell cycle progression
**Chemotaxis**				
Ccl2	1.9	C-C motif chemokine ligand 2		Chemoattractant ligand for CCR2 and -4; attracts monocytes and basophils
Ccl3	3.74	C-C motif chemokine ligand 3	Macrophage inflammatory protein 1α (MIP1α)	Chemoattractant ligand for CCR1, -4, and -5

Ccl4	3.44	C-C motif chemokine ligand 4	Macrophage inflammatory protein 1β (MIP1β)	Chemoattractant for NK cells and monocytes; binds to CCR5 receptors
Ccl5	3.21	C-C motif chemokine ligand 5	Regulated upon activation, normally T-expressed, and presumably secreted (RANTES))	Chemoattractant ligand for CCR1, -3, -4, and -5; attracts blood monocytes, memory T helper cells and eosinophils; causes the release of histamine from basophils and activates eosinophils
Ccl7	1.57	C-C motif chemokine ligand 7	Monocyte chemotactic protein 3 (MCP3)	General chemokine that recruits leukocytes to infected tissues; mainly observed in monocyte mobilization
Ccl8	1.56	C-C motif chemokine ligand 8	Monocyte chemoattractant protein 2 (MCP2)	General chemokine that recruits leukocytes to infected tissues
Ccl11	0.972	C-C motif chemokine ligand 11	Eotaxin	Chemoattractant for eosinophils
Ccl12	0.874	C-C motif chemokine ligand 12	Monocyte chemotactic protein 5 (MCP5)	Chemoattractant specific for eosinophils, monocytes, and lymphocytes; found primarily in the lymph nodes and thymus, but can be strongly expressed by macrophages

Ccl24	1.25	C-C motif chemokine ligand 24	Eotaxin-2	Chemoattractant for resting T cells and eosinophils
Ccr1	2.27	C-C motif chemokine receptor 1	MIP1α receptor	Receptor for CCL3, -5, -7, and -23
Ccr3	1.42	C-C motif chemokine receptor 3	CD193	Receptor for a variety of chemokines, including CCL11, CCL26, CCL7, CCL13, CCL5 (RANTES), and CCL15; signals through Ca(2+) flux
Ccr5	1.37	C-C motif chemokine receptor 5	CD195	Receptor for a number of inflammatory CC-chemokines, including CCL3/MIP1α, CCL4/MIP1β, and RANTES; signals via Ca(2+) flux
Ccr7	2.19	C-C chemokine receptor type 7	CD197	Chemokine receptor that activates B and T cells and promotes their homing to secondary lymphoid organs; also stimulates DC expression of MHC class I and II
Ccr9	0.792	C-C motif chemokine receptor 9		Receptor for CCL25; increases intracellular Ca(2+) levels upon ligand binding
Ccrl2	2.19	C-C chemokine receptor-like 2		Stabilizes TLR4 surface expression on macrophages

Cxcl1	1.94	C-X-C motif chemokine ligand 1	GRO1 oncogene	Chemoattractant ligand for CXCR2; plays a role in inflammation and as a chemoattractant for neutrophils
Cxcl2	3.12	C-X-C motif chemokine ligand 2	Macrophage inflammatory protein 2-alpha (MIP2α); GRO2 oncogene	Chemokine produced by activated monocytes and neutrophils and expressed at sites of inflammation
Cxcl3	3.93	C-X-C motif chemokine ligand 3	GRO3 oncogene	Ligand for CXCR2; attracts neutrophils
Cxcl9	0.947	C-X-C motif chemokine ligand 9	Humig	Chemoattractant ligand for CXCR3; attracts activated T cells
Cxcl10	1.32	C-X-C motif chemokine ligand 10	IFNγ-induced protein 10 (IP-10)	Macrophage, DC, T cell, and NK cell chemoattractant secreted by several cell types in response to IFNγ; binds to CXCR3
Cxcl11	1.24	C-X-C motif chemokine ligand 11		Dominant ligand for CXCR3; attracts activated T cells; strongly induced by IFNγ
Cxcl12	1.29	C-X-C motif chemokine ligand 12	Stromal cell-derived factor 1 (SDF1)	Ubiquitously expressed chemokine that acts a strong chemoattractant for lymphocytes
Cxcl13	1.58	C-X-C motif chemokine ligand 13	BLC, BCA-1	B cell chemokine induced by type I interferons; participates in GC formation

Cxcl16	1.68	C-X-C motif chemokine ligand 16		Chemoattractant for T cells and NKT cells produced by DCs in response to IFNγ and TNFα
Cxcr2	2.57	C-X-C motif chemokine receptor 2	CD182; IL-8 receptor B	Receptor for IL-8 and CXCL3; powerful chemoattractant for neutrophils
Cxcr3	2.23	C-X-C motif chemokine receptor 3	CD183	Induces integrin activation, cytoskeletal remodeling, and chemotaxis; expressed by T cells and NK cells; prominently expressed in effector and memory T cells
Cxcr4	1.74	C-X-C motif chemokine receptor 4	CD184; fusin	Alpha-chemokine receptor specific for SDF1 aka CXCL12
Cxcr6	2.3	C-X-C motif chemokine receptor 6	CD186	Receptor for the C-X-C chemokine CXCL16; expressed in several T lymphocyte subsets and bone marrow stromal cells
Gpr183	0.932	G protein-coupled receptor 183	EBV-induced G protein-coupled receptor 2	Lymphocyte GPCR that acts as a chemotactic receptor for B cells, T cells, splenic DCs, monocytes/macrophages, and astrocytes
Isg15	2.41	Interferon-stimulated gene 15		Ubiquitin-like protein that binds intracellular target proteins upon activation by IFNα or β; can also be secreted to induce NK cell proliferation, act as a chemoattractant for

				neutrophils, and induce IFNγ upon binding to ITGAL/ITGB2
Itgam	2.19	Integrin alpha M	CD11b	Pairs with CD18 to form Mac-1 aka complement receptor 3; mediates leukocyte activation, adhesion, chemotaxis, migration, phagocytosis, and cell-mediated cytotoxicity; serves as a macrophage marker
Pik3cd	1.19	Phosphatidylinositol-4,5-bisphosphate 3-kinase catalytic subunit delta isoform		A subunit of PI3K; acts downstream of TLR4, TCR, BCR, and CD40; contributes to T helper cell expansion, mast cell development, and neutrophil chemotaxis, extravasation, and respiratory burst
S100a8	2.3	S100 calcium-binding protein A8	Calgranulin A	Calcium- and zinc-binding protein involved in pro-inflammatory, antimicrobial, oxidant-scavenging and apoptosis-inducing activities; can induce neutrophil chemotaxis, adhesion, phagocytosis, and degranulation; predominantly found as calprotectin (S100A8/A9) which has a wide plethora of intra- and extracellular functions, including adhesion, apoptosis, autophagy, cytoskeletal remodeling, cytokine production, chemotaxis, migration, inflammation, arachidonic acid

				metabolism, oxidant-scavenging, and PRR signaling
Xcl1	0.805	X-C motif chemokine ligand 1	Lymphotaxin	Chemoattractant for lymphocytes but not monocytes or neutrophils; mediates medullary accumulation of thymic DCs and contributes to Treg development
**Complement & Humoral Immunity**				
C1qa	0.818	Complement C1q subcomponent subunit A		A chain of the C1q complex, which acts as the Ag-Ab-binding subunit of the C1 complex
C1qb	1.17	Complement C1q subcomponent subunit B		B chain of the C1q complex, which acts as the Ag-Ab-binding subunit of the C1 complex
C1ra	0.793	Complement C1r-A subcomponent		Proteolytic subunit of the C1 complex that enzymatically cleaves C1s
C1s1	1.07	Complement component 1s	C1 esterase	Serine protease that enzymatically cleaves C4 and C2
C2	0.757	Complement component 2		Cleaved by activated factor C1 into two fragments: C2b and C2a; C2a combines with C4b to generate C3 or C5 convertase

C3	1.17	Complement component 3		Cleaved by C3 convertase to form C3a and C3b, an anaphalotoxin and an opsonizing agent, respectively
C3ar1	1.5	Complement component 3a receptor 1		GPCR that binds to C3a, activating chemotaxis, granule enzyme release, superoxide anion production, and bacterial opsonization
C4b	0.684	Complement component 4B		Mediates interactions between Ab-bound Ags and other complement components
C6	1.02	Complement component 6		Part of the membrane attack complex
Cd55	2	Cluster of differentiation 55	Complement decay-accelerating factor	Cell surface glycoprotein that interacts with surface-bound C4b and inhibits its conversion of C2 to C2b
Cfb	1.37	Complement factor B		Alternate complement pathway component; when cleaved, produces a serine protease that binds to C3b to form C3 convertase
Cfd	3.27	Complement factor D	Adipsin	Chymotrypsin-family peptidase that cleaves factor B when the latter is complexed with factor C3b, activating C3 convertase
Cfp	1.5	Complement factor properdin		Alternate complement pathway component; when cleaved, produces a serine protease that binds to C3b to form C3 convertase

Fcgr1	2.08	Fc fragment of IgG receptor Ia	CD64	High affinity receptor for the Fc region of γ-Igs; functions in both innate and adaptive immune responses
Fcgr2b	1.41	Fc fragment of IgG receptor IIb	CD32	Low affinity receptor for the Fc region of complexed or aggregated γ-Igs; involved in a variety of effector and regulatory functions such as phagocytosis of immune complexes and modulation of Ab production by B cells; essential for the maintenance of humoral tolerance; acts as a late checkpoint at the level of class-switched memory B cells, plasmablasts or plasma cells; regulates plasma-cell homeostasis and survival
Fcgr4	3.21	Fragment crystallizable gamma receptor 4	Fc receptor-like 3 (Fcrl3); CD16-2	Putative mouse ortholog to human FcγRIIIA
**Costimulation**				
Cd28	2.48	Cluster of differentiation 28		Essential T cell co-receptor that enhances T cell activation, proliferation, cytokine production, and survival; binds to CD80 and CD86

Cd40	1.9	Cluster of differentiation 40		APC-expressed costimulatory protein that binds to CD40L on $CD4^+$ T cells, causing activation of both
Cd80	1.04	Cluster of differentiation 80	B7-1	One of the two ligands for the CD28 costimulatory receptor and the CTLA4 inhibitory receptor, the other being CD86
Cd86	0.98	Cluster of differentiation 86	B7-2	One of the two ligands for the CD28 costimulatory receptor and the CTLA4 inhibitory receptor, the other being CD80
Icam1	1.69	Intracellular adhesion molecule 1	CD54	Cell surface glycoprotein that serves as strong adhesive ligand for LFA-1; important for leukocyte mobility and costimulation
Icos	2.45	Inducible T cell costimulator	CD278	Enhances all basic T cell responses to foreign Ag; essential both for efficient interaction between T and B cells and for normal Ab responses to T cell-dependent Ags
Ptprc	1.5	Protein tyrosine phosphatase receptor type C	CD45; leukocyte common antigen (LCA)	Delivers costimulation during T cell activation upon binding to its ligand DPP4; dephosphorylates Lyn and suppresses JAK kinases

Tnfsf14	2.19	TNF superfamily member 14	CD258; LIGHT; Herpesvirus entry mediator ligand (HVEML)	Cytokine that binds to TNFRSF3/LTBR and TNFRSF14/HVEM; delivers costimulation to T cells; triggers apoptosis of various tumor cells
Tnfrsf4	1.61	Tumor necrosis factor receptor superfamily member 4	OX40; CD134	Receptor for TNFSF4/OX40L/GP34; costimulatory molecule implicated in long-term T cell immunity; activates NFκB through its interaction with adaptor proteins TRAF2 and TRAF5; suppresses apoptosis through upregulation of BCL2
Tnfrsf11a	1.16	Tumor necrosis factor receptor superfamily member 11A	Receptor activator of NFκB (RANK)	Recruits TRAFs and activates NFκB and JNK activation upon binding to RANKL on the surface of T cells
Tnfrsf14	0.731	Tumor necrosis factor receptor superfamily member 14		
**Cytokines**				
Csf1	0.698	Macrophage colony-stimulating factor 1		Cytokine that promote activation and survival of monocytes

Csf1r	1.11	Macrophage colony-stimulating factor 1 receptor	CD115	Receptor for CSF1; promotes release of inflammatory cytokines in response to IL-34 and CSF1
Ifnar1	0.731	Interferon-alpha/beta receptor alpha chain		Component of the receptor for type I IFNs, binding of which activates the JAK-STAT pathway
Ifng	1.93	Interferon gamma		T and NK cell-secreted inflammatory cytokine that stimulates cytolysis, activates macrophages, and stimulates MHC class II expression
Ifngr1	0.707	Interferon gamma receptor 1	CD54	One of the two components of the IFNγ receptor; stimulates activation of the JAK/STAT signaling pathway
Il1a	3.29	Interleukin 1 alpha	Hematopoietin-1	Cytokine produced by monocytes and macrophages in response to cell injury; stimulates thymocyte proliferation by inducing IL-2 release; also stimulates B cell maturation and proliferation, and fibroblast growth factor activity
Il1b	2.04	Interleukin 1 beta	Catabolin	One of the two primary inflammatory cytokines produced by the inflammasome (the other one being IL-18); induces neutrophil influx and activation, T cell activation and

				cytokine production, B cell activation and Ab production, fibroblast proliferation, and collagen production; synergizes with IL-12 to induce IFNγ synthesis from T_H1 cells
Il1rap	1.27	Interleukin 1 receptor accessory protein		Co-receptor for several ligands, including IL-1R1 in the IL-1 pathway, IL-RL1 in the IL-33 pathway, IL-1RL2 in the IL-36 pathway; signaling involves Tollip, MyD88, IRAK1, and IRAK2
Il2ra	2	Interleukin 2 receptor subunit alpha	CD25	Alpha chain of the IL-2 receptor
Il2rg	1.53	Interleukin 2 receptor subunit gamma	Common gamma chain; CD132	Common subunit for the receptors for a variety of interleukins, including IL-2, -4, -7, and -21
Il6	2.7	Interleukin 6		Pro-inflammatory cytokine that signals through the JAK and STAT pathways
Il7r	2.16	Interleukin 7 receptor	CD127	Receptor for IL-7
Il10	1.01	Interleukin 10	Cytokine synthesis inhibitory factor (CSIF)	Major immunoregulatory cytokine that inhibits production of pro-inflammatory cytokines, including GM-CSF, G-CSF, IL-1α, IL-1β, IL-6, IL-8, and TNFα; also interferes with Ag

				presentation by reducing expression of MHC class II and costimulatory molecules, thereby inhibiting their ability to induce T cell activation
Il12rb1	1.07	Interleukin 12 receptor subunit beta 1	CD212	Cytokine receptor component that associates with IL12RB2 to IL23R
Il13ra1	0.717	Interleukin 13 receptor subunit alpha 1	CD213a1	Pairs with IL4RA to form to form the IL-13 receptor; may mediate the signaling processes that lead to the activation of JAK1, STAT3, and STAT6 induced by IL-4 and -13
Il18r1	1.36	Interleukin 18 receptor 1	CD218a	Receptor for IL-18
Tgfb1	0.803	Transforming growth factor beta 1		Multifunctional protein that regulates the growth and differentiation of various cell types and is involved in various processes, such as normal development, immune function, microglia function and responses to neurodegeneration; can induce EMT and cell migration in various cell types; frequently acts as an immunosuppressive cytokine in the TME
Tnf	3.73	Tumor necrosis factor	Cachectin	Inflammatory cytokine mainly produced by macrophages; binds to TNFRSF1A/TNFR1 and

				TNFRSF1B/TNFBR; capable of inducing cell death in certain tumor cell lines
Tnfsf14	2.19	TNF superfamily member 14	CD258; LIGHT; Herpesvirus entry mediator ligand (HVEML)	Cytokine that binds to TNFRSF3/LTBR and TNFRSF14/HVEM; delivers costimulation to T cells; triggers apoptosis of various tumor cells
Tnfrsf4	1.61	Tumor necrosis factor receptor superfamily member 4	OX40; CD134	Receptor for TNFSF4/OX40L/GP34; costimulatory molecule implicated in long-term T cell immunity; activates NFκB through its interaction with adaptor proteins TRAF2 and TRAF5; suppresses apoptosis through upregulation of BCL2
Tnfrsf11a	1.16	Tumor necrosis factor receptor superfamily member 11A	Receptor activator of NFκB (RANK)	Recruits TRAFs and activates NFκB and JNK activation upon binding to RANKL on the surface of T cells
Tnfrsf14	0.731	Tumor necrosis factor receptor superfamily member 14		
**Cytotoxicity**				
Gzmb	3.8	Granzyme B	Fragmentin 2	Abundant protease in the cytosolic granules of cytotoxic T and NK cells that activates caspase-

				mediated cell death when delivered into the target cell through the immunological synapse
Gzmk	3.36	Granzyme K	Tryptase II	Granule-secreted, pro-apoptotic serine protease found in the cytoplasmic granules of CTLs
Fcgr4	3.21	Fragment crystallizable gamma receptor 4	Fc receptor-like 3 (Fcrl3); CD16-2	Putative mouse ortholog to human FcγRIIIA
Itgam	2.19	Integrin alpha M	CD11b	Pairs with CD18 to form Mac-1 aka complement receptor 3; mediates leukocyte activation, adhesion, chemotaxis, migration, phagocytosis, and cell-mediated cytotoxicity; serves as a macrophage marker
S100a8	2.3	S100 calcium-binding protein A8	Calgranulin A	Calcium- and zinc-binding protein involved in pro-inflammatory, antimicrobial, oxidant-scavenging and apoptosis-inducing activities; can induce neutrophil chemotaxis, adhesion, phagocytosis, and degranulation; predominantly found as calprotectin (S100A8/A9) which has a wide plethora of intra- and extracellular functions, including adhesion, apoptosis, autophagy, cytoskeletal remodeling, cytokine production, chemotaxis, migration, inflammation, arachidonic acid

				metabolism, oxidant-scavenging, and PRR signaling
**Dendritic Cell Function**				
Casp8	0.536	Caspase 8		Cysteine-aspartic acid protease; cleaves and activates effector caspases CASP3, -4, -6, -7, -9, and -10; initiates pyroptosis through cleavage of gasdermin-D; mediates noncanonical cleavage of IL-1β in DCs and macrophages
Cxcl16	1.68	C-X-C motif chemokine ligand 16		Chemoattractant for T cells and NKT cells produced by DCs in response to IFNγ and TNFα
Gpr183	0.932	G protein-coupled receptor 183	EBV-induced G protein-coupled receptor 2	Lymphocyte GPCR that acts as a chemotactic receptor for B cells, T cells, splenic DCs, monocytes/macrophages, and astrocytes
Tigit	1.28	T cell immunoreceptor with Ig and ITIM domains		Binds with high affinity to the poliovirus receptor, causing increased secretion of IL-10, decreased secretion of IL-12B, and suppressing T cell activation by promoting the generation of mature immunoregulatory DCs
Itgax	1.47	Integrin alpha X	CD11c	Adhesion molecule; signature marker of Ag-presenting DCs

**Growth/Proliferation**				
Lcn2	1.97	Lipocalin 2	Neutrophil gelatinase-associated lipocalin (NGAL)	Neutrophil-secreted factor that sequesters iron-containing siderophores; also functions as a growth factor
Map2k1	0.266	Dual specificity mitogen-activated protein kinase kinase 1	MAPK/ERK kinase 1 (MEK1)	Essential component of the MAP kinase signal transduction pathway; participates in numerous biological functions, including cell growth, adhesion, survival, differentiation, transcription, metabolism, and cytoskeletal remodeling
Tgfb1	0.803	Transforming growth factor beta 1		Multifunctional protein that regulates the growth and differentiation of various cell types and is involved in various processes, such as normal development, immune function, microglia function and responses to neurodegeneration; can induce EMT and cell migration in various cell types; frequently acts as an immunosuppressive cytokine in the TME
Vegfa	0.592	Vascular endothelial growth factor A		Glycosylated mitogen that promotes vascular permeability, vasculogenesis, angiogenesis, and cell migration
**Hematopoiesis**				

Hck	1.76	Hematopoietic cell kinase		Src family tyrosine kinase that mediates secretory lysosome mobilization, degranulation, and activation of NADPH oxidase
Ikzf1	1.19	IKAROS family zinc finger 1		Transcriptional regulator of hematopoietic cell differentiation; plays a role in T and B cell development
Jam3	1.54	Junctional adhesion molecule C		Immunoglobulin that mediates tight junctions between endothelial cells; mediates transepithelial migration of PMNs; promotes chemotaxis of vascular endothelial cells and stimulates angiogenesis
Stat5b	0.31	Signal transducer and activator of transcription 5b		Carries out a dual function: signal transduction and activation of transcription; positively regulates hematopoietic/erythroid differentiation.
**Inflammation**				
Bst2	1.31	Bone marrow stromal cell antigen 2	Tethrin; CD317	IFN-induced antiviral factor that blocks budding of enveloped viruses by directly tethering nascent virions to the membranes of infected cells

Casp1	0.882	Caspase 1	Interleukin 1β convertase	Cysteine-aspartic acid protease that mediates cleavage-based activation of IL-1β and IL-18; serves as the central enzymatic core of the inflammasome; also induces apoptosis
Casp8	0.536	Caspase 8		Cysteine-aspartic acid protease; cleaves and activates effector caspases CASP3, -4, -6, -7, -9, and -10; initiates pyroptosis through cleavage of gasdermin-D; mediates noncanonical cleavage of IL-1β in DCs and macrophages
Cd38	2.02	Cluster of differentiation 38	ADP-ribosyl cyclase 1	Synthesizes the second messengers cyclic ADP-ribose and NADPH; appears to play a critical role in inflammation, although its exact immunological function(s) remain(s) poorly defined
Cebpb	1.28	CCAAT/enhancer-binding protein beta		Critical macrophage TF that promotes expression of several acute-phase and inflammatory cytokine genes, including Il6
Csf1r	1.11	Macrophage colony-stimulating factor 1 receptor	CD115	Receptor for CSF1; promotes release of inflammatory cytokines in response to IL-34 and CSF1
Ctsh	1.25	Cathepsin H		Lysosomal protease; increased in macrophages in response to IFNγ

Ifnar1	0.731	Interferon-alpha/beta receptor alpha chain		Component of the receptor for type I IFNs, binding of which activates the JAK-STAT pathway
Ifng	1.93	Interferon gamma		T and NK cell-secreted inflammatory cytokine that stimulates cytolysis, activates macrophages, and stimulates MHC class II expression
Ifngr1	0.707	Interferon gamma receptor 1	CD54	One of the two components of the IFNγ receptor; stimulates activation of the JAK/STAT signaling pathway
Il1b	2.04	Interleukin 1 beta	Catabolin	One of the two primary inflammatory cytokines produced by the inflammasome (the other one being IL-18); induces neutrophil influx and activation, T cell activation and cytokine production, B cell activation and Ab production, fibroblast proliferation, and collagen production; synergizes with IL-12 to induce IFNγ synthesis from T_H1 cells
Il6	2.7	Interleukin 6		Pro-inflammatory cytokine that signals through the JAK and STAT pathways
Il12rb1	1.07	Interleukin 12 receptor subunit beta 1	CD212	Cytokine receptor component that associates with IL12RB2 to IL23R

Il18r1	1.36	Interleukin 18 receptor 1	CD218a	Receptor for IL-18
Irf1	1.71	Interferon regulatory factor 1		Transcriptional regulator that promotes inflammatory innate and adaptive immune responses
Irf4	1.2	Interferon regulatory factor 4		Transcriptional activator that complexes with BATF and binds ISREs within the promoters of multiple genes involved in inflammation
Irf7	2.22	Interferon regulatory factor 7		Key transcriptional regulator of type I IFN-dependent immune responses; promotes transcription of IFNα and -β
Irf8	1.12	Interferon regulatory factor 8		TF that regulates of lineage commitment in myeloid cell maturation; promotes monocyte and plasmacytoid DC development
Irgm2	0.832	Immunity-related GTPase family M member 2	Interferon-inducible protein 1 (IFI1)	Function not fully known, but most likely regulates autophagy and pro-inflammatory cytokine production
Isg15	2.41	Interferon-stimulated gene 15		Ubiquitin-like protein that binds intracellular target proteins upon activation by IFNα or β; can also be secreted to induce NK cell proliferation, act as a chemoattractant for

				neutrophils, and induce IFNγ upon binding to ITGAL/ITGB2
Isg20	0.855	Interferon-stimulated gene 20		IFN-induced antiviral exoribonuclease that acts on ssRNA with minor activity towards ssDNA
Jak1	0.709	Janus kinase 1		Essential tyrosine kinase involved signal transduction in type I and II cytokines and IFNs
Jak2	0.797	Janus kinase 2		Tyrosine kinase that participates in IFN and IL6ST signaling cascades
Mefv	2.79	Mediterranean fever	Marenostrin; pyrin	Involved in the regulation of innate immunity and the inflammatory response in response to IFNγ; both stimulates and restrains the inflammasome; also acts as a mediator of pyroptosis
Nlrp3	1.75	NACHT domain-, leucine-rich repeat-, and PYD-containing protein 3	Cryopyrin	PRR with a wide diversity of recognized targets that activates the NLRP3 inflammasome consisting of NLRP3, PYCARD, and caspase-1/-8
Nos2	4.12	Inducible nitric oxide synthase (iNOS)		Produces reactive oxygen species and contributes to inflammatory cytokine production

Pik3cd	1.19	Phosphatidylinositol-4,5-bisphosphate 3-kinase catalytic subunit delta isoform		A subunit of PI3K; acts downstream of TLR4, TCR, BCR, and CD40; contributes to T helper cell expansion, mast cell development, and neutrophil chemotaxis, extravasation, and respiratory burst
Pik3cg	1.21	Phosphatidylinositol-4,5-bisphosphate 3-kinase catalytic subunit gamma isoform		A subunit of PI3K; modulates leukocyte chemotaxis to inflammatory sites and in response to chemoattractant agents
S100a8	2.3	S100 calcium-binding protein A8	Calgranulin A	Calcium- and zinc-binding protein involved in pro-inflammatory, antimicrobial, oxidant-scavenging and apoptosis-inducing activities; can induce neutrophil chemotaxis, adhesion, phagocytosis, and degranulation; predominantly found as calprotectin (S100A8/A9) which has a wide plethora of intra- and extracellular functions, including adhesion, apoptosis, autophagy, cytoskeletal remodeling, cytokine production, chemotaxis, migration, inflammation, arachidonic acid metabolism, oxidant-scavenging, and PRR signaling

Spn	1.63	Sialophorin	Leukosialin; CD43	Cell surface sialoglycoprotein expressed by T cells, B cells, monocytes, and granulocytes; promotes lymph node localization in T cells; shunts T cells away from the T_H2 phenotype and towards T_H1; promotes the expression of IFNγ in $CD4^+$ T cells
Tbk1	1.05	TANK-binding kinase 1		Coordinates the activation of IRF3 and NFκB and induction of type I IFNs
Tnf	3.73	Tumor necrosis factor	Cachectin	Inflammatory cytokine mainly produced by macrophages; binds to TNFRSF1A/TNFR1 and TNFRSF1B/TNFBR; capable of inducing cell death in certain tumor cell lines
Traf6	0.755	Tumor necrosis factor receptor-associated factor 6		Adaptor protein that acts in the CD40 signaling cascade; promotes inflammation, IL-6, and TNFα
**Inhibition**				
Bcl2l1	0.729	B cell lymphoma 2 like 1	Protein phosphatase 1	Potent inhibitor of caspase-mediated cell death
Ccnd3	0.254	Cyclin D3		Regulatory component of the cyclin D3-CDK4 complex that inhibitively phosphorylates members of the retinoblastoma protein

				family; regulates the cell-cycle during G1/S transition
Cd47	0.743	Cluster of differentiation 47	Integrin-associated protein (IAP)	Partners with membrane integrins to serve as an inhibitor of phagocytosis
Cd200r1	1.18	Cluster of differentiation 200 receptor 1		Inhibitory receptor for the CD200/OX2 cell surface glycoprotein; limits inflammation by inhibiting the expression of proinflammatory molecules including TNFα, IFNs, and iNOS
Cd274	2.49	Cluster of differentiation 274	Programmed cell death receptor ligand 1 (PD-L1)	Ubiquitously expressed ligand for co-inhibitory receptor PD-1; upregulated by tumors as an immune evasion strategy
Cdkn1a	1.39	Cyclin dependent kinase inhibitor 1A	p21; CDK-interaction protein 1 (CIP1)	Binds to and inhibits cyclin-dependent kinase activity, preventing phosphorylation of critical cyclin-dependent kinase substrates and blocking cell cycle progression
Ctla4	2.26	Cytotoxic T lymphocyte antigen 4	CD152	Inhibitory receptor that blocks CD28 costimulation by competitively binding its ligands CD80 and CD86
Cyld	0.595	Cylindromatosis lysine 63 deubiquitinase		Inhibits NFκB activation by deubiquitinating upstream signaling factors; inhibits Wnt signaling; restricts polyubiquitination of RIPK1 and -2, thereby limiting necroptosis

Ido1	1.39	Indoleamine 2,3-dioxygenase 1		Initiates catabolism of tryptophan; limits immunopathology by inhibiting T cell division
Il1r2	1.07	Interleukin 1 receptor type II	CD121b	Non-signaling receptor for IL-1α, -β, and RN; serves as a decoy receptor by competitive binding to IL-1β and preventing its binding to IL1R1
Irak3	1.89	Interleukin-1 receptor-associated kinase 3		Adaptor protein that negatively regulates TLR signaling; predominantly expressed in monocytes and macrophages
Foxp3	1.56	Forkhead box P3	DIETER	Master TF for Tregs; represses expression of Il2 and Ifng; activates expression of Tnfrsf18, Il2ra, and Ctla4
Fcgr2b	1.41	Fc fragment of IgG receptor IIb	CD32	Low affinity receptor for the Fc region of complexed or aggregated γ-Igs; involved in a variety of effector and regulatory functions such as phagocytosis of immune complexes and modulation of Ab production by B cells; essential for the maintenance of humoral tolerance; acts as a late checkpoint at the level of class-switched memory B cells, plasmablasts or plasma cells; regulates plasma-cell homeostasis and survival

Klrg1	1.08	Killer cell lectin-like receptor, subfamily G, member 1	C-type lectin domain family 15, member A (CLEC15A)<br>Mast cell function-associated antigen (MAFA)	NK and T cell inhibitory receptor; binds to non-MHC ligands
Lag3	2.17	Lymphocyte activating gene 3	CD223	Inhibitory receptor on activated T cells; binds to ligands, such as FGL1; constitutively expressed on a subset of regulatory Tregs and contributes to their suppressive function; acts as a negative regulator of plasmacytoid DC activation
Nfkbia	1.65	Nuclear factor kappa B inhibitor alpha		Inhibits activity of REL dimers by masking of their nuclear localization signals
Nlrc5	0.956	NLR family CARD domain containing 5	NOD27	Inhibits NFκB and type I IFN signaling pathways; may also regulate the type II IFN signaling pathway
Pdcd1	0.768	Programmed cell death 1	CD279; PD-1	Checkpoint receptor; inhibits T and NK cell activation
Pdcd1lg2	1.45	Programmed cell death 1 ligand 2		

Serping1	0.72	Serpin family G member 1	C1-inhibitor	Inflammation-induced acute phase protein that inhibits C1r and C1s proteases in the C1 complex
Socs1	1.93	Suppressor of cytokine signaling 1		Inhibits JAK proteins; negative regulator of IL-6
Socs3	0.874	Suppressor of cytokine signaling 3		Inhibits IL6ST and JAK2; negative regulator of IL-6
Sh2b2	1.64	Src homology 2B adaptor protein 2		Adapter protein for several members of the tyrosine kinase receptor family; involved in multiple signaling pathways; may be involved in coupling from immunoreceptor to Ras signaling; acts as a negative regulator of cytokine signaling in collaboration with CBL; may induce cytoskeletal reorganization via interaction with Vav3
Tank	0.975	TRAF family member-associated NFκB activator		Inhibitory protein that sequesters TRAFs in the cytoplasm, constitutively binds TBK1, and serves as a negative regulator of NFκB
Tgfb1	0.803	Transforming growth factor beta 1		Multifunctional protein that regulates the growth and differentiation of various cell types and is involved in various processes, such as normal development, immune function, microglia function and responses to

				neurodegeneration; can induce EMT and cell migration in various cell types; frequently acts as an immunosuppressive cytokine in the TME
Tigit	1.28	T cell immunoreceptor with Ig and ITIM domains		Binds with high affinity to the poliovirus receptor, causing increased secretion of IL-10, decreased secretion of IL-12B, and suppressing T cell activation by promoting the generation of mature immunoregulatory DCs
Tollip	0.413	Toll interacting protein		Inhibitory adaptor protein; recruits IRAK1 to the IL-1 receptor complex and inhibitively phosphorylates it
**Interferon Response**				
Bst2	1.31	Bone marrow stromal cell antigen 2	Tethrin; CD317	IFN-induced antiviral factor that blocks budding of enveloped viruses by directly tethering nascent virions to the membranes of infected cells
Ctsh	1.25	Cathepsin H		Lysosomal protease; increased in macrophages in response to IFNγ
Ifih1	1.43	Interferon induced with helicase C domain 1	Helicard; melanoma differentiation-	PRR for cytoplasmic dsRNA; upon target recognition, associates with MAVS to activate TNK1 and IKBKE, which phosphorylate IRF3

			associated protein 5 (MDA5)	and -7, which, in turn, activate transcription of IFNα and -β
Ifitm1	0.626	Interferon-induced transmembrane protein 1	CD225	IFN-induced antiviral protein implicated in cell adhesion and control of cell growth and migration
Ifnar1	0.731	Interferon-alpha/beta receptor alpha chain		Component of the receptor for type I IFNs, binding of which activates the JAK-STAT pathway
Ifng	1.93	Interferon gamma		T and NK cell-secreted inflammatory cytokine that stimulates cytolysis, activates macrophages, and stimulates MHC class II expression
Ifngr1	0.707	Interferon gamma receptor 1	CD54	One of the two components of the IFNγ receptor; stimulates activation of the JAK/STAT signaling pathway
Irf1	1.71	Interferon regulatory factor 1		Transcriptional regulator that promotes inflammatory innate and adaptive immune responses
Irf4	1.2	Interferon regulatory factor 4		Transcriptional activator that complexes with BATF and binds ISREs within the promoters of multiple genes involved in inflammation

Irf7	2.22	Interferon regulatory factor 7		Key transcriptional regulator of type I IFN-dependent immune responses; promotes transcription of IFNα and -β
Irf8	1.12	Interferon regulatory factor 8		TF that regulates of lineage commitment in myeloid cell maturation; promotes monocyte and plasmacytoid DC development
Irgm2	0.832	Immunity-related GTPase family M member 2	Interferon-inducible protein 1 (IFI1)	Function not fully known, but most likely regulates autophagy and pro-inflammatory cytokine production
Isg15	2.41	Interferon-stimulated gene 15		Ubiquitin-like protein that binds intracellular target proteins upon activation by IFNα or β; can also be secreted to induce NK cell proliferation, act as a chemoattractant for neutrophils, and induce IFNγ upon binding to ITGAL/ITGB2
Isg20	0.855	Interferon-stimulated gene 20		IFN-induced antiviral exoribonuclease that acts on ssRNA with minor activity towards ssDNA
Jak1	0.709	Janus kinase 1		Essential tyrosine kinase involved signal transduction in type I and II cytokines and IFNs
Mefv	2.79	Mediterranean fever	Marenostrin; pyrin	Involved in the regulation of innate immunity and the inflammatory response in response to IFNγ; both stimulates and restrains the

				inflammasome; also acts as a mediator of pyroptosis
Mx2	1.03	Myxovirus resistance protein 2		IFN-induced dynamin-like GTPase with potent antiviral activity against HIV-1
Tbk1	1.05	TANK-binding kinase 1		Coordinates the activation of IRF3 and NFκB and induction of type I IFNs
**Ion Transport**				
App	0.801	Amyloid-beta precursor protein		Cell surface receptor and transmembrane precursor protein that is cleaved by secretases to form a number of peptides; involved in cell mobility, copper homeostasis, and oxidative stress
Ms4a1	2.79	Membrane spanning 4-domains A1	CD20; Bp35	B cell-specific membrane protein that functions as a store-operated Ca(2+) channel component, promoting Ca(2+) influx after BCR activation
Slc11a1	2.2	Natural resistance-associated macrophage protein 1		Macrophage-specific metal ion transporter; uptakes divalent metal cations to neutralize ROSs
**IRAKs & TRAFs**				

Irak1	0.24	Interleukin-1 receptor-associated kinase 1		Adaptor protein involved in TLR and IL-1 signaling; recruited to TLRs by MyD88 and phosphorylated by IRAK4; promotes the degradation of TIRAP
Irak2	1.49	Interleukin-1 receptor-associated kinase 2		Adaptor protein involved in TLR and IL-1 signaling
Irak3	1.89	Interleukin-1 receptor-associated kinase 3		Adaptor protein that negatively regulates TLR signaling; predominantly expressed in monocytes and macrophages
Traf6	0.755	Tumor necrosis factor receptor-associated factor 6		Adaptor protein that acts in the CD40 signaling cascade; promotes inflammation, IL-6, and TNFα
**JAK-STAT Pathway**				
Jak1	0.709	Janus kinase 1		Essential tyrosine kinase involved signal transduction in type I and II cytokines and IFNs
Jak2	0.797	Janus kinase 2		Tyrosine kinase that participates in IFN and IL6ST signaling cascades
Stat1	1.53	Signal transducer and activator of transcription 1		Transcriptional activator that mediates cellular responses to IFNs, cytokines, and other growth factors

Stat3	0.649	Signal transducer and activator of transcription 3		Transcriptional activator of genes involved in cell growth and apoptosis; activated by JAKs
Stat4	1.25	Signal transducer and activator of transcription 4		Essential TF for T_H1 $CD4^+$ T cell development and IFNγ production; also promotes expression of MyD88
Stat5b	0.31	Signal transducer and activator of transcription 5b		Carries out a dual function: signal transduction and activation of transcription; positively regulates hematopoietic/erythroid differentiation.
Stat6	0.571	Signal transducer and activator of transcription 6		Essential TF for T_H2 CD4+ T cell and macrophage function and M2 macrophage polarization
**Kinases**				
Btk	1.2	Bruton's tyrosine kinase		Crucial kinase in B cell receptor signal transmission and B cell activation
Hck	1.76	Hematopoietic cell kinase		Src family tyrosine kinase that mediates secretory lysosome mobilization, degranulation, and activation of NADPH oxidase

Itk	2.06	Interleukin-2-inducible T cell kinase	LYK	Key actor in the TCR signaling cascade; phosphorylates PLCγ1, LAT, and LCP2
Lck	1.98	Lymphocyte cell kinase		Src family tyrosine kinase that acts as one of the main signaling intermediaries downstream of the TCR; constitutively associated with the cytoplasmic portion of CD4
Lyn	0.921	Tyrosine-protein kinase Lyn		Src family tyrosine kinase that potentiates signaling from the B cell receptor and CD40
Jak1	0.709	Janus kinase 1		Essential tyrosine kinase involved signal transduction in type I and II cytokines and IFNs
Jak2	0.797	Janus kinase 2		Tyrosine kinase that participates in IFN and IL6ST signaling cascades
Pik3cd	1.19	Phosphatidylinositol-4,5-bisphosphate 3-kinase catalytic subunit delta isoform		A subunit of PI3K; acts downstream of TLR4, TCR, BCR, and CD40; contributes to T helper cell expansion, mast cell development, and neutrophil chemotaxis, extravasation, and respiratory burst
Pik3cg	1.21	Phosphatidylinositol-4,5-bisphosphate 3-kinase catalytic		A subunit of PI3K; modulates leukocyte chemotaxis to inflammatory sites and in response to chemoattractant agents

		subunit gamma isoform		
Prkcd	0.892	Protein kinase C delta		Ca(2+)-independent, phospholipid- and DAG-dependent serine/threonine-protein kinase that promotes apoptosis in response to DNA damage but inhibits it during cytokine receptor-initiated cell death; required for oxygen radical production by NADPH oxidase
Syk	1.95	Spleen-associated tyrosine kinase		Critical kinase that transmits signals from the TCR and BCR
Tbk1	1.05	TANK-binding kinase 1		Coordinates the activation of IRF3 and NFκB and induction of type I IFNs
Zap70	1.72	Zeta chain of T cell receptor associated protein kinase 70		Tyrosine kinase that plays an essential role in regulation of the adaptive immune response; regulates motility, adhesion, proliferation, differentiation, and cytokine expression in T cells; contributes to the development and activation of primary B cells; phosphorylates at least two essential adapter proteins: LAT and LCP2, leading in turn to a large number of signaling molecules being recruited
**Lysosomal Activity**				

Ctsh	1.25	Cathepsin H		Lysosomal protease; increased in macrophages in response to IFNγ
Ctss	2.53	Cathepsin S		Lysosomal protease that participates in processing of Ag by MHC class II
Hck	1.76	Hematopoietic cell kinase		Src family tyrosine kinase that mediates secretory lysosome mobilization, degranlation, and activation of NADPH oxidase
**Macrophage Function**				
Cd14	1.86	Cluster of differentiation 14		PRR that recognizes LPS; mostly found on macrophages
Csf1	0.698	Macrophage colony-stimulating factor 1		Cytokine that promote activation and survival of monocytes
Csf1r	1.11	Macrophage colony-stimulating factor 1 receptor	CD115	Receptor for CSF1; promotes release of inflammatory cytokines in response to IL-34 and CSF1
Cebpb	1.28	CCAAT/enhancer-binding protein beta		Critical macrophage TF that promotes expression of several acute-phase and inflammatory cytokine genes, including Il6
Clec5a	1.73	C-Type lectin domain family 5, member a	Myeloid DAP12-associating lectin-1	Critical macrophage receptor for dengue virus serotypes 1-4; positive regulator of osteoclastogenesis

Ctsh	1.25	Cathepsin H		Lysosomal protease; increased in macrophages in response to IFNγ
Cxcl24		C-X-C motif chemokine ligand 24		Macrophage-produced cytokine; function unknown
Gpr183	0.932	G protein-coupled receptor 183	EBV-induced G protein-coupled receptor 2	Lymphocyte GPCR that acts as a chemotactic receptor for B cells, T cells, splenic DCs, monocytes/macrophages, and astrocytes
Irak3	1.89	Interleukin-1 receptor-associated kinase 3		Adaptor protein that negatively regulates TLR signaling; predominantly expressed in monocytes and macrophages
Itgam	2.19	Integrin alpha M	CD11b	Pairs with CD18 to form Mac-1 aka complement receptor 3; mediates leukocyte activation, adhesion, chemotaxis, migration, phagocytosis, and cell-mediated cytotoxicity; serves as a macrophage marker
Marco	4.23	Macrophage receptor with collagenous structure		A PRR that recognizes LDL
Slamf7	2.32	Signaling lymphocytic		A super-activator of macrophages and a strong promoter of phagocytosis; binds to CD74

		activation molecule family member 7		
Slc11a1	2.2	Natural resistance-associated macrophage protein 1		Macrophage-specific metal ion transporter; uptakes divalent metal cations to neutralize ROSs
Tnf	3.73	Tumor necrosis factor	Cachectin	Inflammatory cytokine mainly produced by macrophages; binds to TNFRSF1A/TNFR1 and TNFRSF1B/TNFBR; capable of inducing cell death in certain tumor cell lines
**MAP Kinase Signaling**				
Mapk1	-0.398	Mitogen-activated protein kinase 1	Extracellular signal-regulated kinase 2 (ERK2)	Serine/threonine kinase that acts as an essential component of the MAP kinase signal transduction pathway
Mapk3	-0.347	Mitogen-activated protein kinase 3	Extracellular signal-regulated kinase 1 (ERK1)	Serine/threonine kinase that acts as an essential component of the MAP kinase signal transduction pathway
Mapk14	0.289	Mitogen-activated protein kinase 14		One of the four p38 MAPKs; key kinase in the cascades of cellular responses evoked by extracellular stimuli such as proinflammatory cytokines

Map2k1	0.266	Dual specificity mitogen-activated protein kinase kinase 1	MAPK/ERK kinase 1 (MEK1)	Essential component of the MAP kinase signal transduction pathway; participates in numerous biological functions, including cell growth, adhesion, survival, differentiation, transcription, metabolism, and cytoskeletal remodeling
Map4k2	0.666	Mitogen-activated protein kinase kinase kinase kinase 2		Essential component of the MAP kinase signal transduction pathway downstream of TRAF6; upstream activator of the SAP/JNK signaling pathway;
Mapkapk2	0.409	MAP kinase-activated protein kinase 2		Serine/threonine-protein kinase involved in cytokine production, endocytosis, reorganization of the cytoskeleton, cell migration, cell cycle control, chromatin remodeling, DNA damage response, and transcriptional regulation
**Mast Cell Function**				
Klrg1	1.08	Killer cell lectin-like receptor, subfamily G, member 1	C-type lectin domain family 15, member A (CLEC15A)	NK and T cell inhibitory receptor; binds to non-MHC ligands

			Mast cell function-associated antigen (MAFA)	
Lgals3	1.19	Galectin 3		Galactose-specific lectin that binds IgE; involved in acute inflammatory responses, including neutrophil activation and adhesion, chemoattraction of monocytes macrophages, opsonization of apoptotic neutrophils, and activation of mast cells
Pik3cd	1.19	Phosphatidylinositol-4,5-bisphosphate 3-kinase catalytic subunit delta isoform		A subunit of PI3K; acts downstream of TLR4, TCR, BCR, and CD40; contributes to T helper cell expansion, mast cell development, and neutrophil chemotaxis, extravasation, and respiratory burst
Pik3cg	1.21	Phosphatidylinositol-4,5-bisphosphate 3-kinase catalytic subunit gamma isoform		A subunit of PI3K; modulates leukocyte chemotaxis to inflammatory sites and in response to chemoattractant agents
**Metabolism**				
Abca1	2.26	ATP-binding cassette transporter A1		Membrane-associated cholesterol efflux pump

Abcg1	1.98	ATP-binding cassette transporter G1		Membrane-associated cholesterol efflux pump
Cd36	3.27	Cluster of differentiation 36	Fatty acid translocase (FAT)	Class B scavenger receptor that mediates fatty acid uptake
Map2k1	0.266	Dual specificity mitogen-activated protein kinase kinase 1	MAPK/ERK kinase 1 (MEK1)	Essential component of the MAP kinase signal transduction pathway; participates in numerous biological functions, including cell growth, adhesion, survival, differentiation, transcription, metabolism, and cytoskeletal remodeling
S100a8	2.3	S100 calcium-binding protein A8	Calgranulin A	Calcium- and zinc-binding protein involved in pro-inflammatory, antimicrobial, oxidant-scavenging and apoptosis-inducing activities; can induce neutrophil chemotaxis, adhesion, phagocytosis, and degranulation; predominantly found as calprotectin (S100A8/A9) which has a wide plethora of intra- and extracellular functions, including adhesion, apoptosis, autophagy, cytoskeletal remodeling, cytokine production, chemotaxis, migration, inflammation, arachidonic acid metabolism, oxidant-scavenging, and PRR signaling

Migration/Motility				
App	0.801	Amyloid-beta precursor protein		Cell surface receptor and transmembrane precursor protein that is cleaved by secretases to form a number of peptides; involved in cell mobility, copper homeostasis, and oxidative stress
Cd97	0.748	Cluster of differentiation 97	BL-Ac[F2]	GPCR that promotes granulocyte adhesion and migration; activates T cells via binding to CD55; stimulates angiogenesis through binding integrin counterreceptors on endothelial cells
Icam1	1.69	Intracellular adhesion molecule 1	CD54	Cell surface glycoprotein that serves as strong adhesive ligand for LFA-1; important for leukocyte mobility and costimulation
Itgam	2.19	Integrin alpha M	CD11b	Pairs with CD18 to form Mac-1 aka complement receptor 3; mediates leukocyte activation, adhesion, chemotaxis, migration, phagocytosis, and cell-mediated cytotoxicity; serves as a macrophage marker
Jam3	1.54	Junctional adhesion molecule C		Immunoglobulin that mediates tight junctions between endothelial cells; mediates transepithelial migration of PMNs; promotes

				chemotaxis of vascular endothelial cells and stimulates angiogenesis
S100a8	2.3	S100 calcium-binding protein A8	Calgranulin A	Calcium- and zinc-binding protein involved in pro-inflammatory, antimicrobial, oxidant-scavenging and apoptosis-inducing activities; can induce neutrophil chemotaxis, adhesion, phagocytosis, and degranulation; predominantly found as calprotectin (S100A8/A9) which has a wide plethora of intra- and extracellular functions, including adhesion, apoptosis, autophagy, cytoskeletal remodeling, cytokine production, chemotaxis, migration, inflammation, arachidonic acid metabolism, oxidant-scavenging, and PRR signaling
Tgfb1	0.803	Transforming growth factor beta 1		Multifunctional protein that regulates the growth and differentiation of various cell types and is involved in various processes, such as normal development, immune function, microglia function and responses to neurodegeneration; can induce EMT and cell migration in various cell types; frequently acts as an immunosuppressive cytokine in the TME

Vegfa	0.592	Vascular endothelial growth factor A		Glysosylated mitogen that promotes vascular permeability, vasculogenesis, angiogenesis, and cell migration
**Neutrophil Function**				
Fpr2	3.4	Formyl peptide receptor 2	Lipoxin A4 receptor	Low affinity receptor for N-formyl-methionyl peptides; activates neutrophils
Lcn2	1.97	Lipocalin 2	Neutrophil gelatinase-associated lipocalin (NGAL)	Neutrophil-secreted factor that sequesters iron-containing siderophores; also functions as a growth factor
Ncf4	1.34	Neutrophil cytosolic factor 4	SH3 and PX domain-containing protein 4 (SH3PXD4)	Cytosolic regulatory component of the superoxide-producing phagocyte NADPH-oxidase, a multicomponent enzyme system important for host defense
Pik3cd	1.19	Phosphatidylinositol-4,5-bisphosphate 3-kinase catalytic subunit delta isoform		A subunit of PI3K; acts downstream of TLR4, TCR, BCR, and CD40; contributes to T helper cell expansion, mast cell development, and neutrophil chemotaxis, extravasation, and respiratory burst
S100a8	2.3	S100 calcium-binding protein A8	Calgranulin A	Calcium- and zinc-binding protein involved in pro-inflammatory, antimicrobial, oxidant-scavenging and apoptosis-inducing activities; can induce neutrophil chemotaxis, adhesion,

				phagocytosis, and degranulation; predominantly found as calprotectin (S100A8/A9) which has a wide plethora of intra- and extracellular functions, including adhesion, apoptosis, autophagy, cytoskeletal remodeling, cytokine production, chemotaxis, migration, inflammation, arachidonic acid metabolism, oxidant-scavenging, and PRR signaling
		**NFκB Signaling**		
Bcl10	0.393	B cell lymphoma/leukemia 10		Activates NFκB via ubiquitination of IKKγ
Card11	1.15	Caspase recruitment domain family member 11	Bcl10-interacting MAGUK protein 3 (BIMP3)	Adapter protein that plays a key role in adaptive immune response by transducing the activation of NFκB downstream of TCR and BCR engagement; upon activation in response to TCR or BCR triggering, homooligomerizes to form a nucleating helical template that recruits BCL10 via CARD-CARD interaction, thereby promoting polymerization of BCL10 and subsequent recruitment of MALT1, leading to IKK phosphorylation and

				degradation and release of NFκB proteins for nuclear translocation
Cxcr3	2.23	C-X-C motif chemokine receptor 3	CD183	Induces integrin activation, cytoskeletal remodeling, and chemotaxis; expressed by T cells and NK cells; prominently expressed in effector and memory T cells
Ikbkb	0.362	Inhibitor of nuclear factor kappa B kinase subunit beta		Part of the IKK complex that inhibits IκBα and permits NFκB nuclear localization
Ikbke	1.36	Inhibitor of nuclear factor kappa B kinase subunit epsilon		Noncanonical IκB kinase; plays an essential role in regulating inflammatory through the activation of the type I IFN, NFκB, and STAT signaling
Nfkb1	0.349	Nuclear factor kappa B subunit 1	p105/p50	One of the NFκB family TFs; inhibits inflammation
Rel	1.47	Avian reticuloendotheliosis viral oncogene homolog	c-Rel	One of the NFκB family TFs; important for B cell and Treg development
Relb	0.86	Avian reticuloendotheliosis		One of the NFκB family TFs; controls lymphoid development, DC biology, and noncanonical NFκB signaling

		viral oncogene homolog B		
**NK Cell Function**				
Cd8a	2.02	Cluster of differentiation 8 alpha	LEU2	Alpha chain of the CD8 coreceptor, which binds to MHC class I; in T cells, functions primarily as a coreceptor for MHC class I molecule:peptide complex; in NK cells, the presence of CD8α homodimers at the cell surface provides a survival mechanism allowing conjugation and lysis of multiple target cells
Cd69	1.46	Cluster of differentiation 69	C-type lectin domain family 2, member C	Signal transmitting receptor in lymphocytes, NK cells, and platelets; induced upon T cell activation; involved in lymphocyte proliferation
Gzmb	3.8	Granzyme B	Fragmentin 2	Abundant protease in the cytosolic granules of cytotoxic T and NK cells that activates caspase-mediated cell death when delivered into the target cell through the immunological synapse
Gzmk	3.36	Granzyme K	Tryptase II	Granule-secreted, pro-apoptotic serine protease found in the cytoplasmic granules of CTLs

Klrg1	1.08	Killer cell lectin-like receptor, subfamily G, member 1	C-type lectin domain family 15, member A (CLEC15A)<br><br>Mast cell function-associated antigen (MAFA)	NK and T cell inhibitory receptor; binds to non-MHC ligands
Pik3cd	1.19	Phosphatidylinositol-4,5-bisphosphate 3-kinase catalytic subunit delta isoform		A subunit of PI3K; acts downstream of TLR4, TCR, BCR, and CD40; contributes to T helper cell expansion, mast cell development, and neutrophil chemotaxis, extravasation, and respiratory burst
Pik3cg	1.21	Phosphatidylinositol-4,5-bisphosphate 3-kinase catalytic subunit gamma isoform		A subunit of PI3K; modulates leukocyte chemotaxis to inflammatory sites and in response to chemoattractant agents
Prdm1	1.77	Positive regulatory domain I-binding factor	B lymphocyte-induced maturation protein (BLIMP1)	TF that plays a role in the development, retention, and long-term establishment of T cell, NK cell, and NK-T cells in non-lymphoid organs; drives the maturation of B cell into Ig secreting cells

Tcf7	1.32	Transcription factor 7		HMG box TF predominantly expressed by T cells that drives their development, although also involved in NK cell development; activates transcription through a Wnt/β-catenin signaling pathway
**Pattern Recognition Receptors**				
Cd14	1.86	Cluster of differentiation 14		PRR that recognizes LPS; mostly found on macrophages
Cd180	2	Cluster of differentiation 180		Heterodimeric binding partner of Ly86 that participates in LPS binding in APCs
Clec4a2	1.75	C-type lectin domain family 4 member A2		PRR that, upon binding mannose or fucose, is endocytosed and processed in the Ag presentation pathway
Clec4n	1.82	C-type lectin domain family 4, member N	Dectin-2	PRR specific for Mycobacterial mannose-capped lipoarabinomannan
Clec5a	1.73	C-Type lectin domain family 5, member a	Myeloid DAP12-associating lectin-1	Critical macrophage receptor for dengue virus serotypes 1-4; positive regulator of osteoclastogenesis
Clec7a	2.64	C-Type lectin domain family 7, member a	Dectin-1	PRR specific for β-1,3- and β-1,6-linked glucans from fungi and plants; necessary for the TLR2-mediated inflammatory response and for TLR2-mediated activation of NF-κB

Ddx58	0.921	DExD/H-box helicase 58	Retinoic acid-inducible gene I (RIG-I)	Cytoplasmic PRR that recognizes dsRNA; can promote T cell-independent B cell activation; uses MAVS as an adaptor
Fpr2	3.4	Formyl peptide receptor 2	Lipoxin A4 receptor	Low affinity receptor for N-formyl-methionyl peptides; activates neutrophils
Ifih1	1.43	Interferon induced with helicase C domain 1	Helicard; melanoma differentiation-associated protein 5 (MDA5)	PRR for cytoplasmic dsRNA; upon target recognition, associates with MAVS to activate TNK1 and IKBKE, which phosphorylate IRF3 and -7, which, in turn, activate transcription of IFNα and -β
Ly86	0.948	Lymphocyte antigen 86	Myeloid differentiation factor 1 (MD-1)	Heterodimeric binding partner of CD180 that participates in LPS binding in APCs
Ly96	0.606	Lymphocyte antigen 96	Myeloid differentiation factor 2 (MD-2)	Heterodimeric binding partner of TLR4 that participates in LPS binding
Marco	4.23	Macrophage receptor with collagenous structure		A PRR that recognizes LDL

Myd88	0.978	Myeloid differentiation primary response 88		Key adaptor in the TLR signaling pathways; interacts with all TLRs except TLR3; activates NFκB and IRFs
Nlrp3	1.75	NACHT domain-, leucine-rich repeat-, and PYD-containing protein 3	Cryopyrin	PRR with a wide diversity of recognized targets that activates the NLRP3 inflammasome consisting of NLRP3, PYCARD, and caspase-1/-8
Nod1	1.02	Nucleotide binding oligomerization domain containing 1		Intracellular PRR that recognizes peptidoglycan-derived muropeptides and Shigella effector proteins
Nod2	1.7	Nucleotide-binding oligomerization domain containing 2		PRR specific for muramyl dipeptide (MDP); upon binding to its ligand, recruits RIPK2 and triggers MAPK and NFκB signaling
S100a8	2.3	S100 calcium-binding protein A8	Calgranulin A	Calcium- and zinc-binding protein involved in pro-inflammatory, antimicrobial, oxidant-scavenging and apoptosis-inducing activities; can induce neutrophil chemotaxis, adhesion, phagocytosis, and degranulation; predominantly found as calprotectin (S100A8/A9) which has a wide plethora of intra- and extracellular functions, including adhesion, apoptosis, autophagy, cytoskeletal remodeling, cytokine production, chemotaxis, migration, inflammation, arachidonic acid

				metabolism, oxidant-scavenging, and PRR signaling
Ticam2	1.79	TIR domain-containing adaptor molecule 2		Sorting adapter in various innate immune signaling cascades; bridges TLR2 and MyD88
Tlr2	1.49	Toll-like receptor 2	CD282	Surface PRR that binds to various lipid-containing PAMPs
Tlr4	1.08	Toll-like receptor 4	CD284	Surface PRR that recognizes LPS; pairs with LY96 and CD14; acts via MYD88, TIRAP, and TRAF6, leading to NFκB activation, cytokine secretion, and the inflammatory response; in complex with TLR6, promotes sterile inflammation in monocytes/macrophages in response to oxLDL or amyloid-β 42
Tlr6	2.37	Toll like receptor 6	CD286	Surface PRR that recognizes diacylated and triacylated lipopeptides
Tlr7	2.19	Toll-like receptor 7	CD287	Endosomic PRR that recognizes ssRNA
Tlr8	1.65	Toll-like receptor 8	CD288	Endosomic PRR that recognizes ssRNA
Tlr9	1.65	Toll like receptor 9	CD289	Endosomic PRR that recognizes unmethylated CpG dinucleotides
**Phagocytosis**				

Fcgr2b	1.41	Fragment crystallizable of immunoglobulin gamma receptor IIb	CD32	Low affinity receptor for the Fc region of complexed or aggregated γ-Igs; involved in a variety of effector and regulatory functions such as phagocytosis of immune complexes and modulation of Ab production by B cells; essential for the maintenance of humoral tolerance; acts as a late checkpoint at the level of class-switched memory B cells, plasmablasts or plasma cells; regulates plasma-cell homeostasis and survival
Itgam	2.19	Integrin alpha M	CD11b	Pairs with CD18 to form Mac-1 aka complement receptor 3; mediates leukocyte activation, adhesion, chemotaxis, migration, phagocytosis, and cell-mediated cytotoxicity; serves as a macrophage marker
Ncf4	1.34	Neutrophil cytosolic factor 4	SH3 and PX domain-containing protein 4 (SH3PXD4)	Cytosolic regulatory component of the superoxide-producing phagocyte NADPH-oxidase, a multicomponent enzyme system important for host defense
S100a8	2.3	S100 calcium-binding protein A8	Calgranulin A	Calcium- and zinc-binding protein involved in pro-inflammatory, antimicrobial, oxidant-scavenging and apoptosis-inducing activities; can induce neutrophil chemotaxis, adhesion, phagocytosis, and degranulation;

				predominantly found as calprotectin (S100A8/A9) which has a wide plethora of intra- and extracellular functions, including adhesion, apoptosis, autophagy, cytoskeletal remodeling, cytokine production, chemotaxis, migration, inflammation, arachidonic acid metabolism, oxidant-scavenging, and PRR signaling
Slamf7	2.32	Signaling lymphocytic activation molecule family member 7		A super-activator of macrophages and a strong promoter of phagocytosis; binds to CD74
**ROS Generation & Protection**				
Cybb	2.37	Cytochrome b-245 heavy chain	Nox2	Part of the NADPH oxidase process; generates superoxides
Ncf4	1.34	Neutrophil cytosolic factor 4	SH3 and PX domain-containing protein 4 (SH3PXD4)	Cytosolic regulatory component of the superoxide-producing phagocyte NADPH-oxidase, a multicomponent enzyme system important for host defense
Nos2	4.12	Inducible nitric oxide synthase (iNOS)		Produces reactive oxygen species and contributes to inflammatory cytokine production

Prkcd	0.892	Protein kinase C delta		Ca(2+)-independent, phospholipid- and DAG-dependent serine/threonine-protein kinase that promotes apoptosis in response to DNA damage but inhibits it during cytokine receptor-initiated cell death; required for oxygen radical production by NADPH oxidase
**Stress Response**				
Aft2		Activator of Fe+ transcription 2		Iron-regulated transcriptional activator; activates genes involved in intracellular iron use; required for iron homeostasis and oxidative stress resistance
App		Amyloid-beta precursor protein		Cell surface receptor and transmembrane precursor protein that is cleaved by secretases to form a number of peptides; involved in cell mobility, copper homeostasis, and oxidative stress
**T Cell Function**				
Cd2	1.68	Cluster of differentiation 2	Leukocyte functional antigen 2 (LFA-2)	Interacts with LFA-3 and CD48 to mediate adhesion between T cells and other cell types

Cd3d	2.3	Cluster of differentiation 3 delta		Component of the TCR-CD3 complex; upon phosphorylation by Lck, serves as a docking station for downstream TCR signaling adaptors
Cd3e	2.27	Cluster of differentiation 3 epsilon		Component of the TCR-CD3 complex; initiates the TCR-CD3 complex assembly by forming the two heterodimers CD3δ/CD3ε and CD3γ/CD3ε; also participates in internalization and cell surface down-regulation of TCR-CD3 complexes via endocytosis sequences present in CD3ε cytosolic region
Cd3g	2.01	Cluster of differentiation 3 gamma		Component of the TCR-CD3 complex; plays an essential role in the dynamic regulation of TCR expression at the cell surface
Cd4	1.68	Cluster of differentiation 4		Signature helper T cell marker; binds to MHC class II and provides necessary costimulation for T cell activation
Cd5	2.56	Cluster of differentiation 5	LEU1	Type-I transmembrane glycoprotein found on the surface of T and B cells; may act as a receptor in regulating T cell proliferation
Cd8a	2.02	Cluster of differentiation 8 alpha	LEU2	Alpha chain of the CD8 coreceptor, which binds to MHC class I; in T cells, functions primarily as a coreceptor for MHC class I molecule:peptide complex; in NK cells, the

				presence of CD8α homodimers at the cell surface provides a survival mechanism allowing conjugation and lysis of multiple target cells
Cd8b1	2.14	Cluster of differentiation 8 beta 1		Beta chain of the CD8 coreceptor, which binds to MHC class I
Cd28	2.48	Cluster of differentiation 28		Essential T cell co-receptor that enhances T cell activation, proliferation, cytokine production, and survival; binds to CD80 and CD86
Cd69	1.46	Cluster of differentiation 69	C-type lectin domain family 2, member C	Signal transmitting receptor in lymphocytes, NK cells, and platelets; induced upon T cell activation; involved in lymphocyte proliferation
Cd37	2.07	Cluster of differentiation 37	Tetraspanin-26	Cell surface glycoprotein known to complex with integrins and other transmembrane 4 superfamily proteins; may play a role in T cell-B cell interactions
Cd97	0.748	Cluster of differentiation 97	BL-Ac[F2]	GPCR that promotes granulocyte adhesion and migration; activates T cells via binding to CD55; stimulates angiogenesis through

				binding integrin counterreceptors on endothelial cells
Cd247	2.49	Cluster of differentiation 247	T cell surface glycoprotein CD3 zeta chain	Central intracellular signaling chain of the TCR, to which downstream signaling adaptors dock
Cxcr3	2.23	C-X-C motif chemokine receptor 3	CD183	Induces integrin activation, cytoskeletal remodeling, and chemotaxis; expressed by T cells and NK cells; prominently expressed in effector and memory T cells
Cyfip2	1.74	Cytoplasmic FMR1-interacting protein 2		Involved in T cell adhesion and p53/TP53-dependent induction of apoptosis
Gata3	1.2	GATA binding protein 3		Transcriptional activator that binds to the enhancer of the TCR α and δ genes; required for T_H2 differentiation following immune and inflammatory responses
Gpr183	0.932	G protein-coupled receptor 183	EBV-induced G protein-coupled receptor 2	Lymphocyte GPCR that acts as a chemotactic receptor for B cells, T cells, splenic DCs, monocytes/macrophages, and astrocytes
Gzmb	3.8	Granzyme B	Fragmentin 2	Abundant protease in the cytosolic granules of cytotoxic T and NK cells that activates caspase-mediated cell death when delivered into the target cell through the immunological synapse

Gzmk	3.36	Granzyme K	Tryptase II	Granule-secreted, pro-apoptotic serine protease found in the cytoplasmic granules of CTLs
Eomes	1.04	Eomesodermin	T-box brain protein 2 (TBR2)	Transcriptional activator critical for development; involved in $CD8^{+}$ T cell differentiation
Icos	2.45	Inducible T cell costimulator	CD278	Enhances all basic T cell responses to foreign Ag; essential both for efficient interaction between T and B cells and for normal Ab responses to T cell-dependent Ags
Ikzf1	1.19	IKAROS family zinc finger 1		Transcriptional regulator of hematopoietic cell differentiation; plays a role in T and B cell development
Il12rb1	1.07	Interleukin 12 receptor subunit beta 1	CD212	Cytokine receptor component that associates with IL12RB2 to IL23R
Itk	2.06	Interleukin-2-inducible T cell kinase	LYK	Key actor in the TCR signaling cascade; phosphorylates PLCγ1, LAT, and LCP2
Lck	1.98	Lymphocyte cell kinase		Src family tyrosine kinase that acts as one of the main signaling intermediaries downstream

				of the TCR; constitutively associated with the cytoplasmic portion of CD4
Lcp1	1.62	Lymphocyte cytosolic protein 1	Plastin-2	Actin-binding protein that promotes T cell activation in response to costimulation through TCR/CD3 and CD2 or CD28; assists with IL2RA transport to the cell surface
Nfatc2	0.971	Nuclear factor of activated T cells, cytoplasmic 2		Cytosolic component of the NFAT TF complex; mediates induction of IL-2, IL-3, IL-4, TNFα, and GM-CSF
Pik3cd	1.19	Phosphatidylinositol-4,5-bisphosphate 3-kinase catalytic subunit delta isoform		A subunit of PI3K; acts downstream of TLR4, TCR, BCR, and CD40; contributes to T helper cell expansion, mast cell development, and neutrophil chemotaxis, extravasation, and respiratory burst
Pik3cg	1.21	Phosphatidylinositol-4,5-bisphosphate 3-kinase catalytic subunit gamma isoform		A subunit of PI3K; modulates leukocyte chemotaxis to inflammatory sites and in response to chemoattractant agents
Prdm1	1.77	Positive regulatory domain I-binding factor	B lymphocyte-induced maturation protein (BLIMP1)	TF that plays a role in the development, retention, and long-term establishment of T cell, NK cell, and NK-T cells in non-lymphoid

				organs; drives the maturation of B cell into Ig secreting cells
Ptprc	1.5	Protein tyrosine phosphatase receptor type C	CD45; leukocyte common antigen (LCA)	Delivers costimulation during T cell activation upon binding to its ligand DPP4; dephosphorylates Lyn and suppresses JAK kinases
Rora	1.69	Retinoic acid receptor-related orphan receptor A		Nuclear receptor that binds hormone response elements upstream of several genes to enhance the expression of those genes; plays an essential role in the development of type 2 ILCs
Spn	1.63	Sialophorin	Leukosialin; CD43	Cell surface sialoglycoprotein expressed by T cells, B cells, monocytes, and granulocytes; promotes lymph node localization in T cells; shunts T cells away from the T_H2 phenotype and towards T_H1; promotes the expression of IFNγ in $CD4^+$ T cells
Syk	1.95	Spleen-associated tyrosine kinase		Critical kinase that transmits signals from the TCR and BCR
Tbx21	1.5	T-box transcription factor 21		Initiates T_H1 lineage development from naïve T_H precursor cells both by activating T_H1 genetic programs and by repressing the opposing T_H2 and T_H17 genetic programs

Tcf7	1.32	Transcription factor 7		HMG box TF predominantly expressed by T cells that drives their development, although also involved in NK cell development; activates transcription through a Wnt/β-catenin signaling pathway
Zap70	1.72	Zeta chain of T cell receptor associated protein kinase 70		Tyrosine kinase that plays an essential role in regulation of the adaptive immune response; regulates motility, adhesion, proliferation, differentiation, and cytokine expression in T cells; contributes to the development and activation of primary B cells; phosphorylates at least two essential adapter proteins: LAT and LCP2, leading in turn to a large number of signaling molecules being recruited
**Transcription Factors & Coactivators**				
Cebpb	1.28	CCAAT/enhancer-binding protein beta		Critical macrophage TF that promotes expression of several acute-phase and inflammatory cytokine genes, including Il6
Eomes	1.04	Eomesodermin	T-box brain protein 2 (TBR2)	Transcriptional activator critical for development; involved in $CD8^+$ T cell differentiation

Foxp3	1.56	Forkhead box P3	DIETER	Master TF for Tregs; represses expression of Il2 and Ifng; activates expression of Tnfrsf18, Il2ra, and Ctla4
Gata3	1.2	GATA binding protein 3		Transcriptional activator that binds to the enhancer of the TCR α and δ genes; required for T_H2 differentiation following immune and inflammatory responses
Irf1	1.71	Interferon regulatory factor 1		Transcriptional regulator that promotes inflammatory innate and adaptive immune responses
Irf4	1.2	Interferon regulatory factor 4		Transcriptional activator that complexes with BATF and binds ISREs within the promoters of multiple genes involved in inflammation
Irf7	2.22	Interferon regulatory factor 7		Key transcriptional regulator of type I IFN-dependent immune responses; promotes transcription of IFNα and -β
Irf8	1.12	Interferon regulatory factor 8		TF that regulates of lineage commitment in myeloid cell maturation; promotes monocyte and plasmacytoid DC development
Ikzf1	1.19	IKAROS family zinc finger 1		Transcriptional regulator of hematopoietic cell differentiation; plays a role in T and B cell development

Mef2c	1.24	Myocyte enhancer factor 2c		Transcriptional activator that binds specifically to the MEF2 element present in the regulatory regions of many muscle-specific genes; controls cardiac morphogenesis and myogenesis, and is also involved in vascular development; required for B cell survival and proliferation in response to BCR stimulation, efficient IgG1 Ab responses to T cell-dependent Ags, and for normal induction of GC B cells
Nfatc2	0.971	Nuclear factor of activated T cells, cytoplasmic 2		Cytosolic component of the NFAT TF complex; mediates induction of IL-2, IL-3, IL-4, TNFα, and GM-CSF
Nfkb1	0.349	Nuclear factor kappa B subunit 1	p105/p50	One of the NFκB family TFs; inhibits inflammation
Pou2f2	1.81	POU domain class 2, transcription factor 2		TF that regulates Ab and IL-6 expression in B cells
Prdm1	1.77	Positive regulatory domain I-binding factor	B lymphocyte-induced maturation protein (BLIMP1)	TF that plays a role in the development, retention, and long-term establishment of T cell, NK cell, and NK-T cells in non-lymphoid organs; drives the maturation of B cell into Ig secreting cells

Stat1	1.53	Signal transducer and activator of transcription 1		Transcriptional activator that mediates cellular responses to IFNs, cytokines, and other growth factors
Stat3	0.649	Signal transducer and activator of transcription 3		Transcriptional activator of genes involved in cell growth and apoptosis; activated by JAKs
Stat4	1.25	Signal transducer and activator of transcription 4		Essential TF for T_H1 $CD4^+$ T cell development and IFNγ production; also promotes expression of MyD88
Stat6	0.571	Signal transducer and activator of transcription 6		Essential TF for T_H2 $CD4^+$ T cell and macrophage function and M2 macrophage polarization
Tbx21	1.5	T-box transcription factor 21		Initiates T_H1 lineage development from naïve T_H precursor cells both by activating T_H1 genetic programs and by repressing the opposing T_H2 and T_H17 genetic programs
Tcf7	1.32	Transcription factor 7		HMG box TF predominantly expressed by T cells that drives their development, although also involved in NK cell development; activates transcription through a Wnt/β-catenin signaling pathway

Zbp1	1.86	Z-DNA binding protein 1	Tumor stroma and activated macrophage protein DLM-1	Key innate sensor that recognizes and binds Z-RNA structures, which are produced by a number of viruses, and induces type-I IFN production; key activator of cellular necroptosis; promotes IL-1α induction in an NLRP3-inflammasome-independent manner
**Ubiquitin Regulation**				
Bcl10	0.393	B cell lymphoma/leukemia 10		Activates NFκB via ubiquitination of IKKγ
Cyld	0.595	Cylindromatosis lysine 63 deubiquitinase		Inhibits NFκB activation by deubiquitinating upstream signaling factors; inhibits Wnt signaling; restricts polyubiquitination of RIPK1 and -2, thereby limiting necroptosis
Tnfaip3	1.34	Tumor necrosis factor, alpha-induced protein 3		Ubiquitin-editing enzyme that complexes with ITCH to degrade inflammatory signaling components in the TNF, IL1, and TLR pathways; targets TRAF2, TRAF6, and IKK
Ubc	0.653	Polyubiquitin C		Serves various roles, including innate immunity, DNA repair, and stimulation of autophagy and the proteasomal response

NBTXR3+PBT+αPD1 vs PBT+αPD1				
**Gene**	**Log2 fold change**	**Full Name**	**Notable Aliases**	**Function**
**Adhesion & Cell-Cell Interactions**				
Cd2	1.66	Cluster of differentiation 2	T cell surface antigen CD2	Interacts with LFA-3 and CD48 to mediate adhesion between T cells and other cell types
Icam1	0.688	Intracellular adhesion molecule 1	CD54	Cell surface glycoprotein that serves as strong adhesive ligand for LFA-1; important for leukocyte mobility and costimulation
Itgal	0.959	Integrin alpha L		Pairs with ITGB2 to form LFA-1, a common leukocyte adhesion molecule and costimulatory receptor
**Antigen Processing & Presentation**				
H2-K1	0.439	Histocompatibility 2, K1, K region		MHC class I molecule; presents Ags to $CD8^+$ T cells
**Anti-Inflammatory**				
Ctla4	0.969	Cytotoxic T lymphocyte antigen 4	CD152	Inhibitory receptor that blocks CD28 costimulation by competitively binding its ligands CD80 and CD86
Stat6	0.392	Signal transducer and activator of		Essential TF for T_H2 $CD4^+$ T cell and macrophage function and M2 macrophage

		transcription 6		polarization
		**Autophagy**		
Ubc	0.39	Polyubiquitin C		Serves various roles, including innate immunity, DNA repair, and stimulation of autophagy and the proteasomal response
		**B Cell-associated Genes**		
Gpr183	1.14	G protein-coupled receptor 183	EBV-induced G protein-coupled receptor 2	Lymphocyte GPCR that acts as a chemotactic receptor for B cells, T cells, splenic DCs, monocytes/macrophages, and astrocytes
Prdm1	1.22	Positive regulatory domain I-binding factor	B lymphocyte-induced maturation protein (BLIMP1)	TF that plays a role in the development, retention, and long-term establishment of T cell, NK cell, and NK-T cells in non-lymphoid organs; drives the maturation of B cell into Ig secreting cells
Spn	0.91	Sialophorin	Leukosialin; CD43	Cell surface sialoglycoprotein expressed by T cells, B cells, monocytes, and granulocytes; promotes lymph node localization in T cells; shunts T cells away from the T_H2 phenotype and towards T_H1; promotes the expression of IFNγ in $CD4^+$ T cells
Syk	0.978	Spleen-associated tyrosine kinase		Critical kinase that transmits signals from the TCR and BCR

**Chemotaxis**				
Ccr9	0.918	C-C motif chemokine receptor 9		Receptor for CCL25; increases intracellular Ca(2+) levels upon ligand binding
Cxcr3	1.38	C-X-C motif chemokine receptor 3	CD183	Induces integrin activation, cytoskeletal remodeling, and chemotaxis; expressed by T cells and NK cells; prominently expressed in effector and memory T cells
Gpr183	1.14	G protein-coupled receptor 183	EBV-induced G protein-coupled receptor 2	Lymphocyte GPCR that acts as a chemotactic receptor for B cells, T cells, splenic DCs, monocytes/macrophages, and astrocytes
**Complement & Humoral Immunity**				
C2	0.885	Complement component 2		Serine protease that binds to C4b to form the C4bC2 complex
Cd55	1.31	Cluster of differentiation 55	Complement decay-accelerating factor	Cell surface glycoprotein that interacts with surface-bound C4b and inhibits its conversion of C2 to C2b
Cfp	0.922	Complement factor properdin		Alternate complement pathway component; when cleaved, produces a serine protease that binds to C3b to form C3 convertase
Fcer1a	1.18	Fragment crystallizable of immunoglobulin		High affinity receptor for IgE; responsible for initiating the allergic response

		epsilon receptor 1a		
		**Costimulation**		
Cd28	0.848	Cluster of differentiation 28		Essential T cell co-receptor that enhances T cell activation, proliferation, cytokine production, and survival; binds to CD80 and CD86
Icam1	0.688	Intracellular adhesion molecule 1	CD54	Cell surface glycoprotein that serves as strong adhesive ligand for LFA-1; important for leukocyte mobility and costimulation
Itgal	0.959	Integrin alpha L		Pairs with ITGB2 to form lymphocyte function-associated antigen-1 (LFA-1), a common leukocyte adhesion molecule and costimulatory receptor
Tnfrsf11a	1.18	Tumor necrosis factor receptor superfamily member 11A	Receptor activator of NFκB (RANK)	Recruits TRAFs and activates NFκB and JNK activation upon binding to RANKL on the surface of T cells
Tnfrsf4	1.21	Tumor necrosis factor receptor superfamily member 4	OX40; CD134	Receptor for TNFSF4/OX40L/GP34; costimulatory molecule implicated in long-term T cell immunity; activates NFκB through its interaction with adaptor proteins TRAF2 and TRAF5; suppresses apoptosis through

				upregulation of BCL2
**Cytokines**				
Il1rl1	1.22	Interleukin 1 receptor-like 1		Receptor for IL-33; recruits MyD88, IRAK1, IRAK4, and TRAF6; activates ERK1, ERK2, and MAPK14
Il18r1	1.09	Interleukin 18 receptor 1	CD218a	Receptor for IL-18
**Hematopoiesis**				
Ikzf2	1.06	IKAROS family zinc finger protein 2		Hematopoietic cell-specific TF involved in early hematopoietic development
**Inflammation**				
Irf7	0.513	Interferon regulatory factor 7		Key transcriptional regulator of type I IFN-dependent immune responses; promotes transcription of IFNα and -β
Jak2	0.473	Janus kinase 2		Tyrosine kinase that participates in IFN and IL6ST signaling cascades
Nod2	0.978	Nucleotide-binding oligomerization domain containing 2		PRR specific for muramyl dipeptide (MDP); upon binding to its ligand, recruits RIPK2 and triggers MAPK and NFκB signaling
Spn	0.91	Sialophorin	Leukosialin; CD43	Cell surface sialoglycoprotein expressed by T cells, B cells, monocytes, and granulocytes;

				promotes lymph node localization in T cells; shunts T cells away from the T_H2 phenotype and towards T_H1; promotes the expression of IFNγ in $CD4^+$ T cells
**Inhibition**				
Ctla4	0.969	Cytotoxic T lymphocyte antigen 4	CD152	Inhibitory receptor that blocks CD28 costimulation by competitively binding its ligands CD80 and CD86
**Interferon Response**				
Irf7	0.513	Interferon regulatory factor 7		Key transcriptional regulator of type I IFN-dependent immune responses; promotes transcription of IFNα and -β
Jak2	0.473	Janus kinase 2		Tyrosine kinase that participates in IFN and IL6ST signaling cascades
**Kinases**				
Itk	1.35	Interleukin-2-inducible T cell kinase	LYK	Key actor in the TCR signaling cascade; phosphorylates PLCγ1, LAT, and LCP2
Jak2	0.473	Janus kinase 2		Tyrosine kinase that participates in IFN and IL6ST signaling cascades
Syk	0.978	Spleen-associated		Critical kinase that transmits signals from the

		tyrosine kinase		TCR and BCR
		**NFκB Signaling**		
Bcl10	0.371	B cell lymphoma/leukemia 10		Activates NFκB via ubiquitination of IKKγ
Card11	1.22	Caspase recruitment domain family member 11	Bcl10-interacting MAGUK protein 3 (BIMP3)	Adapter protein that plays a key role in adaptive immune response by transducing the activation of NFκB downstream of TCR and BCR engagement; upon activation in response to TCR or BCR triggering, homooligomerizes to form a nucleating helical template that recruits BCL10 via CARD-CARD interaction, thereby promoting polymerization of BCL10 and subsequent recruitment of MALT1, leading to IKK phosphorylation and degradation and release of NFκB proteins for nuclear translocation
		**NK Cell Function**		
Prdm1	1.22	Positive regulatory domain I-binding factor	B lymphocyte-induced maturation protein (BLIMP1)	TF that plays a role in the development, retention, and long-term establishment of T cell, NK cell, and NK-T cells in non-lymphoid organs; drives the maturation of B cell into Ig secreting cells

Tcf7	1.19	Transcription factor 7		HMG box TF predominantly expressed by T cells that drives their development, although also involved in NK cell development; activates transcription through a Wnt/β-catenin signaling pathway
**T Cell Function**				
Cd2	1.66	Cluster of differentiation 2	T cell surface antigen CD2	Interacts with LFA-3 and CD48 to mediate adhesion between T cells and other cell types
Cd3e	0.669	Cluster of differentiation 3 epsilon		Component of the TCR-CD3 complex; initiates the TCR-CD3 complex assembly by forming the two heterodimers CD3δ/CD3ε and CD3γ/CD3ε; also participates in internalization and cell surface down-regulation of TCR-CD3 complexes via endocytosis sequences present in CD3ε cytosolic region
Cd4	1.59	Cluster of differentiation 4		Signature helper T cell marker; binds to MHC class II and provides necessary costimulation for T cell activation
Cd8b1	0.856	Cluster of differentiation 8 beta 1		Beta chain of the CD8 coreceptor, which binds to MHC class I
Cd28	0.848	Cluster of		Essential T cell co-receptor that enhances T cell activation, proliferation, cytokine

		differentiation 28		production, and survival; binds to CD80 and CD86
Gata3	1.08	GATA binding protein 3		Transcriptional activator that binds to the enhancer of the TCR α and δ genes; required for T_H2 differentiation following immune and inflammatory responses
Gpr183	1.14	G protein-coupled receptor 183	EBV-induced G protein-coupled receptor 2	Lymphocyte GPCR that acts as a chemotactic receptor for B cells, T cells, splenic DCs, monocytes/macrophages, and astrocytes
Itk	1.35	Interleukin-2-inducible T cell kinase	LYK	Key actor in the TCR signaling cascade; phosphorylates PLCγ1, LAT, and LCP2
Prdm1	1.22	Positive regulatory domain I-binding factor	B lymphocyte-induced maturation protein (BLIMP1)	TF that plays a role in the development, retention, and long-term establishment of T cell, NK cell, and NK-T cells in non-lymphoid organs; drives the maturation of B cell into Ig secreting cells
Spn	0.91	Sialophorin	Leukosialin; CD43	Cell surface sialoglycoprotein expressed by T cells, B cells, monocytes, and granulocytes; promotes lymph node localization in T cells; shunts T cells away from the T_H2 phenotype and towards T_H1; promotes the expression of IFNγ in $CD4^+$ T cells

Syk	0.978	Spleen-associated tyrosine kinase		Critical kinase that transmits signals from the TCR and BCR
Tcf7	1.19	Transcription factor 7		HMG box TF predominantly expressed by T cells that drives their development, although also involved in NK cell development; activates transcription through a Wnt/β-catenin signaling pathway
**Transcription Factors & Coactivators**				
Gata3	1.08	GATA binding protein 3		Transcriptional activator that binds to the enhancer of the TCR α and δ genes; required for T_H2 differentiation following immune and inflammatory responses
Ikzf2	1.06	IKAROS family zinc finger protein 2		Hematopoietic cell-specific TF involved in early hematopoietic development
Irf7	0.513	Interferon regulatory factor 7		Key transcriptional regulator of type I IFN-dependent immune responses; promotes transcription of IFNα and -β
Prdm1	1.22	Positive regulatory domain I-binding factor	B lymphocyte-induced maturation protein (BLIMP1)	TF that plays a role in the development, retention, and long-term establishment of T cell, NK cell, and NK-T cells in non-lymphoid organs; drives the maturation of B cell into Ig secreting cells

Stat6	0.392	Signal transducer and activator of transcription 6		Essential TF for T_H2 $CD4^+$ T cell and macrophage function and M2 macrophage polarization
Tcf7	1.19	Transcription factor 7		HMG box TF predominantly expressed by T cells that drives their development, although also involved in NK cell development; activates transcription through a Wnt/β-catenin signaling pathway
**Ubiquitin Regulation**				
Ubc	0.39	Polyubiquitin C		Serves various roles, including innate immunity, DNA repair, and stimulation of autophagy and the proteasomal response

Supplemental Table 2: Genes Differentially Upregulated in Secondary (Unirradiated) Tumor

**PBT+αPD1 vs Ctrl**				
**Gene**	**Log2 fold change**	**Full Name**	**Notable Aliases**	**Function**
**Acute Phase Response**				
App	0.196	Amyloid-beta precursor protein		Cell surface receptor and transmembrane precursor protein that is cleaved by secretases to form a number of peptides;

				involved in cell mobility, copper homeostasis, and oxidative stress
Psen1	0.258	Presenilin-1		Catalytic subunit of the gamma-secretase complex, which cleaves integral membrane proteins such as APP; also involved in Ca(2+) homeostasis
**Adhesion & Cell-Cell Interactions**				
Cd47	0.269	Cluster of differentiation 47	Integrin-associated protein (IAP)	Partners with membrane integrins to serve as an inhibitor of phagocytosis
Itgax	0.521	Integrin alpha X	CD11c	Adhesion molecule; signature marker of Ag-presenting DCs
Icam1	0.473	Intracellular adhesion molecule 1	CD54	Cell surface glycoprotein that serves as strong adhesive ligand for LFA-1; important for leukocyte mobility and costimulation
Vcam1	0.49	Vascular cell adhesion molecule 1	CD106	Endothelial-cell adhesion molecule that binds to ITGA4/ITGB1 on leukocytes and mediates both adhesion and signal transduction
**Antigen Processing & Presentation**				
H2-D1	0.461	Histocompatibility 2, D region locus 1		MHC class I molecule; presents Ags to $CD8^+$ T cells

Psmb10	0.427	Proteasome subunit beta type 10		20S core β subunit of the proteasome involved in Ag processing to generate class I binding peptides
Tap1	0.5	Transporter antigen peptide 1	Really interesting new gene 4 (RING4)	ATP-binding cassette transporter that pumps degraded cytosolic peptides from the cytosol to the ER for packaging into MHC class I molecules
**Autophagy**				
Irgm2	0.747	Immunity-related GTPase family M member 2	Interferon-inducible protein 1 (IFI1)	Function not fully known, but most likely regulates autophagy and pro-inflammatory cytokine production
Ubc	0.289	Polyubiquitin C		Serves various roles, including innate immunity, DNA repair, and stimulation of autophagy and the proteasomal response
**Complement & Humoral Immunity**				
Cfb	0.567	Complement factor B		Alternate complement pathway component; when cleaved, produces a serine protease that binds to C3b to form C3 convertase
Fcgr4	0.898	Fragment crystallizable gamma receptor 4	Fc receptor-like 3 (Fcrl3); CD16-2	Putative mouse ortholog to human FcγRIIIA

Icam1	0.473	Intracellular adhesion molecule 1	CD54	Cell surface glycoprotein that serves as strong adhesive ligand for LFA-1; important for leukocyte mobility and costimulation
**Costimulation**				
Cd28	1.16	Cluster of differentiation 28		Essential T cell co-receptor that enhances T cell activation, proliferation, cytokine production, and survival; binds to CD80 and CD86
Icos	0.608	Inducible T cell costimulator	CD278	Enhances all basic T cell responses to foreign Ag; essential both for efficient interaction between T and B cells and for normal Ab responses to T cell-dependent Ags
**Chemotaxis**				
Ccl5	1.11	C-C motif chemokine ligand 5	Regulated upon activation, normally T-expressed, and presumably secreted (RANTES)	Chemoattractant ligand for CCR1, -3, -4, and -5; attracts blood monocytes, memory T helper cells and eosinophils; causes the release of histamine from basophils and activates eosinophils
Ccr1	0.523	C-C motif chemokine receptor 1	MIP1α receptor	Receptor for CCL3, -5, -7, and -23

Cxcl9	0.992	C-X-C motif chemokine ligand 9	Humig	Chemoattractant ligand for CXCR3; attracts activated T cells
Cxcl11	0.852	C-X-C motif chemokine ligand 11		Dominant ligand for CXCR3; attracts activated T cells; strongly induced by IFNγ
Cxcr6	0.817	C-X-C motif chemokine receptor 6	CD186	Receptor for the C-X-C chemokine CXCL16; expressed in several T lymphocyte subsets and bone marrow stromal cells
**Growth/Proliferation**				
Map2k1	0.297	Dual specificity mitogen-activated protein kinase kinase 1	MAPK/ERK kinase 1 (MEK1)	Essential component of the MAP kinase signal transduction pathway; participates in numerous biological functions, including cell growth, adhesion, survival, differentiation, transcription, metabolism, and cytoskeletal remodeling
Stat1	0.641	Signal transducer and activator of transcription 1		Transcriptional activator that mediates cellular responses to IFNs, cytokines, and other growth factors
Vegfa	0.397	Vascular endothelial growth factor A		Glysosylated mitogen that promotes vascular permeability, vasculogenesis, angiogenesis, and cell migration
Yy1	0.197	Yin yang 1		Ubiquitous factor that serves as a transcriptional "switch", either promoting

				or repressing the transcription of numerous genes through the selective recruitment of either histone deacetylases or acetyltransferases; plays a fundamental role in diverse processes, such as differentiation, replication, and cellular proliferation
**Inflammation**				
Cebpb	0.312	CCAAT/enhancer-binding protein beta		Critical macrophage TF that promotes expression of several acute-phase and inflammatory cytokine genes, including Il6
Irf1	0.471	Interferon regulatory factor 1		Transcriptional regulator that promotes inflammatory innate and adaptive immune responses
Irgm2	0.747	Immunity-related GTPase family M member 2	Interferon-inducible protein 1 (IFI1)	Function not fully known, but most likely regulates autophagy and pro-inflammatory cytokine production
Isg20	0.446	Interferon-stimulated gene 20		IFN-induced antiviral exoribonuclease that acts on ssRNA with minor activity towards ssDNA
Lgals3	0.415	Galectin 3		Galactose-specific lectin that binds IgE; involved in acute inflammatory responses, including neutrophil activation and adhesion, chemoattraction of

				monocytes/macrophages, opsonization of apoptotic neutrophils, and activation of mast cells
Nos2	1.19	Inducible nitric oxide synthase (iNOS)		Produces reactive oxygen species and contributes to inflammatory cytokine production
**Inhibition**				
Cd47	0.269	Cluster of differentiation 47	Integrin-associated protein (IAP)	Partners with membrane integrins to serve as an inhibitor of phagocytosis
Cd274	0.723	Cluster of differentiation 274	Programmed cell death receptor ligand 1 (PD-L1)	Ubiquitously expressed ligand for co-inhibitory receptor PD-1; upregulated by tumors as an immune evasion strategy
Cdkn1a	0.381	Cyclin dependent kinase inhibitor 1A	p21; CDK-interaction protein 1 (CIP1)	Binds to and inhibits cyclin-dependent kinase activity, preventing phosphorylation of critical cyclin-dependent kinase substrates and blocking cell cycle progression
Ctla4	0.66	Cytotoxic T lymphocyte antigen 4	CD152	Inhibitory receptor that blocks CD28 costimulation by competitively binding its ligands CD80 and CD86
Dusp4	0.318	Dual specificity phosphatase 4		Inactivates ERK1, ERK2, and JNK

Foxp3	0.496	Forkhead box P3	DIETER	Master TF for Tregs; represses expression of Il2 and Ifng; activates expression of Tnfrsf18, Il2ra, and Ctla4
Ido1	1.19	Indoleamine 2,3-dioxygenase 1		Initiates catabolism of tryptophan; limits immunopathology by inhibiting T cell division
Lag3	0.527	Lymphocyte activating gene 3	CD223	Inhibitory receptor on activated T cells; binds to ligands, such as FGL1; constitutively expressed on a subset of regulatory Tregs and contributes to their suppressive function; acts as a negative regulator of plasmacytoid DC activation
Socs1	0.826	Suppressor of cytokine signaling 1		Inhibits JAK proteins; negative regulator of IL-6
Tigit	0.5	T cell immunoreceptor with Ig and ITIM domains		Binds with high affinity to the poliovirus receptor, causing increased secretion of IL-10, decreased secretion of IL-12B, and suppressing T cell activation by promoting the generation of mature immunoregulatory DCs
**Interferon Response**				

Irf1	0.471	Interferon regulatory factor 1		Transcriptional regulator that promotes inflammatory innate and adaptive immune responses
Irgm2	0.747	Immunity-related GTPase family M member 2	Interferon-inducible protein 1 (IFI1)	Function not fully known, but most likely regulates autophagy and pro-inflammatory cytokine production
Isg20	0.446	Interferon-stimulated gene 20		IFN-induced antiviral exoribonuclease that acts on ssRNA with minor activity towards ssDNA
Mx2	0.602	Myxovirus resistance protein 2		IFN-induced dynamin-like GTPase with potent antiviral activity against HIV-1
Stat1	0.641	Signal transducer and activator of transcription 1		Transcriptional activator that mediates cellular responses to IFNs, cytokines, and other growth factors
**Macrophage Function**				
Cd14	0.264	Cluster of differentiation 14		PRR that recognizes LPS; mostly found on macrophages
Cebpb	0.312	CCAAT/enhancer-binding protein beta		Critical macrophage TF that promotes expression of several acute-phase and inflammatory cytokine genes, including Il6

Slamf7	0.615	Signaling lymphocytic activation molecule family member 7		A super-activator of macrophages and a strong promoter of phagocytosis; binds to CD74
**Migration/Motility**				
App	0.196	Amyloid-beta precursor protein		Cell surface receptor and transmembrane precursor protein that is cleaved by secretases to form a number of peptides; involved in cell mobility, copper homeostasis, and oxidative stress
Ccl5	1.11	C-C motif chemokine ligand 5	Regulated upon activation, normally T-expressed, and presumably secreted (RANTES))	Chemoattractant ligand for CCR1, -3, -4, and -5; attracts blood monocytes, memory T helper cells and eosinophils; causes the release of histamine from basophils and activates eosinophils
Cxcl9	0.992	C-X-C motif chemokine ligand 9	Humig	Chemoattractant ligand for CXCR3; attracts activated T cells
Cxcl11	0.852	C-X-C motif chemokine ligand 11		Dominant ligand for CXCR3; attracts activated T cells; strongly induced by IFNγ
Cxcr6	0.817	C-X-C motif chemokine receptor 6	CD186	Receptor for the C-X-C chemokine CXCL16; expressed in several T lymphocyte subsets and bone marrow stromal cells

Lgals3	0.415	Galectin 3		Galactose-specific lectin that binds IgE; involved in acute inflammatory responses, including neutrophil activation and adhesion, chemoattraction of monocytes/macrophages, opsonization of apoptotic neutrophils, and activation of mast cells
Vegfa	0.397	Vascular endothelial growth factor A		Glysosylated mitogen that promotes vascular permeability, vasculogenesis, angiogenesis, and cell migration
**NK Cell Function**				
Cd8a	0.766	Cluster of differentiation 8 alpha	LEU2	Alpha chain of the CD8 coreceptor, which binds to MHC class I; in T cells, functions primarily as a coreceptor for MHC class I molecule:peptide complex; in NK cells, the presence of CD8α homodimers at the cell surface provides a survival mechanism allowing conjugation and lysis of multiple target cells
Gzmb	0.895	Granzyme B	Fragmentin 2	Abundant protease in the cytosolic granules of cytotoxic T and NK cells that activates caspase-mediated cell death when

				delivered into the target cell through the immunological synapse
Gzmk	1.66	Granzyme K	Tryptase II	Granule-secreted, pro-apoptotic serine protease found in the cytoplasmic granules of CTLs
Prdm1	0.82	Positive regulatory domain I-binding factor	B lymphocyte-induced maturation protein (BLIMP1)	TF that plays a role in the development, retention, and long-term establishment of T cell, NK cell, and NK-T cells in non-lymphoid organs; drives the maturation of B cell into Ig secreting cells
**ROS Generation & Protection**				
Cybb	0.418	Cytochrome b-245 heavy chain	Nox2	Part of the NADPH oxidase process; generates superoxides
Nos2	1.19	Inducible nitric oxide synthase (iNOS)		Produces reactive oxygen species and contributes to inflammatory cytokine production
**T Cell Function**				
Cd3d	0.606	Cluster of differentiation 3 delta		Component of the TCR-CD3 complex; upon phosphorylation by Lck, serves as a docking station for downstream TCR signaling adaptors

Cd8a	0.766	Cluster of differentiation 8 alpha	LEU2	Alpha chain of the CD8 coreceptor, which binds to MHC class I; in T cells, functions primarily as a coreceptor for MHC class I molecule:peptide complex; in NK cells, the presence of CD8α homodimers at the cell surface provides a survival mechanism allowing conjugation and lysis of multiple target cells
Cd28	1.16	Cluster of differentiation 28		Essential T cell co-receptor that enhances T cell activation, proliferation, cytokine production, and survival; binds to CD80 and CD86
Gzmb	0.895	Granzyme B	Fragmentin 2	Abundant protease in the cytosolic granules of cytotoxic T and NK cells that activates caspase-mediated cell death when delivered into the target cell through the immunological synapse
Gzmk	1.66	Granzyme K	Tryptase II	Granule-secreted, pro-apoptotic serine protease found in the cytoplasmic granules of CTLs
Icos	0.608	Inducible T cell costimulator	CD278	Enhances all basic T cell responses to foreign Ag; essential both for efficient interaction between T and B cells and for

				normal Ab responses to T cell-dependent Ags
Prdm1	0.82	Positive regulatory domain I-binding factor	B lymphocyte-induced maturation protein (BLIMP1)	TF that plays a role in the development, retention, and long-term establishment of T cell, NK cell, and NK-T cells in non-lymphoid organs; drives the maturation of B cell into Ig secreting cells
**Transcription Factors & Coactivators**				
Cebpb	0.312	CCAAT/enhancer-binding protein beta		Critical macrophage TF that promotes expression of several acute-phase and inflammatory cytokine genes, including Il6
Irf1	0.471	Interferon regulatory factor 1		Transcriptional regulator that promotes inflammatory innate and adaptive immune responses
Prdm1	0.82	Positive regulatory domain I-binding factor	B lymphocyte-induced maturation protein (BLIMP1)	TF that plays a role in the development, retention, and long-term establishment of T cell, NK cell, and NK-T cells in non-lymphoid organs; drives the maturation of B cell into Ig secreting cells
Stat1	0.641	Signal transducer and activator of transcription 1		Transcriptional activator that mediates cellular responses to IFNs, cytokines, and other growth factors

Yy1	0.197	Yin yang 1		Ubiquitous factor that serves as a transcriptional "switch", either promoting or repressing the transcription of numerous genes through the selective recruitment of either histone deacetylases or acetyltransferases; plays a fundamental role in diverse processes, such as differentiation, replication, and cellular proliferation

NBTXR3+PBT+αPD1 vs Ctrl				
**Gene**	**Log2 fold change**	**Full Name**	**Notable Aliases**	**Function**
**Acute Phase Response**				
App	0.157	Amyloid-beta precursor protein		Cell surface receptor and transmembrane precursor protein that is cleaved by secretases to form a number of peptides; involved in cell mobility, copper homeostasis, and oxidative stress
Psen2	0.337	Presenilin-2		Putative catalytic subunit of the gamma-secretase complex, which cleaves integral membrane proteins such as APP; also

				involved in Ca(2+) homeostasis between the ER and the mitochondria
**Adhesion & Cell-Cell Interactions**				
Cd37	1.12	Cluster of differentiation 37	Tetraspanin-26	Cell surface glycoprotein known to complex with integrins and other transmembrane 4 superfamily proteins; may play a role in T cell-B cell interactions
Cd97	0.29	Cluster of differentiation 97	BL-Ac[F2]	GPCR that promotes granulocyte adhesion and migration; activates T cells via binding to CD55; stimulates angiogenesis through binding integrin counterreceptors on endothelial cells
Cyfip2	0.877	Cytoplasmic FMR1-interacting protein 2		Involved in T cell adhesion and p53/TP53-dependent induction of apoptosis
Dpp4	0.388	Dipeptidyl-peptidase 4	CD26	Cell surface glycoprotein receptor involved in the costimulatory signal essential for TCR-mediated T cell activation; serine exopeptidase that cleaves various substrates, thereby inactivating them; involved in the migration and invasion of endothelial cells into the ECM; also regulates lymphocyte-epithelial cell adhesion

Itgam	0.681	Integrin alpha M	CD11b	Pairs with CD18 to form Mac-1 aka complement receptor 3; mediates leukocyte activation, adhesion, chemotaxis, migration, phagocytosis, and cell-mediated cytotoxicity; serves as a macrophage marker
Itgax	0.617	Integrin alpha X	CD11c	Adhesion molecule; signature marker of Ag-presenting DCs
Itgb2	0.735	Integrin subunit beta 2		Pairs with ITGAL to form a receptor for ICAM1, with ITGAM or ITGAX for iC3b and fibronectin
Lgals3	0.299	Galectin 3		Galactose-specific lectin that binds IgE; involved in acute inflammatory responses, including neutrophil activation and adhesion, chemoattraction of monocytes macrophages, opsonization of apoptotic neutrophils, and activation of mast cells
Map2k1	0.181	Dual specificity mitogen-activated protein kinase kinase 1	MAPK/ERK kinase 1 (MEK1)	Essential component of the MAP kinase signal transduction pathway; participates in numerous biological functions, including cell growth, adhesion, survival, differentiation, transcription, metabolism, and cytoskeletal remodeling

Sell	1.15	L-selectin		Mediates cell adhesion by binding to glycoproteins on neighboring cells
**Antigen Processing & Presentation**				
Cd1d1	0.628	Cluster of differentiation 1 D1		Murine non-classical class I MHC; primarily presents lipid and glycolipid Ags
Ctss	0.736	Cathepsin S		Lysosomal protease that participates in processing of Ag by MHC class II
Cyfip2	0.877	Cytoplasmic FMR1-interacting protein 2		Involved in T cell adhesion and p53/TP53-dependent induction of apoptosis
H2-D1	0.353	Histocompatibility 2, D region locus 1		MHC class I molecule; presents Ags to $CD8^+$ T cells
H2-M3	0.603	Histocompatibility 2, M region locus 3		MHC class Ib molecule; presents Ags to $CD8^+$ T cells, with a preference for N-formylated peptides
H2-T23	0.67	Histocompatibility 2, Q region locus 10		MHC class I molecule; presents Ags to $CD8^+$ T cells
Psmb10	0.446	Proteasome subunit beta type 10		20S core β subunit of the proteasome involved in Ag processing to generate class I binding peptides
**Anti-Inflammatory**				

Bcl2	0.418	B cell lymphoma 2		Outer mitochondrial membrane protein that inhibits apoptosis and autophagy; may attenuate inflammation by impairing inflammasome formation
Cd200r1	0.436	Cluster of differentiation 200 receptor 1		Inhibitory receptor for the CD200/OX2 cell surface glycoprotein; limits inflammation by inhibiting the expression of proinflammatory molecules including TNFα, IFNs, and iNOS
Ctla4	0.786	Cytotoxic T lymphocyte antigen 4	CD152	Inhibitory receptor that blocks CD28 costimulation by competitively binding its ligands CD80 and CD86
Tnfaip3	0.387	Tumor necrosis factor, alpha-induced protein 3		Ubiquitin-editing enzyme that complexes with ITCH to degrade inflammatory signaling components in the TNF, IL1, and TLR pathways; targets TRAF2, TRAF6, and IKK
**Apoptosis**				
Bid	0.285	BH3 interacting domain death agonist	Desmocollin type 4, apoptotic death agonist	Induces caspases and apoptosis; counters the protective effect of BCL2, allowing release of cytochrome C

Casp1	0.417	Caspase 1	Interleukin 1β convertase	Cysteine-aspartic acid protease that mediates cleavage-based activation of IL-1β and IL-18; serves as the central enzymatic core of the inflammasome; also induces apoptosis
Cyfip2	0.877	Cytoplasmic FMR1-interacting protein 2		Involved in T cell adhesion and p53/TP53-dependent induction of apoptosis
Fas	0.901	Fragment apoptosis stimulating		Cell surface death receptor; interaction with FAS-ligand triggers an apoptotic signaling cascade; also activates NFκB, ERK1, and MAPK8
Trp53	0.403	Transformation-related protein 53		TF that induces cell-cycle arrest and apoptosis through stimulation of Fas expression
**B Cell-associated Genes**				
Blnk	1.18	B cell linker	Src homology 1 domain-containing leukocyte protein of 65 kDa (SLP-65); Ly57	Functions as a central linker protein downstream of the B cell receptor, bridging SYK kinase to a multitude of signaling pathways and regulating biological outcomes of B cell function and development; plays a role in the activation of ERK/EPHB2, MAP kinase p38 and JNK;

				modulates AP1 activation; important for the activation of NFκB and NFAT
Btk	0.615	Bruton's tyrosine kinase		Crucial kinase in B cell receptor signal transmission and B cell activation
Btla	1.73	B and T lymphocyte attenuator	CD272	Inhibitory cell surface protein that inhibits T cell function by binding to B7H4 and TNFRSF14
Cd19	2.29	Cluster of differentiation 19	B-lymphocyte surface antigen B4	BCR coreceptor; activates signaling pathways that lead to the activation of PI3K and Ca(2+) flux
Cd22	2.08	Cluster of differentiation 22	Sialic acid-binding Ig-like lectin 2 (SIGLEC2)<br>B lymphocyte cell adhesion molecule (BL-CAM)	Mediates interactions between B cells; binds CD45
Cd37	1.12	Cluster of differentiation 37	Tetraspanin-26	Cell surface glycoprotein known to complex with integrins and other transmembrane 4 superfamily proteins; may play a role in T cell-B cell interactions
Cd48	0.639	Cluster of differentiation 48	B-lymphocyte activation marker	B cell-specific cellular differentiation Ag; when bound to CD2, promotes T cell

			(BLAST-1); signaling lymphocytic activation molecule 2 (SLAMF2)	activation, and the formation of lipid rafts and caveolae for macrophages
Cd69	0.997	Cluster of differentiation 69	C-type lectin domain family 2, member C	Signal transmitting receptor in lymphocytes, NK cells, and platelets; induced upon T cell activation; involved in lymphocyte proliferation
Cd79b	2.51	Cluster of differentiation 79b	B29	One of the two flanking proteins that initiate signaling downstream of the BCR
Fcgr1	0.456	Fc fragment of IgG receptor Ia	CD64	High affinity receptor for the Fc region of γ-Igs; functions in both innate and adaptive immune responses
Icosl	0.655	Inducible T cell costimulator ligand	CD275	Ligand for T cell-specific co-receptor ICOS; also induces B cell proliferation and plasma cell differentiation
Ikzf1	0.994	IKAROS family zinc finger 1		Transcriptional regulator of hematopoietic cell differentiation; plays a role in T and B cell development
Lyn	0.359	Lck/Yes-related novel kinase		Src family tyrosine kinase that potentiates signaling from the B cell receptor and CD40

Ms4a1	2.89	Membrane spanning 4-domains A1	CD20; Bp35	B cell-specific membrane protein that functions as a store-operated Ca(2+) channel component, promoting Ca(2+) influx after BCR activation
Pik3cd	0.741	Phosphatidylinositol-4,5-bisphosphate 3-kinase catalytic subunit delta isoform		A subunit of PI3K; acts downstream of TLR4, TCR, BCR, and CD40; contributes to T helper cell expansion, mast cell development, and neutrophil chemotaxis, extravasation, and respiratory burst
Pik3cg	0.387	Phosphatidylinositol-4,5-bisphosphate 3-kinase catalytic subunit gamma isoform		A subunit of PI3K; modulates leukocyte chemotaxis to inflammatory sites and in response to chemoattractant agents
Pou2f2	1.4	POU domain class 2, transcription factor 2		TF that regulates Ab and IL-6 expression in B cells
Spn	0.812	Sialophorin	Leukosialin; CD43	Cell surface sialoglycoprotein expressed by T cells, B cells, monocytes, and granulocytes; promotes lymph node localization in T cells; shunts T cells away from the T_H2 phenotype and towards T_H1; promotes the expression of IFNγ in $CD4^+$ T cells

Tnfrsf13c	2.42	TNF receptor superfamily member 13C		B cell-activating factor; enhances B cell survival in vitro; regulator of the peripheral B cell population
Zap70	0.772	Zeta chain of T cell receptor associated protein kinase 70		Tyrosine kinase that plays an essential role in regulation of the adaptive immune response; regulates motility, adhesion, proliferation, differentiation, and cytokine expression in T cells; contributes to the development and activation of primary B cells; phosphorylates at least two essential adapter proteins: LAT and LCP2, leading in turn to a large number of signaling molecules being recruited
**Cell Cycle**				
Atm	0.29	Ataxia telangiectasia mutated		Serine/threonine protein kinase that activates checkpoint signaling upon DSBs, apoptosis, and genotoxic stresses; acts as a master controller for cell cycle checkpoint signaling pathways required for the DNA damage response and genomic stability
Cdkn1a	0.478	Cyclin dependent kinase inhibitor 1A	p21; CDK-interaction protein 1 (CIP1)	Binds to and inhibits cyclin-dependent kinase activity, preventing phosphorylation of critical cyclin-dependent kinase

				substrates and blocking cell cycle progression
**Chemotaxis**				
Ccl3	0.891	C-C motif chemokine ligand 3	Macrophage inflammatory protein 1α (MIP1α)	Chemoattractant ligand for CCR1, -4, and -5
Ccl4	0.609	C-C motif chemokine ligand 4	Macrophage inflammatory protein 1β (MIP1β)	Chemoattractant for NK cells and monocytes; binds to CCR5 receptors
Ccl5	1.03	C-C motif chemokine ligand 5	Regulated upon activation, normally T-expressed, and presumably secreted (RANTES))	Chemoattractant ligand for CCR1, -3, -4, and -5; attracts blood monocytes, memory T helper cells and eosinophils; causes the release of histamine from basophils and activates eosinophils
Ccr1	0.523	C-C motif chemokine receptor 1	MIP1α receptor	Receptor for CCL3, -5, -7, and -23
Ccr5	0.68	C-C motif chemokine receptor 5	CD195	Receptor for a number of inflammatory CC-chemokines, including CCL3/MIP1α, CCL4/MIP1β, and RANTES; signals via Ca(2+) flux
Ccrl2	0.338	C-C chemokine receptor-like 2		Stabilizes TLR4 surface expression on macrophages

Cxcl2	0.609	C-X-C motif chemokine ligand 2	Macrophage inflammatory protein 2-alpha (MIP2α); GRO2 oncogene	Chemokine produced by activated monocytes and neutrophils and expressed at sites of inflammation
Cxcl3	0.724	C-X-C motif chemokine ligand 3	GRO3 oncogene	Ligand for CXCR2; attracts neutrophils
Cxcl9	0.677	C-X-C motif chemokine ligand 9	Humig	Chemoattractant ligand for CXCR3; attracts activated T cells
Cxcl10	0.709	C-X-C motif chemokine ligand 10	IFNγ-induced protein 10 (IP-10)	Macrophage, DC, T cell, and NK cell chemattractant secreted by several cell types in response to IFNγ; binds to CXCR3
Cxcl11	0.769	C-X-C motif chemokine ligand 11		Dominant ligand for CXCR3; attracts activated T cells; strongly induced by IFNγ
Cxcr3	0.768	C-X-C motif chemokine receptor 3	CD183	Induces integrin activation, cytoskeletal remodeling, and chemotaxis; expressed by T cells and NK cells; prominently expressed in effector and memory T cells
Cxcr4	0.743	C-X-C motif chemokine receptor 4	CD184; fusin	Alpha-chemokine receptor specific for SDF1 aka CXCL12

Cxcr6	0.736	C-X-C motif chemokine receptor 6	CD186	Receptor for the C-X-C chemokine CXCL16; expressed in several T lymphocyte subsets and bone marrow stromal cells
Isg15	0.717	Interferon-stimulated gene 15		Ubiquitin-like protein that binds intracellular target proteins upon activation by IFNα or β; can also be secreted to induce NK cell proliferation, act as a chemoattractant for neutrophils, and induce IFNγ upon binding to ITGAL/ITGB2
Itgam	0.681	Integrin alpha M	CD11b	Pairs with CD18 to form Mac-1 aka complement receptor 3; mediates leukocyte activation, adhesion, chemotaxis, migration, phagocytosis, and cell-mediated cytotoxicity; serves as a macrophage marker
Pik3cd	0.741	Phosphatidylinositol-4,5-bisphosphate 3-kinase catalytic subunit delta isoform		A subunit of PI3K; acts downstream of TLR4, TCR, BCR, and CD40; contributes to T helper cell expansion, mast cell development, and neutrophil chemotaxis, extravasation, and respiratory burst
**Complement & Humoral Immunity**				
C3ar1	0.506	Complement component 3a receptor 1		GPCR that binds to C3a, activating chemotaxis, granule enzyme release,

				superoxide anion production, and bacterial opsonization
Cfb	0.358	Complement factor B		Alternate complement pathway component; when cleaved, produces a serine protease that binds to C3b to form C3 convertase
Fcgr1	0.456	Fc fragment of IgG receptor Ia	CD64	High affinity receptor for the Fc region of γ-Igs; functions in both innate and adaptive immune responses
Fcgr4	1.13	Fragment crystallizable gamma receptor 4	Fc receptor-like 3 (Fcrl3); CD16-2	Putative mouse ortholog to human FcγRIIIA
**Costimulation**				
Cd40	0.843	Cluster of differentiation 40		APC-expressed costimulatory protein that binds to CD40L on $CD4^+$ T cells, causing activation of both
Dpp4	0.388	Dipeptidyl-peptidase 4	CD26	Cell surface glycoprotein receptor involved in the costimulatory signal essential for TCR-mediated T cell activation; serine exopeptidase that cleaves various substrates, thereby inactivating them; involved in the migration and invasion of endothelial cells into the ECM; also

				regulates lymphocyte-epithelial cell adhesion
Icos	0.984	Inducible T cell costimulator	CD278	Enhances all basic T cell responses to foreign Ag; essential both for efficient interaction between T and B cells and for normal Ab responses to T cell-dependent Ags
Icosl	0.655	Inducible T cell costimulator ligand	CD275	Ligand for T cell-specific co-receptor ICOS; also induces B cell proliferation and plasma cell differentiation
Ptprc	0.937	Protein tyrosine phosphatase receptor type C	CD45; leukocyte common antigen (LCA)	Delivers costimulation during T cell activation upon binding to its ligand DPP4; dephosphorylates Lyn and suppresses JAK kinases
Tnfrsf13c	2.42	TNF receptor superfamily member 13C		B cell-activating factor; enhances B cell survival in vitro; regulator of the peripheral B cell population
Tnfrsf14	0.671	Tumor necrosis factor receptor superfamily member 14	CD270	Receptor for four distinct ligands: LIGHT, lymphotoxin-α, BTLA, and CD160, altogether defining a complex stimulatory and inhibitory signaling network; signals via the TRAF2-TRAF3 E3 ligase pathway to promote immune cell survival and

				differentiation; participates in bidirectional cell-cell contact signaling between APCs and lymphocytes; delivers costimulatory signals to T cells, promoting cell proliferation and effector functions; interacts with CD160 on NK cells, enhancing IFNγ production and antitumor immune response; upon binding to CD160 on activated $CD4^+$ T cells, downregulates CD28 costimulatory signaling; participates in cis or trans reactions with BTLA; cis interactions seem to promote quiescence; trans interactions seem to promote survival
**Cytokines**				
Il1a	0.916	Interleukin 1 alpha	Hematopoietin-1	Cytokine produced by monocytes and macrophages in response to cell injury; stimulates thymocyte proliferation by inducing IL-2 release; also stimulates B cell maturation and proliferation, and fibroblast growth factor activity
Il1b	0.694	Interleukin 1 beta	Catabolin	One of the two primary inflammatory cytokines produced by the inflammasome (the other one being IL-18); induces neutrophil influx and activation, T cell

				activation and cytokine production, B cell activation and Ab production, fibroblast proliferation, and collagen production; synergizes with IL-12 to induce IFNγ synthesis from T_H1 cells
Il2rg	0.874	Interleukin 2 receptor subunit gamma	Common gamma chain; CD132	Common subunit for the receptors for a variety of interleukins, including IL-2, -4, -7, and -21
Il7r	1.07	Interleukin 7 receptor	CD127	Receptor for IL-7
Il12rb1	0.83	Interleukin 12 receptor subunit beta 1	CD212	Cytokine receptor component that associates with IL12RB2 to IL23R
Il12rb2	0.612	Interleukin 12 receptor subunit beta 2		Signaling component coupling to the JAK2/STAT4 pathway; promotes T and NK cell proliferation; promotes T_H1 polarization by enhancing IFNγ production
**Cytotoxicity**				
Gzmb	1.2	Granzyme B	Fragmentin 2	Abundant protease in the cytosolic granules of cytotoxic T and NK cells that activates caspase-mediated cell death when

				delivered into the target cell through the immunological synapse
Gzmk	1.07	Granzyme K	Tryptase II	Granule-secreted, pro-apoptotic serine protease found in the cytoplasmic granules of CTLs
Fcgr4	1.13	Fragment crystallizable gamma receptor 4	Fc receptor-like 3 (Fcrl3); CD16-2	Putative mouse ortholog to human FcγRIIIA
**Growth/Proliferation**				
Map2k1	0.181	Dual specificity mitogen-activated protein kinase kinase 1	MAPK/ERK kinase 1 (MEK1)	Essential component of the MAP kinase signal transduction pathway; participates in numerous biological functions, including cell growth, adhesion, survival, differentiation, transcription, metabolism, and cytoskeletal remodeling
Rps6	0.492	Ribosomal protein 6		Component of the 40S small ribosomal subunit; plays an important role in controlling cell growth and proliferation through the selective translation of particular classes of mRNA
Yy1	0.0857	Yin yang 1		Ubiquitous factor that serves as a transcriptional "switch", either promoting or repressing the transcription of numerous

				genes through the selective recruitment of either histone deacetylases or acetyltransferases; plays a fundamental role in diverse processes, such as differentiation, replication, and cellular proliferation
**Hematopoiesis**				
Hck	0.486	Hematopoietic cell kinase		Src family tyrosine kinase that mediates secretory lysosome mobilization, degranulation, and activation of NADPH oxidase
Ikzf1	0.994	IKAROS family zinc finger 1		Transcriptional regulator of hematopoietic cell differentiation; plays a role in T and B cell development
Ikzf2	0.383	IKAROS family zinc finger protein 2		Hematopoietic cell-specific TF involved in early hematopoietic development
Stat5b	0.314	Signal transducer and activator of transcription 5b		Carries out a dual function: signal transduction and activation of transcription; positively regulates hematopoietic/erythroid differentiation.
**Inflammation**				
Casp1	0.417	Caspase 1	Interleukin 1β convertase	Cysteine-aspartic acid protease that mediates cleavage-based activation of IL-1β

				and IL-18; serves as the central enzymatic core of the inflammasome; also induces apoptosis
Cd38	1.02	Cluster of differentiation 38	ADP-ribosyl cyclase 1	Synthesizes the second messengers cyclic ADP-ribose and NADPH; appears to play a critical role in inflammation, although its exact immunological function(s) remain(s) poorly defined
Ctsh	0.363	Cathepsin H		Lysosomal protease; increased in macrophages in response to IFNγ
Il1b	0.694	Interleukin 1 beta	Catabolin	One of the two primary inflammatory cytokines produced by the inflammasome (the other one being IL-18); induces neutrophil influx and activation, T cell activation and cytokine production, B cell activation and Ab production, fibroblast proliferation, and collagen production; synergizes with IL-12 to induce IFNγ synthesis from T_H1 cells
Il12rb1	0.83	Interleukin 12 receptor subunit beta 1	CD212	Cytokine receptor component that associates with IL12RB2 to IL23R

Il12rb2	0.612	Interleukin 12 receptor subunit beta 2		Signaling component coupling to the JAK2/STAT4 pathway; promotes T and NK cell proliferation; promotes T_H1 polarization by enhancing IFNγ production
Irf1	0.693	Interferon regulatory factor 1		Transcriptional regulator that promotes inflammatory innate and adaptive immune responses
Irf4	0.927	Interferon regulatory factor 4		Transcriptional activator that complexes with BATF and binds ISREs within the promoters of multiple genes involved in inflammation
Irgm2	0.564	Immunity-related GTPase family M member 2	Interferon-inducible protein 1 (IFI1)	Function not fully known, but most likely regulates autophagy and pro-inflammatory cytokine production
Isg15	0.717	Interferon-stimulated gene 15		Ubiquitin-like protein that binds intracellular target proteins upon activation by IFNα or β; can also be secreted to induce NK cell proliferation, act as a chemoattractant for neutrophils, and induce IFNγ upon binding to ITGAL/ITGB2
Jak1	0.493	Janus kinase 1		Essential tyrosine kinase involved signal transduction in type I and II cytokines and IFNs

Lgals3	0.299	Galectin 3		Galactose-specific lectin that binds IgE; involved in acute inflammatory responses, including neutrophil activation and adhesion, chemoattraction of monocytes/macrophages, opsonization of apoptotic neutrophils, and activation of mast cells
Nlrp3	0.652	NACHT domain-, leucine-rich repeat-, and PYD-containing protein 3	Cryopyrin	PRR with a wide diversity of recognized targets that activates the NLRP3 inflammasome consisting of NLRP3, PYCARD, and caspase-1/-8
Nos2	1.58	Inducible nitric oxide synthase (iNOS)		Produces reactive oxygen species and contributes to inflammatory cytokine production
Pik3cd	0.741	Phosphatidylinositol-4,5-bisphosphate 3-kinase catalytic subunit delta isoform		A subunit of PI3K; acts downstream of TLR4, TCR, BCR, and CD40; contributes to T helper cell expansion, mast cell development, and neutrophil chemotaxis, extravasation, and respiratory burst
Pik3cg	0.387	Phosphatidylinositol-4,5-bisphosphate 3-kinase catalytic		A subunit of PI3K; modulates leukocyte chemotaxis to inflammatory sites and in response to chemoattractant agents

		subunit gamma isoform		
Spn	0.812	Sialophorin	Leukosialin; CD43	Cell surface sialoglycoprotein expressed by T cells, B cells, monocytes, and granulocytes; promotes lymph node localization in T cells; shunts T cells away from the T_H2 phenotype and towards T_H1; promotes the expression of IFNγ in $CD4^+$ T cells
Tbk1	0.338	TANK-binding kinase 1		Coordinates the activation of IRF3 and NFκB and induction of type I IFNs
Tmem173	0.485	Transmembrane protein 173	Stimulator of interferon genes (STING)	Adaptor protein in type I IFN signaling; activates STAT6 and IRF3 through TBK1 to induce type I IFN production
**Inhibition**				
Bcl2	0.418	B cell lymphoma 2		Outer mitochondrial membrane protein that inhibits apoptosis and autophagy; may attenuate inflammation by impairing inflammasome formation
Btla	1.73	B and T lymphocyte attenuator	CD272	Inhibitory cell surface protein that inhibits T cell function by binding to B7H4 and TNFRSF14

Cd200r1	0.436	Cluster of differentiation 200 receptor 1		Inhibitory receptor for the CD200/OX2 cell surface glycoprotein; limits inflammation by inhibiting the expression of proinflammatory molecules including TNFα, IFNs, and iNOS
Cdkn1a	0.478	Cyclin dependent kinase inhibitor 1A	p21; CDK-interaction protein 1 (CIP1)	Binds to and inhibits cyclin-dependent kinase activity, preventing phosphorylation of critical cyclin-dependent kinase substrates and blocking cell cycle progression
Ctla4	0.786	Cytotoxic T lymphocyte antigen 4	CD152	Inhibitory receptor that blocks CD28 costimulation by competitively binding its ligands CD80 and CD86
Cd274	1.1	Cluster of differentiation 274	Programmed cell death receptor ligand 1 (PD-L1)	Ubiquitously expressed ligand for co-inhibitory receptor PD-1; upregulated by tumors as an immune evasion strategy
Cyld	0.305	Cylindromatosis lysine 63 deubiquitinase		Inhibits NFκB activation by deubiquitinating upstream signaling factors; inhibits Wnt signaling; restricts polyubiquitination of RIPK1 and -2, thereby limiting necroptosis
Irak3	0.515	Interleukin-1 receptor-associated kinase 3		Adaptor protein that negatively regulates TLR signaling; predominantly expressed in monocytes and macrophages

Foxp3	1.02	Forkhead box P3	DIETER	Master TF for Tregs; represses expression of Il2 and Ifng; activates expression of Tnfrsf18, Il2ra, and Ctla4
Nfkbia	0.553	Nuclear factor kappa B inhibitor alpha		Inhibits activity of REL dimers by masking of their nuclear localization signals
Nlrc5	0.709	NLR family CARD domain containing 5	NOD27	Inhibits NFκB and type I IFN signaling pathways; may also regulate the type II IFN signaling pathway
Socs1	0.758	Suppressor of cytokine signaling 1		Inhibits JAK proteins; negative regulator of IL-6
Tnfrsf14	0.671	TNF receptor superfamily member 14	CD270	Receptor for four distinct ligands: LIGHT, lymphotoxin-α, BTLA, and CD160, altogether defining a complex stimulatory and inhibitory signaling network; signals via the TRAF2-TRAF3 E3 ligase pathway to promote immune cell survival and differentiation; participates in bidirectional cell-cell contact signaling between APCs and lymphocytes; delivers costimulatory signals to T cells, promoting cell proliferation and effector functions; interacts with CD160 on NK cells, enhancing IFNγ production and antitumor immune response; upon binding to CD160 on activated $CD4^+$ T cells,

				downregulates CD28 costimulatory signaling; participates in cis or trans reactions with BTLA; cis interactions seem to promote quiescence; trans interactions seem to promote survival
**Interferon Response**				
Ctsh	0.363	Cathepsin H		Lysosomal protease; increased in macrophages in response to IFNγ
Irf1	0.693	Interferon regulatory factor 1		Transcriptional regulator that promotes inflammatory innate and adaptive immune responses
Irf4	0.927	Interferon regulatory factor 4		Transcriptional activator that complexes with BATF and binds ISREs within the promoters of multiple genes involved in inflammation
Irgm2	0.564	Immunity-related GTPase family M member 2	Interferon-inducible protein 1 (IFI1)	Function not fully known, but most likely regulates autophagy and pro-inflammatory cytokine production
Isg15	0.717	Interferon-stimulated gene 15		Ubiquitin-like protein that binds intracellular target proteins upon activation by IFNα or β; can also be secreted to induce NK cell proliferation, act as a

				chemoattractant for neutrophils, and induce IFNγ upon binding to ITGAL/ITGB2
Jak1	0.493	Janus kinase 1		Essential tyrosine kinase involved signal transduction in type I and II cytokines and IFNs
Mx2	0.932	Myxovirus resistance protein 2		IFN-induced dynamin-like GTPase with potent antiviral activity against HIV-1
Tmem173	0.485	Transmembrane protein 173	Stimulator of interferon genes (STING)	Adaptor protein in type I IFN signaling; activates STAT6 and IRF3 through TBK1 to induce type I IFN production
**Ion Transport**				
App	0.157	Amyloid-beta precursor protein		Cell surface receptor and transmembrane precursor protein that is cleaved by secretases to form a number of peptides; involved in cell mobility, copper homeostasis, and oxidative stress
Ms4a1	2.89	Membrane spanning 4-domains A1	CD20; Bp35	B cell-specific membrane protein that functions as a store-operated Ca(2+) channel component, promoting Ca(2+) influx after BCR activation

Slc11a1	0.95	Natural resistance-associated macrophage protein 1		Macrophage-specific metal ion transporter; uptakes divalent metal cations to neutralize ROSs
**IRAKs & TRAFs**				
Irak2	0.515	Interleukin-1 receptor-associated kinase 2		Adaptor protein involved in TLR and IL-1 signaling
Irak3	0.734	Interleukin-1 receptor-associated kinase 3		Adaptor protein that negatively regulates TLR signaling; predominantly expressed in monocytes and macrophages
**JAK-STAT Pathway**				
Jak1	0.493	Janus kinase 1		Essential tyrosine kinase involved signal transduction in type I and II cytokines and IFNs
Jak3	0.434	Janus kinase 3		Non-receptor tyrosine kinase involved in various processes such as cell growth, development, or differentiation; mediates essential signaling events in both innate and adaptive immunity

Stat1	0.675	Signal transducer and activator of transcription 1		Transcriptional activator that mediates cellular responses to IFNs, cytokines, and other growth factors
Stat5b	0.314	Signal transducer and activator of transcription 5b		Carries out a dual function: signal transduction and activation of transcription; positively regulates hematopoietic/erythroid differentiation.
Stat6	0.187	Signal transducer and activator of transcription 6		Essential TF for T_H2 $CD4^+$ T cell and macrophage function and M2 macrophage polarization
**Kinases**				
Atm	0.29	Ataxia telangiectasia mutated		Serine/threonine protein kinase that activates checkpoint signaling upon DSBs, apoptosis, and genotoxic stresses; acts as a master controller for cell cycle checkpoint signaling pathways required for the DNA damage response and genomic stability
Btk	0.615	Bruton's tyrosine kinase		Crucial kinase in B cell receptor signal transmission and B cell activation
Hck	0.486	Hematopoietic cell kinase		Src family tyrosine kinase that mediates secretory lysosome mobilization,

				degranulation, and activation of NADPH oxidase
Itk	0.932	Interleukin-2-inducible T cell kinase	LYK	Key actor in the TCR signaling cascade; phosphorylates PLCγ1, LAT, and LCP2
Lck	0.983	Lymphocyte cell kinase		Src family tyrosine kinase that acts as one of the main signaling intermediaries downstream of the TCR; constitutively associated with the cytoplasmic portion of CD4
Lyn	0.359	Tyrosine-protein kinase Lyn		Src family tyrosine kinase that potentiates signaling from the B cell receptor and CD40
Jak1	0.493	Janus kinase 1		Essential tyrosine kinase involved signal transduction in type I and II cytokines and IFNs
Jak3	0.434	Janus kinase 3		Non-receptor tyrosine kinase involved in various processes such as cell growth, development, or differentiation; mediates essential signaling events in both innate and adaptive immunity
Pik3cd	0.741	Phosphatidylinositol-4,5-bisphosphate 3-		A subunit of PI3K; acts downstream of TLR4, TCR, BCR, and CD40; contributes to T helper cell expansion, mast cell development, and

		kinase catalytic subunit delta isoform		neutrophil chemotaxis, extravasation, and respiratory burst
Pik3cg	0.387	Phosphatidylinositol-4,5-bisphosphate 3-kinase catalytic subunit gamma isoform		A subunit of PI3K; modulates leukocyte chemotaxis to inflammatory sites and in response to chemoattractant agents
Tbk1	0.338	TANK-binding kinase 1		Coordinates the activation of IRF3 and NFκB and induction of type I IFNs
Txk	1.07	TXK tyrosine kinase		Regulates the development, function, and differentiation of conventional T cells and nonconventional NK-T cells; contributes to signaling from many receptors and participates in multiple downstream pathways, including regulation of the actin cytoskeleton; can phosphorylate PLCγ1, leading to its localization in lipid rafts and activation, followed by subsequent cleavage of its substrates
Zap70	0.772	Zeta chain of T cell receptor associated protein kinase 70		Tyrosine kinase that plays an essential role in regulation of the adaptive immune response; regulates motility, adhesion, proliferation, differentiation, and cytokine expression in T cells; contributes to the

				development and activation of primary B cells; phosphorylates at least two essential adapter proteins: LAT and LCP2, leading in turn to a large number of signaling molecules being recruited
**Lysosomal Activity**				
Ctsh	0.363	Cathepsin H		Lysosomal protease; increased in macrophages in response to IFNγ
Ctss	0.736	Cathepsin S		Lysosomal protease that participates in processing of Ag by MHC class II
Hck	0.486	Hematopoietic cell kinase		Src family tyrosine kinase that mediates secretory lysosome mobilization, degranlation, and activation of NADPH oxidase
**Macrophage Function**				
Cd14	0.268	Cluster of differentiation 14		PRR that recognizes LPS; mostly found on macrophages
Ctsh	0.363	Cathepsin H		Lysosomal protease; increased in macrophages in response to IFNγ

Irak3	0.515	Interleukin-1 receptor-associated kinase 3		Adaptor protein that negatively regulates TLR signaling; predominantly expressed in monocytes and macrophages
Itgam	0.681	Integrin alpha M	CD11b	Pairs with CD18 to form Mac-1 aka complement receptor 3; mediates leukocyte activation, adhesion, chemotaxis, migration, phagocytosis, and cell-mediated cytotoxicity; serves as a macrophage marker
Marco	2.03	Macrophage receptor with collagenous structure		A PRR that recognizes LDL
Slamf7	1.07	Signaling lymphocytic activation molecule family member 7		A super-activator of macrophages and a strong promoter of phagocytosis; binds to CD74
Slc11a1	0.95	Natural resistance-associated macrophage protein 1		Macrophage-specific metal ion transporter; uptakes divalent metal cations to neutralize ROSs
**MAP Kinase Signaling**				
Mapk1	0.444	Mitogen-activated protein kinase 1	Extracellular signal-regulated kinase 2 (ERK2)	Serine/threonine kinase that acts as an essential component of the MAP kinase signal transduction pathway

Mapk11	0.345	Mitogen-activated protein kinase 11	Stress-activated protein kinase 2 (SAPK2)	Serine/threonine kinase that acts as an essential component of the MAP kinase signal transduction pathway
Map2k1	0.181	Dual specificity mitogen-activated protein kinase kinase 1	MAPK/ERK kinase 1 (MEK1)	Essential component of the MAP kinase signal transduction pathway; participates in numerous biological functions, including cell growth, adhesion, survival, differentiation, transcription, metabolism, and cytoskeletal remodeling
Map4k2	0.536	Mitogen-activated protein kinase kinase kinase kinase 2		Essential component of the MAP kinase signal transduction pathway downstream of TRAF6; upstream activator of the SAP/JNK signaling pathway;
**Mast Cell Function**				
Lgals3	0.299	Galectin 3		Galactose-specific lectin that binds IgE; involved in acute inflammatory responses, including neutrophil activation and adhesion, chemoattraction of monocytes macrophages, opsonization of apoptotic neutrophils, and activation of mast cells
Pik3cd	0.741	Phosphatidylinositol-4,5-bisphosphate 3-		A subunit of PI3K; acts downstream of TLR4, TCR, BCR, and CD40; contributes to T helper cell expansion, mast cell development, and

		kinase catalytic subunit delta isoform		neutrophil chemotaxis, extravasation, and respiratory burst
Pik3cg	0.387	Phosphatidylinositol-4,5-bisphosphate 3-kinase catalytic subunit gamma isoform		A subunit of PI3K; modulates leukocyte chemotaxis to inflammatory sites and in response to chemoattractant agents
**Metabolism**				
Abcg1	1.01	ATP-binding cassette transporter G1		Membrane-associated cholesterol efflux pump
Cd36	0.93	Cluster of differentiation 36	Fatty acid translocase (FAT)	Class B scavenger receptor that mediates fatty acid uptake
Map2k1	0.181	Dual specificity mitogen-activated protein kinase kinase 1	MAPK/ERK kinase 1 (MEK1)	Essential component of the MAP kinase signal transduction pathway; participates in numerous biological functions, including cell growth, adhesion, survival, differentiation, transcription, metabolism, and cytoskeletal remodeling
**Migration/Motility**				
App	0.157	Amyloid-beta precursor protein		Cell surface receptor and transmembrane precursor protein that is cleaved by secretases to form a number of peptides;

				involved in cell mobility, copper homeostasis, and oxidative stress
Cd97	0.29	Cluster of differentiation 97	BL-Ac[F2]	GPCR that promotes granulocyte adhesion and migration; activates T cells via binding to CD55; stimulates angiogenesis through binding integrin counterreceptors on endothelial cells
Dpp4	0.388	Dipeptidyl-peptidase 4	CD26	Cell surface glycoprotein receptor involved in the costimulatory signal essential for TCR-mediated T cell activation; serine exopeptidase that cleaves various substrates, thereby inactivating them; involved in the migration and invasion of endothelial cells into the ECM; also regulates lymphocyte-epithelial cell adhesion
Itgam	0.681	Integrin alpha M	CD11b	Pairs with CD18 to form Mac-1 aka complement receptor 3; mediates leukocyte activation, adhesion, chemotaxis, migration, phagocytosis, and cell-mediated cytotoxicity; serves as a macrophage marker
**Neutrophil Function**				

Fpr2	1.04	Formyl peptide receptor 2	Lipoxin A4 receptor	Low affinity receptor for N-formyl-methionyl peptides; activates neutrophils
Ncf4	0.545	Neutrophil cytosolic factor 4	SH3 and PX domain-containing protein 4 (SH3PXD4)	Cytosolic regulatory component of the superoxide-producing phagocyte NADPH-oxidase, a multicomponent enzyme system important for host defense
Pik3cd	0.741	Phosphatidylinositol-4,5-bisphosphate 3-kinase catalytic subunit delta isoform		A subunit of PI3K; acts downstream of TLR4, TCR, BCR, and CD40; contributes to T helper cell expansion, mast cell development, and neutrophil chemotaxis, extravasation, and respiratory burst
Cxcl3	0.724	C-X-C motif chemokine ligand 3	GRO3 oncogene	Ligand for CXCR2; attracts neutrophils
**NFκB Signaling**				
Bcl10	0.137	B cell lymphoma/leukemia 10		Activates NFκB via ubiquitination of IKKγ
Card11	1.24	Caspase recruitment domain family member 11	Bcl10-interacting MAGUK protein 3 (BIMP3)	Adapter protein that plays a key role in adaptive immune response by transducing the activation of NFκB downstream of TCR and BCR engagement; upon activation in response to TCR or BCR triggering, homooligomerizes to form a nucleating

				helical template that recruits BCL10 via CARD-CARD interaction, thereby promoting polymerization of BCL10 and subsequent recruitment of MALT1, leading to IKK phosphorylation and degradation and release of NFκB proteins for nuclear translocation
Cxcr3	0.768	C-X-C motif chemokine receptor 3	CD183	Induces integrin activation, cytoskeletal remodeling, and chemotaxis; expressed by T cells and NK cells; prominently expressed in effector and memory T cells
Ikbke	0.868	Inhibitor of nuclear factor kappa B kinase subunit epsilon		Noncanonical IκB kinase; plays an essential role in regulating inflammatory through the activation of the type I IFN, NFκB, and STAT signaling
Rel	0.623	Avian reticuloendotheliosis viral oncogene homolog	c-Rel	One of the NFκB family TFs; important for B cell and Treg development
Relb	0.332	Avian reticuloendotheliosis viral oncogene homolog B		One of the NFκB family TFs; controls lymphoid development, DC biology, and noncanonical NFκB signaling

Ripk2	0.374	Receptor-interacting serine/threonine-protein kinase 2		RIP kinase that potentiates signals downstream of NOD1 and -2, leading to NFκB activation; promotes BCL10 phosphorylation and subsequent NFκB activation following TCR engagement
**NK Cell Function**				
Cd69	0.997	Cluster of differentiation 69	C-type lectin domain family 2, member C	Signal transmitting receptor in lymphocytes, NK cells, and platelets; induced upon T cell activation; involved in lymphocyte proliferation
Gzmb	1.2	Granzyme B	Fragmentin 2	Abundant protease in the cytosolic granules of cytotoxic T and NK cells that activates caspase-mediated cell death when delivered into the target cell through the immunological synapse
Gzmk	1.07	Granzyme K	Tryptase II	Granule-secreted, pro-apoptotic serine protease found in the cytoplasmic granules of CTLs
Il12rb2	0.612	Interleukin 12 receptor subunit beta 2		Signaling component coupling to the JAK2/STAT4 pathway; promotes T and NK cell proliferation; promotes T_H1 polarization by enhancing IFNγ production

Pik3cd	0.741	Phosphatidylinositol-4,5-bisphosphate 3-kinase catalytic subunit delta isoform		A subunit of PI3K; acts downstream of TLR4, TCR, BCR, and CD40; contributes to T helper cell expansion, mast cell development, and neutrophil chemotaxis, extravasation, and respiratory burst
Pik3cg	0.387	Phosphatidylinositol-4,5-bisphosphate 3-kinase catalytic subunit gamma isoform		A subunit of PI3K; modulates leukocyte chemotaxis to inflammatory sites and in response to chemoattractant agents
Pvr	0.319	Poliovirus receptor	CD155	Mediates NK cell adhesion and triggers NK cell effector functions; binds CD96 and CD226, leading to the formation of a mature immunological synapse between NK cell and target cell
Tcf7	1.24	Transcription factor 7		HMG box TF predominantly expressed by T cells that drives their development, although also involved in NK cell development; activates transcription through a Wnt/β-catenin signaling pathway
**Pattern Recognition Receptors**				
Cd14	0.268	Cluster of differentiation 14		PRR that recognizes LPS; mostly found on macrophages

Cd180	1.2	Cluster of differentiation 180		Heterodimeric binding partner of Ly86 that participates in LPS binding in APCs
Clec7a	0.519	C-Type lectin domain family 7, member a	Dectin-1	PRR specific for β-1,3- and β-1,6-linked glucans from fungi and plants; necessary for the TLR2-mediated inflammatory response and for TLR2-mediated activation of NF-κB
Fpr2	1.04	Formyl peptide receptor 2	Lipoxin A4 receptor	Low affinity receptor for N-formyl-methionyl peptides; activates neutrophils
Ly96	0.285	Lymphocyte antigen 96	Myeloid differentiation factor 2 (MD-2)	Heterodimeric binding partner of TLR4 that participates in LPS binding
Marco	2.03	Macrophage receptor with collagenous structure		A PRR that recognizes LDL
Nlrp3	0.652	NACHT domain-, leucine-rich repeat-, and PYD-containing protein 3	Cryopyrin	PRR with a wide diversity of recognized targets that activates the NLRP3 inflammasome consisting of NLRP3, PYCARD, and caspase-1/-8
Ticam2	0.445	TIR domain-containing adaptor molecule 2		Sorting adapter in various innate immune signaling cascades; bridges TLR2 and MyD88

Tlr1	0.561	Toll-like receptor 1	CD281	Ubiquitously expressed surface PRR that recognizes diacylated and triacylated lipopeptides; pairs with TLR2 and CD14
Tlr7	0.619	Toll-like receptor 7	CD287	Endosomic PRR that recognizes ssRNA
**Phagocytosis**				
Itgam	0.681	Integrin alpha M	CD11b	Pairs with CD18 to form Mac-1 aka complement receptor 3; mediates leukocyte activation, adhesion, chemotaxis, migration, phagocytosis, and cell-mediated cytotoxicity; serves as a macrophage marker
Ncf4	0.545	Neutrophil cytosolic factor 4	SH3 and PX domain-containing protein 4 (SH3PXD4)	Cytosolic regulatory component of the superoxide-producing phagocyte NADPH-oxidase, a multicomponent enzyme system important for host defense
Slamf7	1.07	Signaling lymphocytic activation molecule family member 7		A super-activator of macrophages and a strong promoter of phagocytosis; binds to CD74
**ROS Generation & Protection**				
Cybb	0.516	Cytochrome b-245 heavy chain	Nox2	Part of the NADPH oxidase process; generates superoxides

Ncf4	0.545	Neutrophil cytosolic factor 4	SH3 and PX domain-containing protein 4 (SH3PXD4)	Cytosolic regulatory component of the superoxide-producing phagocyte NADPH-oxidase, a multicomponent enzyme system important for host defense
Nos2	1.58	Inducible nitric oxide synthase (iNOS)		Produces reactive oxygen species and contributes to inflammatory cytokine production
Txnip	0.364	Thioredoxin interacting protein		Thiol-oxidoreductase; protects cells from oxidative stress by inhibiting thioredoxin
**Stress Response**				
App	0.157	Amyloid-beta precursor protein		Cell surface receptor and transmembrane precursor protein that is cleaved by secretases to form a number of peptides; involved in cell mobility, copper homeostasis, and oxidative stress
Atm	0.29	Ataxia telangiectasia mutated		Serine/threonine protein kinase that activates checkpoint signaling upon DSBs, apoptosis, and genotoxic stresses; acts as a master controller for cell cycle checkpoint signaling pathways required for the DNA damage response and genomic stability
**T Cell Function**				

Cd2	1.09	Cluster of differentiation 2	Leukocyte functional antigen 2 (LFA-2)	Interacts with LFA-3 and CD48 to mediate adhesion between T cells and other cell types
Cd3d	1.16	Cluster of differentiation 3 delta		Component of the TCR-CD3 complex; upon phosphorylation by Lck, serves as a docking station for downstream TCR signaling adaptors
Cd3e	0.69	Cluster of differentiation 3 epsilon		Component of the TCR-CD3 complex; initiates the TCR-CD3 complex assembly by forming the two heterodimers CD3δ/CD3ε and CD3γ/CD3ε; also participates in internalization and cell surface down-regulation of TCR-CD3 complexes via endocytosis sequences present in CD3ε cytosolic region
Cd3g	0.672	Cluster of differentiation 3 gamma		Component of the TCR-CD3 complex; plays an essential role in the dynamic regulation of TCR expression at the cell surface
Cd4	1.1	Cluster of differentiation 4		Signature helper T cell marker; binds to MHC class II and provides necessary costimulation for T cell activation

Cd5	0.972	Cluster of differentiation 5	LEU1	Type-I transmembrane glycoprotein found on the surface of T and B cells; may act as a receptor in regulating T cell proliferation
Cd8b1	0.82	Cluster of differentiation 8 beta 1		Beta chain of the CD8 coreceptor, which binds to MHC class I
Cd37	1.12	Cluster of differentiation 37	Tetraspanin-26	Cell surface glycoprotein known to complex with integrins and other transmembrane 4 superfamily proteins; may play a role in T cell-B cell interactions
Cd69	0.997	Cluster of differentiation 69	C-type lectin domain family 2, member C	Signal transmitting receptor in lymphocytes, NK cells, and platelets; induced upon T cell activation; involved in lymphocyte proliferation
Cd97	0.29	Cluster of differentiation 97	BL-Ac[F2]	GPCR that promotes granulocyte adhesion and migration; activates T cells via binding to CD55; stimulates angiogenesis through binding integrin counterreceptors on endothelial cells
Cd247	1.37	Cluster of differentiation 247	T cell surface glycoprotein CD3 zeta chain	Central intracellular signaling chain of the TCR, to which downstream signaling adaptors dock

Cyfip2	0.877	Cytoplasmic FMR1-interacting protein 2		Involved in T cell adhesion and p53/TP53-dependent induction of apoptosis
Cxcr3	0.768	C-X-C motif chemokine receptor 3	CD183	Induces integrin activation, cytoskeletal remodeling, and chemotaxis; expressed by T cells and NK cells; prominently expressed in effector and memory T cells
Dpp4	0.388	Dipeptidyl-peptidase 4	CD26	Cell surface glycoprotein receptor involved in the costimulatory signal essential for TCR-mediated T cell activation; serine exopeptidase that cleaves various substrates, thereby inactivating them; involved in the migration and invasion of endothelial cells into the ECM; also regulates lymphocyte-epithelial cell adhesion
Eomes	0.488	Eomesodermin	T-box brain protein 2 (TBR2)	Transcriptional activator critical for development; involved in $CD8^+$ T cell differentiation
Gata3	0.763	GATA binding protein 3		Transcriptional activator that binds to the enhancer of the TCR α and δ genes; required for T_H2 differentiation following immune and inflammatory responses

Gzmb	1.2	Granzyme B	Fragmentin 2	Abundant protease in the cytosolic granules of cytotoxic T and NK cells that activates caspase-mediated cell death when delivered into the target cell through the immunological synapse
Gzmk	1.07	Granzyme K	Tryptase II	Granule-secreted, pro-apoptotic serine protease found in the cytoplasmic granules of CTLs
Icos	0.984	Inducible T cell costimulator	CD278	Enhances all basic T cell responses to foreign Ag; essential both for efficient interaction between T and B cells and for normal Ab responses to T cell-dependent Ags
Ikzf1	0.994	IKAROS family zinc finger 1		Transcriptional regulator of hematopoietic cell differentiation; plays a role in T and B cell development
Il12rb2	0.612	Interleukin 12 receptor subunit beta 2		Signaling component coupling to the JAK2/STAT4 pathway; promotes T and NK cell proliferation; promotes T_H1 polarization by enhancing IFNγ production
Itk	0.932	Interleukin-2-inducible T cell kinase	LYK	Key actor in the TCR signaling cascade; phosphorylates PLCγ1, LAT, and LCP2

Lck	0.983	Lymphocyte cell kinase		Src family tyrosine kinase that acts as one of the main signaling intermediaries downstream of the TCR; constitutively associated with the cytoplasmic portion of CD4
Lcp1	0.504	Lymphocyte cytosolic protein 1	Plastin-2	Actin-binding protein that promotes T cell activation in response to costimulation through TCR/CD3 and CD2 or CD28; assists with IL2RA transport to the cell surface
Nfatc1	0.435	Nuclear factor of activated T cells, cytoplasmic 1		Inducible nuclear component of the NFAT TF complex; mediates induction of IL-2 and IL-4 in T cells
Nfatc2	0.602	Nuclear factor of activated T cells, cytoplasmic 2		Cytosolic component of the NFAT TF complex; mediates induction of IL-2, IL-3, IL-4, TNFα, and GM-CSF
Pik3cd	0.741	Phosphatidylinositol-4,5-bisphosphate 3-kinase catalytic subunit delta isoform		A subunit of PI3K; acts downstream of TLR4, TCR, BCR, and CD40; contributes to T helper cell expansion, mast cell development, and neutrophil chemotaxis, extravasation, and respiratory burst
Pik3cg	0.387	Phosphatidylinositol-4,5-bisphosphate 3-kinase catalytic		A subunit of PI3K; modulates leukocyte chemotaxis to inflammatory sites and in response to chemoattractant agents

		subunit gamma isoform		
Ptprc	0.937	Protein tyrosine phosphatase receptor type C	CD45; leukocyte common antigen (LCA)	Delivers costimulation during T cell activation upon binding to its ligand DPP4; dephosphorylates Lyn and suppresses JAK kinases
Spn	0.812	Sialophorin	Leukosialin; CD43	Cell surface sialoglycoprotein expressed by T cells, B cells, monocytes, and granulocytes; promotes lymph node localization in T cells; shunts T cells away from the T_H2 phenotype and towards T_H1; promotes the expression of IFNγ in $CD4^+$ T cells
Tbx21	0.624	T-box transcription factor 21		Initiates T_H1 lineage development from naïve T_H precursor cells both by activating T_H1 genetic programs and by repressing the opposing T_H2 and T_H17 genetic programs
Tcf7	1.24	Transcription factor 7		HMG box TF predominantly expressed by T cells that drives their development, although also involved in NK cell development; activates transcription through a Wnt/β-catenin signaling pathway

Txk	1.07	TXK tyrosine kinase		Regulates the development, function, and differentiation of conventional T cells and nonconventional NK-T cells; contributes to signaling from many receptors and participates in multiple downstream pathways, including regulation of the actin cytoskeleton; can phosphorylate PLCγ1, leading to its localization in lipid rafts and activation, followed by subsequent cleavage of its substrates
Txnip	0.364	Thioredoxin interacting protein		Thiol-oxidoreductase; protects cells from oxidative stress by inhibiting thioredoxin
Zap70	0.772	Zeta chain of T cell receptor associated protein kinase 70		Tyrosine kinase that plays an essential role in regulation of the adaptive immune response; regulates motility, adhesion, proliferation, differentiation, and cytokine expression in T cells; contributes to the development and activation of primary B cells; phosphorylates at least two essential adapter proteins: LAT and LCP2, leading in turn to a large number of signaling molecules being recruited
**Transcription Factors & Coactivators**				

Foxp3	1.02	Forkhead box P3	DIETER	Master TF for Tregs; represses expression of Il2 and Ifng; activates expression of Tnfrsf18, Il2ra, and Ctla4
Eomes	0.488	Eomesodermin	T-box brain protein 2 (TBR2)	Transcriptional activator critical for development; involved in $CD8^+$ T cell differentiation
Gata3	0.763	GATA binding protein 3		Transcriptional activator that binds to the enhancer of the TCR α and δ genes; required for T_H2 differentiation following immune and inflammatory responses
Irf1	0.693	Interferon regulatory factor 1		Transcriptional regulator that promotes inflammatory innate and adaptive immune responses
Irf4	0.927	Interferon regulatory factor 4		Transcriptional activator that complexes with BATF and binds ISREs within the promoters of multiple genes involved in inflammation
Ikzf1	0.994	IKAROS family zinc finger 1		Transcriptional regulator of hematopoietic cell differentiation; plays a role in T and B cell development
Ikzf2	0.383	IKAROS family zinc finger protein 2		Hematopoietic cell-specific TF involved in early hematopoietic development

Nfatc1	0.435	Nuclear factor of activated T cells, cytoplasmic 1		Inducible nuclear component of the NFAT TF complex; mediates induction of IL-2 and IL-4 in T cells
Nfatc2	0.602	Nuclear factor of activated T cells, cytoplasmic 2		Cytosolic component of the NFAT TF complex; mediates induction of IL-2, IL-3, IL-4, TNFα, and GM-CSF
Stat1	0.675	Signal transducer and activator of transcription 1		Transcriptional activator that mediates cellular responses to IFNs, cytokines, and other growth factors
Stat6	0.187	Signal transducer and activator of transcription 6		Essential TF for T_H2 $CD4^+$ T cell and macrophage function and M2 macrophage polarization
Tbx21	0.624	T-box transcription factor 21		Initiates T_H1 lineage development from naïve T_H precursor cells both by activating T_H1 genetic programs and by repressing the opposing T_H2 and T_H17 genetic programs
Tcf7	1.24	Transcription factor 7		HMG box TF predominantly expressed by T cells that drives their development, although also involved in NK cell development; activates transcription through a Wnt/β-catenin signaling pathway
Yy1	0.0857	Yin yang 1		Ubiquitous factor that serves as a transcriptional "switch", either promoting

				or repressing the transcription of numerous genes through the selective recruitment of either histone deacetylases or acetyltransferases; plays a fundamental role in diverse processes, such as differentiation, replication, and cellular proliferation
Zbp1	0.822	Z-DNA binding protein 1	Tumor stroma and activated macrophage protein DLM-1	Key innate sensor that recognizes and binds Z-RNA structures, which are produced by a number of viruses, and induces type-I IFN production; key activator of cellular necroptosis; promotes IL-1α induction in an NLRP3-inflammasome-independent manner
**Ubiquitin Regulation**				
Bcl10	0.137	B cell lymphoma/leukemia 10		Activates NFκB via ubiquitination of IKKγ
Cyld	0.305	Cylindromatosis lysine 63 deubiquitinase		Inhibits NFκB activation by deubiquitinating upstream signaling factors; inhibits Wnt signaling; restricts polyubiquitination of RIPK1 and -2, thereby limiting necroptosis
Tnfaip3	0.387	Tumor necrosis factor, alpha-induced protein 3		Ubiquitin-editing enzyme that complexes with ITCH to degrade inflammatory signaling components in the TNF, IL1, and

				TLR pathways; targets TRAF2, TRAF6, and IKK

NBTXR3+PBT+αPD1 vs PBT+αPD1				
**Gene**	**Log2 fold change**	**Full Name**	**Notable Aliases**	**Function**
**Acute Phase Response**				
Cma1	0.453	Chymase 1	Mast cell protease 1	Serine protease with pro-inflammatory peptidolytic activation predominantly secreted by mast cells
Dpp4	0.585	Dipeptidyl-peptidase 4	CD26	Serine exopeptidase that cleaves various substrates, thereby inactivating them
Cfd	1.33	Complement factor D	Adipsin	Chymotrypsin-family peptidase that cleaves factor B when the latter is complexed with factor C3b, activating C3 convertase
**Adhesion & Cell-Cell Interactions**				
Sell	0.962	L-selectin		Mediates cell adhesion by binding to glycoproteins on neighboring cells
**Antigen Processing & Presentation**				

Cd1d1	0.7	Cluster of differentiation 1 D1		Murine non-classical class I MHC; primarily presents lipid and glycolipid Ags
**Apoptosis**				
Tnf	1.12	Tumor necrosis factor	Cachectin	Inflammatory cytokine mainly produced by macrophages; binds to TNFRSF1A/TNFR1 and TNFRSF1B/TNFBR; capable of inducing cell death in certain tumor cell lines
Nlrp3	0.865	NACHT domain-, leucine-rich repeat-, and PYD-containing protein 3	Cryopyrin	PRR with a wide diversity of recognized targets that activates the NLRP3 inflammasome consisting of NLRP3, PYCARD, and caspase-1/-8
**B Cell-associated Genes**				
Blnk	1.02	B cell linker	Src homology 1 domain-containing leukocyte protein of 65 kDa (SLP-65); Ly57	Functions as a central linker protein downstream of the B cell receptor, bridging SYK kinase to a multitude of signaling pathways and regulating biological outcomes of B cell function and development; plays a role in the activation of ERK/EPHB2, MAP kinase p38 and JNK; modulates AP1 activation; important for the activation of NFκB and NFAT
Btk	0.885	Bruton’s tyrosine		Crucial kinase in B cell receptor signal

		kinase		transmission and B cell activation
Btla	1.68	B and T lymphocyte attenuator	CD272	Inhibitory cell surface protein that inhibits T cell function by binding to B7H4 and TNFRSF14
Cd19	2.14	Cluster of differentiation 19	B-lymphocyte surface antigen B4	BCR coreceptor; activates signaling pathways that lead to the activation of PI3K and Ca(2+) flux
Cd22	1.67	Cluster of differentiation 22	Sialic acid-binding Ig-like lectin 2 (SIGLEC2)<br><br>B lymphocyte cell adhesion molecule (BL-CAM)	Mediates interactions between B cells; binds CD45
Cd40lg	1.48	CD40 ligand	CD154; TNF-related activation protein (TRAP)<br><br>T-B cell activating molecule (T-BAM)	Costimulates T cell proliferation and cytokine production; enhances IL-4 and IL-10 production in conjunction with TCR/CD3 ligation and CD28 costimulation; induces NFκB activation; mediates B cell proliferation in the absence of costimulation; ligand for integrins ITGA5:ITGB1 and ITGAV:ITGB3
Cd69	0.867	Cluster of differentiation 69	C-type lectin domain family 2, member C	Signal transmitting receptor in lymphocytes, NK cells, and platelets; induced upon T cell

				activation; involved in lymphocyte proliferation
Cd79b	2.56	Cluster of differentiation 79b	B29	One of the two flanking proteins that initiate signaling downstream of the BCR
Icosl	0.897	Inducible T cell costimulator ligand	CD275	Ligand for T cell-specific co-receptor ICOS; also induces B cell proliferation and plasma cell differentiation
Ikzf1	1.05	IKAROS family zinc finger 1		Transcriptional regulator of hematopoietic cell differentiation; plays a role in T and B cell development
Ms4a1	2.07	Membrane spanning 4-domains A1	CD20; Bp35	B cell-specific membrane protein that functions as a store-operated Ca(2+) channel component, promoting Ca(2+) influx after BCR activation
Pou2f2	1.12	POU domain class 2, transcription factor 2		TF that regulates Ab and IL-6 expression in B cells
Tnfrsf13c	2.38	TNF receptor superfamily member 13C		B cell-activating factor; enhances B cell survival in vitro; regulator of the peripheral B cell population
**Costimulation**				

Cd40lg	1.48	CD40 ligand	CD154; TNF-related activation protein (TRAP)<br><br>T-B cell activating molecule (T-BAM)	Costimulates T cell proliferation and cytokine production; enhances IL-4 and IL-10 production in conjunction with TCR/CD3 ligation and CD28 costimulation; induces NFκB activation; mediates B cell proliferation in the absence of costimulation; ligand for integrins ITGA5:ITGB1 and ITGAV:ITGB3
Icosl	0.897	Inducible T cell costimulator ligand	CD275	Ligand for T cell-specific co-receptor ICOS; also induces B cell proliferation and plasma cell differentiation
**Cytokines**				
Il1b	0.765	Interleukin 1 beta	Catabolin	One of the two primary inflammatory cytokines produced by the inflammasome (the other one being IL-18); induces neutrophil influx and activation, T cell activation and cytokine production, B cell activation and Ab production, fibroblast proliferation, and collagen production; synergizes with IL-12 to induce IFNγ synthesis from T_H1 cells
Il7r	1.02	Interleukin 7 receptor	CD127	Receptor for IL-7

Tnf	1.12	Tumor necrosis factor	Cachectin	Inflammatory cytokine mainly produced by macrophages; binds to TNFRSF1A/TNFR1 and TNFRSF1B/TNFBR; capable of inducing cell death in certain tumor cell lines
**Growth/Proliferation**				
Rps6	0.606	Ribosomal protein 6		Component of the 40S small ribosomal subunit; plays an important role in controlling cell growth and proliferation through the selective translation of particular classes of mRNA
Tyk2	0.74	Tyrosine kinase 2	JTK1	Plays both structural and catalytic roles in numerous cytokines and interferons signaling; associates with cytokine and growth factor receptors and activate STAT family members including STAT1, STAT3, STAT4, or STAT6
**Inflammation**				
Il1b	0.765	Interleukin 1 beta	Catabolin	One of the two primary inflammatory cytokines produced by the inflammasome (the other one being IL-18); induces neutrophil influx and activation,T cell activation and cytokine production, B cell activation and Ab production, fibroblast

				proliferation, and collagen production; synergizes with IL-12 to induce IFNγ synthesis from T_H1 cells
Irf4	0.828	Interferon regulatory factor 4		Transcriptional activator that complexes with BATF and binds ISREs within the promoters of multiple genes involved in inflammation
Nlrp3	0.865	NACHT domain-, leucine-rich repeat-, and PYD-containing protein 3	Cryopyrin	PRR with a wide diversity of recognized targets that activates the NLRP3 inflammasome consisting of NLRP3, PYCARD, and caspase-1/-8
Tnf	1.12	Tumor necrosis factor	Cachectin	Inflammatory cytokine mainly produced by macrophages; binds to TNFRSF1A/TNFR1 and TNFRSF1B/TNFBR; capable of inducing cell death in certain tumor cell lines
		**Inhibition**		
Btla	1.68	B and T lymphocyte attenuator	CD272	Inhibitory cell surface protein that inhibits T cell function by binding to B7H4 and TNFRSF14
Foxp3	0.544	Forkhead box P3	DIETER	Master TF for Tregs; represses expression of Il2 and Ifng; activates expression of Tnfrsf18, Il2ra, and Ctla4

Nfkbia	0.442	Nuclear factor kappa B inhibitor alpha		Inhibits activity of REL dimers by masking of their nuclear localization signals
Sh2b2	0.6	Src homology 2B adaptor protein 2		Adapter protein for several members of the tyrosine kinase receptor family; involved in multiple signaling pathways; may be involved in coupling from immunoreceptor to Ras signaling; acts as a negative regulator of cytokine signaling in collaboration with CBL; may induce cytoskeletal reorganization via interaction with Vav3
**Kinases**				
Btk	0.885	Bruton's tyrosine kinase		Crucial kinase in B cell receptor signal transmission and B cell activation
Txk	0.93	TXK tyrosine kinase		Regulates the development, function, and differentiation of conventional T cells and nonconventional NK-T cells; contributes to signaling from many receptors and participates in multiple downstream pathways, including regulation of the actin cytoskeleton; can phosphorylate PLCγ1, leading to its localization in lipid rafts and activation, followed by subsequent cleavage

				of its substrates
Tyk2	0.74	Tyrosine kinase 2	JTK1	Plays both structural and catalytic roles in numerous cytokines and interferons signaling; associates with cytokine and growth factor receptors and activate STAT family members including STAT1, STAT3, STAT4, or STAT6
**Macrophage Function**				
Marco	1.37	Macrophage receptor with collagenous structure		A PRR that recognizes LDL
Slc11a1	0.667	Natural resistance-associated macrophage protein 1		Macrophage-specific metal ion transporter; uptakes divalent metal cations to neutralize ROSs
**Metabolism**				
Abcg1	1.05	ATP-binding cassette transporter G1		Membrane-associated cholesterol efflux pump
**Migration/Motility**				

Ccl3	0.512	C-C motif chemokine ligand 3	Macrophage inflammatory protein 1α (MIP1α)	Chemoattractant ligand for CCR1, -4, and -5
Ccr2	0.694	C-C motif chemokine receptor 2	CD192	Receptor for CCL2, a monocyte-specific chemokine
Ccr6	1.74	C-C motif chemokine receptor 6	CD196	Receptor for CCL20 and β-defensins; signals through Ca(2+) flux; controls chemotaxis of DCs and effector/memory T cells and B cells; essential for recruitment of T_H17 cells and Tregs
Cxcr5	1.83	C-X-C motif chemokine receptor 5	CD185; Burkitt's lymphoma receptor 1 (BLR1)	Cytokine receptor that binds to B lymphocyte chemoattractant (BLC); involved in B cell migration into splenic follicles and Peyer's patches
**NFκB Signaling**				
Card11	1.33	Caspase recruitment domain family member 11	Bcl10-interacting MAGUK protein 3 (BIMP3)	Adapter protein that plays a key role in adaptive immune response by transducing the activation of NFκB downstream of TCR and BCR engagement; upon activation in response to TCR or BCR triggering, homooligomerizes to form a nucleating helical template that recruits BCL10 via

				CARD-CARD interaction, thereby promoting polymerization of BCL10 and subsequent recruitment of MALT1, leading to IKK phosphorylation and degradation and release of NFκB proteins for nuclear translocation
Ikbke	0.776	Inhibitor of nuclear factor kappa B kinase subunit epsilon		Noncanonical IκB kinase; plays an essential role in regulating inflammatory through the activation of the type I IFN, NFκB, and STAT signaling
**Pattern Recognition Receptors**				
Cd180	1.33	Cluster of differentiation 180		Heterodimeric binding partner of Ly86 that participates in LPS binding in APCs
Marco	1.37	Macrophage receptor with collagenous structure		A PRR that recognizes LDL
Nlrp3	0.865	NACHT domain-, leucine-rich repeat-, and PYD-containing protein 3	Cryopyrin	PRR with a wide diversity of recognized targets that activates the NLRP3 inflammasome consisting of NLRP3, PYCARD, and caspase-1/-8
Tlr7	0.876	Toll-like receptor 7	CD287	Endosomic PRR that recognizes ssRNA

T Cell Function				
Cd69	0.867	Cluster of differentiation 69	C-type lectin domain family 2, member C	Signal transmitting receptor in lymphocytes, NK cells, and platelets; induced upon T cell activation; involved in lymphocyte proliferation
Ikzf1	1.05	IKAROS family zinc finger 1		Transcriptional regulator of hematopoietic cell differentiation; plays a role in T and B cell development
Txk	0.93	TXK tyrosine kinase		Regulates the development, function, and differentiation of conventional T cells and nonconventional NK-T cells; contributes to signaling from many receptors and participates in multiple downstream pathways, including regulation of the actin cytoskeleton; can phosphorylate PLCγ1, leading to its localization in lipid rafts and activation, followed by subsequent cleavage of its substrates

Supplemental Table 3: Differentially expressed genes in various immune cells

Markers	Cluster	Markers	Cluster	Markers	Cluster
Mrc1	Macrophages	Ms4a4c	Monocytes	Cd8a	Cytotoxic T cells
Sdc3	Macrophages	Ms4a6c	Monocytes	Cd8b1	Cytotoxic T cells
Msr1	Macrophages	Plac8	Monocytes	Lag3	Cytotoxic T cells
Lrp1	Macrophages	Tgfbi	Monocytes	Nkg7	Cytotoxic T cells

Cxcl16	Macrophages	Ms4a4a	Monocytes	Gzmb	Cytotoxic T cells
Grn	Macrophages	Gm9733	Monocytes	Gzmk	Cytotoxic T cells
Lgmn	Macrophages	Plbd1	Monocytes	Klrc1	Cytotoxic T cells
Dab2	Macrophages	Ifitm3	Monocytes	Prf1	Cytotoxic T cells
Trem2	Macrophages	Lrp1	Monocytes	Cxcr6	Cytotoxic T cells
Csf1r	Macrophages	H2-DMb1	Monocytes	Pdcd1	Cytotoxic T cells
Cd4	CD4+ T cells	Cd8a	CD8+ T cells	Klra4	NK cells
Tcf7	CD4+ T cells	Cd8b1	CD8+ T cells	Ncr1	NK cells
Nsg2	CD4+ T cells	Lat	CD8+ T cells	Klra7	NK cells
S1pr1	CD4+ T cells	Fcer1g	CD8+ T cells	Klra1	NK cells
Ccr7	CD4+ T cells	Rps7	CD8+ T cells	Trdc	NK cells
Tnfsf8	CD4+ T cells	Rps5	CD8+ T cells	Gzma	NK cells
Tbc1d4	CD4+ T cells	Rpsa	CD8+ T cells	Car2	NK cells
Ets1	CD4+ T cells	Lck	CD8+ T cells	Nrarp	NK cells
Lrig1	CD4+ T cells	Rpl30	CD8+ T cells	Klrb1f	NK cells
Lck	CD4+ T cells	Rps15a	CD8+ T cells	Atp1b1	NK cells
Rpl7	Neutrophils	Flt3	DC	Kif11	Tgd
Cxcr2	Neutrophils	3-Sep	DC	Cd8b1	Tgd
G0s2	Neutrophils	Clec9a	DC	Cd8a	Tgd
Rpl7a	Neutrophils	Gcsam	DC	Cd3e	Tgd
Hdc	Neutrophils	Mycl	DC	Lat	Tgd
Cebpb	Neutrophils	Tspan33	DC	Thy1	Tgd
Rps3a1	Neutrophils	Timd4	DC	Cd3d	Tgd
S100a8	Neutrophils	Cbfa2t3	DC	Trbc2	Tgd
S100a9	Neutrophils	Hmgn3	DC	1-Sep	Tgd
Mcl1	Neutrophils	Gnb4	DC	Cd3g	Tgd
Rps20	Neutrophils	Nkg7	ILC	Foxp3	Tregs
Rps8	Neutrophils	Klrd1	ILC	Cd4	Tregs
Csf3r	Neutrophils	Il2rb	ILC	Rln3	Tregs
Rpl11	Neutrophils	Klrc1	ILC	Izumo1r	Tregs
Cxcl2	Neutrophils	Ctsw	ILC	Tnfrsf4	Tregs
Rplp0	Neutrophils	Ctla2a	ILC	Ikzf2	Tregs
Rpl6	Neutrophils	Thy1	ILC	Tnfrsf18	Tregs
Tmsb10	Neutrophils	Klrk1	ILC	Cd2	Tregs
Rpl32	Neutrophils	Ms4a4b	ILC	Igsf23	Tregs
Rpl28	Neutrophils	Klre1	ILC	Neb	Tregs
Rps5	Neutrophils	Ccr7	CD4-CD8- T cells	Cd79a	B cells
Rps19	Neutrophils	S1pr1	CD4-CD8- T cells	Ebf1	B cells
Rps11	Neutrophils	Tcf7	CD4-CD8- T cells	Ly6d	B cells
Rpl18	Neutrophils	Lat	CD4-CD8- T cells	Iglc3	B cells
Rps3	Neutrophils	Ets1	CD4-CD8- T cells	Iglc2	B cells

Rpl27a	Neutrophils	Emilin2	CD4-CD8- T cells	Mzb1	B cells
Rps15a	Neutrophils	Plek	CD4-CD8- T cells	Cd79b	B cells
Rpl13	Neutrophils	Lgals3	CD4-CD8- T cells	Spib	B cells
Eef1a1	Neutrophils	Ifitm2	CD4-CD8- T cells	Igkc	B cells
Rpsa	Neutrophils	Ctsd	CD4-CD8- T cells	Snn	B cells
Rpl14	Neutrophils	Cd3e	NKT	Fcer1a	Mast cells
Srgn	Neutrophils	Cd3d	NKT	Cpa3	Mast cells
Eef2	Neutrophils	Cd3g	NKT	Car8	Mast cells
Ppia	Neutrophils	Icos	NKT	Atp8b5	Mast cells
Rack1	Neutrophils	Trac	NKT	Gata2	Mast cells
Rpl23	Neutrophils	Ctla4	NKT	Tph1	Mast cells
Rpl19	Neutrophils	Cd2	NKT	Cyp11a1	Mast cells
Rps7	Neutrophils	Ptprcap	NKT	Cobl	Mast cells
Rps23	Neutrophils	Cd6	NKT	Cd200r3	Mast cells
Rpl15	Neutrophils	H2-Q7	NKT	Maob	Mast cells
Rps24	Neutrophils				
Rpl8	Neutrophils				
Rpl3	Neutrophils				
Rps2	Neutrophils				
Rps10	Neutrophils				
Rpl10a	Neutrophils				
Rps18	Neutrophils				
Hsp90ab1	Neutrophils				
Rps4x	Neutrophils				
mt-Co1	Neutrophils				
mt-Co2	Neutrophils				
mt-Atp6	Neutrophils				
mt-Co3	Neutrophils				
mt-Cytb	Neutrophils				

Supplemental Table 4: Genes Differentially Upregulated in Survivor Mice Prior to Rechallenge

Gene	Log2 fold change	Full Name	Notable Aliases	Function

**Acute Phase Response**				
App	2.46	Amyloid-beta precursor protein		Cell surface receptor and transmembrane precursor protein that is cleaved by secretases to form a number of peptides; involved in cell mobility, copper homeostasis, and oxidative stress
Clu	1.62	Clusterin	Apolipoprotein J; Ku70-binding protein 1 (KUB1)	Extracellular chaperone that promotes clearance of inflammation and injury-induced immune complexes; protects cells against apoptosis and against cytolysis by complement
Lcn2	2.98	Lipocalin 2	Neutrophil gelatinase-associated lipocalin (NGAL)	Neutrophil-secreted factor that sequesters iron-containing siderophores; also functions as a growth factor
**Adhesion & Cell-Cell Interactions**				
Cd97	1.35	Cluster of differentiation 97	BL-Ac[F2]	GPCR that promotes granulocyte adhesion and migration; activates T cells via binding to CD55; stimulates angiogenesis through binding integrin counterreceptors on endothelial cells

Ifih1	1.09	Interferon induced with helicase C domain 1	Helicard; melanoma differentiation-associated protein 5 (MDA5)	PRR for cytoplasmic dsRNA; upon target recognition, associates with MAVS to activate TNK1 and IKBKE, which phosphorylate IRF3 and -7, which, in turn, activate transcription of IFNα and -β
Itgam	1.77	Integrin alpha M	CD11b	Pairs with CD18 to forms Mac-1 aka complement receptor 3; mediates leukocyte activation, adhesion, chemotaxis, migration, phagocytosis, and cell-mediated cytotoxicity; serves as a macrophage marker
Lgals3	1.99	Galectin 3		Galactose-specific lectin that binds IgE; involved in acute inflammatory responses, including neutrophil activation and adhesion, chemoattraction of monocytes macrophages, opsonization of apoptotic neutrophils, and activation of mast cells
Map2k1	0.824	Dual specificity mitogen-activated protein kinase kinase 1	MAPK/ERK kinase 1 (MEK1)	Essential component of the MAP kinase signal transduction pathway; participates in numerous biological functions, including cell growth, adhesion, survival, differentiation, transcription, metabolism, and cytoskeletal remodeling

S100a8	2.82	S100 calcium-binding protein A8	Calgranulin A	Calcium- and zinc-binding protein involved in pro-inflammatory, antimicrobial, oxidant-scavenging and apoptosis-inducing activities; can induce neutrophil chemotaxis, adhesion, phagocytosis, and degranulation; predominantly found as calprotectin (S100A8/A9) which has a wide plethora of intra- and extracellular functions, including adhesion, apoptosis, autophagy, cytoskeletal remodeling, cytokine production, chemotaxis, migration, inflammation, arachidonic acid metabolism, oxidant-scavenging, and PRR signaling
Thbs1	1.44	Thrombospondin 1		Adhesive glycoprotein that mediates cell-to-cell and cell-to-matrix interactions; ligand for CD36
**Angiogenesis**				
Angpt1	1.72	Angiopoietin 1		Secreted glycoprotein that binds and activates the TEK/TIE2 receptor by inducing its dimerization and tyrosine phosphorylation; involved in angiogenesis, endothelial cell survival, proliferation,

				migration, adhesion, cell spreading, and reorganization of the actin cytoskeleton
Cd97	1.35	Cluster of differentiation 97	BL-Ac[F2]	GPCR that promotes granulocyte adhesion and migration; activates T cells via binding to CD55; stimulates angiogenesis through binding integrin counterreceptors on endothelial cells
Il1b	2.64	Interleukin 1 beta	Catabolin	One of the two primary inflammatory cytokines produced by the inflammasome (the other one being IL-18); induces neutrophil influx and activation,T cell activation and cytokine production, B cell activation and Ab production, fibroblast proliferation, and collagen production; synergizes with IL-12 to induce IFNγ synthesis from T_H1 cells
**Antigen Processing & Presentation**				
Cd74	0.853	Cluster of differentiation 74	MHC class II gamma chain	Stabilizes peptide-free class II αβ heterodimers during MHC-Ag complex formation
Clec4a2	2.18	C-type lectin domain family 4 member A2		PRR that, upon binding mannose or fucose, is endocytosed and processed in the Ag presentation pathway

Ctss	1.42	Cathepsin S		Lysosomal protease that participates in processing of Ag by MHC class II
H2-Q10	1.98	Histocompatibility 2, Q region locus 10		MHC class I molecule; presents Ags to $CD8^+$ T cells
Icam1	1.22	Intracellular adhesion molecule 1	CD54	Cell surface glycoprotein that serves as strong adhesive ligand for LFA-1; important for leukocyte mobility and costimulation
**Anti-Inflammatory**				
Bcl2	1.12	B cell lymphoma 2		Outer mitochondrial membrane protein that inhibits apoptosis and autophagy; may attenuate inflammation by impairing inflammasome formation
Clu	1.62	Clusterin	Apolipoprotein J; Ku70-binding protein 1 (KUB1)	Extracellular chaperone that promotes clearance of inflammation and injury-induced immune complexes; protects cells against apoptosis and against cytolysis by complement
Tnfaip3	1.38	Tumor necrosis factor, alpha-induced protein 3		Ubiquitin-editing enzyme that complexes with ITCH to degrade inflammatory signaling components in the TNF, IL1, and TLR pathways; targets TRAF2, TRAF6, and IKK

Tollip	1.32	Toll interacting protein		Inhibitory adaptor protein; recruits IRAK1 to the IL-1 receptor complex and inhibitively phosphorylates it
**Apoptosis**				
Bid	1.4	BH3 interacting domain death agonist	Desmocollin type 4, apoptic death agonist	Induces caspases and apoptosis; counters the protective effect of BCL2, allowing release of cytochrome C
Casp1	1.66	Caspase 1	Interleukin 1β convertase	Cysteine-aspartic acid protease that mediates cleavage-based activation of IL-1β and IL-18; serves as the central enzymatic core of the inflammasome; also induces apoptosis
Casp8	0.914	Caspase 8		Cysteine-aspartic acid protease; cleaves and activates effector caspases CASP3, -4, -6, -7, -9, and -10; initiates pyroptosis through cleavage of gasdermin-D; mediates noncanonical cleavage of IL-1β in DCs and macrophages
Ifitm2	2.63	Interferon-induced transmembrane protein 2		IFN-induced antiviral protein which inhibits the entry of viruses to the host cell cytoplasm; induces cell cycle arrest and mediates p53-independent apoptosis through caspase activation

S100a8	2.82	S100 calcium-binding protein A8	Calgranulin A	Calcium- and zinc-binding protein involved in pro-inflammatory, antimicrobial, oxidant-scavenging and apoptosis-inducing activities; can induce neutrophil chemotaxis, adhesion, phagocytosis, and degranulation; predominantly found as calprotectin (S100A8/A9) which has a wide plethora of intra- and extracellular functions, including adhesion, apoptosis, autophagy, cytoskeletal remodeling, cytokine production, chemotaxis, migration, inflammation, arachidonic acid metabolism, oxidant-scavenging, and PRR signaling
**Autophagy**				
Atg5	1.26	Autophagy related 5		Pairs with ATG12 to promote the extension of the phagophoric membrane in autophagic vesicles
Irgm2	1.39	Immunity-related GTPase family M member 2	Interferon-inducible protein 1 (IFI1)	Function not fully known, but most likely regulates autophagy and pro-inflammatory cytokine production
S100a8	2.82	S100 calcium-binding protein A8	Calgranulin A	Calcium- and zinc-binding protein involved in pro-inflammatory, antimicrobial, oxidant-scavenging and apoptosis-inducing

				activities; can induce neutrophil chemotaxis, adhesion, phagocytosis, and degranulation; predominantly found as calprotectin (S100A8/A9) which has a wide plethora of intra- and extracellular functions, including adhesion, apoptosis, autophagy, cytoskeletal remodeling, cytokine production, chemotaxis, migration, inflammation, arachidonic acid metabolism, oxidant-scavenging, and PRR signaling
Ubc	0.99	Polyubiquitin C		Serves various roles, including immate immunity, DNA repair, and stimulation of autophagy and the proteasomal response
**B Cell-associated Genes**				
Lyn	1.88	Lck/Yes-related novel kinase		Src family tyrosine kinase that potentiates signaling from the B cell receptor and CD40
Pik3cg	1.44	Phosphatidylinositol-4,5-bisphosphate 3-kinase catalytic		A subunit of PI3K; modulates leukocyte chemotaxis to inflammatory sites and in response to chemoattractant agents

		subunit gamma isoform		
**Chemotaxis**				
Ccl3	1.01	C-C motif chemokine ligand 3	Macrophage inflammatory protein 1α (MIP1α)	Chemoattractant ligand for CCR1, -4, and -5
Ccr1	1.85	C-C motif chemokine receptor 1	MIP1α receptor	Receptor for CCL3, -5, -7, and -23
Ccr2	2.01	C-C motif chemokine receptor 2	CD192	Receptor for CCL2, a monocyte-specific chemokine
Ccr3	-0.901	C-C motif chemokine receptor 3	CD193	Receptor for a variety of chemokines, including CCL11, CCL26, CCL7, CCL13, CCL5 (RANTES), and CCL15; signals through Ca(2+) flux
Ccr5	1.18	C-C motif chemokine receptor 5	CD195	Receptor for a number of inflammatory CC-chemokines, including CCL3/MIP1α, CCL4/MIP1β, and RANTES; signals via Ca(2+) flux
Cklf	1.14	Chemokine-like factor		Chemoattractant for monocytes, neutrophils, and lymphocytes

Cxcr2	2.38	C-X-C motif chemokine receptor 2	CD182; IL-8 receptor B	Receptor for IL-8 and CXCL3; powerful chemoattractant for neutrophils
Cxcr3	1.55	C-X-C motif chemokine receptor 3	CD183	Induces integrin activation, cytoskeletal remodeling, and chemotaxis; expressed by T cells and NK cells; prominently expressed in effector and memory T cells
Itgam	1.77	Integrin alpha M	CD11b	Pairs with CD18 to forms Mac-1 aka complement receptor 3; mediates leukocyte activation, adhesion, chemotaxis, migration, phagocytosis, and cell-mediated cytotoxicity; serves as a macrophage marker
Lgals3	1.99	Galectin 3		Galactose-specific lectin that binds IgE; involved in acute inflammatory responses, including neutrophil activation and adhesion, chemoattraction of monocytes macrophages, opsonization of apoptotic neutrophils, and activation of mast cells
S100a8	2.82	S100 calcium-binding protein A8	Calgranulin A	Calcium- and zinc-binding protein involved in pro-inflammatory, antimicrobial, oxidant-scavenging and apoptosis-inducing activities; can induce neutrophil chemotaxis, adhesion, phagocytosis, and

				degranulation; predominantly found as calprotectin (S100A8/A9) which has a wide plethora of intra- and extracellular functions, including adhesion, apoptosis, autophagy, cytoskeletal remodeling, cytokine production, chemotaxis, migration, inflammation, arachidonic acid metabolism, oxidant-scavenging, and PRR signaling
**Chromatin Remodeling**				
Ep300	1.1	Adenovirus early region 1A-associated protein p300		Histone acetyltransferase; participates in chromatin remodeling to facilitate gene accessibility
Hmgb1	1.19	High-mobility group box 1		Remodels chromatin to make DNA more available for transcription
**Complement & Humoral Immunity**				
C3	2	Complement component 3		Cleaved by C3 convertase to form C3a and C3b, an anaphalotoxin and an opsonizing agent, respectively

Cfh	2.77	Complement factor H		Soluble glycoprotein that regulates the alternate pathway by accelerating decay of C3 convertase
Cfp	1.82	Complement factor properdin		Alternate complement pathway component; when cleaved, produces a serine protease that binds to C3b to form C3 convertase
Fcer1a	1.7	Fragment crystallizable of immunoglobulin epsilon receptor 1a		High affinity receptor for IgE; responsible for initiating the allergic response
Lgals3	1.99	Galectin 3		Galactose-specific lectin that binds IgE; involved in acute inflammatory responses, including neutrophil activation and adhesion, chemoattraction of monocytes macrophages, opsonization of apoptotic neutrophils, and activation of mast cells
**Costimulation**				
Cd28	1.04	Cluster of differentiation 28		Essential T cell co-receptor that enhances T cell activation, proliferation, cytokine production, and survival; binds to CD80 and CD86

Icam1	1.22	Intracellular adhesion molecule 1	CD54	Cell surface glycoprotein that serves as strong adhesive ligand for LFA-1; important for leukocyte mobility and costimulation
		**Cytokines**		
Csf1	1.75	Macrophage colony-stimulating factor 1		Cytokine that promote activation and survival of monocytes
Csf1r	1.94	Macrophage colony-stimulating factor 1 receptor	CD115	Receptor for CSF1; promotes release of inflammatory cytokines in response to IL-34 and CSF1
Ifnar1	1.12	Interferon-alpha/beta receptor alpha chain		Component of the receptor for type I IFNs, binding of which activates the JAK-STAT pathway
Ifngr1	1.45	Interferon gamma receptor 1	CD119	One of the two components of the IFNγ receptor; stimulates activation of the JAK/STAT signaling pathway
Il1b	2.64	Interleukin 1 beta	Catabolin	One of the two primary inflammatory cytokines produced by the inflammasome (the other one being IL-18); induces neutrophil influx and activation,T cell activation and cytokine production, B cell activation and Ab production, fibroblast

				proliferation, and collagen production; synergizes with IL-12 to induce IFNγ synthesis from T_H1 cells
Il1rap	2.32	Interleukin 1 receptor accessory protein		Co-receptor for several ligands, including IL-1R1 in the IL-1 pathway, IL-RL1 in the IL-33 pathway, IL-1RL2 in the IL-36 pathway; signaling involves Tollip, MyD88, IRAK1, and IRAK2
Il1rl1	1.63	Interleukin 1 receptor-like 1		Receptor for IL-33; recruits MyD88, IRAK1, IRAK4, and TRAF6; activates ERK1, ERK2, and MAPK14
Il6st	0.913	Interleukin 6 cytokine family signal transducer	Glycoprotein 130 (Gp130); CD130	Transmembrane protein that acts a component in several cytokine receptors, including IL-6
Il18	1.29	Interleukin 18	Iboctadekin	One of the two primary inflammatory cytokines produced by the inflammasome (the other one being IL-1β); upon binding to IL18R1 and IL18RAP, forms a signaling ternary complex which activates NFκB, triggering synthesis of inflammatory mediators
Il18r1	1.21	Interleukin 18 receptor 1	CD218a	Receptor for IL-18

Il18rap	1.83	Interleukin 18 receptor accessory protein	CD218b	Accessory subunit of the heterodimeric IL-18 receptor involved in IL18-dependent signal transduction, leading to NFκB and JNK activation
**Cytoskeletal Remodeling**				
Angpt1	1.72	Angiopoietin 1		Secreted glycoprotein that binds and activates the TEK/TIE2 receptor by inducing its dimerization and tyrosine phosphorylation; involved in angiogenesis, endothelial cell survival, proliferation, migration, adhesion, cell spreading, and reorganization of the actin cytoskeleton
Map2k1	0.824	Dual specificity mitogen-activated protein kinase kinase 1	MAPK/ERK kinase 1 (MEK1)	Essential component of the MAP kinase signal transduction pathway; participates in numerous biological functions, including cell growth, adhesion, survival, differentiation, transcription, metabolism, and cytoskeletal remodeling
**Cytotoxicity**				
Itgam	1.77	Integrin alpha M	CD11b	Pairs with CD18 to forms Mac-1 aka complement receptor 3; mediates leukocyte activation, adhesion, chemotaxis, migration, phagocytosis, and cell-mediated

				cytotoxicity; serves as a macrophage marker
S100a8	2.82	S100 calcium-binding protein A8	Calgranulin A	Calcium- and zinc-binding protein involved in pro-inflammatory, antimicrobial, oxidant-scavenging and apoptosis-inducing activities; can induce neutrophil chemotaxis, adhesion, phagocytosis, and degranulation; predominantly found as calprotectin (S100A8/A9) which has a wide plethora of intra- and extracellular functions, including adhesion, apoptosis, autophagy, cytoskeletal remodeling, cytokine production, chemotaxis, migration, inflammation, arachidonic acid metabolism, oxidant-scavenging, and PRR signaling
**Growth/Proliferation**				
Angpt1	1.72	Angiopoietin 1		Secreted glycoprotein that binds and activates the TEK/TIE2 receptor by inducing its dimerization and tyrosine phosphorylation; involved in angiogenesis, endothelial cell survival, proliferation, migration, adhesion, cell spreading, and reorganization of the actin cytoskeleton

Atf2	1.13	Activating transcription factor 2	Cyclic AMP-responsive element-binding protein 2 (CREB2)	Regulates transcription of various genes involved in anti-apoptosis, cell growth, and DNA damage response; in the nucleus, contributes to global transcription and the DNA damage response, in addition to specific transcriptional activities that are related to cell development, proliferation and death; in the cytoplasm, impairs mitochondrial membrane potential, inducing mitochondrial leakage and promoting cell death; phosphorylated form (mediated by ATM) plays a role in the DNA damage response
Map2k1	0.824	Dual specificity mitogen-activated protein kinase kinase 1	MAPK/ERK kinase 1 (MEK1)	Essential component of the MAP kinase signal transduction pathway; participates in numerous biological functions, including cell growth, adhesion, survival, differentiation, transcription, metabolism, and cytoskeletal remodeling
Tyk2	1.54	Tyrosine kinase 2	JTK1	Plays both structural and catalytic roles in numerous cytokines and interferons signaling; associates with cytokine and growth factor receptors and activate STAT

				family members including STAT1, STAT3, STAT4, or STAT6
**Ion Transport**				
App	2.46	Amyloid-beta precursor protein		Cell surface receptor and transmembrane precursor protein that is cleaved by secretases to form a number of peptides; involved in cell mobility, copper homeostasis, and oxidative stress
Slc11a1	2.01	Natural resistance-associated macrophage protein 1		Macrophage-specific metal ion transporter; uptakes divalent metal cations to neutralize ROSs
**Inflammation**				
Bst2	0.822	Bone marrow stromal cell antigen 2	Tethrin; CD317	IFN-induced antiviral factor that blocks budding of enveloped viruses by directly tethering nascent virions to the membranes of infected cells
Casp1	1.66	Caspase 1	Interleukin 1β convertase	Cysteine-aspartic acid protease that mediates cleavage-based activation of IL-1β and IL-18; serves as the central enzymatic core of the inflammasome; also induces apoptosis

Casp8	0.914	Caspase 8		Cysteine-aspartic acid protease; cleaves and activates effector caspases CASP3, -4, -6, -7, -9, and -10; initiates pyroptosis through cleavage of gasdermin-D; mediates noncanonical cleavage of IL-1β in DCs and macrophages
Cebpb	2.13	CCAAT/enhancer-binding protein beta		Critical macrophage TF that promotes expression of several acute-phase and inflammatory cytokine genes, including Il6
Clu	1.62	Clusterin	Apolipoprotein J; Ku70-binding protein 1 (KUB1)	Extracellular chaperone that promotes clearance of inflammation and injury-induced immune complexes; protects cells against apoptosis and against cytolysis by complement
Csf1r	1.94	Macrophage colony-stimulating factor 1 receptor	CD115	Receptor for CSF1; promotes release of inflammatory cytokines in response to IL-34 and CSF1
Ifnar1	1.12	Interferon-alpha/beta receptor alpha chain		Component of the receptor for type I IFNs, binding of which activates the JAK-STAT pathway

Ifngr1	1.45	Interferon gamma receptor 1	CD119	One of the two components of the IFNγ receptor; stimulates activation of the JAK/STAT signaling pathway
Il1b	2.64	Interleukin 1 beta	Catabolin	One of the two primary inflammatory cytokines produced by the inflammasome (the other one being IL-18); induces neutrophil influx and activation,T cell activation and cytokine production, B cell activation and Ab production, fibroblast proliferation, and collagen production; synergizes with IL-12 to induce IFNγ synthesis from T_H1 cells
Il6st	0.913	Interleukin 6 cytokine family signal transducer	Glycoprotein 130 (Gp130); CD130	Transmembrane protein that acts a component in several cytokine receptors, including IL-6
Il18	1.29	Interleukin 18	Iboctadekin	One of the two primary inflammatory cytokines produced by the inflammasome (the other one being IL-1β); upon binding to IL18R1 and IL18RAP, forms a signaling ternary complex which activates NFκB, triggering synthesis of inflammatory mediators

Il18r1	1.21	Interleukin 18 receptor 1	CD218a	Receptor for IL-18
Il18rap	1.83	Interleukin 18 receptor accessory protein	CD218b	Accessory subunit of the heterodimeric IL-18 receptor involved in IL18-dependent signal transduction, leading to NFκB and JNK activation
Irf7	1.22	Interferon regulatory factor 7		Key transcriptional regulator of type I IFN-dependent immune responses; promotes transcription of IFNα and -β
Irgm2	1.39	Immunity-related GTPase family M member 2	Interferon-inducible protein 1 (IFI1)	Function not fully known, but most likely regulates autophagy and pro-inflammatory cytokine production
Lgals3	1.99	Galectin 3		Galactose-specific lectin that binds IgE; involved in acute inflammatory responses, including neutrophil activation and adhesion, chemoattraction of monocytes macrophages, opsonization of apoptotic neutrophils, and activation of mast cells
Lilra5	2.23	Leukocyte immunoglobulin-like receptor A5	CD85	Function poorly understood; may play a role in triggering innate immune responses through triggering Ca(2+) influx and proinflammatory cytokine release

Mavs	0.866	Mitochondrial antiviral signaling protein	IFNβ promoter stimulator protein 1 (ISP-1)	Intermediary protein involved in the nonclassical inflammasome pathway; acts downstream of DDX58 and IFIH1, leading to the activation of NFκB, IRF3, and IRF7, and the subsequent induction of IFNβ and RANTES
Mefv	2.88	Mediterranean fever	Marenostrin; pyrin	Involved in the regulation of innate immunity and the inflammatory response in response to IFNγ; both stimulates and restrains the inflammasome; also acts as a mediator of pyroptosis
Mif	1.07	Macrophage migration inhibitory factor	L-dopachrome tautomerase	Pro-inflammatory cytokine that promotes macrophage function through suppression of anti-inflammatory effects of glucocorticoids
Nlrp3	2.42	NACHT domain-, leucine-rich repeat-, and PYD-containing protein 3	Cryopyrin	PRR with a wide diversity of recognized targets that activates the NLRP3 inflammasome consisting of NLRP3, PYCARD, and caspase-1/-8
Pik3cg	1.44	Phosphatidylinositol-4,5-bisphosphate 3-kinase catalytic		A subunit of PI3K; modulates leukocyte chemotaxis to inflammatory sites and in response to chemoattractant agents

		subunit gamma isoform		
S100a8	2.82	S100 calcium-binding protein A8	Calgranulin A	Calcium- and zinc-binding protein involved in pro-inflammatory, antimicrobial, oxidant-scavenging and apoptosis-inducing activities; can induce neutrophil chemotaxis, adhesion, phagocytosis, and degranulation; predominantly found as calprotectin (S100A8/A9) which has a wide plethora of intra- and extracellular functions, including adhesion, apoptosis, autophagy, cytoskeletal remodeling, cytokine production, chemotaxis, migration, inflammation, arachidonic acid metabolism, oxidant-scavenging, and PRR signaling
Tbk1	1.22	TANK-binding kinase 1		Coordinates the activation of IRF3 and NFκB and induction of type I IFNs
Tmem173	0.811	Transmembrane protein 173	Stimulator of interferon genes (STING)	Adaptor protein in type I IFN signaling; activates STAT6 and IRF3 through TBK1 to induce type I IFN production

Tollip	1.32	Toll interacting protein		Inhibitory adaptor protein; recruits IRAK1 to the IL-1 receptor complex and inhibitively phosphorylates it
**Inhibition**				
Bcl2	1.12	B cell lymphoma 2		Outer mitochondrial membrane protein that inhibits apoptosis and autophagy; may attenuate inflammation by impairing inflammasome formation
Chuk	0.868	Conserved helix-loop-helix ubiquitous kinase	Inhibitor of NFκB kinase subunit alpha (IKKα)	Part of the IKK complex that inhibits IκBα and permits NFκB nuclear localization
Clu	1.62	Clusterin	Apolipoprotein J; Ku70-binding protein 1 (KUB1)	Extracellular chaperone that promotes clearance of inflammation and injury-induced immune complexes; protects cells against apoptosis and against cytolysis by complement
Cyld	0.997	Cylindromatosis lysine 63 deubiquitinase		Inhibits NFκB activation by deubiquitinating upstream signaling factors; inhibits Wnt signaling; restricts polyubiquitination of RIPK1 and -2, thereby limiting necroptosis
Dusp6	1.46	Dual specificity phosphatase 6		Inhibitively phosphorylates ERK1 and 2

Foxp3	1.5	Forkhead box P3	DIETER	Master TF for Tregs; represses expression of Il2 and Ifng; activates expression of Tnfrsf18, Il2ra, and Ctla4
Irak3	1.31	Interleukin-1 receptor-associated kinase 3		Adaptor protein that negatively regulates TLR signaling; predominantly expressed in monocytes and macrophages
Nfkb1	0.928	Nuclear factor kappa B subunit 1	p105/p50	One of the NFκB family TFs; inhibits inflammation
Nlrc5	0.845	NLR family CARD domain containing 5	NOD27	Inhibits NFκB and type I IFN signaling pathways; may also regulate the type II IFN signaling pathway
Tank	0.916	TRAF family member-associated NFκB activator		Inhibitory protein that sequesters TRAFs in the cytoplasm, constitutively binds TBK1, and serves as a negative regulator of NFκB
**Interferon Response**				
Bst2	0.822	Bone marrow stromal cell antigen 2	Tethrin; CD317	IFN-induced antiviral factor that blocks budding of enveloped viruses by directly tethering nascent virions to the membranes of infected cells
Ifih1	1.09	Interferon induced with helicase C domain 1	Helicard; melanoma differentiation-	PRR for cytoplasmic dsRNA; upon target recognition, associates with MAVS to activate TNK1 and IKBKE, which

			associated protein 5 (MDA5)	phosphorylate IRF3 and -7, which, in turn, activate transcription of IFNα and -β
Ifitm1	3.2	Interferon-induced transmembrane protein 1	CD225	IFN-induced antiviral protein implicated in cell adhesion and control of cell growth and migration
Ifitm2	2.63	Interferon-induced transmembrane protein 2		IFN-induced antiviral protein which inhibits the entry of viruses to the host cell cytoplasm; induces cell cycle arrest and mediates p53-independent apoptosis through caspase activation
Ifnar1	1.12	Interferon-alpha/beta receptor alpha chain		Component of the receptor for type I IFNs, binding of which activates the JAK-STAT pathway
Ifngr1	1.45	Interferon gamma receptor 1	CD119	One of the two components of the IFNγ receptor; stimulates activation of the JAK/STAT signaling pathway
Irf7	1.22	Interferon regulatory factor 7		Key transcriptional regulator of type I IFN-dependent immune responses; promotes transcription of IFNα and -β

Irgm2	1.39	Immunity-related GTPase family M member 2	Interferon-inducible protein 1 (IFI1)	Function not fully known, but most likely regulates autophagy and pro-inflammatory cytokine production
Mefv	2.88	Mediterranean fever	Marenostrin; pyrin	Involved in the regulation of innate immunity and the inflammatory response in response to IFNγ; both stimulates and restrains the inflammasome; also acts as a mediator of pyroptosis
Tbk1	1.22	TANK-binding kinase 1		Coordinates the activation of IRF3 and NFκB and induction of type I IFNs
Tmem173	0.811	Transmembrane protein 173	Stimulator of interferon genes (STING)	Adaptor protein in type I IFN signaling; activates STAT6 and IRF3 through TBK1 to induce type I IFN production
Tyk2	1.54	Tyrosine kinase 2	JTK1	Plays both structural and catalytic roles in numerous cytokines and interferons signaling; associates with cytokine and growth factor receptors and activate STAT family members including STAT1, STAT3, STAT4, or STAT6
**IRAKs & TRAFs**				

Irak1	1.2	Interleukin-1 receptor-associated kinase 1		Adaptor protein involved in TLR and IL-1 signaling; recruited to TLRs by MyD88 and phosphorylated by IRAK4; promotes the degradation of TIRAP
Irak2	0.791	Interleukin-1 receptor-associated kinase 2		Adaptor protein involved in TLR and IL-1 signaling
Irak3	1.31	Interleukin-1 receptor-associated kinase 3		Adaptor protein that negatively regulates TLR signaling; predominantly expressed in monocytes and macrophages
Irak4	1.33	Interleukin-1 receptor-associated kinase 4		The primary IRAK family member in mammalian TLR and IL-1 signaling; joins with IRAK2 and MyD88 to form the myddosome complex to activate IRAK1
Traf2	0.8	Tumor necrosis factor receptor-associated factor 2		Adaptor protein required for TNFα-mediated activation of JNK and NFκB
Traf3	0.939	Tumor necrosis factor receptor-associated factor 3		Adaptor protein that acts in the CD40 signaling cascade; induces NFκB and MAPK activation

Traf6	0.707	Tumor necrosis factor receptor-associated factor 6		Adaptor protein that acts in the CD40 signaling cascade; promotes inflammation, IL-6, and TNFα
**JAK-STAT Pathway**				
Jak1	0.834	Janus kinase 1		Essential tyrosine kinase involved signal transduction in type I and II cytokines and IFNs
Jak2	1.33	Janus kinase 2		Tyrosine kinase that participates in IFN and IL6ST signaling cascades
Stat1	1.24	Signal transducer and activator of transcription 1		Transcriptional activator that mediates cellular responses to IFNs, cytokines, and other growth factors
Stat3	1.26	Signal transducer and activator of transcription 3		Transcriptional activator of genes involved in cell growth and apoptosis; activated by JAKs
Stat6	1.22	Signal transducer and activator of transcription 6		Essential TF for T_H2 $CD4^+$ T cell and macrophage function and M2 macrophage polarization
**Kinases**				
Btk	1.24	Bruton's tyrosine kinase		Crucial kinase in B cell receptor signal transmission and B cell activation

Hck	1.62	Hematopoietic cell kinase		Src family tyrosine kinase that mediates secretory lysosome mobilization, degranulation, and activation of NADPH oxidase
Jak1	0.834	Janus kinase 1		Essential tyrosine kinase involved signal transduction in type I and II cytokines and IFNs
Jak2	1.33	Janus kinase 2		Tyrosine kinase that participates in IFN and IL6ST signaling cascades
Lyn	1.88	Lck/Yes-related novel kinase		Src family tyrosine kinase that potentiates signaling from the B cell receptor and CD40
Pik3cg	1.44	Phosphatidylinositol-4,5-bisphosphate 3-kinase catalytic subunit gamma isoform		A subunit of PI3K; modulates leukocyte chemotaxis to inflammatory sites and in response to chemoattractant agents
Ripk2	1.37	Receptor-interacting serine/threonine-protein kinase 2		RIP kinase that potentiates signals downstream of NOD1 and -2, leading to NFκB activation; promotes BCL10 phosphorylation and subsequent NFκB activation following TCR engagement

Syk	1.23	Spleen-associated tyrosine kinase		Critical kinase that transmits signals from the TCR and BCR
Tbk1	1.22	TANK-binding kinase 1		Coordinates the activation of IRF3 and NFκB and induction of type I IFNs
Tyk2	1.54	Tyrosine kinase 2	JTK1	Plays both structural and catalytic roles in numerous cytokines and interferons signaling; associates with cytokine and growth factor receptors and activate STAT family members including STAT1, STAT3, STAT4, or STAT6
		**Lysosomal Activity**		
Ctss	1.42	Cathepsin S		Lysosomal protease that participates in processing of Ag by MHC class II
Hck	1.62	Hematopoietic cell kinase		Src family tyrosine kinase that mediates secretory lysosome mobilization, degranulation, and activation of NADPH oxidase
		**Macrophage Function**		
Casp8	0.914	Caspase 8		Cysteine-aspartic acid protease; cleaves and activates effector caspases CASP3, -4, -6, -7, -9, and -10; initiates pyroptosis through cleavage of gasdermin-D; mediates

				noncanonical cleavage of IL-1β in DCs and macrophages
Ccl3	1.01	C-C motif chemokine ligand 3	Macrophage inflammatory protein 1α (MIP1α)	Chemoattractant ligand for CCR1, -4, and -5
Ccr2	2.01	C-C motif chemokine receptor 2	CD192	Receptor for CCL2, a monocyte-specific chemokine
Cd14	2.78	Cluster of differentiation 14		PRR that recognizes LPS; mostly found on macrophages
Cebpb	2.13	CCAAT/enhancer-binding protein beta		Critical macrophage TF that promotes expression of several acute-phase and inflammatory cytokine genes, including Il6
Cklf	1.14	Chemokine-like factor		Chemoattractant for monocytes, neutrophils, and lymphocytes
Clec5a	2.43	C-Type lectin domain family 5, member a	Myeloid DAP12-associating lectin-1	Critical macrophage receptor for dengue virus serotypes 1-4; positive regulator of osteoclastogenesis
Csf1	1.75	Macrophage colony-stimulating factor 1		Cytokine that promote activation and survival of monocytes

Csf1r	1.94	Macrophage colony-stimulating factor 1 receptor	CD115	Receptor for CSF1; promotes release of inflammatory cytokines in response to IL-34 and CSF1
Irak3	1.31	Interleukin-1 receptor-associated kinase 3		Adaptor protein that negatively regulates TLR signaling; predominantly expressed in monocytes and macrophages
Mif	1.07	Macrophage migration inhibitory factor	L-dopachrome tautomerase	Pro-inflammatory cytokine that promotes macrophage function through suppression of anti-inflammatory effects of glucocorticoids
Slc11a1	2.01	Natural resistance-associated macrophage protein 1		Macrophage-specific metal ion transporter; uptakes divalent metal cations to neutralize ROSs
Stat6	1.22	Signal transducer and activator of transcription 6		Essential TF for T_H2 $CD4^+$ T cell and macrophage function and M2 macrophage polarization
**Mast Cell Function**				
Lgals3	1.99	Galectin 3		Galactose-specific lectin that binds IgE; involved in acute inflammatory responses, including neutrophil activation and adhesion, chemoattraction of monocytes

				macrophages, opsonization of apoptotic neutrophils, and activation of mast cells
Ms4a2	1.57	Membrane spanning 4-domains A2	Fc fragment of IgE, high affinity I, receptor subunit beta (FCER1B)<br><br>Immunoglobulin E receptor (IGER)	High affinity receptor that binds to the Fc region of IgE; required for the full mast cell response; also mediates the secretion of important lymphokines
**MAP Kinase Signaling**				
Mapk1	0.938	Mitogen-activated protein kinase 1	Extracellular signal-regulated kinase 2 (ERK2)	Serine/threonine kinase that acts as an essential component of the MAP kinase signal transduction pathway
Mapk3	1.57	Mitogen-activated protein kinase 3	Extracellular signal-regulated kinase 1 (ERK1)	Serine/threonine kinase that acts as an essential component of the MAP kinase signal transduction pathway
Mapk8	0.903	Mitogen-activated protein kinase 8	c-Jun N-terminal kinase 1 (JNK1); Stress-activated protein kinase 1c (SAPK1)	Serine/threonine-protein kinase involved in various processes such as cell proliferation, differentiation, migration, transformation and programmed cell death; phosphorylates a number of TFs, primarily components of AP-1 such as JUN, JDP2, and ATF2, thus regulating AP-1 transcriptional activity; promotes stressed cell apoptosis

				by phosphorylating key regulatory factors including p53/TP53 and Yes-associates protein YAP1; required for T_H1 differentiation
Mapk14	1.42	Mitogen-activated protein kinase 14		One of the four p38 MAPKs; key kinase in the cascades of cellular responses evoked by extracellular stimuli such as proinflammatory cytokines
Map2k1	0.824	Dual specificity mitogen-activated protein kinase kinase 1	MAPK/ERK kinase 1 (MEK1)	Essential component of the MAP kinase signal transduction pathway; participates in numerous biological functions, including cell growth, adhesion, survival, differentiation, transcription, metabolism, and cytoskeletal remodeling
Map2k2	1.22	Mitogen-activated protein kinase kinase 2	MAPK/ERK kinase 2 (MEK2)	Catalyzes the concomitant phosphorylation of a threonine and a tyrosine residue in a TQY sequence located in MAP kinases; activates ERK1 and -2
Map2k4	1.03	Mitogen-activated protein kinase kinase 4	MAPK/ERK kinase 4 (MEK4); c-Jun N-terminal kinase kinase 1 (JNKK1)	Dual specificity protein kinase that acts as an essential component of the stress-activated protein/c-Jun N-terminal kinase (SAP/JNK) signaling pathway

Map3k1	0.853	Mitogen-activated protein kinase kinase kinase 1		Serine/threonine kinase that activates the ERK and JNK kinase pathways by phosphorylation of MAP2K1 and MAP2K4; also activates CHUK and IKBKB, the central protein kinases of the NFκB pathway
Map3k5	1.54	Mitogen-activated protein kinase kinase kinase 5	Apoptosis signal-regulating kinase 1	Essential component of the MAP kinase signal transduction pathway; mediates signaling for determination of cell fate such as differentiation and survival; plays a crucial role in the apoptosis signal transduction pathway through mitochondria-dependent caspase activation; required for the innate immune response; mediates signal transduction of receptor-mediated inflammatory signals, such as TNF or LPS
Map3k7	0.844	Mitogen-activated protein kinase kinase kinase 7	TGFβ-activated kinase (TAK1)	Signal transducer downstream of TGFβ and BMP; controls a variety of cell functions, including transcription regulation and apoptosis
Mapkapk2	1.49	MAP kinase-activated protein kinase 2		Serine/threonine-protein kinase involved in cytokine production, endocytosis, reorganization of the cytoskeleton, cell migration, cell cycle control, chromatin

				remodeling, DNA damage response, and transcriptional regulation
Tab1	0.88	TGF-beta activated kinase 1 binding protein 1		Activates TAK1 kinase; mediates various intracellular signaling pathways, such as those induced by TGFβ, IL-1, and WNT-1
**Metabolism**				
Cd36	2.24	Cluster of differentiation 36	Fatty acid translocase (FAT)	Class B scavenger receptor that mediates fatty acid uptake
Map2k1	0.824	Dual specificity mitogen-activated protein kinase kinase 1	MAPK/ERK kinase 1 (MEK1)	Essential component of the MAP kinase signal transduction pathway; participates in numerous biological functions, including cell growth, adhesion, survival, differentiation, transcription, metabolism, and cytoskeletal remodeling
S100a8	2.82	S100 calcium-binding protein A8	Calgranulin A	Calcium- and zinc-binding protein involved in pro-inflammatory, antimicrobial, oxidant-scavenging and apoptosis-inducing activities; can induce neutrophil chemotaxis, adhesion, phagocytosis, and degranulation; predominantly found as calprotectin (S100A8/A9) which has a wide plethora of intra- and extracellular functions, including adhesion, apoptosis,

				autophagy, cytoskeletal remodeling, cytokine production, chemotaxis, migration, inflammation, arachidonic acid metabolism, oxidant-scavenging, and PRR signaling
**Migration/Motility**				
Angpt1	1.72	Angiopoietin 1		Secreted glycoprotein that binds and activates the TEK/TIE2 receptor by inducing its dimerization and tyrosine phosphorylation; involved in angiogenesis, endothelial cell survival, proliferation, migration, adhesion, cell spreading, and reorganization of the actin cytoskeleton
App	2.46	Amyloid-beta precursor protein		Cell surface receptor and transmembrane precursor protein that is cleaved by secretases to form a number of peptides; involved in cell mobility, copper homeostasis, and oxidative stress
Cd97	1.35	Cluster of differentiation 97	BL-Ac[F2]	GPCR that promotes granulocyte adhesion and migration; activates T cells via binding to CD55; stimulates angiogenesis through binding integrin counterreceptors on endothelial cells

S100a8	2.82	S100 calcium-binding protein A8	Calgranulin A	Calcium- and zinc-binding protein involved in pro-inflammatory, antimicrobial, oxidant-scavenging and apoptosis-inducing activities; can induce neutrophil chemotaxis, adhesion, phagocytosis, and degranulation; predominantly found as calprotectin (S100A8/A9) which has a wide plethora of intra- and extracellular functions, including adhesion, apoptosis, autophagy, cytoskeletal remodeling, cytokine production, chemotaxis, migration, inflammation, arachidonic acid metabolism, oxidant-scavenging, and PRR signaling
**Neutrophil Function**				
Cklf	1.14	Chemokine-like factor		Chemoattractant for monocytes, neutrophils, and lymphocytes
Cxcr2	2.38	C-X-C motif chemokine receptor 2	CD182; IL-8 receptor B	Receptor for IL-8 and CXCL3; powerful chemoattractant for neutrophils
Fpr2	2.91	Formyl peptide receptor 2	Lipoxin A4 receptor	Low affinity receptor for N-formyl-methionyl peptides; activates neutrophils

Lcn2	2.98	Lipocalin 2	Neutrophil gelatinase-associated lipocalin (NGAL)	Neutrophil-secreted factor that sequesters iron-containing siderophores; also functions as a growth factor
Lgals3	1.99	Galectin 3		Galactose-specific lectin that binds IgE; involved in acute inflammatory responses, including neutrophil activation and adhesion, chemoattraction of monocytes macrophages, opsonization of apoptotic neutrophils, and activation of mast cells
Ncf4	1.98	Neutrophil cytosolic factor 4	SH3 and PX domain-containing protein 4 (SH3PXD4)	Cytosolic regulatory component of the superoxide-producing phagocyte NADPH-oxidase, a multicomponent enzyme system important for host defense
S100a8	2.82	S100 calcium-binding protein A8	Calgranulin A	Calcium- and zinc-binding protein involved in pro-inflammatory, antimicrobial, oxidant-scavenging and apoptosis-inducing activities; can induce neutrophil chemotaxis, adhesion, phagocytosis, and degranulation; predominantly found as calprotectin (S100A8/A9) which has a wide plethora of intra- and extracellular functions, including adhesion, apoptosis, autophagy, cytoskeletal remodeling, cytokine production, chemotaxis,

				migration, inflammation, arachidonic acid metabolism, oxidant-scavenging, and PRR signaling
**NFκB Signaling**				
Bcl10	1.26	B cell lymphoma/leukemia 10		Activates NFκB via ubiquitination of IKKγ
Ikbkb	1.02	Inhibitor of nuclear factor kappa B kinase subunit beta		Part of the IKK complex that inhibits IκBα and permits NFκB nuclear localization
Nfkb1	0.928	Nuclear factor kappa B subunit 1	p105/p50	One of the NFκB family TFs; inhibits inflammation
Nfkb2	0.857	Nuclear factor kappa B subunit 2	p100/p52	One of the NFκB family TFs; major driver of inflammation
Nfkbia	1.19	Nuclear factor kappa B inhibitor alpha		Inhibits activity of REL dimers by masking of their nuclear localization signals
Rela	1	Avian reticuloendotheliosis viral oncogene homolog A	p65	One of the NFκB family TFs; major driver of inflammation

Ripk2	1.37	Receptor-interacting serine/threonine-protein kinase 2		RIP kinase that potentiates signals downstream of NOD1 and -2, leading to NFκB activation; promotes BCL10 phosphorylation and subsequent NFκB activation following TCR engagement
**Pattern Recognition Receptors**				
Cd14	2.78	Cluster of differentiation 14		PRR that recognizes LPS; mostly found on macrophages
Cd180	0.93	Cluster of differentiation 180		Heterodimeric binding partner of Ly86 that participates in LPS binding in APCs
Clec4a2	2.18	C-type lectin domain family 4 member A2		PRR that, upon binding mannose or fucose, is endocytosed and processed in the Ag presentation pathway
Clec5a	2.43	C-Type lectin domain family 5, member a	Myeloid DAP12-associating lectin-1	Critical macrophage receptor for dengue virus serotypes 1-4; positive regulator of osteoclastogenesis
Clec7a	2.29	C-Type lectin domain family 7, member a	Dectin-1	PRR specific for β-1,3- and β-1,6-linked glucans from fungi and plants; necessary for the TLR2-mediated inflammatory response and for TLR2-mediated activation of NF-κB

Ddx58	0.956	DExD/H-box helicase 58	Retinoic acid-inducible gene I (RIG-I)	Cytoplasmic PRR that recognizes dsRNA; can promote T cell-independent B cell activation; uses MAVS as an adaptor
Ifih1	1.09	Interferon induced with helicase C domain 1	Helicard; melanoma differentiation-associated protein 5 (MDA5)	PRR for cytoplasmic dsRNA; upon target recognition, associates with MAVS to activate TNK1 and IKBKE, which phosphorylate IRF3 and -7, which, in turn, activate transcription of IFNα and -β
Ly86	1.15	Lymphocyte antigen 86	Myeloid differentiation factor 1 (MD-1)	Heterodimeric binding partner of CD180 that participates in LPS binding in APCs
Ly96	2.07	Lymphocyte antigen 96	Myeloid differentiation factor 2 (MD-2)	Heterodimeric binding partner of TLR4 that participates in LPS binding
Myd88	1.97	Myeloid differentiation primary response 88		Key adaptor in the TLR signaling pathways; interacts with all TLRs except TLR3; activates NFκB and IRFs
Nod2	1.2	Nucleotide-binding oligomerization domain containing 2		PRR specific for muramyl dipeptide (MDP); upon binding to its ligand, recruits RIPK2 and triggers MAPK and NFκB signaling

Nlrp3	2.42	NACHT domain-, leucine-rich repeat-, and PYD-containing protein 3	Cryopyrin	PRR with a wide diversity of recognized targets that activates the NLRP3 inflammasome consisting of NLRP3, PYCARD, and caspase-1/-8
S100a8	2.82	S100 calcium-binding protein A8	Calgranulin A	Calcium- and zinc-binding protein involved in pro-inflammatory, antimicrobial, oxidant-scavenging and apoptosis-inducing activities; can induce neutrophil chemotaxis, adhesion, phagocytosis, and degranulation; predominantly found as calprotectin (S100A8/A9) which has a wide plethora of intra- and extracellular functions, including adhesion, apoptosis, autophagy, cytoskeletal remodeling, cytokine production, chemotaxis, migration, inflammation, arachidonic acid metabolism, oxidant-scavenging, and PRR signaling
Ticam2	1.87	TIR domain-containing adaptor molecule 2		Sorting adapter in various innate immune signaling cascades; bridges TLR2 and MyD88

Tirap	2	TIR domain-containing adaptor protein		Adaptor protein involved in TLR2 and TLR4 signaling; acts via IRAK2 and TRAF6, leading to the activation of NFκB, MAPK1, MAPK3 and JNK, and resulting in cytokine secretion and the inflammatory response; positively regulates the production of TNFα and IL-6.
Tlr2	2.27	Toll-like receptor 2	CD282	Surface PRR that binds to various lipid-containing PAMPs
Tlr4	2.38	Toll-like receptor 4	CD284	Surface PRR that recognizes LPS; pairs with LY96 and CD14; acts via MYD88, TIRAP, and TRAF6, leading to NFκB activation, cytokine secretion, and the inflammatory response; in complex with TLR6, promotes sterile inflammation in monocytes/macrophages in response to oxLDL or amyloid-β 42
Tlr6	2.43	Toll like receptor 6	CD286	Surface PRR that recognizes diacylated and triacylated lipopeptides
Tlr7	2.49	Toll-like receptor 7	CD287	Endosomic PRR that recognizes ssRNA
Tlr8	2.26	Toll-like receptor 8	CD288	Endosomic PRR that recognizes ssRNA
**Phagocytosis**				
Itgam	1.77	Integrin alpha M	CD11b	Pairs with CD18 to forms Mac-1 aka complement receptor 3; mediates

				leukocyte activation, adhesion, chemotaxis, migration, phagocytosis, and cell-mediated cytotoxicity; serves as a macrophage marker
Ncf4	1.98	Neutrophil cytosolic factor 4	SH3 and PX domain-containing protein 4 (SH3PXD4)	Cytosolic regulatory component of the superoxide-producing phagocyte NADPH-oxidase, a multicomponent enzyme system important for host defense
S100a8	2.82	S100 calcium-binding protein A8	Calgranulin A	Calcium- and zinc-binding protein involved in pro-inflammatory, antimicrobial, oxidant-scavenging and apoptosis-inducing activities; can induce neutrophil chemotaxis, adhesion, phagocytosis, and degranulation; predominantly found as calprotectin (S100A8/A9) which has a wide plethora of intra- and extracellular functions, including adhesion, apoptosis, autophagy, cytoskeletal remodeling, cytokine production, chemotaxis, migration, inflammation, arachidonic acid metabolism, oxidant-scavenging, and PRR signaling
**ROS Generation & Protection**				

Cybb	1.8	Cytochrome b-245 heavy chain	Nox2	Part of the NADPH oxidase process; generates superoxides
Ncf4	1.98	Neutrophil cytosolic factor 4	SH3 and PX domain-containing protein 4 (SH3PXD4)	Cytosolic regulatory component of the superoxide-producing phagocyte NADPH-oxidase, a multicomponent enzyme system important for host defense
Txnip	1.1	Thioredoxin interacting protein		Thiol-oxidoreductase; protects cells from oxidative stress by inhibiting thioredoxin
**Stress Response**				
App	2.46	Amyloid-beta precursor protein		Cell surface receptor and transmembrane precursor protein that is cleaved by secretases to form a number of peptides; involved in cell mobility, copper homeostasis, and oxidative stress
Mapk8	0.903	Mitogen-activated protein kinase 8	c-Jun N-terminal kinase 1 (JNK1); Stress-activated protein kinase 1c (SAPK1)	Serine/threonine-protein kinase involved in various processes such as cell proliferation, differentiation, migration, transformation and programmed cell death; phosphorylates a number of TFs, primarily components of AP-1 such as JUN, JDP2, and ATF2, thus regulating AP-1 transcriptional activity; promotes stressed cell apoptosis by phosphorylating key regulatory factors

				including p53/TP53 and Yes-associates protein YAP1; required for T_H1 differentiation
Map2k4	1.03	Mitogen-activated protein kinase kinase 4	MAPK/ERK kinase 4 (MEK4); c-Jun N-terminal kinase kinase 1 (JNKK1)	Dual specificity protein kinase that acts as an essential component of the stress-activated protein/c-Jun N-terminal kinase (SAP/JNK) signaling pathway
Txnip	1.1	Thioredoxin interacting protein		Thiol-oxidoreductase; protects cells from oxidative stress by inhibiting thioredoxin
**T Cell Function**				
Cd28	1.04	Cluster of differentiation 28		Essential T cell co-receptor that enhances T cell activation, proliferation, cytokine production, and survival; binds to CD80 and CD86
Cd4	0.937	Cluster of differentiation 4		Signature helper T cell marker; binds to MHC class II and provides necessary costimulation for T cell activation
Cd97	1.35	Cluster of differentiation 97	BL-Ac[F2]	GPCR that promotes granulocyte adhesion and migration; activates T cells via binding to CD55; stimulates angiogenesis through binding integrin counterreceptors on endothelial cells

Pik3cg	1.44	Phosphatidylinositol-4,5-bisphosphate 3-kinase catalytic subunit gamma isoform		A subunit of PI3K; modulates leukocyte chemotaxis to inflammatory sites and in response to chemoattractant agents
**Transcription Factors & Coactivators**				
Atf1	0.973	Activating transcription factor 1		Basic leucine zipper TF regulating the expression of genes related to growth and survival
Atf2	1.13	Activating transcription factor 2	Cyclic AMP-responsive element-binding protein 2 (CREB2)	Regulates transcription of various genes involved in anti-apoptosis, cell growth, and DNA damage response; in the nucleus, contributes to global transcription and the DNA damage response, in addition to specific transcriptional activities that are related to cell development, proliferation and death; in the cytoplasm, impairs mitochondrial membrane potential, inducing mitochondrial leakage and promoting cell death; phosphorylated form (mediated by ATM) plays a role in the DNA damage response

Cebpb	2.13	CCAAT/enhancer-binding protein beta		Critical macrophage TF that promotes expression of several acute-phase and inflammatory cytokine genes, including Il6
Crebbp	0.949	CREB binding protein	Lysine acetyltransferase 3A (KAT3A)	Binds specifically to phosphorylated CREB and enhances its transcriptional activity toward cAMP-responsive genes; also acetylates histones, giving a specific tag for transcriptional activation
Fos	2.49	Finkel-Biskel-Jenkins osteosarcoma proto-oncogene homolog		Basic leucine zipper TF that dimerizes with JUN proteins to form the AP-1 TF complex
Foxp3	1.5	Forkhead box P3	DIETER	Master TF for Tregs; represses expression of Il2 and Ifng; activates expression of Tnfrsf18, Il2ra, and Ctla4
Nfkb1	0.928	Nuclear factor kappa B subunit 1	p105/p50	One of the NFκB family TFs; inhibits inflammation
Nfkb2	0.857	Nuclear factor kappa B subunit 2	p100/p52	One of the NFκB family TFs; major driver of inflammation
Rela	1	Avian reticuloendothelios	p65	One of the NFκB family TFs; major driver of inflammation

		is viral oncogene homolog A		
Stat1	1.24	Signal transducer and activator of transcription 1		Transcriptional activator that mediates cellular responses to IFNs, cytokines, and other growth factors
Stat3	1.26	Signal transducer and activator of transcription 3		Transcriptional activator of genes involved in cell growth and apoptosis; activated by JAKs
Stat6	1.22	Signal transducer and activator of transcription 6		Essential TF for T_H2 $CD4^+$ T cell and macrophage function and M2 macrophage polarization
Zbp1	1.05	Z-DNA binding protein 1	Tumor stroma and activated macrophage protein DLM-1	Key innate sensor that recognizes and binds Z-RNA structures, which are produced by a number of viruses, and induces type-I IFN production; key activator of cellular necroptosis; promotes IL-1α induction in an NLRP3-inflammasome-independent manner
**Ubiquitin Regulation**				
Bcl10	1.26	B cell lymphoma/leukemia 10		Activates NFκB via ubiquitination of IKKγ

Cyld	0.997	Cylindromatosis lysine 63 deubiquitinase		Inhibits NFκB activation by deubiquitinating upstream signaling factors; inhibits Wnt signaling; restricts polyubiquitination of RIPK1 and -2, thereby limiting necroptosis
Itch	0.947	Itchy E3 ubiquitin protein ligase		Participates with TNFAIP3 in a ubiquitin-editing complex that marks components of inflammatory signaling pathways such as JUNB and CXCR4 for degradation
Tnfaip3	1.38	Tumor necrosis factor, alpha-induced protein 3		Ubiquitin-editing enzyme that complexes with ITCH to degrade inflammatory signaling components in the TNF, IL1, and TLR pathways; targets TRAF2, TRAF6, and IKK
Ubc	0.99	Polyubiquitin C		Serves various roles, including immate immunity, DNA repair, and stimulation of autophagy and the proteasomal response
**Vesicular Trafficking**				
Atg5	1.26	Autophagy related 5		Pairs with ATG12 to promote the extension of the phagophoric membrane in autophagic vesicles
